# Gene expression patterns of two dominant tallgrass prairie species differ in response to warming and altered precipitation

**DOI:** 10.1038/srep25522

**Published:** 2016-05-13

**Authors:** Melinda D. Smith, Ava M. Hoffman, Meghan L. Avolio

**Affiliations:** 1 Department of Biology and Graduate Degree Program in Ecology, Colorado State University, Fort Collins, CO 80523, USA; 2 National Socio-Environmental Synthesis Center, Annapolis, MD, 21401, USA

## Abstract

To better understand the mechanisms underlying plant species responses to climate change, we compared transcriptional profiles of the co-dominant C_4_ grasses, *Andropogon gerardii* Vitman and *Sorghastrum nutans* (L.) Nash, in response to increased temperatures and more variable precipitation regimes in a long-term field experiment in native tallgrass prairie. We used microarray probing of a closely related model species (*Zea mays*) to assess correlations in leaf temperature (T_leaf_) and leaf water potential (LWP) and abundance changes of ~10,000 transcripts in leaf tissue collected from individuals of both species. A greater number of transcripts were found to significantly change in abundance levels with T_leaf_ and LWP in *S. nutans* than in *A. gerardii*. *S. nutans* also was more responsive to short-term drought recovery than *A. gerardii*. Water flow regulating transcripts associated with stress avoidance (e.g., aquaporins), as well as those involved in the prevention and repair of damage (e.g., antioxidant enzymes, HSPs), were uniquely more abundant in response to increasing T_leaf_ in *S. nutans*. The differential transcriptomic responses of the co-dominant C_4_ grasses suggest that these species may cope with and respond to temperature and water stress at the molecular level in distinct ways, with implications for tallgrass prairie ecosystem function.

Predicted climate change includes increased average temperatures and more variable precipitation regimes^1^, which should result in increased water and temperature stress for many plant communities. Such chronic alterations in environmental conditions are expected to lead to phenotypic adaptations at the individual level^2^,^3^. For example, increased interannual variability in rainfall has been shown to select for greater morphological and physiological plasticity, which may result in evolutionary change^4^. Plant phenotypic responses to changing environmental conditions are achieved through the regulation of gene expression at transcriptional and post-transcriptional levels, which results in changes in metabolism and physiology. Thus, linking phenotype and gene expression could provide a more sensitive indicator of response to altered environmental conditions and stress. Furthermore, evaluating functional responses of plants at the genetic and biochemical level will increase our understanding of underlying genetic bases of responses that limit growth and survivorship, which can be related to fitness variation, selection and adaptation^5^,^6^. Individual responses also may cascade to affect community and ecosystem structure and function, particularly if the focal species is dominant in the system^2^,^7^.

Until recently, the molecular basis of plant responses to changing environmental conditions and stress in natural systems was relegated to an unknown ‘black box’^8^. However, genomic tools can elucidate molecular mechanisms and assess their importance in a real-world context^8^,^9^,^10^,^11^,^12^. Microarray profiling provides an analytical tool by which expression of thousands of genes can be studied simultaneously and has been used to monitor global gene expression in response to abiotic stresses in a number of higher plants^13^,^14^,^15^,^16^. However, most of these studies have focused on model species for which cDNA microarrays are readily available (e.g., Arabidopsis, maize, *Populus*, rice, and soybean) and have imposed relatively extreme conditions over short time frames and under controlled conditions (e.g., plants grown in pots exposed to drought or seedlings experiencing short-term stress). These studies provide insights into transcriptional profile changes involved in responses to various stresses and contribute to our understanding of the function of related genes. However, it is unclear how findings from these studies apply to non-model plants in natural systems. Environmental changes in natural systems may be more subtle with stress accumulating over time (allowing for acclimation). Plants in these systems are also exposed to greater variation in environmental conditions, as well as many other factors (e.g., competition, herbivory). All of these differences may alter the magnitude of variation in gene expression observed in more controlled settings^8^. Thus, if we are to improve our mechanistic understanding of plant responses to altered environmental conditions associated with climate change, a key step forward is to conduct genomic studies of non-model plant species under ecologically relevant field conditions.

Incorporating transcript profiling in field studies has been hampered by the lack of genomic resources available for non-model species^10^, although new resources are being generated at a rapid pace^17^. Most of the microarrays currently available for higher plants are for model and commercially important species^8^,^17^. While recent high-throughput genotyping tools, such as RAD-seq, negate having to sequence whole genomes, these techniques still require considerable time and/or resources (such as sequencing depth) to develop for non-model species. An alternative approach is to utilize microarrays designed for model species and apply them to closely related non-model species (i.e., heterologous or cross-species hybridization). Although shortcomings, such as loss of species-specific sequences and hybridization artifacts, do exist^18^,^19^, heterologous hybridizations have been successfully used to examine the transcriptomes of non-model plant species in response to a range of environmental stresses^9^,^20^,^21^,^22^,^23^,^24^, including competition^25^,^26^. However, studies of non-model plants in natural field settings are still uncommon (but see^9^,^10^).

Here, we present results from a field study designed to comparatively assess genomic responses of two native C_4_ grass species, *Andropogon gerardii* Vitman (big bluestem) and *Sorghastrum nutans* (L.) Nash (Indian grass), to two aspects of forecast climate change^1^: increased temperatures and increased variability in precipitation regimes. Previous studies reported that *A. gerardii* responded to experimentally altered precipitation regimes by down-regulating expression of genes related to photosynthesis, suggesting a productivity cost of drought stress adaptation or reduced performance due to drought stress^10^. When plants were exposed to both increased temperatures and altered precipitation regimes, *A. gerardii* shifted expression levels of genes in response to thermal stress rather than to water stress^9^. In the tallgrass prairie ecosystem, *A. gerardii* co-dominates with *S. nutans* and the two species interact to influence community and ecosystem structure and function^27^ and responses to climate change^28^,^29^,^30^. Both species are found throughout central and eastern North America, although historically *S. nutans* tended to dominate more mesic (southern) regions^31^. Despite close phylogenetic relatedness^32^,^33^, these two species fluctuate in abundance and differ in their ecophysiological responses to changes in temperature and water availability^30^,^34^,^35^,^36^,^37^. For example, net photosynthesis and stomatal conductance of *A. gerardii* was affected by increases in temperature, whereas these ecophysiological responses were more closely linked with alterations in water availability in *S. nutans*^37^. Ecophysiology of *S. nutans* also was more sensitive to extreme climate manipulations (heat waves) in the field^30^. In other words, under the same conditions, *S. nutans* exhibited a greater decline in ecophysiology compared to *A. gerardii*. Thus, these studies suggest that the two focal species presumed to be functionally similar ecologically may actually differ in important ways in their sensitivity to climate change.

As an initial step in understanding the potential molecular mechanisms underlying the differences in sensitivity observed at the ecophysiological level, we analyzed the transcriptional profiles of individuals of these co-dominant grass species exposed to both increased temperatures and altered precipitation regimes within natural field plots. Over a growing season (including a mid-July drought-recovery event), we used heterologous hybridizations with cDNA microarrays designed for a closely related model species, *Zea mays*, to characterize transcriptional profiles of each species in response to experimentally-induced temperature and water stress in the field. We expected that *Zea mays* would provide a suitable template due to conservation of stress response genes among plants^38^. We hypothesized that transcriptional responses would be differentially up- and down-regulated for different groups of genes, such as those involved in stress response^11^,^16^ and photosynthesis^39^, for *A. gerardii* and *S. nutans*. We expected that absolute differences in transcripts (sensitivity) would differ in response to temperature and water stress over the course of the growing season, with *A. gerardii* being more sensitive to increased temperatures and *S. nutans* being more sensitive to increasing water stress. These findings will help reveal mechanisms by which these two co-dominant species, and ultimately tallgrass prairie communities, respond to climate stress in a natural setting.

## Results

### Variation in T_leaf_ and LWP over the growing season for *A. gerardii* and *S. nutans*

**Figure 1:**
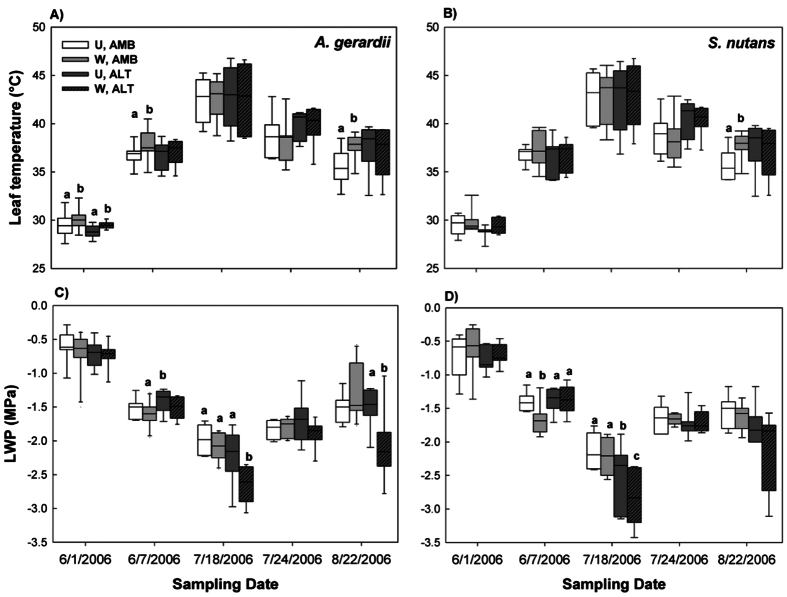
Response of leaf temperature (T_leaf_) and leaf water potential (LWP) of *Andropogon gerardii* (**A,C**) and *Sorghastrum nutans* (**B,D**) to the warming and altered precipitation treatments for five sampling dates during 2006. U: un-warmed; W: warmed; AMB: ambient precipitation; ALT: altered precipitation. Box plots are shown, with median line and upper 75 and lower 25 percentiles. Error bars are the 5 and 95 percentiles of variation. Letters indicate significant difference of T_leaf_ or LWP among different treatments for an individual sampling date (p < 0.05).

*A. gerardii* and *S. nutans* did not differ significantly in the response of leaf surface temperature (T_leaf_; F_1,25_ = 0.62, p = 0.439) or leaf water potential (LWP; F_1,26_ = 1.74, p = 0.198) to the warming or altered precipitation treatments over the duration of the study, although *S. nutans* tended to exhibit lower (more negative) LWP than *A. gerardii* during mid-July. In general, T_leaf_ was lowest at the beginning of the growing season, peaked at the height of the drought (July 18), and then remained intermediate for the remainder of the growing season (Fig. 1A,B). In contrast, LWP became more negative as the growing season progressed (Fig. 1C,D), with LWP lowest in mid-July (July 18). For both species, T_leaf_ of individual plants in warmed plots was generally 1 or 2 °C higher than plants in unwarmed plots (Fig. 1, F_1,6_ = 8.02, p = 0.030 for *A. gerardii*; F_1,17_ = 0.23, p = 0.635 for *S. nutans*). But this difference in T_leaf_ diminished as the growing season progressed and air temperatures increased later in the growing season. Compared with plants exposed to the ambient precipitation treatment (rainfall immediately applied), plants experiencing the altered precipitation treatment (50% increase in time between ambient rainfall events) tended towards lower although not significantly different LWP and higher T_leaf_ (Fig. 1C,D, F_1,13_ = 0.64, p = 0.439 for *A. gerardii*; F_1,40_ = 0.49, p = 0.486 for *S. nutans*). The warming treatment decreased LWP of plants consistently compared to unwarmed plants under the altered precipitation treatment (Fig. 1C,D). As a consequence, the effects of altered precipitation vs. warming treatment were likely confounded, particularly as water became limiting (e.g., July and Aug dates). During the mid-July drought, plants had lower LWP (Fig. 1C,D) and exhibited drought and heat stress related phenotypes (e.g. dry leaf edges, leaf rolling/folding). Application of rain on July 19 to both the ambient and altered treatments resulted in recovery of LWP on July 24 (drought-recovery), but not to levels observed in June (Fig. 1C,D).

### Transcript expression patterns in response to T_leaf_, LWP, and their interaction over the growing season

**Figure 2:**
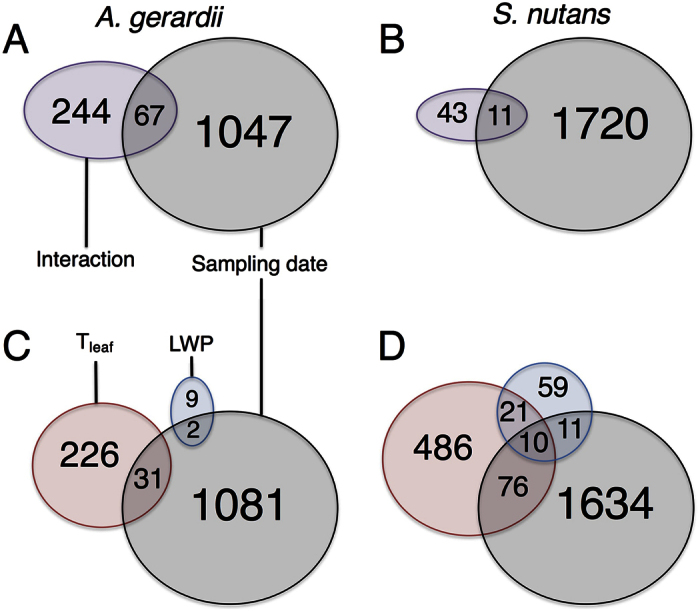
The effect of the combination of variation in leaf temperature (T_leaf_) and leaf water potential (LWP; Interaction), sampling date, and variation in T_leaf_ and LWP on the transcript abundance changes for individuals of *Andropogon gerardii* (**A,C**) and *Sorghastrum nutans* (**B,D**) exposed to the warming and altered precipitation treatments in 2006. The values represent the number of transcripts with significant abundance change (significant positive or negative slopes, q-value < 0.05). Number of transcripts with shared changed abundance between individual effects is shown in the overlapping areas. In all cases, the number of transcripts with abundance change was significantly different between the two species by Pearson χ^2^ tests (p < 0.0001).

High-throughput regression analysis showed that the abundance of a subset of transcripts in leaf tissue of *A. gerardii* and *S. nutans* varied significantly with variation in T_leaf_ and LWP and the interaction of T_leaf_ and LWP across the sampling dates. To detect those transcripts responding separately to variation in T_leaf_ and LWP, we first identified transcripts correlated with the interaction of T_leaf_ and LWP and how many of these also responded to sampling date. Abundance of a small number of transcripts was significantly correlated with the interaction of T_leaf_ and LWP, with significantly more for *A. gerardii* (311) than *S. nutans* (54; χ^2^ = 185.46, p < 0.0001, Fig. 2A,B). Among these transcripts, 67 transcripts in *A. gerardii* and 11 transcripts in *S. nutans* also changed abundance in response to the sampling date (Fig. 2A,B).

After accounting for those transcripts significantly correlated with the interaction of T_leaf_ and LWP, a greater number of transcripts were correlated with variation in T_leaf_ for *S. nutans* (593) than *A. gerardii* (257, χ^2^ = 127.60, p < 0.0001, Fig. 2C,D). A small proportion of transcripts were correlated with variation in LWP for both species (1.5 vs. 0.1%), with again a greater number for *S. nutans* (101) than *A. gerardii* (11, χ^2^ = 64.86, p < 0.0001, Fig. 2C,D). In *A. gerardii*, there was no significant transcript level change correlated with both T_leaf_ and LWP, while the abundance of 2 and 31 transcripts changed by sampling date and variation in LWP and T_leaf_, respectively. In *S. nutans*, 31 transcripts changed their abundance with both LWP and T_leaf_, among which the abundance of 10 of these transcripts also were significantly correlated with sampling date. Eleven and 76 transcripts were significantly correlated with sampling date and variation in LWP and T_leaf_, respectively.

### Differential transcript abundance changes in *A. gerardii* and *S. nutans* in response to variation in T_leaf_, LWP, their interaction and sampling date[Fig f3][Table t1]

**Figure 3 f3:**
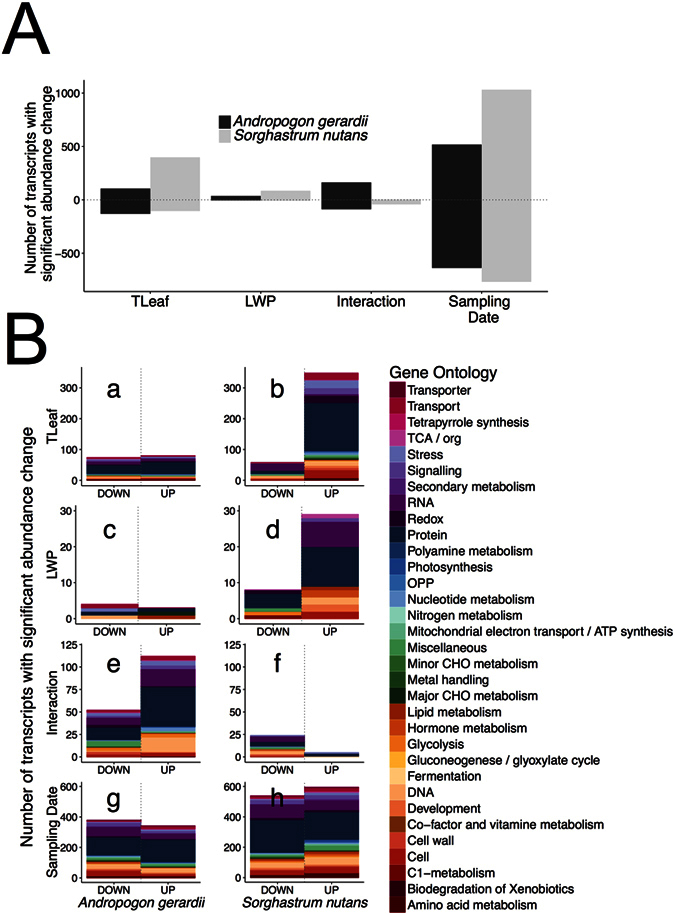
(**A**) Number of transcripts with significant abundance change in response to variation of leaf temperature (T_leaf_), leaf water potential (LWP), their interaction (Interaction) and sampling date for *Andropogon gerardii* (black bars) and *Sorghastrum nutans* (grey bars). In all cases, the number of transcripts with increased or decreased abundance change was significantly different between the two species by Pearson χ^2^ tests (or Fisher’s exact test) (p < 0.001). (**B**) The distribution of transcripts with significant abundance changes in different functional groups according to MapMan gene ontology in *A. gerardii* and *S. nutans* in response to variation in (a,b) leaf temperature (T_leaf_), (c,d) leaf water potential (LWP), (e,f) the combination of T_leaf_ and LWP (Interaction), and (g,h) sampling date. Down = the number of transcripts with decreased abundance (negative slope). Up = the number of transcripts with increased abundance (positive slope) in response to increasing T_leaf_ and decreasing LWP.

1. Leaf temperatureUsing an extension of the MapMan gene ontology from Arabidopsis to maize, transcripts with significant abundance changes were categorized into major functional groups and then further subdivided into specific functions. After accounting for statistical differences due to sampling date through multiple regression, the numbers of transcripts in *A. gerardii* that were correlated with variation in T_leaf_ positively (increased their abundance in response to increased T_leaf_) vs. negatively (decreased their abundance in response to increased T_leaf_) were similar (99 vs. 126, respectively; Fig. 3A). Conversely, more than two-thirds (383 of 486) of the transcripts in *S. nutans* were positively correlated to variation in T_leaf_ (Fig. 3A). Most of these were assigned to MapMan ontology cell, DNA, protein, redox, stress and transport groups (Fig. 3B-b; see Appendix S3, S4). Of the transcripts with changed abundance in response to variation in T_leaf_, seven transcripts with functions in development, nucleotide metabolism, protein synthesis, redox and RNA transcriptional regulation, showed altered abundance in both *A. gerardii* and *S. nutans* (see Appendix S5). A notable difference between these two species is that more transcripts from the functional groups of stress, redox and cell changed their abundance in *S. nutans* compared to that in *A. gerardii* in connection with increasing T_leaf_ (Fig. 3B-a,b; Table 1). These transcripts in *S. nutans* were primarily represented by various heat shock proteins (see Appendix S5). In the redox group, the majority were superoxide dismutase and ascorbate peroxidase genes. All of these transcripts were positively correlated with variation in T_leaf_ (Table 1). Major intrinsic protein (MIP) and Tonoplast intrinsic protein (TIP), and p-/v- ATPases made up the majority of transcripts with changed abundance in the transport group for *S. nutans*, which again were all positively correlated with T_leaf_. All the transcripts in the subgroup of “MIP” were positively correlated with T_leaf_ in *S. nutans*, but not in *A. gerardii* (Table 1, see Appendix S5). Similarly, in the signaling group, two transcripts related to calcium and two related to G-proteins in *A. gerardii* were negatively correlated with T_leaf_, while most transcripts related to calcium, G-protein, receptor kinases and phosphinositides from *S. nutans* were positively correlated with T_leaf_, except for one transcript (phosphinositides), which were negatively correlated with T_leaf_ (Table 1, see Appendix S5). In the photosynthesis group, only three transcripts were correlated with T_leaf_ in *A. gerardii*, one positively and two negatively, while three transcripts were all positively correlated with T_leaf_ in *S. nutans* (Table 1, see Appendix S5).
2. Leaf water potentialChange in abundance of transcripts differed significantly in correlation with lower LWP (i.e., more negative) in both *A. gerardii* vs. *S. nutans* (Fig. 3A). Of the 9 transcripts with significant abundance changes in *A. gerardii*, 5 were negatively correlated with LWP (i.e., *increased* abundance in response to increasing water stress, or lower LWP; Fig. 3B-c), and these were primarily in the functional groups of DNA, lipid metabolism, protein and stress (see Appendix S3, S6). In contrast, more than 80% (51 of 59) of transcripts with changed abundance in *S. nutans* were positively correlated with LWP (i.e., *decreased* abundance in response to increasing water stress, Fig. 3A), with most in the functional groups of protein and RNA, followed by groups, such as cell, development, DNA and hormone metabolism (Fig. 3B-d, see Appendix S4, S6). No transcript was found changed in abundance in both species (see Appendix S6). No transcripts from the functional group of photosynthesis showed response to variation in LWP in either species (Table 1). Two transcripts from the groups of stress and transport were all negatively correlated with LWP in *A. gerardii*. One transcript from the group of signaling was negatively correlated with LWP in *S. nutans* (Table 1, see Appendix S6).
3. Interaction of T_leaf_ and LWPIn *A. gerardii*, about 65% (159 of 244) of transcripts were positively correlated with the interaction of T_leaf_ and LWP, while about 88% of transcripts (38 of 43) were negatively correlated with the interaction of T_leaf_ and LWP in *S. nutans* (Fig. 3A), and only one transcript (Spotid 13430, with 41% sequence similarity to a rice protein, Osr40c1 protein) showed decreased abundance in both species (see Appendix S7). For *A. gerardii*, most transcripts from DNA, protein and RNA synthesis groups were positively correlated with the interaction of LWP and T_leaf_ (Fig. 3B-e, see Appendix S7). The small number of transcripts with changed abundance in *S. nutans* were scattered among different functional groups (e.g., cell wall, DNA, hormone metabolism, protein and RNA), in which most transcripts in the groups of protein and RNA synthesis were decreased in abundance (Fig. 3B-f, see Appendix S7). No transcripts in the functional group of photosynthesis responded to the combined effect of changing LWP and T_leaf_ in *S. nutans*, while one transcript from *A. gerardii* was positively correlated to the combined effect (Table 1). Transcripts from the functional groups such as redox, signaling, stress and transport showed similar regulation with the number of transcripts increased vs. decreased abundance for *S. nutans* (1 vs. 1) and for *A. gerardii* (14 vs. 9) (Table 1, see Appendix S7).
4. Sampling dateFive dates (two June dates, two July dates and one August date) that encompassed most of the growing season (early June to mid-August) were chosen for sampling. A large proportion of variation in transcript abundance was attributable to sampling date (Fig. 3A). Among the transcripts with changed abundance for both species, the number of transcripts with increased or decreased abundance was similar, with 501 vs. 613 for *A. gerardii*, 995 vs. 736 for *S. nutans* (Fig. 3A). Transcripts with changed abundance in response to sampling date were distributed in the similar groups for the two grass species, such as cell, development, DNA, protein, RNA, transport (Fig. 3B-g,h, see Appendix S8). Among the transcripts with changed abundance in response to sampling date, 159 transcripts were common in *A. gerardii* and *S. nutans*, most of which were assigned in the groups of protein, transport, RNA, and DNA (Fig. 3B-g,h, see Appendix S8).

### Response of transcript abundance to short-term drought recovery

**Figure 4:**
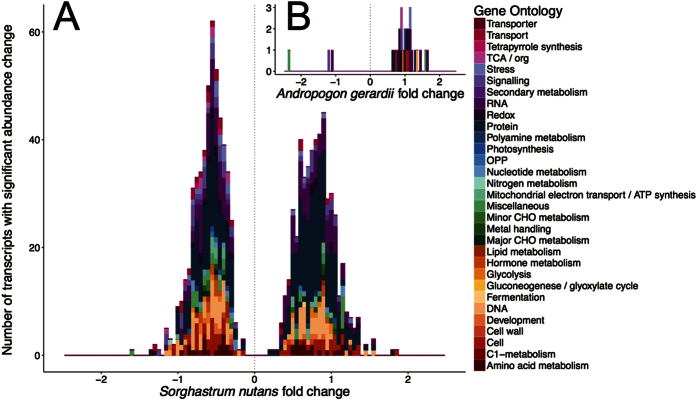
The distribution of transcripts within major functional groups according to MapMan gene ontology with significant abundance change in leaf tissue of (**A**) *Sorghastrum nutans* and (**B**) *Andropogon gerardii* in response to recovery from a mid-July drought period. Positive numbers indicate the number of transcripts with increased abundance (positive slope) and negative numbers indicate the number of transcripts with reduced abundance (negative slope).

Differences in transcript abundance between the two sampling dates in July were used to assess short-term drought-recovery responses of the two grass species because drought stress had been largely relieved in the second sampling date by a significant rainfall event to both the ambient and altered treatments. *A. gerardii* and *S. nutans* recover leaf water status rapidly after exposure to water deficits (Fig. 1), therefore the short recovery window allowed us to minimize other abiotic and biotic variables. For *S. nutans*, 1,639 transcripts (excluding 1,829 transcripts with significant abundance change to the interaction of sampling date and altered precipitation) were significantly changed in abundance between the two sampling dates, with 901 decreased (reduced expression in the second date) and 738 increased in abundance. However, only 42 transcripts (excluding 3,898 transcripts with significant abundance change to the interaction of sampling date and altered precipitation) were significantly changed in abundance between the drought (July 18) and recovery (July 24) dates for *A. gerardii*. Only five showed reduced abundance on the July 24 date compared to the July 18 date, with three transcripts annotated (see Appendix S9). The large number of transcripts with changed abundance in *S. nutans* were distributed in a wide range of functional groups, e.g. cell, DNA, protein, RNA, signaling, and stress groups (Fig. 4A, see Appendix S9). The number of transcripts with increased or decreased abundance was similar except in the groups protein and RNA in which the abundance of more transcripts were increased (Fig. 4A). More transcripts in the groups of redox, stress, and transport were decreased in abundance upon recovery from drought in *S. nutans* (47 vs. 20). These included transcripts associated with ascorbate peroxidase, thioredoxin, HSPs, DNA J-like proteins, and metabolite transporters (see Appendix S9). Transcripts whose abundance varied in *A. gerardii* were among the groups such as cell, protein, RNA and signaling, most of which increased in abundance (Fig. 4B).

Comparison of the transcript level changes during drought-recovery in *A. gerardii* and *S. nutans* to those transcripts significantly correlated with variation in LWP (season-long drought) showed that there were no transcripts that changed abundance similarly under both long-term altered soil moisture conditions and short-term drought recovery in *A. gerardii*. However, abundance levels of 11 transcripts were affected by both long-term drought and short-term recovery in *S. nutans* (see Appendix S9), and these were dispersed across a range of functional groups (see Appendix S9).

### Validation of microarray results using qRT-PCR

Results of the microarray experiment were confirmed by qRT-PCR of nine differentially expressed genes for each species (CB833735, CD651724, CB380843, CB833708, CD568792, DV622645, CD001262, DV621372 for both species and DV551311 for *A. gerardii*; CD001612 for *S. nutans*; see Appendix S2). Consistent results were obtained in the expression pattern (increased or decreased in abundance) of the selected clones. Although some variation in the relative amounts of transcript accumulation was observed between the two techniques, the two were significantly correlated overall (p = < 0.0001, rho = 0.481), within *A. gerardii* samples (p = < 0.0001, rho = 0.559), and within *S. nutans* samples (p = 0.0003, rho = 0.418, see Appendix S10).

## Discussion

As predicted, we found that *A. gerardii* and *S. nutans* differed significantly in the correlation of transcriptional responses with season-long variation in T_leaf_ and LWP, as well as their combined interaction, under field conditions. *S. nutans* had a greater number of transcripts that changed in abundance overall, with the majority of transcripts correlated with date and variation in T_leaf_ (Fig. 2). By contrast, *A. gerardii* showed similar numbers of transcripts changing abundance (both positive and negative) with variation in T_leaf_ and the combined variation in T_leaf_ and LWP, with few responsive to LWP. Interestingly, sampling date had the largest effect on both *A. gerardii* and *S. nutans* transcripts. This indicates that both species modify their gene expression according to changing abiotic and biotic conditions throughout the growing season regardless of the climate change treatments. Although this is difficult to distinguish from leaf maturation and ontogeny in these plants, we tried to control for this by selecting morphologically and developmentally similar individuals (vegetative, each with 3–5 leaves) at each sample date within each treatment. Among transcripts correlated with T_leaf_ and LWP interaction, most of these were reduced in abundance for *S. nutans*, while more were increased in abundance for *A. gerardii* (Fig. 3). During the mid-season drought-recovery period, *A. gerardii* and *S. nutans* again exhibited differential responses, during which the levels of thousands of transcripts varied in *S. nutans*, compared to less than 50 transcripts with significantly altered levels for *A. gerardii* (Fig. 4, Appendix S9), which suggests that *S. nutans* was more sensitive to drought conditions and responded more to alleviation from drought than *A. gerardii*. The finding of differential correlations of transcript responses with T_leaf_ and LWP, both season-long and during a mid-season drought-recovery period, is consistent with previous research showing evidence for differential sensitivity of transcript responses to temperature and water availability in *A. gerardii*^9^ and ecophysiological responses for the same individuals of *A. gerardii* and *S. nutans* sampled concurrently^37^ and in other experiments^30^.

We also observed different patterns of up- and down-regulation, and different functional groups of transcripts were found changed in abundance in *A. gerardii* vs. *S. nutans*. Moreover, rarely was there overlap between the two species in the identity of transcripts that responded significantly (Fig. 3; Appendix S3, S4). Differential regulation of multiple functional groups was observed between these two species in response to the combination of higher T_leaf_ and lower LWP (most transcripts in *A. gerardii* had increased transcript abundance levels vs. reduced abundance levels in *S. nutans*), LWP (lower LWP associated with increased transcripts abundance only in *S. nutans*), T_leaf_ (higher T_leaf_ associated with increased transcripts abundance only in *S. nutans*) and the drought-recovery period (increased transcript abundance associated with increased soil moisture in *A. gerardii*). These large differences were observed primarily in transcripts involved in different categories of processes, such as cell, glycolysis, protein, redox, RNA, signaling, stress, transport. In contrast, contrary to our expectations, we observed few responses of transcripts related to photosynthesis, and when there were significant responses, these were mostly related to variation in T_leaf_ rather than LWP. Other studies have shown that photosynthesis-related genes can be over-represented in response to drought recovery in several plant systems^40^. Previous research with *A. gerardii* found down-regulation of a number of photosynthesis-related genes to season-long drought^10^. The lack of response of photosynthesis-related genes in our study may be related to the relatively subtle nature of the experimental treatments and the lack of extreme water or temperature stress during the study period.

The differential patterns of regulation, particularly with respect to those genes involved in stress responses, suggest that the two species may use different strategies when coping with and/or responding to the increasing temperatures and water stress and their combined effects in the field. Even though no differential regulation of transcripts involved in stress recognition, such as genes in ABA synthesis pathway or transcription factor cascades, were found for the two species, we detected differential regulation of genes involved in stress avoidance and those associated with damage prevention and repair. For example, transcripts encoding sucrose synthase, which is related to osmotic adjustment for stress avoidance^41^, were only found in increased abundance in connection to LWP in *A. gerardii*. Antioxidant enzymes, such as glutathione reductase and superoxide dismutase, and HSPs play an important role in stress response, with antioxidant enzymes generally involved in the prevention of damage^42^ and HSP involved in damage repair by assisting with protein folding and stabilization^43^. In our study, transcripts encoding antioxidant enzymes and HSPs showed a consistent increase in response to increasing T_leaf_ in *S. nutans*, but not *A. gerardii*. The increased levels of these two groups of transcripts in response to increasing T_leaf_ in *S. nutans* suggests that this grass may be experiencing damage associated with temperature stress, whereas *A. gerardii* may not be experiencing stress or may responding to stress in different ways, such as through acclimation^44^ and osmotic adjustment.

Transcript abundance levels of aquaporins also changed significantly in *S. nutans* but not in *A. gerardii* in response to the variation of T_leaf_, where generally there was increased abundance with the increase of T_leaf_. Plant aquaporins, which serve as membrane water channels to increase water permeability, is a group of proteins generally thought to be involved in stress avoidance^45^. Aquaporins, mostly studied in Arabidopsis, have been associated with diverse abiotic stress responses, including water, nutrient, cold stress and other biotic stresses. However, the regulation of aquaporins in stress responses is inconsistent in the literature. ABA and water stress were found to increase aquaporin transcript abundance in some studies^46^,^47^, but decrease transcript levels in others^47^,^48^. As far as we know, studies of the regulation of aquaporins and their subgroups and isoforms in response to warming in plants are lacking. Regardless, the regulation of aquaporins in *S. nutans* but not in *A. gerardii* again suggests that one of the strategies for *S. nutans* to avoid temperature stress may be to increase expression level of aquaporins, while this was not the case for *A. gerardii*.

Overall, the patterns of transcript abundance levels associated with T_leaf_ and LWP, taken together with the ecophysiological performance of both species^30^,^37^, suggests that *S. nutans* is more sensitive and responsive to increasing temperatures with the increased transcripts abundance of HSPs, aquaporins, superoxide dismutase and ascorbate peroxidase. In contrast, *A. gerardii* appears less sensitive to the variation in temperature and water availability experienced in this experiment. A similar pattern in transcript abundance changes was observed for the short-term drought-recovery response for both species. In *S. nutans*, a large number of transcripts from multiple functional groups changed in abundance, with similar numbers of transcripts increased or decreased transcript abundance, while many fewer transcripts changed in abundance for *A. gerardii*. We cannot conclude which species is more responsive to short-term recovery from drought because it is not clear if both species responded at the same rate. This should be verified with more detailed time-course responses of both species to recovery from drought.

Plant stress response is a temporal process in which the stress is first recognized and then different strategies are adopted to either avoid the stress or respond to the stress by preventing and/or repairing the damage. Comparison of changes in levels of specific transcripts during season-long water stress and a short-term, mid-season drought-recovery period showed that, among the 1,639 transcripts changed in abundance during the drought-recovery period and the 101 transcripts that were related to the variation of LWP for *S. nutans*, only 11 were common between the two. Similarly, only 10% of the genes were in common when rapidly dehydrated (~6 h) barley roots were compared to gradually dehydrated (~7 d) barley roots^49^. This is partially due to different gene expression responses to acute versus chronic stress^50^,^51^,^52^. In our study, the stress gradually increased over time and encompassed a large portion of the growing season. This may have allowed for plant acclimation, which could explain the small number of transcripts regulated over the entire growing season when compared to the large number of transcripts regulated during a short-term drought-recovery period. For *S. nutans*, even though thousands of transcripts changed significantly in abundance with recovery from drought, many fewer responded during the season-long variation in water availability associated with the altered precipitation and warming treatments, and very few transcripts were common to the two types of stress.

In this study, we investigated the transcriptional profiles of the co-dominant tallgrass prairie C_4_ grass species, *A. gerardii* and *S. nutans*, exposed to variable rainfall regimes and increased temperatures under field conditions. Although our use of heterologous hybridization with maize cDNA microarrays (which was the most accessible technique available at the time of the experiment) has limitations resulting from the sequence divergence between the focal species and the model species^8^,^18^,^19^, we used stringent criteria to minimize these. First, during the quality control process, spots with signal to noise ratios of less than 10 were excluded from further analysis to decrease the inclusion of cross-hybridization artifacts^53^. As a result of these screening, approximately 70% of the transcripts with above-background detection remained for statistical analysis for both species (see Appendix S1), which suggests heterologous hybridization is feasible for analysis of transcript regulation for these two species without losing generality. Second, the microarray results of nine transcripts from each species were generally consistent with species-specific qRT-PCR results (see Appendix S10), confirming the expression changes observed with the heterologous hybridizations. Moreover, a number of significantly up- or down-regulated genes common to stress response or damage repair according to MapMan ontology were discovered in the two species responding to our imposed environmental stresses. The detection of these specific transcript changes provides confidence in the results from heterologous hybridizations. Finally, the cDNA sequences of the maize microarray SAM1.2 (18862 sequences) we used in this experiment were searched against a non-redundant transcripts data set of *A. gerardii* and *S. nutans* (unpublished data). With an E-value cutoff of <1E-10, ~75% and 73% of the probes had one or more hit to *A. gerardii* and *S. nutans* transcripts data set, respectively. This roughly corresponds to the percentage of the hybridized features to maize, with 75.14% and 78.60% for *A. gerardii* and *S. nutans*, respectively. The hybridization data suggests that there is likely conservation of stress response genes across these three species. RNA-seq techniques may provide further insight to gene expression changes, including transcripts conserved across species, as well as species specific transcripts. Although species-specific transcripts may provide insight into unique adaptations in these native grasses, it less likely that the annotated function of such transcripts is known.

In summary, the two focal species in this study, *A. gerardii* and *S. nutans*, co-dominate tallgrass prairie plant communities. They are close phylogenetic relatives^32^,^33^ who are generally considered ecologically and functionally equivalent and similarly adapted to high heat and drought conditions. However, our study found that these grasses differ substantially in their transcriptional responses to changing environmental conditions; these differences included the number, pattern and the identity of functional groups of transcripts with significant changes in abundance in response to variation in leaf temperature and leaf water potential. In particular, transcripts related to stress response, as well as other important function groups (cell function, signaling, transport), were consistently increased in abundance in *S. nutans* leaf tissue in response to both decreasing water availability (e.g., LWP in the season-long stress) and increasing temperatures. Little overlap was shown between the two species in the types of genes being regulated in correlation with variation in leaf-level temperature and water content. The different sensitivities of *A. gerardii* and *S. nutans* to individual stressors, which is in line with differential sensitivities observed at the ecophysiological, whole plant and community levels (e.g.,^30^,^34^,^35^,^37^), imply that these two closely related species may utilize different strategies for coping with and/or responding to environmental stressors. The differential transcriptomic responses of these co-dominant species could influence individual performance (physiological and growth) and ultimately have consequences for the structure and function of the tallgrass prairie ecosystem, as the two species contribute disproportionately to ecosystem function and structure^27^,^54^. Because this study was conducted in the field, it provides valuable insight to the mechanisms these co-dominant species use in a natural setting. We suspect that increased sensitivity by *S. nutans* may contribute to maladaptive plasticity^55^, which could lead to greater dominance in the community by *A. gerardii*. It is unknown whether selection for drought-resistant genotypes had already occurred in this field experiment when individuals were sampled, though is evidence for selection of genotypes over the long term^56^. Regardless of whether selection occurred, we posit that both plasticity within genotypes and different responses by selected genotypes will be important for understanding these species’ responses to gradual climate change.

Given that the precipitation and warming treatments represent relatively subtle changes in temperature and water availability and that it is often difficult to distinguish effects of temperature vs. water stress, the next steps to understanding how these two grass species differ in their sensitivity to climate change are to impose more severe temperature and water stress at a range of treatment levels^57^ and integrate proteomic and metabolomic analyses, together with the application of next generation sequencing techniques (e.g., RNAseq). This will allow us to improve our mechanistic understanding of the effects of increasing temperature vs. water stress on responses of the co-dominant grasses, and to determine whether there are thresholds in their responses.

## Methods

### Site description

The study was conducted at the Rainfall Manipulation Plots (RaMPs), a long-term climate change field experiment established maintained since 1998 and located at the Konza Prairie Biological Station in northeastern Kansas^58^. The RaMPs experiment consists of 12 location-fixed shelters (14 × 9 m) located over 6 × 6 m native tallgrass prairie plots, which are divided into four 2 × 2 m plots, two of which have infrared heating lamps installed. The RaMPs shelters allow for the manipulation of growing season precipitation in either an ambient (rainfall immediately applied) or altered pattern (50% increase in time between ambient rainfall events). On average, the altered precipitation treatment reduces volumetric soil moisture by ~15%^58^. Warming treatments (unwarmed, warmed) are nested within the precipitation treatments and are applied year-round to achieve on average a + 2 °C increase in ambient air temperature^58^. Thus, both treatments result in moderate changes in water availability and temperature in the experimental plots.

### Focal species and field sampling

We focused on the two co-dominant tallgrass prairie species, *A. gerardii* and *S. nutans*, both of which are C_4_ grasses that reproduce primarily vegetatively via buds on belowground rhizomes^59^, forming dense intermixed stands of tillers (individuals). As clones grow and spread, they sever their root and rhizome connections^60^, and as a consequence individual tillers of sufficient distance apart are independent of each other. Because the RaMPs experiment is located in an annually burned area, tillers typically emerge in early-mid April (after burning) and grow vegetatively until mid-July/August when some (but not all) individuals initiate flowering^61^. Individuals typically increase in size vegetatively from mid-Apr to mid- to late-June (unpubl. data). We did not control for plant genotype due to an interest in average species-level response, although this approach can create additional noise in transcriptional response.

We sampled a single tiller of each species within the control (unwarmed) and a randomly selected warmed subplot in paired (based on adjacency) ambient and altered RaMPs. Sampling occurred five times during the growing season in 2006: June 1, June 7, July 18, July 24 and August 22. June 1 was chosen to capture early growing season conditions when water and temperature stress are typically minimal. However, due to less than ideal conditions (lower PAR (photosynthetically active radiation)), June 7 also was chosen to capture early growing season conditions with higher light availability. The two dates in July were chosen specifically to capture a drought and recovery period; the first (July 18) when soil moisture was low and the second (July 24) five days after rainfall events were applied to both the altered and ambient precipitation treatments. Finally, August 22 was chosen to capture late growing season conditions. For each sampling date, morphologically similar vegetative (non-flowering) tillers (3–5 fully expanded leaves) of each species were identified in each of the subplots. At each sampling date, one individual of each species was selected from each replicate plot subjected to the precipitation (n = 2, ambient, altered) and warming (n = 2, control, warmed) treatments, for n = 2 species × 4 treatment combinations × 3 replicates per treatment combination = 24 individuals per five sample periods, n = 120 total. Because timing discrepancies may affect gene expression^12^, leaf tissue was collected from individuals located in paired ambient and altered RaMPs within five minutes of each other. The first or second fully expanded leaf was randomly selected for genomic analysis from each individual to ensure leaves had finished developing but were not old enough to be senescing or have reduced cellular activity. The entire leaf was clipped and immediately flash-frozen and stored in liquid nitrogen until brought to the laboratory. Immediately after collection of the first leaf, we measured leaf surface temperature (T_leaf_) on the remaining leaf, as part of intensive physiological measurements reported elsewhere (see Nippert *et al*.^37^). The whole leaf was then collected for determination of leaf water potential (LWP) using a Scholander-type pressure chamber (PMS Instruments, Inc., Corvallis, OR, USA). LWP reflects the xylem pressure required to continue the flow of water from roots to leaves, where more negative water potentials generally represent plant water stress. We focused on T_leaf_ as an indicator of the warming treatment and temperature stress, as it is highly correlated with canopy temperature^37^ and was significantly elevated in response to the warming treatment (Fig. 1A,B). Focusing on LWP as an indicator of the precipitation treatment and water stress was due to its highly correlation with soil water content at 25, 50 and 75 cm (r = 0.64, 0.61 and 0.57, respectively, with p-values < 0.0001), which was measured on a bi-weekly basis with neutron probes located in the warmed and unwarmed subplots within each RaMP. In addition, LWP was significantly reduced with the altered precipitation treatment (Fig. 1C,D). The ranges of both T_leaf_ and LWP captured included levels considered stressful and potentially inhibitory for ecophysiology for the two species^30^.

Mixed-model ANOVAs were implemented in SAS (SAS/STAT Software version 9.1.3, SAS Institute, Cary, NC, USA) to assess the effects of sampling date (five dates during the growing seaon) and the precipitation (ambient, altered) and warming (control, warmed) treatments on T_leaf_ and LWP between the two grass species (Fig. 1). Precipitation was treated as a fixed effect, with warming treated as a random effect nested within precipitation. Sampling date was treated as a random effect.

### RNA preparation and microarray hybridization

Leaf tissue samples were stored in an −80 °C freezer for less than one year prior to RNA extraction. Total RNA was extracted from each of the 240 leaf samples (two species) using TRIzol reagent (Invitrogen, Carlsbad, CA, USA)^62^, and purified with the RNeasy kit (Qiagen, Valencia, CA, USA). RNA quantity was measured by a NanoDrop spectrophotometer (Nanodrop products, Thermo Scientific, Wilmington, DE, USA). The verification of RNA quality, preparation of cDNA, and subsequent steps leading to hybridization and array scanning were performed by the Keck Biotechnology Resource Facility at Yale University (http://keck.med.yale.edu/). We used maize spotted cDNA arrays (SAM 1.2, GEO platform GPL4521) produced by the Center for Plant Genomics at Iowa State University for hybridization. The arrays included 15,680 maize cDNA clones (14,118 informative) isolated from maize ear tissue. The sample pairing for microarray hybridization followed Design C of Milliken *et al*.^63^, in which pairs were assigned within a level (ambient vs. altered precipitation) of the whole plot treatment and across levels (unwarmed vs. warmed) of the subplot treatment.

### Quality control of heterologous hybridizations

In total, there were hybridizations for 47 leaf samples of *A. gerardii* and 49 leaf samples of *S. nutans*. Some samples had to be discarded due to low RNA quality. Array image data were collected using GenePix software (Version 6, Molecular Devices, Inc., Sunnyvale, CA, USA) and normalized using the ratio of medians. Prior to the normalization, features with obvious abnormality and saturated signal were flagged as uninformative and excluded from statistical analysis. Because the signal intensity of some spots was low as a consequence of the heterologous hybridization, stringent criteria concerning signal to noise ratios were applied during quality control of the images. Features with signal to noise ratios (for both channels, 635 nm and 532 nm) less than 10 were excluded from further normalization. After quality control (see Appendix S1 in Supporting Information), there were 10,607 features in total for further analysis for *A. gerardii*, while 11,095 features were included for *S. nutans*, among which 10,329 were common for both species. Considering the different hybridization efficiencies (Appendix S1), we focused the following statistical analysis on only the common features of *A. gerardii* and *S. nutans*, discarding 278 unique transcripts from *A. gerardii* and 766 from *S. nutans*.

### Statistical analysis of microarray data

The microarray data were analyzed for each species separately using high-throughput regression^9^ to assess how gene expression varied among individual plants as a consequence of altered precipitation and temperatures over the entire growing season. Specifically, multiple regression analysis was used to capture the linear relationship between gene expression levels, as the dependent variable, and T_leaf_, LWP, together with their interaction, and date (representing the five sampling dates) as the independent variables. The inclusion of date was used to distinguish responses of the transcriptome that are due to changes in temperature or water availability from those due to sampling time. Prior to regression analysis, the microarray data were normalized following Travers *et al*.^9^.

Regression analysis was conducted for each species separately with the residuals from the normalization analysis. Data was subset so only the common hybridized features (10,329) represented by all five sampling dates were included in the regression analyses: 10,309 (99.8% of the common hybridized features) features in total for both species. Statistical significance of either positive (increased transcript abundance associated with higher T_leaf_ or decreased transcript abundance associated with lower LWP) or negative (decreased transcript abundance associated with higher T_leaf_ or increased transcript abundance associated with lower LWP) slopes was tested against the null hypothesis that the slope was zero. Significant differences in the number of transcripts with abundance change in response to T_leaf_, LWP, their interaction, and sampling date between the two species were tested by Pearson χ^2^ test.

To assess genes responding to drought vs. recovery, we subsetted the data focusing on the two sampling dates in July. Specifically, mixed-model ANOVA was used to assess alteration of transcript levels under drought conditions (July 18) vs. drought recovery (July 24). There were 8,662 and 10,776 (82% and 97% of hybridized features) transcripts analyzed for *A. gerardii* and *S. nutans* respectively, among which 7869 features were common for both species. Similarly, we focused on these common features in the drought-recovery analysis. The normalized expression data (residuals from the normalization analyses above) were used as the dependent variable, with precipitation and warming treatments, sampling date and their interactions as independent variables (fixed effects), and RaMP as random effect.

The normalization analyses, high-throughput regression analyses, mix-model ANOVA for drought recovery evaluation, and Pearson χ^2^ test were implemented in SAS (version 9.1.3). For regression analyses and mix-model ANOVA, statistical significance was further evaluated using the q-statistic to control the experiment-wise false discovery rate (FDR)^64^. A q-value of less than 0.05 was chosen to identify significant changes in transcript levels. The transcripts with significant abundance changes were grouped into major functional groups using an extension of MapMan gene ontology^65^ to maize using protein sequence identity to Arabidopsis.

### Quantitative Real-time Polymerase Chain Reaction (qRT-PCR)

We used qRT-PCR to validate the microarray results for the same RNA samples used in the heterologous hybridization (see Appendix S2). Because leaf tissue of *A. gerardii* and *S. nutans* was often limited and a relatively large amount of RNA was utilized for the microarray hybridizations, RNA samples were not available for qRT-PCR analysis for all individuals or for all sampling dates. Therefore, we focused on only those sampling dates in which there were adequate samples available for a subset of individuals (June 7 and July 18 for *A. gerardii*, June 7 and August 22 for *S. nutans*). Nine annotated transcripts with significant response to variation in LWP or T_leaf_ according to microarray analysis and which have been shown to be involved in stress response (e.g., superoxide dismutase, HSP 90) were selected and cloned (Appendix S2). Expression levels of these transcripts were analyzed for each species with qRT-PCR, with β-tubulin and Elongation factor (EF) 1α as the internal standard for *A. gerardii* and *S. nutans*, respectively. The range of the primer amplification efficiency was between 90% and 98%, and correlation coefficiency was between 99.1% and 100.0% as determined by serial dilution of cDNA templates.

## Additional Information

**How to cite this article**: Smith, M. D. *et al*. Gene expression patterns of two dominant tallgrass prairie species differ in response to warming and altered precipitation. *Sci. Rep*. **6**, 25522; doi: 10.1038/srep25522 (2016).

## Supplementary Material

Supplementary Information

## Figures and Tables

**Table 1 t1:**
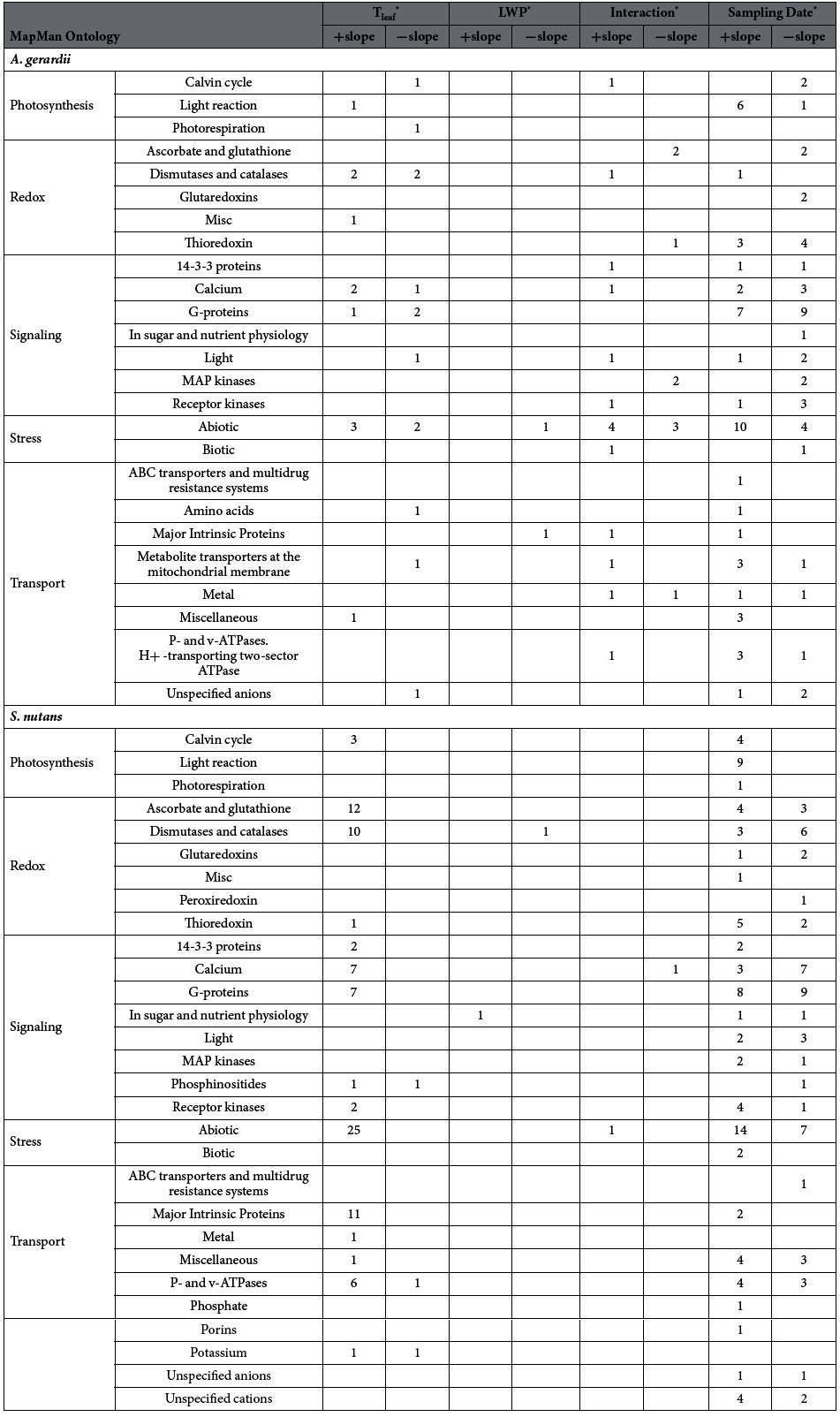
Transcript abundance changes in leaf tissue of *Andropogon gerardii* and *Sorghastrum nutans* in response to variation in leaf temperature (T_leaf_), leaf water potential (LWP), their interaction, and sampling date (as indicated by significant positive or negative slopes, q-value < 0.05).

Transcripts were categorized into major functional groups and further subdivided in specific functions according to MapMan gene ontology for Maize. Only the groups of photosynthesis, redox, signaling, stress, and transport were shown here; for the full list see Appendix S3 & S4.

^*^Positive slopes with T_leaf_ indicate increased transcript levels with higher leaf temperatures; positive slopes with LWP indicate decreased transcript levels with increased water stress (lower LWP); positive slopes with date indicate increased transcript levels at later harvest dates.
