# Supplementary material for: Gene expression patterns of two dominant tallgrass prairie species differ in response to warming and altered precipitation
Source: Sci Rep. 2016 May 13;6:25522. doi: 10.1038/srep25522 (PMC4865957; doi:10.1038/srep25522)
Supplement: Supplementary Information [file srep25522-s1.pdf]

Gene expression patterns of two dominant tallgrass prairie species differ in response to warming and altered precipitation

Melinda D. Smith[1], Ava M. Hoffman[1], and Meghan L. Avolio[2]

[1]Department of Biology and Graduate Degree Program in Ecology, Colorado State University, Fort Collins, CO 80523, USA.

[2] National Socio-Environmental Synthesis Center, Annapolis, MD, 21401, USA.

Appendix S1. Quality control of the heterlogous hybridization of maize cDNA arrays with cDNAs from *Andropogon gerardii* and *Sorghastrum nutans*.

Species	Number of bad features* (%)	Number of non-hybridized features† (%)	Number of hybridized features (%)
A. gerardii	3710 (23.67)	1362 (8.69)	10608 (67.65)(75.14% of informative features)
S. nutans	3135 (19.99)	1449 (9.24)	11096 (70.76)(78.60% of informative features)

* denotes features with poor hybridization quality;

† denotes features automatically flagged as bad features by Genepix software.

Appendix S2. Quantitative Real-time Polymerase Chain Reaction (qRT-PCR) and primers.

Orthologs of the following maize cDNAs were cloned and sequenced: CD651724, CB833735, DV551311, CD001612, CB380843, DV621372, DB622645, CD568792, CB833708, CD001262. Primers of the nine genes differentially expressed for each species were designed using PrimerQuest with the parameter set of "real-time PCR" (http://www.idtdna.com/Scitools/Applications/Primerquest/). DNase-treated total RNA (500 ng) was used for the synthesis of first strand cDNA for RT-PCR using OmniScript RT-PCR kit (Cat. 205110, Qiagen, Valencia, CA, USA) according to the supplier's manual. The synthesized cDNA was diluted 5 times with 80 µl ddH_2O. Twenty microliter quantitative real time PCRs with one microliter cDNAs were run on ABI 7500 Fast PCR system (Applied Biosystems, Foster City, CA) using Power SYBR green PCR master mix (ABI 4367656, Applied Biosystems, Foster City, CA). The cycling condition was 95 ºC 10 min, 40 times of 95 ºC for 15 s, 60 ºC 1 min. Three replicates were performed for each sample in addition to one non-template-control. The Ct values were quantified and analyzed according to Livak and Schmittgen (2001) by averaging the three independent calculated normalized expression values that were duplicated on the plate for each treatment.

Primers used in quantitative real-time PCR for validation of heterologous hybridizations of maize cDNA arrays with cDNAs from *Andropogon gerardii* and *Sorghastrum nutans*

Orthologous cDNA clones	Annotation	Species	Forward primer (5'→3')	Reverse primer (5'→3')
CD651724	Glyoxalase I	Both	TGGGCTATGCTGATGAGGACAA	GCATATGCATTGCCCTTGGTGT
DV551311	22 kDa drought-inducible protein	Both	TGGGCCAAACTCAATCATTGGC	TGGGCCAAACTCAATCATTGGC
CB380843	Dehydroascorbate reductase	*A.gerardii*	AGAAGCGCCTTCTCTGAACCATCA	AGTTCCCAACTCCACTCTGGTCA
CB380843	Dehydroascorbate reductase	*S. nutans*	TAAGCCATCCGAGGAGCATGTGAT	ATACACACCACTGGCAACGACTGA
DV621372	Superoxide dismutase [Cu-Zn] 4AP	Both	AGGGTGGACATGAGCTTAGCAAGA	AACAGCAATTCCTGATGTGCTCGC

DV622645	Dehydroascorbate reductase	*A gerardii*	ATCTGATCAGTCGTTGCCAGTGGT	ACCGGCAATAGAAGGTAAGACGCA
DV622645	Dehydroascorbate reductase	*S. nutans*	TTTGTGCTTTGGTGCTACCTGGTG	CCAACTGAATCATGCGTCTAGGCT
CD568792	Heat shock protein 90	*A.gerardii*	TCTTCAAACTCTTCCACCTCGGCA	TCTGGGCGCCTACAAGGATAAGAA
CD568792	Heat shock protein 90	*S. nutans*	AGAGCAAGAAGGCAGTGGAGAACT	TCGAACTCCTTGAGCTGACCAACA
CB833708	Tonoplast intrinsic protein	*A. gerardii*	AGAAGCGCCTTCTCTGAACCATCA	AGTTCCCAACTCCATCTCTGGTCA
CB833708	Tonoplast intrinsic protein	*S. nutans*	ATCTCTCTCCCTCGCCGTTTAGTT	TCCAAGAAAGCAAACGATGCGAGC
CD001262	Heat shock protein 90	*A. gerardii*	TGCTTTCTTGGTCTTGGGCTTAGG	ATTGGCACAACACATGGGAGCAG
CD001262	Heat shock protein 90	*S. nutans*	AGGAACCGCCTTGCTAGCTTCTTA	AGAAGATGTCTTTCTGCCCTGGCT
CD001612	Superoxide dismutase [Cu-Zn] 4AP	*S. nutans*	CTGTTGTTGTTCACGCTGATCCTG	TTTCAGCCCTGGAGTCCAATGA
CB833735	14-3-3-like protein GF14-6	*A. gerardii*	AGCTTGCTCCAACTCACCCTAT	AAGCCTGCTTTGCAAGACTGC
β-tubulin		*A. gerardii*	AGATGTTCCGCCGTGTCAGC	CCTCGCCCGTGTACCAATGC
EF-1α		*S. nutans*	GCCACCACCTCCCACATCG	CATCTTCACCATACCAGCATCACC

Appendix S3. Transcript abundance changes in leaf tissue of *Andropogon gerardii* in response to variation in leaf temperature (T_{leaf}), leaf water potential (LWP), their interaction, and sampling date (as indicated by significant positive or negative slopes, q-value < 0.05). Transcripts are categorized into major functional groups and further subdivided in specific functions according to MapMan gene ontology for Maize (S. Yuan & M.D. Smith, unpublished manuscript).

**MapMan Ontology**		**Tleaf***		**LWP***		**Interaction***		**Sampling date***	
		**+ slope**	**- slope**	**+ slope**	**- slope**	**+ slope**	**- slope**	**+ slope**	**- slope**
Amino acid metabolism	Degradation	3				1		3	7
	Synthesis		3					6	2
C1-metabolism	S-(hydroxymethyl)glutathione dehydrogenase & S-(hydroxymethyl)glutathione synthase							1	
Cell wall	Cell wall proteins							1	
	Cellulose synthesis						1	1	1
	cell wall.degradation.mannan-xylose-arabinose-fucose								1
	Degradation						1		
	Modification								2
	Pectin*esterases							2	2
	Precursor synthesis								1
	Vesicle transport	1					1	1	4
	Cycle					1		2	7
	Division						1	1	4
	Organisation	3				3	2	6	17
Co-factor and vitamine metabolism	Biotin							1	
	Folate & vitamine K.folate							1	
Development	Storage proteins							1	1
	Unspecified	1	3					5	4
DNA	DNA.repair							1	

	Repair				1				
	Synthesis/chromatin structure	4	4			15	1	26	24
	Unspecified							1	1
Fermentation	Aldehyde dehydrogenase							1	3
Glycolysis	Aldolase					1			
	Enolase						1		
	G6PIsomerase					1			1
	Glyceraldehyde 3-phosphate dehydrogenase					1	1		1
	PEPCK					1			
	Pyrophosphate-fructose-6-P phosphotransferase		1				1		
Hormone metabolism	Abscisic acid								1
	Auxin						1		3
	Brassinosteroid							1	1
	Ethylene		1						1
	Gibberelin								3
Lipid metabolism	Exotics' (steroids, squalene etc)			1					2
	FA synthesis and FA elongation							2	3
	Lipid degradation							2	1
	Phospholipid synthesis		1			1		1	1
Major CHO metabolism	Degradation			1				1	3
	Synthesis							1	1
Metal handling	Binding, chelation and storage						1	1	3
Minor CHO metabolism	Callose	1							
	Myo-inositol.poly-phosphatases								1
	Others							2	2

	Trehalose								1
Misc	Other Ferredoxins and Rieske domain							1	
	Acid and other phosphatases							1	1
	Calcineurin-like phosphoesterase family protein							1	
	Cytochrome P450					1		1	
	GDSL-motif lipase							1	1
	Gluco-, galacto- and mannosidases						1		
	Glutathione S transferases	1	1					3	3
	Myrosinases-lectin-jacalin						1		1
	Nitrilases, *nitrile lyases, berberine bridge enzymes, reticuline oxidases, troponine reductases								1
	Protease inhibitor/seed storage/lipid transfer protein (LTP) family protein						1	1	1
	Short chain dehydrogenase/reductase (SDR)						1	1	
	UDP glucosyl and glucoronyl transferases		2					1	
Mitochondrial electron transport / ATP synthesis	Cytochrome c							1	
	Cytochrome c oxidase					1		1	1
	Cytochrome c reductase							1	1
	F1-ATPase		1						2
	NADH-DH.localisation not clear							3	3
N-metabolism	Ammonia metabolism								1
Nucleotide	Degradation							1	

metabolism	Deoxynucleotide metabolism								1
	Phosphotransfer and pyrophosphatases	2				4	1		2
	Salvage	1							1
	Synthesis								1
OPP	Oxidative PP							1	2
Protein	Assembly and cofactor ligation							1	1
	Amino acid activation		2			1	1	2	3
	Degradation	4	8	1		7	3	29	34
	Folding							2	
	Glycosylation		1					1	
	Postranslational modification	1	1			3	2	11	15
	Synthesis	31	13		1	29	6	95	58
	Targeting	1	3			4	1	5	4
Photosynthesis	Calvin cycle		1			1			2
	Lightreaction	1						6	1
	Photorespiration		1						
Redox	Ascorbate and glutathione						2		2
	Dismutases and catalases	2	2			1		1	
	Glutaredoxins								1
	Misc	1							
	Thioredoxin						1	3	4
RNA	Processing	2	2			5	1	4	7
	Regulation of transcription	5	8			10	6	30	34
	RNA binding	1						1	14
	Transcription					1	1	1	3
Secondary	Isoprenoids								1

metabolism	N misc								1
	Phenylpropanoids							1	2
	Simple phenols							1	
Signaling	14-3-3 proteins					1		1	1
	Calcium	2	1			1		2	3
	G-proteins	1	2					7	9
	In sugar and nutrient physiology								1
	Light		1			1		1	2
	MAP kinases						2		2
	Receptor kinases					1		1	3
Stress	Abiotic	3	2		1	4	3	10	4
	Biotic					1			1
TCA / org	Transformation		2			1	1	3	1
Tetrapyrrole synthesis	Chlorophyll b synthase								1
	Heme oxygenase							1	
	Magnesium chelatase							1	
	Porphobilinogen deaminase								1
Transport	ABC transporters and multidrug resistance systems							1	
	Amino acids		1					1	
	Major Intrinsic Proteins				1	1		1	
	Metabolite transporters at the mitochondrial membrane		1			1		3	1
	Metal					1	1	1	1
	Misc	1						3	
	P- and v-ATPases.H+-transporting two-sector ATPase					1		3	1

	Unspecified anions		1					1	2
Transporter	Sugars						1		1
Not assigned	No ontology	8	16			7	3	25	34
	Unknown		9			14	8	36	62
No homology in Arabidopsis		18		1	1	27	25	115	161
Total		99	126	4	5	159	85	501	613

* Positive slopes with T_{leaf} indicate increased transcript levels with higher leaf temperatures; positive slopes with LWP indicate decreased transcript levels with increased water stress (lower LWP); positive slopes with date indicate increased transcript levels at later harvest dates.

Appendix S4. Transcript abundance changes in leaf tissue of *Sorghastrum nutans* in response to to variation in T_{leaf}, LWP, their interaction and sampling date (as indicated by significant positive or negative slopes, q-value < 0.05). Transcripts are categorized into major functional groups and further subdivided in specific functions according to MapMan gene ontology for Maize (S. Yuan & M.D. Smith, unpublished manuscript).

**MapMan ontology**		**Tleaf***		**LWP***		**Interaction***		**Samping date***	
		**+ slope**	**- slope**	**+ slope**	**- slope**	**+ slope**	**- slope**	**+ slope**	**- slope**
Amino acid metabolism	Degradation	1						7	8
	Synthesis	5	1					15	8
C1-metabolism	Formate dehydrogenase							1	
	Methylenetetrahydrofolate dehydrogenase & Methenyltetrahydrofolate cyclohydrolase								1
	S-(hydroxymethyl)glutathione dehydrogenase & S-(hydroxymethyl)glutathione synthase				1				
Cell wall	Cell wall proteins	1							1
	Cellulose synthesis	5						2	1
	Degradation						1	2	
	Modification								2
	Precursor synthesis	1						1	2
	Vesicle transport	1	1				1	7	1
	Cycle	16		1				6	2
	Division		1					6	3
	Organisation	7	3	1				19	22

Co-factor and vitamine metabolism	Biotin							1	
Development	Late embryogenesis abundant								1
	Multitarget							1	
	Storage proteins	1	1					1	1
	Unspecified	6		1				4	4
DNA	Repair							3	1
	Synthesis/chromatin structure	12	8	2		1	4	39	28
	Unspecified	1						3	3
Fermentation	Aldehyde dehydrogenase	1						3	
Gluconeogenese/ glyoxylate cycle	Citrate synthase	1							
Glycolysis	Enolase							1	
	Glyceraldehyde 3-phosphate dehydrogenase				1			3	2
	PEPCase							3	1
	PEPCK	1							
	PGM							1	1
	Phosphoglycerate kinase							2	
	Pyrophosphate-fructose-6-P phosphotransferase							2	1
	TPI								2
Hormone metabolism	Abscisic acid	1					1	1	1
	Auxin	1		1			1	3	1

	Brassinosteroid							5	2
	Cytokinin								1
	Ethylene	1						1	1
	Jasmonate							1	
Lipid metabolism	Exotics' (steroids, squalene etc).cycloartenol synthase							2	
	FA desaturation							1	
	FA synthesis and FA elongation							3	7
	Glyceral metabolism								1
	Glycolipid synthesis								1
	Lipid degradation			1				5	2
	Lipid transfer proteins etc							2	1
	Phospholipid synthesis	1						1	
	TAG synthesis							1	
Major CHO metabolism	Degradation							2	1
	Synthesis	2						2	2
Metal handling	Binding, chelation and storage							1	5
	Regulation	3							1
Minor CHO metabolism	Callose								1
	Others	1						3	2
	Trehalose								2
Misc	Acid and other phosphatases							1	
	Cytochrome P450							1	1
	Dynamin							1	

	GCN5-related N-acetyltransferase								1
	GDSL-motif lipase							3	
	Gluco-, galacto- and mannosidases							2	
	Glutathione S transferases	5	2					5	1
	Invertase/pectin methylesterase inhibitor family protein							1	
	Myrosinases-lectin-jacalin						1	2	2
	Nitrilases, *nitrile lyases, berberine bridge enzymes, reticuline oxidases, troponine reductases							1	
	O- methyl transferases								1
	Other Ferredoxins and Rieske domain	1							
	Oxidases - copper, flavone etc.		1						
	Protease inhibitor/seed storage/lipid transfer protein (LTP) family protein							5	2
	Short chain dehydrogenase/reductase (SDR)							1	
	UDP glucosyl and glucoronyl transferases							4	
Mitochondrial electron transport / ATP synthesis	Cytochrome c	1	1						1
	Cytochrome c oxidase							1	
	F1-ATPase	1	1					3	2
	NADH-DH	1						6	3

N-metabolism	Ammonia metabolism							2	
	Misc								1
Nucleotide metabolism								1	
	Degradation						1		1
	Deoxynucleotide metabolism							3	
	Phosphotransfer and pyrophosphatases	6						4	4
	Salvage							1	
	Synthesis							1	1
OPP	Oxidative PP	1							1
Polyamine metabolism	Degradation							1	
	Synthesis		1					4	1
Protein	Assembly and cofactor ligation	1						1	1
	Amino acid activation	1						6	2
	Degradation	21	3	7		1	1	46	37
	Folding	3						1	3
	Glycosylation	1							
	Postranslational modification	8					1	20	11
	Synthesis	111	5	4	4		2	92	144
	Targeting	4	1			1		10	10
Photosynthesis	Calvin cycle	3						4	
	Lightreaction							9	
	Photorespiration							1	
Redox	Ascorbate and glutathione	12						4	3
	Dismutases and catalases	10			1			3	6

	Glutaredoxins							1	2
	Misc							1	
	Peroxiredoxin								1
	Thioredoxin	1						5	2
RNA	Processing							10	17
	Regulation of transcription	3	22	5			5	45	52
	RNA binding	1	1	1				5	18
	Transcription	1						2	
Secondary metabolism	Flavonoids							1	
	Isoprenoids	1						1	1
	Phenylpropanoids							5	1
	Wax							1	
Signaling	14-3-3 proteins	2						2	
	Calcium	7					1	3	7
	G-proteins	7						8	9
	In sugar and nutrient physiology			1				1	1
	Light							2	3
	MAP kinases							2	1
	Phosphinositides	1	1						1
	Receptor kinases	2						4	1
Stress	Abiotic	25				1		14	7
	Biotic							2	
TCA / org	Transformation	2		1				6	3
Tetrapyrrole synthesis	Chlorophyll b synthase							3	2

Transport	ABC transporters and multidrug resistance systems								1
	Major Intrinsic Proteins	11						2	
	Metal	1							
	Misc	1						4	3
	P- and v-ATPases	6	1					4	3
	Phosphate							1	
	Porins							1	
	Potassium	1	1						
	Unspecified anions							1	1
	Unspecified cations							4	2
Transporter	Sugars							3	
Not assigned	No ontology	14	4	4			5	72	61
	Unknown	5	9	6		1		72	56
No homology in Arabidopsis		29	34	14	1		13	289	112
Total		383	103	51	8	5	38	995	736

*Positive slopes with T_{leaf} indicate increased transcript levels with higher leaf temperatures; positive slopes with LWP indicate decreased transcript levels with increased water stress (lower LWP); positive slopes with date indicate increased transcript levels at later harvest dates.

Appendix S5. Transcripts with significant abundance change in *Andropogon gerardii* and *Sorghastrum nutans* in response to variation in leaf temperature (T_{leaf}; q-value<0.05). Light grey shaded SPOTID's were transcripts changed abundance in both species.

**Gene Ontology**		**SPOTID**	**Gene Index**	**Gene Annotation**	**Slope Estimate**	
					**A. gerardii**	**S. nutans**
Amino acid metabolism	Degradation	10523	TC323304	UP|Q9XHH0_MAIZE (Q9XHH0) Acetoacetyl CoA thiolase, 92%	0.20	
		8966	TC367225	UP|Q84VE1_ORYSA (Q84VE1) Adenosylhomocysteinase-like protein		0.17
		6479	TC316141	UP|Q6XC06_MAIZE (Q6XC06) Glyoxalase I		0.20
		7653	TC355817	UP|Q5I7K2_WHEAT (Q5I7K2) Ribosomal protein S7	0.17	
		8059	TC371041	UP|CYPH_MAIZE (P21569) Peptidyl-prolyl cis-trans isomerase	0.23	
	Synthesis	1969	TC317448	UP|Q9LXU2_ARATH (Q9LXU2) Anthranilate phosphoribosyltransferase-like protein, 43%	-0.18	
		14299	TC370557	UP|TRPB2_MAIZE (P43284) Tryptophan synthase beta chain 2, chloroplast precursor (Orange pericarp 2) , 13%		0.20
		5417	TC327456	UP|Q8W0Q7_SORBI (Q8W0Q7) Methionine synthase protein	-0.17	
		3335	TC316943	UP|Q8W0Q7_SORBI (Q8W0Q7) Methionine synthase protein, 61%	-0.20	
		2850	TC365565	UP|METK_ORYSA (P46611) S-adenosylmethionine synthetase 1		0.17
		4825	TC338042	UP|METK_ORYSA (P46611) S-adenosylmethionine synthetase 1, 49%		0.17
		6175	TC365565	UP|METK_ORYSA (P46611) S-adenosylmethionine synthetase 1		0.17
		4607	TC331403	UP|Q8LJJ2_ORYSA (Q8LJJ2) Threonine synthase-like, 58%		0.19
		5210	TC318365	UP|Q5F4K8_PINPS (Q5F4K8) Aspartate aminotransferase, 77%		-0.28
		8136	TC368200	UP|Q9FPJ3_ARATH (Q9FPJ3) AT4g13930, 31%	-0.25	

Biodegradation of Xenobiotics	.lactoylglutathione lyase	6479	TC316141	UP|Q6XC06_MAIZE (Q6XC06) Glyoxalase I		0.20
		7653	TC356842	UP|Q6XC06_MAIZE (Q6XC06) Glyoxalase I, 22%	0.17	
C1-metabolism	Glycine hydroxymethyltransferase	8136	TC368200	UP|Q9FPJ3_ARATH (Q9FPJ3) AT4g13930, 31%	-0.25	
Cell wall	Cell wall proteins	5160	TC327388	UP|UPTG_MAIZE (P80607) Alpha-1, 4-glucan-protein synthase, 51%		0.19
	Cellulose synthesis	1459	TC332516	UP|Q9LLI9_MAIZE (Q9LLI9) Cellulose synthase-1, 29%		0.24
		4912	TC315858	UP|Q9LLI2_MAIZE (Q9LLI2) Cellulose synthase-8		0.21
		8843	TC315858	UP|Q9LLI2_MAIZE (Q9LLI2) Cellulose synthase-8		0.28
		12093	TC315858	UP|Q9LLI2_MAIZE (Q9LLI2) Cellulose synthase-8		0.22
		12236	TC347929	UP|Q9LLI7_MAIZE (Q9LLI7) Cellulose synthase-3, 24%		0.26
	Precursor synthesis	6022	TC356905	UP|Q2V4C2_ARATH (Q2V4C2) Protein At1g78570, 69%		0.20
Cell	Vesicle transport	302	TC366946	UP|Q9SV20_ARATH (Q9SV20) Beta-COP-like protein, 21%		0.28
		7608	TC364951	UP|Q8H5R6_ORYSA (Q8H5R6) Vesicle soluble NSF attachment protein receptor-like protein, 97%	0.22	
		8637	TC336352	UP|Q5N7M9_ORYSA (Q5N7M9) Anti-silencing function 1A-like, 30%		-0.18
		11398	TC323133	UP|Q94GI1_ORYSA (Q94GI1) Clathrin assembly protein AP19-like protein, 88%		0.19
	Cycle	2482	TC371041	UP|CYPH_MAIZE (P21569) Peptidyl-prolyl cis-trans isomerase		0.19
		3452	TC371041	UP|CYPH_MAIZE (P21569) Peptidyl-prolyl cis-trans isomerase		0.19
		3794	TC371041	UP|CYPH_MAIZE (P21569) Peptidyl-prolyl cis-trans isomerase		0.20
		4620	TC371041	UP|CYPH_MAIZE (P21569) Peptidyl-prolyl cis-trans isomerase		0.21
		5386	TC321726	UP|CYPH_MAIZE (P21569) Peptidyl-prolyl cis-trans isomerase		0.20

	6028	TC371041	UP|CYPH_MAIZE (P21569) Peptidyl-prolyl cis-trans isomerase		0.19
	7606	TC371041	UP|CYPH_MAIZE (P21569) Peptidyl-prolyl cis-trans isomerase		0.16
	7851	TC371041	UP|CYPH_MAIZE (P21569) Peptidyl-prolyl cis-trans isomerase		0.20
	8059	TC371041	UP|CYPH_MAIZE (P21569) Peptidyl-prolyl cis-trans isomerase	0.23	
	8457	-	-		0.15
	8816	TC371041	UP|CYPH_MAIZE (P21569) Peptidyl-prolyl cis-trans isomerase		0.21
	9777	TC371041	UP|CYPH_MAIZE (P21569) Peptidyl-prolyl cis-trans isomerase		0.17
	10475	TC371041	UP|CYPH_MAIZE (P21569) Peptidyl-prolyl cis-trans isomerase		0.20
	11247	TC371041	UP|CYPH_MAIZE (P21569) Peptidyl-prolyl cis-trans isomerase		0.16
	12071	TC371041	UP|CYPH_MAIZE (P21569) Peptidyl-prolyl cis-trans isomerase		0.18
	12404	TC371041	UP|CYPH_MAIZE (P21569) Peptidyl-prolyl cis-trans isomerase		0.15
	12521	TC371041	UP|CYPH_MAIZE (P21569) Peptidyl-prolyl cis-trans isomerase		0.20
Division	13617	TC318242	RF|NP_566644.1|18402300|NM_112849 nucleotide binding {Arabidopsis thaliana}, 96%		-0.20
Organisation	1262	TC337651	UP|Q6Z2W0_ORYSA (Q6Z2W0) Chromosome-associated kinesin-like, 15%	0.17	
	3421	TC316322	UP|Q5XPX5_SACOF (Q5XPX5) Actin		0.18
	4761	TC322283	Zea mays clone Contig305 mRNA sequence		-0.31
	4825	TC346820	UP|TBA6_MAIZE (P33627) Tubulin alpha-6 chain		0.17
	6195	TC367492	UP|TBA1_MAIZE (P14640) Tubulin alpha-1 chain		0.16
	6531	TC357833	RF|XP_506942.1|51964314|XM_506942 OJ1008_D06.15 gene product {Oryza sativa (japonica cultivar-group)}, 38%		-0.18
	6808	TC361986	UP|ADT1_MAIZE (P04709) ADP,ATP carrier protein 1, mitochondrial precursor	0.18	
	8819	TC362989	UP|TBA1_MAIZE (P14640) Tubulin alpha-1 chain	0.18	

		10234	TC328991	UP|TBB_HORVU (P93176) Tubulin beta chain, 96%		0.18
		10974	TC362989	UP|TBA1_MAIZE (P14640) Tubulin alpha-1 chain		0.20
		11678	TC335773	UP|TBB7_MAIZE (Q41784) Tubulin beta-7 chain, 52%		0.21
		12063	TC357833	RF|XP_506942.1|51964314|XM_506942 OJ1008_D06.15 gene product {Oryza sativa (japonica cultivar-group)}, 38%		0.22
		12570	TC316402	UP|Q8LLW2_ORYSA (Q8LLW2) Receptor-like kinase Xa21-binding protein 3, 84%		0.24
		12809	TC331173	UP|Q5ZEA0_ORYSA (Q5ZEA0) Kinesin heavy chain-like, 95%		-0.18
Development	Storage proteins	2356	TC340116	UP|Q41880_MAIZE (Q41880) Zein		-0.29
		15261	TC329291	UP|Q41884_MAIZE (Q41884) Zein		0.21
	Unspecified	1022	TC332106	Zea mays clone EL01N0526B05.d mRNA sequence	-0.21	
		1563	TC332381	UP|Q56YT3_ARATH (Q56YT3) Squamosa promoter binding protein-like 1, 25%		-0.26
		3671	TC335389	UP|Q656P7_ORYSA (Q656P7) Root hair defective 3 GTP-binding protein-like, 40%	-0.17	
		3869	TC360988	UP|Q654W0_ORYSA (Q654W0) G-box binding protein-like, 94%	-0.19	
		3959	TC342312	UP|Q9VNS7_DROME (Q9VNS7) CG14454-PA (CG32453-PA) (RE01153p), 19%	-0.18	
		8566	TC327894	UP|Q8LQG0_ORYSA (Q8LQG0) Leaf senescence protein-like, 36%		0.17
		8592	TC337080	UP|Q654W0_ORYSA (Q654W0) G-box binding protein-like, 94%		0.18
		8956	TC355702	UP|Q60EC2_ORYSA (Q60EC2) Unknow protein, 83%		0.16
		9321	TC337080	UP|Q654W0_ORYSA (Q654W0) G-box binding protein-like, 94%		0.20
		11752	TC360988	UP|Q654W0_ORYSA (Q654W0) G-box binding protein-like, 94%		0.23
		12494	TC360988	UP|Q654W0_ORYSA (Q654W0) G-box binding protein-like, 94%	0.19	0.22
DNA	Synthesis/chromatin structure	7053	TC342004	UP|Q9SX04_MAIZE (Q9SX04) Replication origin activator 2, 23%		0.17
		10551	TC317565	UP|DPOD2_ORYSA (Q9LRE5) DNA polymerase delta small subunit	0.19	

11429	TC327736	UP|O65573_ARATH (O65573) PRL1-associated protein-like protein, 25%		0.20
73	TC349142	UP|H2B5_MAIZE (P54348) Histone H2B		0.19
108	TC335527	UP|H2B5_MAIZE (P54348) Histone H2B, 98%		0.21
3265	TC364001	UP|H2A_MAIZE (P40280) Histone H2A, 57%		-0.20
3337	TC359088	UP|Q76MV0_TOBAC (Q76MV0) H3 histone		-0.17
3639	TC327808	UP|Q76MV0_TOBAC (Q76MV0) H3 histone	-0.20	
3686	TC355183	UP|H2B2_MAIZE (P30756) Histone H2B.2		0.17
4531	TC365788	UP|H2A_MAIZE (P40280) Histone H2A	-0.19	
4591	TC347498	UP|Q76MV0_TOBAC (Q76MV0) H3 histone	-0.19	
4964	TC332445	UP|H1_MAIZE (P23444) Histone H1, 98%		0.22
4988	TC329978	UP|H2B2_MAIZE (P30756) Histone H2B.2		-0.18
5773	TC329978	UP|H2B2_MAIZE (P30756) Histone H2B.2		0.18
6155	TC365788	UP|H2A_MAIZE (P40280) Histone H2A	-0.18	
6487	TC331139	UP|Q76MV0_TOBAC (Q76MV0) H3 histone		-0.19
6490	TC316760	UP|H1_MAIZE (P23444) Histone H1		-0.18
6590	TC327808	UP|Q76MV0_TOBAC (Q76MV0) H3 histone		-0.29
6886	TC336965	UP|Q8CGN9_MOUSE (Q8CGN9) Histone protein Hist2h3c1 (H3 histone, family 2), 78%	0.19	
7366	TC334942	UP|Q811M0_MOUSE (Q811M0) Hist1h4h protein, 97%		0.17
8109	TC342976	UP|H2AV3_ORYSA (Q84MP7) Probable histone H2A variant 3		-0.16
8972	TC357259	UP|H2A_MAIZE (P40280) Histone H2A		-0.22
9704	TC318102	UP|Q8LK07_MAIZE (Q8LK07) Histone H1-like protein HON101		0.19

		10824	TC341422	UP|Q811M0_MOUSE (Q811M0) Hist1h4h protein, 97%	0.17	
		11254	TC341533	UP|Q76N07_SOLME (Q76N07) Histone H4-like protein		0.18
		11268	TC338056	UP|Q76N07_SOLME (Q76N07) Histone H4-like protein	0.19	
		11364	TC363966	UP|Q76N07_SOLME (Q76N07) Histone H4-like protein		0.16
		12079	TC333345	UP|Q76N07_SOLME (Q76N07) Histone H4-like protein		0.22
	Unspecified	12629	TC323772	RF|XP_506641.1|51963722|XM_506641 P0523B07.38-1 gene product {Oryza sativa (japonica cultivar-group)}		0.17
Fermentation	Aldehyde dehydrogenase	10494	TC315983	UP|Q7FWR0_MAIZE (Q7FWR0) Mitochondrial aldehyde dehydrogenase RF2B		0.23
Gluconeogenese / glyoxylate cycle	Citrate synthase	10916	TC315933	Zea mays clone cr1.pk0029.c7, mRNA sequence		0.19
Glycolysis	PEPCK	10155	TC325108	UP|Q5EC59_MAIZE (Q5EC59) Phosphoenolpyruvate carboxylase kinase 1		0.23
	Pyrophosphate -fructose-6-P phosphotransf erase	4897	TC330834	RF|NP_192313.2|30679628|NM_116642 6-phosphofructokinase {Arabidopsis thaliana}, 27%	-0.17	
Hormone metabolism	Abscisic acid	15515	TC353513	UP|Q9SE96_ARATH (Q9SE96) FH protein interacting protein FIP1 (At1g28200/F3H9_12), 59%		0.18
	Auxin	12568	TC320382	GB|AAA33436.1|168422|MZEAUX auxin-binding protein precursor {Zea mays}		0.18
	Ethylene	1911	TC333057	Zea mays clone Contig194 mRNA sequence	-0.18	
		11367	TC316791			0.23
Lipid metabolism	Phospholipid synthesis	15282	TC349151	UP|Q8VYX1_WHEAT (Q8VYX1) Phosphoethanolamine methyltransferase, 98%		0.16
		12291	TC352163	GB|AAD29709.2|71164865|AF140496 cholinephosphate cytidylyltransferase {Oryza sativa (japonica cultivar-group)}, 83%	-0.18	
Major CHO metabolism	Synthesis	5159	TC316062	UP|Q947C0_MAIZE (Q947C0) ADP-glucose pyrophosphorylase small subunit		0.19
		5444	TC332324	UP|Q8GTK0_ORYSA (Q8GTK0) Granule binding starch synthase II, 33%		0.17

Metal handling	Binding, chelation and storage	2850	TC365565	UP|METK_ORYSA (P46611) S-adenosylmethionine synthetase 1		0.17
	Regulation	2653	TC350429	UP|ARD2_ORYSA (Q58FK4) 1, 2-dihydroxy-3-keto-5-methylthiopentene dioxygenase 2		0.17
		6338	TC331083	UP|ARD2_ORYSA (Q58FK4) 1, 2-dihydroxy-3-keto-5-methylthiopentene dioxygenase 2		0.24
		7623	TC350429	UP|ARD2_ORYSA (Q58FK4) 1, 2-dihydroxy-3-keto-5-methylthiopentene dioxygenase 2		0.19
Minor CHO metabolism	Callose	11076	TC329438	RF|NP_850271.1|30686934|NM_179940 ATGSL08 (GLUCAN SYNTHASE-LIKE 8) 1, 3-beta-glucan synthase/ transferase, transferring glycosyl groups {Arabidopsis thaliana}, 23%	0.21	
	Others	7854	TC315959	Zea mays clone Contig515 mRNA sequence		0.23
Misc	Other Ferredoxins and Rieske domain	11124	TC329071	UP|FER3_MAIZE (P27788) Ferredoxin-3, chloroplast precursor		0.27
	Glutathione S transferases	3772	TC316394	UP|Q9FQA9_MAIZE (Q9FQA9) Glutathione S-transferase GST 30		-0.29
		4431	TC322201	PRF|1303351A|225458|1303351A transferase,glutathione S. {Zea mays}		0.20
		6099	TC316394	UP|Q9FQA9_MAIZE (Q9FQA9) Glutathione S-transferase GST 30	-0.30	
		8078	TC322201	PRF|1303351A|225458|1303351A transferase,glutathione S. {Zea mays}		0.17
		8995	TC317007	UP|Q9FQC9_MAIZE (Q9FQC9) Glutathione S-transferase GST 10		-0.16
		9057	TC336687	PRF|1303351A|225458|1303351A transferase,glutathione S. {Zea mays}		0.22
		10192	TC317007	UP|Q9FQC9_MAIZE (Q9FQC9) Glutathione S-transferase GST 10	0.20	
		10819	TC336687	PRF|1303351A|225458|1303351A transferase,glutathione S. {Zea mays}		0.23
		12513	TC336687	PRF|1303351A|225458|1303351A transferase,glutathione S. {Zea mays}		0.20
	Oxidases - copper, flavone etc.	6616	TC321693	RF|NP_176759.1|15218830|NM_105256 amine oxidase/ oxidoreductase {Arabidopsis thaliana}, 84%		-0.18
		8059	TC371041	UP|CYPH_MAIZE (P21569) Peptidyl-prolyl cis-trans isomerase	0.23	

	UDP glucosyl and glucoronyl transferases	3467	TC317945	UP|Q84SY6_ORYSA (Q84SY6) Glycosyl transferase family 8 protein, 72%	-0.18	
		4750	TC316485	Zea mays clone EL01N0552D06.c mRNA sequence	-0.17	
Mitochondrial electron transport / ATP synthesis	Cytochrome c	11824	TC336065	UP|CYC_MAIZE (P00056) Cytochrome c		0.20
		14193	TC345346	UP|O50041_CHLRE (O50041) CCS1 protein (C-type cytochrome synthesis 1), 19%		-0.33
	F1-ATPase	5528	TC319963	UP|ATP4_IPOBA (Q40089) ATP synthase delta' chain, mitochondrial precursor, 88%	-0.17	
		6407	TC318927	Zea mays clone EL01N0316B09.c mRNA sequence		-0.19
		10222	TC352293	UP|Q9AYP0_ORYSA (Q9AYP0) Mitochondrial ATP synthase 6 KD subunit, 90%		0.22
	NADH-DH.localisation not clear	11307	TC357528	UP|N7BM_ARATH (Q9M9M9) Probable NADH-ubiquinone oxidoreductase subunit B17.2, 92%		0.20
Nucleotide metabolism	Phosphotransfer and pyrophosphatases.adenylate kinase	9055	TC340768	UP|KAD2_ORYSA (Q08480) Adenylate kinase B, 50%	0.20	
		11451	TC362735	UP|KAD2_ORYSA (Q08480) Adenylate kinase B		0.23
		1709	TC360950	PDB|1PKU_A|61679782|1PKU_A Chain A, Crystal Structure Of Nucleoside Diphosphate Kinase From Rice. {Oryza sativa}		0.18
		4434	TC332430	UP|NDK1_SACOF (P93554) Nucleoside diphosphate kinase 1		0.21
		10143	TC332430	UP|NDK1_SACOF (P93554) Nucleoside diphosphate kinase 1		0.23
		10185	TC332430	UP|NDK1_SACOF (P93554) Nucleoside diphosphate kinase 1	0.19	0.20
		12321	TC360950	PDB|1PKU_A|61679782|1PKU_A Chain A, Crystal Structure Of Nucleoside Diphosphate Kinase From Rice. {Oryza sativa}		0.19
	Salvage.phosphoribosyltransferases.aprt	7990	TC317036	UP|Q9LW89_HORVU (Q9LW89) Adenine phosphoribosyltransferase, 93%	0.27	
OPP	Electron transfer	11124	TC329071	UP|FER3_MAIZE (P27788) Ferredoxin-3, chloroplast precursor		0.27

	Non-reductive PP	10303	TC331852	UP|Q8RU73_SPIOL (Q8RU73) Chloroplast ribose-5-phosphate isomerase precursor, 80%		0.17
	Oxidative PP	7690	TC315976	UP|Q7FRX8_ORYSA (Q7FRX8) Cytosolic 6-phosphogluconate dehydrogenase, 98%		0.16
Polyamine metabolism	Synthesis	8529	TC368082	UP|DCAM_MAIZE (O24575) S-adenosylmethionine decarboxylase proenzyme		-0.19
Protein	Assembly and cofactor ligation	4438	TC339616	UP|Q7F270_ORYSA (Q7F270) ADP-ribosylation factor 1, 85%		0.19
	Amino acid activation	5784	TC340968	RF|NP_180591.1|15227735|NM_128585 pseudouridylate synthase/ tRNA-pseudouridine synthase {Arabidopsis thaliana} , 44%	-0.18	
		13374	TC369342	UP|Q41754_MAIZE (Q41754) Ubiquitin		0.17
		5736	TC329407	RF|XP_507519.1|51979332|XM_507519 OJ1717_A09.34 gene product {Oryza sativa (japonica cultivar-group)}, 31%	-0.19	
	Degradation	343	TC328083	UP|Q53PA1_ORYSA (Q53PA1) Serine carboxypeptidase, 64%	-0.20	
		4625	TC339421	RF|NP_172401.2|30681070|NM_100800 metalloexopeptidase/ metallopeptidase/ methionyl aminopeptidase/ peptidase {Arabidopsis thaliana}, 51%	-0.18	
		10803	TC331953	UP|O48555_MAIZE (O48555) Ubiquitin conjugating enzyme	0.24	
		14257	TC347585	UP|PSA2_ORYSA (Q9LSU2) Proteasome subunit alpha type 2	0.20	
		15540	TC316294	Zea mays clone Contig612.F mRNA sequence		0.23
		3574	TC327481	RF|NP_187347.2|30680058|NM_111571 cysteine-type peptidase {Arabidopsis thaliana}, 21%	-0.16	
		11204	TC359494	UP|Q43705_MAIZE (Q43705) Cysteine protease precursor		0.20
		1928	TC356405	UP|Q9ZP50_TOBAC (Q9ZP50) FtsH-like protein Pftf precursor, 90%		0.18
		1647	TC331627	UP|Q41753_MAIZE (Q41753) Ubiquitin fusion protein		0.18
		2024	TC342043	GB|BAD33626.1|50726105|AP005579 polyubiquitin 2 {Oryza sativa (japonica cultivar-group)}		0.17
		2677	TC327646	UP|Q75GT2_ORYSA (Q75GT2) Expressed protein, 97%	-0.16	

3204	TC336063	GB|BAD46215.1|52077170|AP005546 ubiquitin {Oryza sativa (japonica cultivar-group)}	-0.18	
8020	TC348584	UP|Q6LCT7_MAIZE (Q6LCT7) Ubiquitin fusion protein		-0.17
9033	TC342043	GB|BAD33626.1|50726105|AP005579 polyubiquitin 2 {Oryza sativa (japonica cultivar-group)}		0.17
11579	TC337097	GB|BAD46215.1|52077170|AP005546 ubiquitin {Oryza sativa (japonica cultivar-group)}, 89%		0.17
12575	TC348584	UP|Q6LCT7_MAIZE (Q6LCT7) Ubiquitin fusion protein		0.19
4037	TC330934	RF|NP_565834.1|18404032|NM_129165 ubiquitin conjugating enzyme/ ubiquitin-like activating enzyme {Arabidopsis thaliana}, 98%		-0.27
4364	TC336060	RF|NP_916873.1|34911052|NM_191984 ubiquitin-conjugating enzyme E2 {Oryza sativa (japonica cultivar-group)}	-0.17	
5230	TC325241	GB|AAL16250.1|16226747|AF428320 AT5g05080/MUG13_6 {Arabidopsis thaliana}, 77%		-0.26
11383	-	-		0.21
6441	TC318614	Zea mays clone EL01N0439F08.d mRNA sequence		-0.18
11033	TC340811	UP|O48700_ARATH (O48700) F3I6.27 protein, 27%		0.21
11369	TC338695	UP|Q5JNE3_ORYSA (Q5JNE3) RING zinc finger protein-like, 89%		0.18
11661	TC359015	RF|XP_506706.1|51963846|XM_506706 OJ1007_D04.4-2 gene product {Oryza sativa (japonica cultivar-group)}, 45%		0.20
12402	TC322618	UP|ATL1D_ARATH (Q8GW38) RING-H2 finger protein ATL1D, 47%	0.17	
14435	TC330059	UP|Q84PD9_ORYSA (Q84PD9) Ring zinc finger protein-like protein, 92%		0.25
11333	TC362648	UP|Q5ZC88_ORYSA (Q5ZC88) CUL1, 64%		0.19
13703	TC318589	UP|Q93VH7_ORYSA (Q93VH7) Cullin-like protein (CUL1), 49%		0.18
1724	TC317201	UP|Q9M7E5_MAIZE (Q9M7E5) Elongation factor 1 alpha		0.17
3799	TC369442	UP|Q9FER4_MAIZE (Q9FER4) 20S proteasome alpha subunit	-0.17	
6648	TC316315	UP|PSB1_ORYSA (O64464) Proteasome subunit beta type 1	0.18	

	9950	TC338345	UP|Q69IK6_ORYSA (Q69IK6) Proteasome maturation factor-like, 94%		0.25
	11096	TC316581	UP|Q9LST4_ORYSA (Q9LST4) Beta 7 subunit of 20S proteasome, 93%		0.19
	14794	TC325131	UP|PSA5_ORYSA (Q9LSU1) Proteasome subunit alpha type 5		0.25
	15581	TC317681	GB|BAB78489.1|17297983|AB037152 26S proteasome regulatory particle non-ATPase subunit11 {Oryza sativa (japonica cultivar-group)}		0.22
	1670	TC341370	UP|Q8RZ90_ORYSA (Q8RZ90) Ribosomal protein L18a-like, 81%		0.17
	1943	TC370401	UP|Q2RAM6_ORYSA (Q2RAM6) Ubiquitin family	-0.19	
	9946	TC339404	UP|SMT3_ORYSA (P55857) Ubiquitin-like protein SMT3, 93%		0.26
	10168	-	-		0.16
Folding	4975	TC316727	UP|Q53NM9_ORYSA (Q53NM9) DnaK-type molecular chaperone hsp70-rice		0.19
	6868	TC316727	UP|Q53NM9_ORYSA (Q53NM9) DnaK-type molecular chaperone hsp70-rice		0.16
	8893	TC319755	UP|Q6B4V4_VITVI (Q6B4V4) Chloroplast chaperonin 21, 81%		0.17
	10058	TC319755	UP|Q6B4V4_VITVI (Q6B4V4) Chloroplast chaperonin 21, 81%		0.20
	11379	TC316727	UP|Q53NM9_ORYSA (Q53NM9) DnaK-type molecular chaperone hsp70-rice		0.17
	11722	TC336547	UP|Q8H9B2_9ROSI (Q8H9B2) T-complex polypeptide 1		0.20
Glycosylation	8603	TC341714	UP|Q84T09_ORYSA (Q84T09) Unknow protein, 5'-partial, 63%	-0.18	
	12070	TC327579	Zea mays clone EL01N0553E10.d mRNA sequence		0.17
Postranslational modification	4504	TC360845	RF|NP_917765.1|34912836|NM_192876 Sgt1 {Oryza sativa (japonica cultivar-group)}, 97%		0.20
	4512	TC343915	RF|XP_506188.1|51963290|XM_506188 P0496D04.2 gene product {Oryza sativa (japonica cultivar-group)}, 67%		0.21
	5247	TC326787	UP|O24186_ORYSA (O24186) 10 kDa chaperonin		0.17
	5401	TC347134	UP|SAPK4_ORYSA (Q5N942) Serine/threonine-protein kinase SAPK4 (Osmotic stress/abscisic acid-activated protein kinase 4) , 98%	-0.17	

	9692	TC345397	UP|Q56I14_SOYBN (Q56I14) TPR-containing protein kinase, 60%		0.18
	10117	TC340620	UP|ZB14_MAIZE (P42856) 14 kDa zinc-binding protein (Protein kinase C inhibitor) (PKCI), 98%	0.19	
	11555	TC326787	UP|O24186_ORYSA (O24186) 10 kDa chaperonin		0.19
	12592	TC323670	UP|Q6IV73_MAIZE (Q6IV73) Protein phosphatase 2C		0.17
	15618	TC361611	RF|NP_915999.1|34909304|NM_191110 kinase-like protein {Oryza sativa (japonica cultivar-group)}, 8%		0.17
	9692	TC331057	UP|Q5MJV4_TOBAC (Q5MJV4) Avr9/Cf-9 rapidly elicited protein 261, 42%		0.18
	9820	TC324529	RF|NP_192172.1|15235432|NM_116497 ATP binding {Arabidopsis thaliana}, 69%	0.20	
	12044	TC370637	UP|M3KSL_DROME (Q95UN8) Mitogen-activated protein kinase kinase kinase, 3%		0.17
Synthesis.elongation	542	TC336969	UP|O50018_MAIZE (O50018) Elongation factor 1-alpha, 57%		0.20
	1673	TC350451	UP|Q9M7E5_MAIZE (Q9M7E5) Elongation factor 1 alpha		0.20
	1724	TC316293	UP|PSB3_ORYSA (Q9LST7) Proteasome subunit beta type 3		0.17
	6027	TC340493	UP|Q9M7E6_MAIZE (Q9M7E6) Elongation factor 1 alpha		0.19
	6169	TC337809	UP|O50018_MAIZE (O50018) Elongation factor 1-alpha	-0.18	
	8526	TC358596	UP|O50018_MAIZE (O50018) Elongation factor 1-alpha		0.18
	10178	TC340493	UP|Q9M7E6_MAIZE (Q9M7E6) Elongation factor 1 alpha	0.20	
	10855	TC367625	Zea mays clone EL01N0413C10.c mRNA sequence		0.18
	11275	TC364493	GB|AAO72574.1|29367403|AY224455 elongation factor 1 gamma-like protein {Oryza sativa (japonica cultivar-group)}, 60%		0.20
	12452	TC336969	UP|O50018_MAIZE (O50018) Elongation factor 1-alpha, 57%		0.17
	12454	TC337809	UP|O50018_MAIZE (O50018) Elongation factor 1-alpha		0.20
	15396	TC317201	UP|Q9M7E5_MAIZE (Q9M7E5) Elongation factor 1 alpha		0.17
Synthesis.initi	575	TC359771	UP|IF5A_MAIZE (P80639) Eukaryotic translation initiation factor 5A		0.17

ation	4155	TC322920	UP|Q9C5Y8_ARATH (Q9C5Y8) Initiation factor 3d, 65%		-0.23
	4895	TC363663	UP|IF5A_MAIZE (P80639) Eukaryotic translation initiation factor 5A	0.19	
	7676	TC363663	UP|IF5A_MAIZE (P80639) Eukaryotic translation initiation factor 5A		0.18
	8065	TC320007	GB|AAM52229.1|21464549|AY120686 At1g11480/T23J18_15 {Arabidopsis thaliana}, 9%		-0.17
	9735	TC351546	UP|Q2R678_ORYSA (Q2R678) Expressed protein		0.20
	11741	TC364749	UP|IF5A_MAIZE (P80639) Eukaryotic translation initiation factor 5A		0.18
	11794	TC345731	UP|IF1A_WHEAT (P47815) Eukaryotic translation initiation factor 1A		0.19
	12040	TC320119	GB|AAC17220.1|3108209|AF028809 novel cap-binding protein nCBP {Arabidopsis thaliana}, 88%		0.23
	12449	TC321218	UP|IF5A_MAIZE (P80639) Eukaryotic translation initiation factor 5A		0.17
Synthesis.misc ribososomal protein	25	TC344993	GB|AAP85547.1|32493112|AY323130 ribosomal protein large subunit 13 {Oryza sativa (japonica cultivar-group)}		0.17
	47	TC346361	GB|AAP21361.1|30102886|BT006553 At4g39880 {Arabidopsis thaliana}, 35%		0.17
	53	TC316836	UP|Q6RJY1_CAPAN (Q6RJY1) 60S ribosomal protein L12		0.17
	490	TC366592	UP|RL18A_ORYSA (Q943F3) 60S ribosomal protein L18a		0.16
	547	TC316578	UP|Q2QNF3_ORYSA (Q2QNF3) 60s ribosomal protein l2		0.18
	2159	TC336615	UP|Q9FJA6_ARATH (Q9FJA6) 40S ribosomal protein S3 (AT5g35530/MOK9_14), 86%		0.18
	2304	TC342218	RF|XP_506724.1|51963882|XM_506724 OJ9003_G05.34 gene product {Oryza sativa (japonica cultivar-group)}		0.17
	2405	TC342817	UP|RS12_HORVU (Q9XHS0) 40S ribosomal protein S12, 45%		-0.28
	2656	TC364589	Zea mays clone Contig325 mRNA sequence		0.20
	2815	TC324399	UP|Q9FJA6_ARATH (Q9FJA6) 40S ribosomal protein S3 (AT5g35530/MOK9_14), 88%		0.17
	3019	TC330969	UP|RS11_MAIZE (P25460) 40S ribosomal protein S11		0.19
	3234	TC349027	GB|AAX96401.1|62734292|AC133931 Ribosomal L38e protein family {Oryza sativa (japonica cultivar-group)}	-0.18	

3395	TC370212	UP|Q8H8S1_ORYSA (Q8H8S1) Ribosomal protein L15	-0.17	
3764	TC328196	Zea mays clone Contig301 mRNA sequence	-0.18	
4146	TC336615	UP|Q9FJA6_ARATH (Q9FJA6) 40S ribosomal protein S3 (AT5g35530/MOK9_14), 86%		0.19
4188	TC347178	UP|Q762A6_ORYSA (Q762A6) BRI1-KD interacting protein 108, 89%	-0.17	
4332	TC332130	UP|Q94AF6_ARATH (Q94AF6) AT5g20160/F5O24_50 (Ribosomal protein L7Ae-like), 97%	0.20	
4350	TC326208	UP|Q7XBH6_ORYSA (Q7XBH6) Ribosomal L9-like protein	-0.21	
4415	TC368426	UP|Q8W1C9_MAIZE (Q8W1C9) Ribosomal protein L35A		0.16
4449	TC351050	UP|Q6L5M1_BROIN (Q6L5M1) Glycoprotein, 97%	-0.18	
4475	TC316403	RF|XP_506804.1|51964042|XM_506804 P0483C08.42 gene product {Oryza sativa (japonica cultivar-group)}		0.18
4476	TC362382	UP|RS19_ORYSA (P40978) 40S ribosomal protein S19, 98%		0.18
4529	TC318771	UP|Q8GTE2_CICAR (Q8GTE2) Ribosomal protein RL5	-0.20	
4760	TC328072	UP|Q3HVL2_SOLTU (Q3HVL2) Ribosomal protein L27a-like protein, 91%	-0.20	
4892	TC324399	UP|Q9FJA6_ARATH (Q9FJA6) 40S ribosomal protein S3 (AT5g35530/MOK9_14), 88%		0.18
5325	TC358642	UP|Q5I7K8_WHEAT (Q5I7K8) Ribosomal protein l34, 97%	-0.19	
5631	TC335683	RF|XP_506724.1|51963882|XM_506724 OJ9003_G05.34 gene product {Oryza sativa (japonica cultivar-group)}		0.16
5673	TC364589	Zea mays clone Contig325 mRNA sequence		0.19
5770	TC323152	UP|Q9AV87_ORYSA (Q9AV87) 60S ribosomal protein L21		0.21
5777	TC326068	UP|Q9SM26_MAIZE (Q9SM26) Acidic ribosomal protein P2a-2		0.20
6031	TC363721	UP|Q7XR19_ORYSA (Q7XR19) 60S ribosomal protein L6		0.16
6040	TC368426	UP|Q8W1C9_MAIZE (Q8W1C9) Ribosomal protein L35A		0.19
6118	TC317715	Zea mays clone cho1c.pk003.i13, mRNA sequence		0.17

6126	TC329095	UP|ZEB2_MAIZE (P08031) Zein-beta precursor (16 kDa)	-0.19	
6343	TC324399	UP|Q9FJA6_ARATH (Q9FJA6) 40S ribosomal protein S3 (AT5g35530/MOK9_14), 88%		0.18
6402	TC364389	UP|RS15_ORYSA (P31674) 40S ribosomal protein S15, 89%		-0.20
6436	TC366592	UP|RL18A_ORYSA (Q943F3) 60S ribosomal protein L18a	0.18	
6608	TC323112	UP|RL7A_ORYSA (P35685) 60S ribosomal protein L7a		0.18
7025	TC323112	UP|RL7A_ORYSA (P35685) 60S ribosomal protein L7a		0.20
7146	TC338457	UP|Q9FUL7_MAIZE (Q9FUL7) 40S ribosomal protein S24, 94%		0.24
7150	TC369629	UP|Q5I7L1_WHEAT (Q5I7L1) Ribosomal protein L13a		0.22
7348	TC324494	RF|XP_473060.1|50926169|XM_473060 {Oryza sativa (japonica cultivar-group)}		0.21
7615	TC344993	GB|AAP85547.1|32493112|AY323130 ribosomal protein large subunit 13 {Oryza sativa (japonica cultivar-group)}	0.20	
7631	TC346815	GB|BAA02155.1|303853|RICRPL3A ribosomal protein L3 {Oryza sativa (japonica cultivar-group)}		0.17
7639	TC325255	UP|Q2R1J8_ORYSA (Q2R1J8) Ribosomal protein S4, 98%	0.21	
7653	TC356842	UP|Q6XC06_MAIZE (Q6XC06) Glyoxalase I, 22%	0.17	
7745	TC341415	UP|Q2R1J8_ORYSA (Q2R1J8) Ribosomal protein S4, 95%		0.18
7785	TC316306	UP|Q9FYS0_MAIZE (Q9FYS0) Ribosomal protein s6 RPS6-2		0.17
7849	TC346815	GB|BAA02155.1|303853|RICRPL3A ribosomal protein L3 {Oryza sativa (japonica cultivar-group)}	0.21	
8018	TC350465	UP|Q8GTE2_CICAR (Q8GTE2) Ribosomal protein RL5	0.26	
8020	TC348584	UP|Q6LCT7_MAIZE (Q6LCT7) Ubiquitin fusion protein		-0.17
8409	TC323771	UP|RS10_ORYSA (Q9AYP4) 40S ribosomal protein S10, 95%	0.19	
8428	TC345208	GB|AAP85547.1|32493112|AY323130 ribosomal protein large subunit 13 {Oryza sativa (japonica cultivar-group)}		0.24

8443	TC330969	UP|RS11_MAIZE (P25460) 40S ribosomal protein S11		0.18
8449	TC330576	UP|Q5I7L3_WHEAT (Q5I7L3) Ribosomal protein L10A		0.18
8451	TC330576	UP|Q5I7L3_WHEAT (Q5I7L3) Ribosomal protein L10A		0.18
8511	TC326068	UP|Q9SM26_MAIZE (Q9SM26) Acidic ribosomal protein P2a-2		0.18
8619	TC370785	UP|Q3MST7_ORYSA (Q3MST7) Ribosomal L32	0.19	
8882	TC329253		0.23	
8902	TC332667	UP|Q5I7K8_WHEAT (Q5I7K8) Ribosomal protein l34, 97%		0.18
8955	TC366592	UP|RL18A_ORYSA (Q943F3) 60S ribosomal protein L18a	0.22	
9210	TC338085	UP|Q7XR19_ORYSA (Q7XR19) 60S ribosomal protein L6	0.18	
9275	TC369026	UP|RS8_MAIZE (Q08069) 40S ribosomal protein S8		0.18
9379	TC324946	UP|RL7A_ORYSA (P35685) 60S ribosomal protein L7a		0.22
9543	TC316836	UP|Q6RJY1_CAPAN (Q6RJY1) 60S ribosomal protein L12	0.18	
9697	TC346005	UP|Q9FUL7_MAIZE (Q9FUL7) 40S ribosomal protein S24		0.17
9857	TC346170	Zea mays clone Contig334 mRNA sequence		0.18
10074	TC324499	UP|Q8RZ90_ORYSA (Q8RZ90) Ribosomal protein L18a-like, 91%	0.19	
10075	TC334532	UP|Q7XC31_ORYSA (Q7XC31) 60S ribosomal protein L27		0.19
10085	TC366533	RF|XP_506775.1|51963984|XM_506775 OJ1115_D03.49 gene product {Oryza sativa (japonica cultivar-group)}, 85%	0.18	
10088	TC323112	UP|RL7A_ORYSA (P35685) 60S ribosomal protein L7a	0.20	
10110	TC369629	UP|Q5I7L1_WHEAT (Q5I7L1) Ribosomal protein L13a		0.19
10113	TC321360	UP|Q9AV87_ORYSA (Q9AV87) 60S ribosomal protein L21	0.19	
10123	TC320924	UP|RL7A_ORYSA (P35685) 60S ribosomal protein L7a		0.19

10128	TC341880	UP|Q2R1J8_ORYSA (Q2R1J8) Ribosomal protein S4, 98%		0.18
10166	TC344993	GB|AAP85547.1|32493112|AY323130 ribosomal protein large subunit 13 {Oryza sativa (japonica cultivar-group)}		0.16
10189	TC324855	UP|Q7XR19_ORYSA (Q7XR19) 60S ribosomal protein L6		0.17
10214	TC354419	UP|Q5I7K3_WHEAT (Q5I7K3) Ribosomal protein S29		0.16
10345	TC316205	Zea mays clone EL01N0502H12.c mRNA sequence	0.18	
10347	TC318913	UP|Q8H8S1_ORYSA (Q8H8S1) Ribosomal protein L15		0.17
10429	TC316159	RF|XP_507392.1|51979030|XM_507392 B1056G08.113 gene product {Oryza sativa (japonica cultivar-group)}, 72%	0.21	
10441	TC317715	Zea mays clone cho1c.pk003.i13, mRNA sequence		0.21
10456	TC370212	UP|Q8H8S1_ORYSA (Q8H8S1) Ribosomal protein L15		0.17
10490	TC344993	GB|AAP85547.1|32493112|AY323130 ribosomal protein large subunit 13 {Oryza sativa (japonica cultivar-group)}	0.17	
10542	TC336888	RF|XP_507392.1|51979030|XM_507392 B1056G08.113 gene product {Oryza sativa (japonica cultivar-group)}, 73%	0.19	0.21
10545	TC334634	Zea mays clone Contig717.F mRNA sequence	0.17	
10592	TC316271	UP|O22453_MAIZE (O22453) Ribsomal protein S4		0.29
10774	TC368507	UP|Q6L5M1_BROIN (Q6L5M1) Glycoprotein, 97%		0.16
10810	TC335247	UP|RL24_HORVU (P50888) 60S ribosomal protein L24	0.17	
10869	TC330969	UP|RS11_MAIZE (P25460) 40S ribosomal protein S11		0.16
10881	TC364589	Zea mays clone Contig325 mRNA sequence		0.19
10895	TC369722	UP|RL41_ARATH (P62120) 60S ribosomal protein L41		0.18
10960	TC323112	UP|RL7A_ORYSA (P35685) 60S ribosomal protein L7a		0.18

11203	TC330576	UP|Q5I7L3_WHEAT (Q5I7L3) Ribosomal protein L10A	0.19	
11214	TC371050	UP|Q6K853_ORYSA (Q6K853) 40S ribosomal protein S30-like		0.18
11314	TC343031	RF|XP_507607.1|51979721|XM_507607 P0562A06.14 gene product {Oryza sativa (japonica cultivar-group)}		0.20
11330	TC336888	RF|XP_507392.1|51979030|XM_507392 B1056G08.113 gene product {Oryza sativa (japonica cultivar-group)}, 73%		0.18
11334	TC370380	UP|RLA0_MAIZE (O24573) 60S acidic ribosomal protein P0		0.17
11377	TC317715	Zea mays clone cho1c.pk003.i13, mRNA sequence	0.18	
11418	TC364589	Zea mays clone Contig325 mRNA sequence	0.18	
11458	TC362382	UP|RS19_ORYSA (P40978) 40S ribosomal protein S19, 98%		0.18
11558	TC324194	GB|AAL16201.1|16226563|AF428432 At2g02800/T20F6.6 {Arabidopsis thaliana}, 74%		0.17
11617	TC339626	UP|Q7GD83_ARATH (Q7GD83) 40S ribosomal protein S15A	0.21	
11692	TC316390	UP|Q7XY20_WHEAT (Q7XY20) Ribosomal protein L19, 89%		0.18
11695	TC324494	RF|XP_473060.1|50926169|XM_473060 {Oryza sativa (japonica cultivar-group)}		0.21
11704	TC335946	UP|RLA1_MAIZE (P52855) 60S acidic ribosomal protein P1 (L12)		0.19
11706	TC325572	UP|RS141_MAIZE (P19950) 40S ribosomal protein S14 (Clone MCH1)		0.22
11726	TC349051	UP|RLA2A_MAIZE (P46252) 60S acidic ribosomal protein P2A (P2)		0.18
11774	TC323766	UP|Q9FUL7_MAIZE (Q9FUL7) 40S ribosomal protein S24		0.20
11797	TC348097	UP|Q6SPR2_SOYBN (Q6SPR2) Ribosomal protein L37, 98%		0.24
11803	TC352496	Zea mays clone Contig349 mRNA sequence	0.19	
11924	TC328196	Zea mays clone Contig301 mRNA sequence		0.22
11945	TC364589	Zea mays clone Contig325 mRNA sequence		0.21
11951	TC363169	UP|Q7XY20_WHEAT (Q7XY20) Ribosomal protein L19, 89%		0.22

11956	TC361542	UP|Q5I7L5_WHEAT (Q5I7L5) Ribosomal protein L36, 93%		0.17
11973	TC371087	UP|Q5GMM4_CAPCH (Q5GMM4) 60S ribosomal protein L37a		0.18
12009	TC333658	UP|RS12_HORVU (Q9XHS0) 40S ribosomal protein S12, 91%		0.17
12015	TC325572	UP|RS141_MAIZE (P19950) 40S ribosomal protein S14 (Clone MCH1)		0.16
12020	TC331279	RF|XP_506724.1|51963882|XM_506724 OJ9003_G05.34 gene product {Oryza sativa (japonica cultivar-group)}		0.16
12113	TC342981	UP|RS26_ORYSA (P49216) 40S ribosomal protein S26 (S31), 91%		0.17
12133	TC346815	GB|BAA02155.1|303853|RICRPL3A ribosomal protein L3 {Oryza sativa (japonica cultivar-group)}		0.17
12417	TC321558	UP|Q2R4A1_ORYSA (Q2R4A1) Ribosomal protein S7		0.17
12418	TC336615	UP|Q9FJA6_ARATH (Q9FJA6) 40S ribosomal protein S3 (AT5g35530/MOK9_14), 86%		0.21
12460	TC330969	UP|RS11_MAIZE (P25460) 40S ribosomal protein S11		0.16
12476	TC326383	UP|Q8S2Y8_MAIZE (Q8S2Y8) Glycine-rich RNA binding protein		0.17
12477	TC325572	UP|RS141_MAIZE (P19950) 40S ribosomal protein S14 (Clone MCH1)		0.20
12517	TC329919	UP|Q9FUL7_MAIZE (Q9FUL7) 40S ribosomal protein S24		0.19
12575	TC348584	UP|Q6LCT7_MAIZE (Q6LCT7) Ubiquitin fusion protein		0.19
12586	TC317715	Zea mays clone cho1c.pk003.i13, mRNA sequence		0.17
12596	TC368816	UP|RL10_MAIZE (P45633) 60S ribosomal protein L10		0.22
12617	TC358005	UP|RS11_MAIZE (P25460) 40S ribosomal protein S11		0.17
12751	TC336123	UP|RLA2A_MAIZE (P46252) 60S acidic ribosomal protein P2A (P2)		0.22
13698	TC338957	UP|RS15_ORYSA (P31674) 40S ribosomal protein S15		0.19
15120	TC370380	UP|RLA0_MAIZE (O24573) 60S acidic ribosomal protein P0	0.22	
15401	TC332427	UP|RS26_ORYSA (P49216) 40S ribosomal protein S26 (S31), 94%	-0.25	

		15653	TC366445	UP|O04014_MAIZE (O04014) Ribosomal protein S6 RPS6-1		0.23
	Synthesis.mito /plastid ribosomal protein	1973	TC324442	UP|Q9XGC7_MAIZE (Q9XGC7) Iron sulfur subunit of succinate dehydrogenase (Truncated) and ribosomal protein S14 precursor	-0.18	
		10912	TC317556	UP|Q6H730_ORYSA (Q6H730) Ribosomal protein L12-like protein, 96%	0.22	
	Targeting.chloroplast	3916	TC351158	UP|Q67UZ3_ORYSA (Q67UZ3) Chloroplast thylakoidal processing peptidase-like protein, 75%	-0.20	
		14431	TC362534	RF|XP_507375.1|51978996|XM_507375 OJ1092_A07.131 gene product {Oryza sativa (japonica cultivar-group)}, 74%		0.18
	Targeting.mitochondria	2346	TC316309	Zea mays clone EL01N0323E09.c mRNA sequence	-0.18	
		5740	TC340295	UP|TIM8_ARATH (Q9XGY4) Mitochondrial import inner membrane translocase subunit Tim8, 87%		-0.26
	Targeting.secretory pathway	9265	TC318641	UP|Q8W403_ORYSA (Q8W403) Sec13p		0.16
		12599	TC316097	Zea mays clone Contig412 mRNA sequence	0.23	
		8465	TC324392	UP|Q2R135_ORYSA (Q2R135) Expressed protein, 97%		0.24
		11398	TC323133	UP|Q94GI1_ORYSA (Q94GI1) Clathrin assembly protein AP19-like protein, 88%		0.19
		729	TC350464	UP|Q2R0X0_ORYSA (Q2R0X0) Vacuolar sorting protein-like embryogenesis protein H beta 58-like protein	-0.25	
Photosynthesis	Calvin cycle	3766	TC369233	UP|TPIC_SECCE (P46225) Triosephosphate isomerase, chloroplast precursor, 88%		0.18
		8178	TC369233	UP|TPIC_SECCE (P46225) Triosephosphate isomerase, chloroplast precursor, 88%		0.18
		14881	TC370414	UP|ALFC_ORYSA (Q40677) Fructose-bisphosphate aldolase, chloroplast precursor	-0.21	
		10303	TC331852	UP|Q8RU73_SPIOL (Q8RU73) Chloroplast ribose-5-phosphate isomerase precursor, 80%		0.17
	Lightreaction	9820	TC324529	RF|NP_192172.1|15235432|NM_116497 ATP binding {Arabidopsis thaliana}, 69%	0.20	
	Photorespiration	8136	-	-	-0.25	
Redox	Ascorbate and glutathione.ascorbate	1247	TC325195	UP|Q65XA0_ORYSA (Q65XA0) Dehydroascorbate reductase		0.16
		3274	TC324341	GB|BAA08264.1|1321661|D45423 ascorbate peroxidase {Oryza sativa}		0.21

	3786	TC324341	GB|BAA08264.1|1321661|D45423 ascorbate peroxidase {Oryza sativa}		0.16
	4323	TC364641	UP|Q41772_MAIZE (Q41772) Cytosolic ascorbate peroxidase		0.21
	4555	TC324341	GB|BAA08264.1|1321661|D45423 ascorbate peroxidase {Oryza sativa}		0.16
	6038	TC317139	UP|Q65XA0_ORYSA (Q65XA0) Dehydroascorbate reductase		0.22
	6423	TC324341	GB|BAA08264.1|1321661|D45423 ascorbate peroxidase {Oryza sativa}		0.19
	6848	TC364641	UP|Q41772_MAIZE (Q41772) Cytosolic ascorbate peroxidase		0.18
	7239	TC364641	UP|Q41772_MAIZE (Q41772) Cytosolic ascorbate peroxidase		0.30
	8597	TC324341	GB|BAA08264.1|1321661|D45423 ascorbate peroxidase {Oryza sativa}		0.22
	12128	TC336263	GB|BAA08264.1|1321661|D45423 ascorbate peroxidase {Oryza sativa}		0.17
	12474	TC324341	GB|BAA08264.1|1321661|D45423 ascorbate peroxidase {Oryza sativa}		0.26
Dismutases and catalases	417	TC337070	UP|SODC5_MAIZE (P23346) Superoxide dismutase [Cu-Zn] 4AP	0.20	
	2091	TC337070	UP|SODC5_MAIZE (P23346) Superoxide dismutase [Cu-Zn] 4AP		0.18
	6186	TC329768	GB|AAA33511.1|168622|MZESOD2A SOD2 protein {Zea mays}	-0.18	
	6527	TC337070	UP|SODC5_MAIZE (P23346) Superoxide dismutase [Cu-Zn] 4AP		0.19
	6950	TC337070	UP|SODC5_MAIZE (P23346) Superoxide dismutase [Cu-Zn] 4AP		0.27
	8917	TC337070	UP|SODC5_MAIZE (P23346) Superoxide dismutase [Cu-Zn] 4AP		0.25
	8974	TC321188	UP|SODC4_MAIZE (P23345) Superoxide dismutase [Cu-Zn] 4A		0.21
	9375	TC337070	UP|SODC5_MAIZE (P23346) Superoxide dismutase [Cu-Zn] 4AP	0.20	0.22
	9722	TC329768	GB|AAA33511.1|168622|MZESOD2A SOD2 protein {Zea mays}		0.23
	10579	TC337070	UP|SODC5_MAIZE (P23346) Superoxide dismutase [Cu-Zn] 4AP		0.19
	10868	TC321188	UP|SODC4_MAIZE (P23345) Superoxide dismutase [Cu-Zn] 4A		0.22

		11315	TC337070	UP|SODC5_MAIZE (P23346) Superoxide dismutase [Cu-Zn] 4AP		0.24
		15295	TC321188	UP|SODC4_MAIZE (P23345) Superoxide dismutase [Cu-Zn] 4A	-0.19	
	Misc	9591	TC347454	UP|Q8W2K4_MAIZE (Q8W2K4) Cytochrome b5 reductase isoform II, 67%	0.21	
	Thioredoxin	8103	TC322717	GB|AAL25614.1|16648849|AY058202 AT4g04950/T1J1_6 {Arabidopsis thaliana}, 84%		0.19
RNA	Processing	3925	TC334832	UP|Q9FKB0_ARATH (Q9FKB0) Sm-like protein, 95%	-0.20	
		8422	TC334832	UP|Q9FKB0_ARATH (Q9FKB0) Sm-like protein, 95%	0.18	
		10106	TC353173	GB|AAM70547.1|21700847|AY124838 AT3g07590/MLP3_4 {Arabidopsis thaliana}	0.17	
		15081	TC348359	GB|AAO23652.1|27765062|BT003087 At2g03870 {Arabidopsis thaliana}, 94%	-0.23	
	Regulation of transcription	2230	TC319612	UP|O49216_ORYSA (O49216) Nucleic acid binding protein, 79%		-0.18
		10283	TC322823	UP|Q9LFY5_ARATH (Q9LFY5) T7N9.6, 31%		0.20
		4944	TC331266	UP|Q8W0W6_MAIZE (Q8W0W6) Repressor protein, 78%		-0.17
		10980	-	-	0.24	
		4623	TC315992	GB|AAM28228.1|20977602|AY093417 histone acetyl transferase {Zea mays}	-0.17	
		9175	TC342669	UP|Q5JKD1_ORYSA (Q5JKD1) Transcription factor jumonji (JmjC) domain-containing protein-like, 35%	0.21	
		2168	TC364640	UP|MNB1B_MAIZE (P27347) DNA-binding protein MNB1B		0.18
		3301	TC364640	UP|MNB1B_MAIZE (P27347) DNA-binding protein MNB1B	-0.18	
		3385	TC364640	UP|MNB1B_MAIZE (P27347) DNA-binding protein MNB1B		-0.22
		3429	TC361335	UP|P93630_MAIZE (P93630) HMGc1 protein	-0.18	
		3668	TC364640	UP|MNB1B_MAIZE (P27347) DNA-binding protein MNB1B	-0.19	
		6188	TC364640	UP|MNB1B_MAIZE (P27347) DNA-binding protein MNB1B	-0.17	
		6442	TC364640	UP|MNB1B_MAIZE (P27347) DNA-binding protein MNB1B		-0.15

6550	TC364640	UP|MNB1B_MAIZE (P27347) DNA-binding protein MNB1B		-0.21
6577	TC364640	UP|MNB1B_MAIZE (P27347) DNA-binding protein MNB1B		-0.19
6946	TC365371	UP|Q8W510_MAIZE (Q8W510) HMG type nucleosome/chromatin assembly factor D	-0.18	-0.21
6995	TC364640	UP|MNB1B_MAIZE (P27347) DNA-binding protein MNB1B		-0.18
7298	TC364640	UP|MNB1B_MAIZE (P27347) DNA-binding protein MNB1B		-0.17
7384	TC364640	UP|MNB1B_MAIZE (P27347) DNA-binding protein MNB1B		-0.21
7665	TC364640	UP|MNB1B_MAIZE (P27347) DNA-binding protein MNB1B		-0.20
7807	TC364640	UP|MNB1B_MAIZE (P27347) DNA-binding protein MNB1B		-0.20
8091	TC364640	UP|MNB1B_MAIZE (P27347) DNA-binding protein MNB1B		-0.17
8159	TC364640	UP|MNB1B_MAIZE (P27347) DNA-binding protein MNB1B		-0.18
8425	TC364640	UP|MNB1B_MAIZE (P27347) DNA-binding protein MNB1B		-0.19
8545	TC364640	UP|MNB1B_MAIZE (P27347) DNA-binding protein MNB1B		-0.16
8929	TC364640	UP|MNB1B_MAIZE (P27347) DNA-binding protein MNB1B		-0.22
9770	TC364640	UP|MNB1B_MAIZE (P27347) DNA-binding protein MNB1B		-0.19
10216	TC364640	UP|MNB1B_MAIZE (P27347) DNA-binding protein MNB1B		-0.17
4011	TC325028	UP|PCNA_MAIZE (Q43266) Proliferating cell nuclear antigen (PCNA)		-0.20
10025	TC324617	UP|Q9LTV0_ARATH (Q9LTV0) Nucleolar protein, 86%	0.21	
10721	TC327074	UP|Q9LHA3_ARATH (Q9LHA3) Arabidopsis thaliana genomic DNA, chromosome 3, BAC clone: T19N8, 26%	0.18	
10834	TC346179	UP|Q7XIG8_ORYSA (Q7XIG8) AcinusL protein-like, 22%	0.20	
1563	TC332381	UP|Q56YT3_ARATH (Q56YT3) Squamosa promoter binding protein-like 1, 25%		-0.26
11304	TC319117	UP|ATXR2_ARATH (Q5PP37) Histone-lysine N-methyltransferase ATXR2 , 29%		0.19

		380	TC334418	UP|PFD5_ARATH (P57742) Probable prefoldin subunit 5, 83%	-0.18	
		1755	TC316620	UP|Q6F4N5_ORYSA (Q6F4N5) Radc1, 97%		-0.21
		8157	TC365388	RF|NP_196708.1|15239055|NM_121185 DNA binding {Arabidopsis thaliana}, 3%	-0.27	
	RNA binding	229	TC344711	UP|P91632_DROME (P91632) CG7437-PA, isoform A (Nucleic acid binding protein), 5%		0.21
		535	TC318428	Zea mays clone EL01N0360D09.c mRNA sequence		-0.18
		12747	TC353332	UP|Q70KT2_ORYSA (Q70KT2) TA8 protein	0.21	
		10529	TC329493	RF|NP_178540.1|15224039|NM_126492 DNA binding {Arabidopsis thaliana}, 80%		0.19
Secondary metabolism	Isoprenoids	10523	TC323304	UP|Q9XHH0_MAIZE (Q9XHH0) Acetoacetyl CoA thiolase, 92%	0.20	
		7292	TC352186	UP|HMDH_MAIZE (O24594) 3-hydroxy-3-methylglutaryl-coenzyme A reductase, 28%		0.24
Signaling	14-3-3 proteins	8162	TC316225	UP|Q6XNL1_9POAL (Q6XNL1) 14-3-3-like protein		0.17
		10612	TC326875	GB|AAA33505.1|168603|MZEREGP regulatory protein {Zea mays}, 66%		0.18
	Calcium	2275	TC359253	UP|POLC2_JUNOX (O64943) Polcalcin Jun o 2 (Calcium-binding pollen allergen Jun o 2), 27%	-0.19	
		3273	TC336540	UP|Q41798_MAIZE (Q41798) Calnexin, 97%		0.17
		3942	TC348942	UP|Q9SDJ0_ORYSA (Q9SDJ0) Calmodulin, 95%	-0.19	
		4921	TC339753	UP|CALX_HELTU (Q39994) Calnexin homolog precursor, 39%		0.23
		8081	TC364854	UP|Q43712_MAIZE (Q43712) Calcium-binding protein precursor (Calreticulin)		0.17
		9765	TC336540	UP|Q41798_MAIZE (Q41798) Calnexin, 97%		0.26
		11459	TC336540	UP|Q41798_MAIZE (Q41798) Calnexin, 97%	0.19	
		11776	TC336540	UP|Q41798_MAIZE (Q41798) Calnexin, 97%		0.29
		12081	TC345850	UP|O49184_ORYSA (O49184) Calmodulin		0.16
		12525	TC345850	UP|O49184_ORYSA (O49184) Calmodulin		0.17

		14870	TC350699	UP|Q3HRP0_ORYSA (Q3HRP0) Calcineurin B-like protein 7, 38%	0.21	
	G-proteins	834	TC357523	UP|Q94K24_LYCES (Q94K24) Ran binding protein-1, 62%	0.18	
		3295	TC357523	UP|Q94K24_LYCES (Q94K24) Ran binding protein-1, 62%		0.19
		3671	TC335389	UP|Q656P7_ORYSA (Q656P7) Root hair defective 3 GTP-binding protein-like, 40%	-0.17	
		4961	TC319276	RF|NP_193883.2|30685465|NM_118272 nucleotide binding {Arabidopsis thaliana}, 66%	-0.20	
		8153	TC361666	UP|Q7GD79_ORYSA (Q7GD79) Small GTP-binding protein (Ran2)		0.23
		9560	TC366622	GB|AAT28677.1|47499878|AY620417 GTP-binding protein {Oryza sativa (japonica cultivar-group)}		0.23
		11058	TC326054	UP|O81695_AVEFA (O81695) Ras-like small monomeric GTP-binding protein		0.19
		11313	TC348968	UP|Q94K24_LYCES (Q94K24) Ran binding protein-1, 63%		0.18
		12504	TC345387	UP|Q7GD79_ORYSA (Q7GD79) Small GTP-binding protein (Ran2)		0.24
		13531	TC336372	UP|RGP1_ORYSA (P25766) Ras-related protein RGP1 (GTP-binding regulatory protein RGP1), 98%		0.17
	Light	15406	TC371218	UP|Q9M6R3_ORYSA (Q9M6R3) Constitutive photomorphogenic 11, 31%	-0.21	
	Phosphinositides	3374	TC320095	UP|Q33BI9_ORYSA (Q33BI9) Inositol 1, 3, 4-trisphosphate 5/6-kinase, 88%		-0.18
		9438	TC335200	UP|Q6ZLF2_ORYSA (Q6ZLF2) 1-phosphatidylinositol-3-phosphate 5-kinase-like, 17%		0.23
	Receptor kinases	12274	TC352360	UP|Q8LA44_ARATH (Q8LA44) Receptor protein kinase-like protein, 25%		0.19
		13361	TC329137	UP|Q5JK22_ORYSA (Q5JK22) Systemin receptor-like, 92%		0.21
Stress	Abiotic.cold	8994	TC326521	Zea mays clone EL01N0323A01.c mRNA sequence	0.23	
		10254	TC360345	Zea mays clone Contig55 mRNA sequence	0.20	
		12520	TC326521	Zea mays clone EL01N0323A01.c mRNA sequence		0.21
	Abiotic.heat	3361	TC316727	UP|Q53NM9_ORYSA (Q53NM9) DnaK-type molecular chaperone hsp70-rice		0.14
		3546	TC346999	UP|Q8RV04_ARATH (Q8RV04) Expressed protein (DNAJ protein-like)	-0.18	

3714	TC340577	Zea mays clone Contig439 mRNA sequence		0.17
4368	TC337494	UP|HSP81_ORYSA (P33126) Heat shock protein 81-1		0.19
4878	TC344345	UP|Q5EBY7_MAIZE (Q5EBY7) Heat shock protein 70, 84%		0.19
4975	TC316727	UP|Q53NM9_ORYSA (Q53NM9) DnaK-type molecular chaperone hsp70-rice		0.19
5326	TC316384	UP|Q76B83_ORYSA (Q76B83) Heat shock protein 90, 60%		0.21
5710	TC349327	UP|HSP81_ORYSA (P33126) Heat shock protein 81-1		0.17
6524	TC349327	UP|HSP81_ORYSA (P33126) Heat shock protein 81-1		0.21
6865	TC335657	UP|HSP7M_PHAVU (Q01899) Heat shock 70 kDa protein, mitochondrial precursor, 43%		0.25
6868	TC316727	UP|Q53NM9_ORYSA (Q53NM9) DnaK-type molecular chaperone hsp70-rice		0.16
7717	TC352555	UP|Q9SWB5_SOYBN (Q9SWB5) Seed maturation protein PM37		0.16
9003	TC316042	UP|Q8SB39_ORYSA (Q8SB39) Heat shock protein 90, 54%		0.19
9221	TC340577	Zea mays clone Contig439 mRNA sequence		0.22
9839	TC334144	UP|Q43638_SECCE (Q43638) Heat-shock protein precursor, 16%		0.23
9842	TC321298	UP|HSP81_ORYSA (P33126) Heat shock protein 81-1, 64%		0.17
11176	TC321442	UP|O50047_SPIOL (O50047) Heat shock 70 protein, 40%		0.18
11308	TC334920	UP|Q9LHA8_ARATH (Q9LHA8) 70 kDa heat shock protein (AT3g12580/T2E22_110), 75%		0.17
11317	TC364289	UP|Q43638_SECCE (Q43638) Heat-shock protein precursor, 39%		0.17
11379	TC316727	UP|Q53NM9_ORYSA (Q53NM9) DnaK-type molecular chaperone hsp70-rice		0.17
12159	TC347411	UP|Q9MB32_ORYSA (Q9MB32) Heat shock protein 90, 19%		0.17
12357	TC349327	UP|HSP81_ORYSA (P33126) Heat shock protein 81-1		0.21
14671	TC320707	UP|Q53NM9_ORYSA (Q53NM9) DnaK-type molecular chaperone hsp70-rice (DnaK-type molecular chaperone hsp70), 38%		0.18

		14991	TC337494	UP|HSP81_ORYSA (P33126) Heat shock protein 81-1		0.21
		15561	TC342488	GB|AAL25548.1|16648712|AY058132 AT3g62190/T17J13_150 {Arabidopsis thaliana}, 56%	0.21	
	Abiotic.unspecified	2275	TC359253	UP|POLC2_JUNOX (O64943) Polcalcin Jun o 2 (Calcium-binding pollen allergen Jun o 2), 27%	-0.19	
		13697	TC342426	UP|Q6TM44_MAIZE (Q6TM44) Germin-like protein, 66%		0.25
TCA / org	Transformation	2235	TC369443	Zea mays clone Contig506.F mRNA sequence	-0.18	
		6199	TC338918	UP|MDHC_MAIZE (Q08062) Malate dehydrogenase, cytoplasmic		0.17
		4378	TC327187	UP|Q9SWR9_MAIZE (Q9SWR9) Dihydrolipoamide S-acetyltransferase	-0.18	
		11291	TC318675	UP|Q9SWR9_MAIZE (Q9SWR9) Dihydrolipoamide S-acetyltransferase		0.21
Transport	Amino acids	15205	TC354722	UP|Q4H2F4_MOUSE (Q4H2F4) Sublingual acinar membrane protein, 4%	-0.21	
	Major Intrinsic Proteins.PIP	211	TC369033	UP|Q9XF59_MAIZE (Q9XF59) Plasma membrane MIP protein		0.22
		9072	TC316339	UP|Q9AQU5_MAIZE (Q9AQU5) Plasma membrane integral protein ZmPIP1-4		0.22
		10026	TC369033	UP|Q9XF59_MAIZE (Q9XF59) Plasma membrane MIP protein		0.20
		12470	TC370281	UP|PTH2_DROME (O97067) Probable peptidyl-tRNA hydrolase 2 (PTH 2) , 22%		0.23
	Transport.Major Intrinsic Proteins.TIP	2056	TC340492	UP|O64964_MAIZE (O64964) Tonoplast intrinsic protein		0.17
		2417	TC340492	UP|O64964_MAIZE (O64964) Tonoplast intrinsic protein		0.18
		2801	TC340492	UP|O64964_MAIZE (O64964) Tonoplast intrinsic protein		0.19
		5233	TC369263			0.25
		6576	TC340492	UP|O64964_MAIZE (O64964) Tonoplast intrinsic protein		0.22
		7302	TC340492	UP|O64964_MAIZE (O64964) Tonoplast intrinsic protein		0.22
		11702	TC340492	UP|O64964_MAIZE (O64964) Tonoplast intrinsic protein		0.19
	Metabolite	3355	TC341294	Zea mays clone Contig981.F mRNA sequence	-0.21	

	transporters at the mitochondrial membrane	6808	TC326808	UP|ADT1_MAIZE (P04709) ADP,ATP carrier protein 1, mitochondrial precursor , 60%	0.18	
	Metal	12172	TC322120	UP|Q84ND6_STYHA (Q84ND6) Cation diffusion facilitator 8, 35%		0.18
	Misc	22	TC357671	RF|XP_507389.1|51979024|XM_507389 OJ1003_H02.130 gene product {Oryza sativa (japonica cultivar-group)}, 18%	0.21	
		11670	TC324295	UP|Q8GRK9_ORYSA (Q8GRK9) Transport protein particle component Bet3-like protein, 97%		0.19
		12629	TC323772	RF|XP_506641.1|51963722|XM_506641 P0523B07.38-1 gene product {Oryza sativa (japonica cultivar-group)}		0.17
	P- and v-ATPases	13426	TC350657	UP|Q8GUB2_MESCR (Q8GUB2) Vacuolar ATPase subunit e-like, 94%		-0.33
		453	TC343388	UP|Q945E8_PENAM (Q945E8) Vacuolar H+-ATPase 16 kDa proteolipid subunit c		0.20
		9256	TC353176	UP|Q945E8_PENAM (Q945E8) Vacuolar H+-ATPase 16 kDa proteolipid subunit c		0.21
		10939	-	-		0.23
		11218	TC336271	UP|VATB1_HORVU (Q40078) Vacuolar ATP synthase subunit B isoform 1		0.18
		12126	TC329656	UP|Q945E8_PENAM (Q945E8) Vacuolar H+-ATPase 16 kDa proteolipid subunit c		0.18
		12637	TC340755	UP|Q94G11_ORYCO (Q94G11) V-ATPase subunit c, 65%		0.23
	Potassium	3873	TC338613	RF|NP_176222.2|42562825|NM_104706 potassium ion transporter {Arabidopsis thaliana}, 49%		-0.29
		9291	TC344894	UP|HAK2_ORYSA (Q942X8) Probable potassium transporter 2 (OsHAK2), 24%		0.18
	Unspecified anions	3812	TC320818	UP|Q2L3B0_BRASY (Q2L3B0) Chloride channel-f protein, 14%	-0.18	
Not assigned	No ontology	289	TC319593	RF|XP_450402.1|50899228|XM_450402 BolA-like family protein {Oryza sativa (japonica cultivar-group)}, 74%		0.17
		814	TC370071	UP|Q8H6A5_MAIZE (Q8H6A5) Translationally controlled tumor protein-like protein	0.18	
		1659	TC321171	UP|Q9LK52_ARATH (Q9LK52) Dbj|BAA90629.1		0.20

2107	TC329533	GB|AAK91338.1|15215586|AY050321 AT4g23630/F9D16_100 {Arabidopsis thaliana}, 78%		0.22
2204	TC339339	UP|Q8RW94_ARATH (Q8RW94) At1g80210/F18B13_28, 10%	-0.20	
2378	TC346485		-0.18	
2683	TC337690	UP|Q9SUL3_ARATH (Q9SUL3) OBP33PEP like protein, 59%	-0.18	
3769	TC318294	UP|Q337S2_ORYSA (Q337S2) HR-like lesion-inducing	-0.19	
3878	TC349744	UP|NUD23_ARATH (P93740) Nudix hydrolase 23, chloroplast precursor (AtNUDT23) , 69%	-0.20	
4131	TC318488	UP|Q7XI46_ORYSA (Q7XI46) Hydrolase-like protein, 72%		-0.32
4234	TC336299	RF|XP_506823.1|51964080|XM_506823 P0470G10.26 gene product {Oryza sativa (japonica cultivar-group)}, 71%	-0.17	
4243	TC324206	UP|Q67U13_ORYSA (Q67U13) Translocation protein-related-like, 90%	-0.17	
4322	TC343175	RF|NP_191890.1|15229411|NM_116196 glutamate binding {Arabidopsis thaliana}, 60%	-0.18	
4357	TC327629	RF|XP_507481.1|51979234|XM_507481 OJ1342_D02.8 gene product {Oryza sativa (japonica cultivar-group)}, 82%	-0.19	
4507	TC317946	UP|Q3MST6_ORYSA (Q3MST6) Peptidyl prolyl cis-trans isomerase	-0.18	
4742	TC316827	UP|Q5UDB6_MAIZE (Q5UDB6) INDETERMINATE-related protein 9	-0.19	
4749	TC331877	RF|XP_507328.1|51965088|XM_507328 OJ1125_C01.28 gene product {Oryza sativa (japonica cultivar-group)}, 31%	-0.21	
4751	TC326894	UP|Q9AR47_MAIZE (Q9AR47) VIP3 protein	-0.19	
5223	TC316196	UP|Q9M588_MAIZE (Q9M588) Prohibitin	-0.19	
5575	TC330725		-0.17	
6413	TC331442		0.20	
6659	TC330208	GB|AAK32844.1|13605702|AF361832 AT3g51520/F26O13_160 {Arabidopsis thaliana}, 82%		-0.19
7699	-	-		0.18

	8758	TC357811	UP|Q6JB14_MAIZE (Q6JB14) MEG2	0.18	
	9056	TC328093	GB|AAD31570.1|4883601|AC006922 expressed protein {Arabidopsis thaliana}, 68%	0.21	
	9195	TC331398		0.21	
	9656	TC318893	UP|Q6Z1N4_ORYSA (Q6Z1N4) Type 1 membrane protein-like, 87%		0.19
	9787	TC324578	RF|NP_200011.1|15242242|NM_124577 nucleic acid binding {Arabidopsis thaliana}, 70%		0.22
	10601	TC340570	UP|Q337C1_ORYSA (Q337C1) Expressed protein, 47%	0.18	
	11207	TC326846		0.20	
	11500	TC346339	UP|Q9LK52_ARATH (Q9LK52) Dbj|BAA90629.1, 97%		0.18
	12233	TC323187	Zea mays clone EL01N0444B10.c mRNA sequence		0.28
	12473	TC370071	UP|Q8H6A5_MAIZE (Q8H6A5) Translationally controlled tumor protein-like protein		0.16
	12723	TC348925			0.19
	14209	TC343018	UP|Q2VCI0_SOLTU (Q2VCI0) Translocon-associated protein beta family protein-like, 70%		0.21
	14649	TC345176	UP|Q2R2S2_ORYSA (Q2R2S2) Maf-like protein, 85%		-0.36
	15508	TC362224	UP|Q6Z7V1_ORYSA (Q6Z7V1) LMBR1 integral membrane protein-like, 20%		0.20
	15678	TC339386	RF|NP_198706.1|15241578|NM_123252 protein disulfide oxidoreductase {Arabidopsis thaliana}, 85%		0.18
	1980	TC343467	UP|Q651U7_ORYSA (Q651U7) Phosphoprotein-like, 19%		-0.19
	3747	TC323380	UP|Q41719_ZEADI (Q41719) Hydroxyproline-rich glycoprotein precursor, 98%	-0.18	
	3779	TC340260	Zea mays clone Contig275 mRNA sequence		0.17
	6685	TC339113	UP|Q9SCP4_ARATH (Q9SCP4) Nodulin / glutamate-ammonia ligase-like protein, 17%	0.19	
Unknown	1819	TC346432			-0.18
	1971	TC358473		-0.19	

2389	TC330254	RF|XP_507377.1|51979000|XM_507377 OJ1699_E05.22-1 gene product {Oryza sativa (japonica cultivar-group)}, 80%	-0.22	
2451	TC366138	UP|Q9VL71_DROME (Q9VL71) CG4602-PA (LD29830p), 5%		0.15
2562	TC320051	UP|Q9SXG0_ORYSA (Q9SXG0) F1F0-ATPase inhibitor protein, 93%		0.16
2641	TC330547	UP|Q2V3F4_ARATH (Q2V3F4) Protein At4g24380, 68%		-0.26
3228	TC317029	UP|Q5EUJ4_MAIZE (Q5EUJ4) Terminal acidic SANT 1	-0.18	
3549	TC334240	GB|BAD06873.1|40714351|AB111915 replication protein A 14kDa {Oryza sativa (japonica cultivar-group)}	-0.18	
3598	TC335984	RF|XP_506646.1|51963732|XM_506646 P0651G05.38 gene product {Oryza sativa (japonica cultivar-group)}	-0.17	
3782	TC337035	UP|Q6X1Z6_9ALPH (Q6X1Z6) BICP22 transcription factor, 8%	-0.18	
3867	TC345806	UP|Q6A4S5_DROME (Q6A4S5) SGG, 11%	-0.19	
4272	TC351821			-0.24
4402	TC366047	UP|Q5U7K6_9POAL (Q5U7K6) Metallothionein-like protein, 93%	-0.17	
6410	TC339717	UP|U139_ARATH (Q9SD88) UPF0139 protein At5g07960, 85%		-0.19
6418	TC349259	GB|AAC28224.1|3377842|T27D20 {Arabidopsis thaliana}, 65%		-0.27
6540	TC318327	UP|Y3377_ARATH (Q6ID70) Protein At3g03773, 68%		-0.22
7239	TC327906	UP|Q5YLM3_MAIZE (Q5YLM3) Roothairless 1		0.30
7606	TC328615	UP|Q2HIG7_ARATH (Q2HIG7) At4g26550, 90%		0.16
8112	TC356037			-0.18
8566	TC320124	UP|Q8LQG0_ORYSA (Q8LQG0) Leaf senescence protein-like, 64%		0.17
8881	TC342886	UP|Q8RX56_ARATH (Q8RX56) AT5g06970/MOJ9_14, 10%	0.22	
9538	TC323560	UP|Q5Z8P6_ORYSA (Q5Z8P6) Transcription factor-like, 50%		-0.25
10826	TC363203	UP|Q5NAX2_ORYSA (Q5NAX2) Ubiquitin-conjugating enzyme-like, 59%	0.23	

	10940	TC325872	GB|AAP54551.1|31432987|AE016959 expressed protein {Oryza sativa (japonica cultivar-group)}, 58%		0.22
	11547	TC338201	UP|U139_ARATH (Q9SD88) UPF0139 protein At5g07960, 85%	-0.19	
	12001	TC327546	UP|Q9SXG0_ORYSA (Q9SXG0) F1F0-ATPase inhibitor protein, 93%		0.16
	13074	TC350009	UP|O82167_ARATH (O82167) Expressed protein (At2g35260/T4C15.7), 22%		-0.40
	15035	TC322578	Zea mays clone EL01N0521H06.c mRNA sequence		0.18
No homology in Arabidopsis	664	-	-		-0.36
	797	TC326608	RF|NP_188755.2|22331235|NM_113013 ACA9 calcium-transporting ATPase/ calmodulin binding {Arabidopsis thaliana}, 17%		-0.30
	1030	TC326949			-0.24
	1175	TC365024	UP|Q69K57_ORYSA (Q69K57) Smr domain-containing protein-like, 5%		-0.18
	1562	TC340137	UP|Q74M80_NANEQ (Q74M80) NEQ455, 7%		-0.35
	1839	TC348917	UP|Q38HS8_SOLTU (Q38HS8) Ribosomal protein L23 family protein, 52%	-0.18	
	1970	TC356464		-0.19	
	1976	TC359952		-0.19	
	2241	TC366500	Zea mays clone EL01N0442A09.c mRNA sequence	-0.22	
	2269	TC365915	UP|Q2QSC6_ORYSA (Q2QSC6) Expressed protein, 36%	-0.25	
	2377	TC345126	UP|Q9Z5T4_ZYMMO (Q9Z5T4) Basal-body rod modification protein, 7%	-0.17	
	2379	TC355308	UP|Q9SD81_ARATH (Q9SD81) Glycerophosphodiester phosphodiesterase-like protein (At5g08030), 8%	-0.19	
	2396	TC332870	UP|Q339L3_ORYSA (Q339L3) Expressed protein, 57%		-0.25
	2666	TC325950	UP|Q7X9C0_LOTJA (Q7X9C0) NIN-like protein 2, 32%		-0.26
	2669	TC357609	UP|Q2RAM6_ORYSA (Q2RAM6) Ubiquitin family, 21%		0.23
	2692	-	-		-0.21

2723	TC343045		-0.23	
3045	-	-		-0.25
3331	TC342290	UP|O23965_HELAN (O23965) Dc3 promoter-binding factor-2, 24%	-0.19	
3383	TC349665	UP|Q3H3A5_9ACTO (Q3H3A5) Uncharacterised conserved protein UCP005026, 3%	-0.19	
3388	TC341949	UP|Q40ZX5_KINRA (Q40ZX5) Aldo/keto reductase, 4%	-0.17	
3420	TC353259		-0.17	
3437	TC370236	GB|AAK60291.1|14326491|AF385698 At2g43780/F18O19.11 {Arabidopsis thaliana}, 70%	-0.17	
3563	TC348586	GB|AAA83618.1|1125842|U43375 Sulfatase domain protein protein 1 {Caenorhabditis elegans} , 3%	-0.21	
3578	TC335689			-0.20
3659	TC361585	GB|AAQ88769.1|37181931|AY358403 RASL651 {Homo sapiens}, 3%		-0.20
3688	TC318199	UP|Q5ZCB3_ORYSA (Q5ZCB3) Seven in absentia protein-like, 17%	-0.18	
3752	TC334717	Zea mays clone Contig483 mRNA sequence	-0.17	
3840	TC326422	UP|Q99367_SOYBN (Q99367) DNA-directed RNA polymerase, 53%	-0.17	
3976	TC352574		-0.20	
4266	TC328370	UP|Q5ZC67_ORYSA (Q5ZC67) Serine/threonine-specific protein kinase-like, 34%	-0.17	
4358	TC342192		-0.20	
4776	TC340131	UP|Q6K9T9_ORYSA (Q6K9T9) Metallo-beta-lactamase-like, 66%	-0.18	
5168	TC337016	UP|Q3GT62_9ACTO (Q3GT62) Phosphoglycerate/bisphosphoglycerate mutase:RNase H, 4%		-0.20
5178	TC345640		-0.17	
5240	TC331387	UP|Q8W4Q5_ARATH (Q8W4Q5) AT5g51450/MFG13_16, 10%		-0.24
5364	TC335058		-0.17	

5540	TC333108	emb|Z00028.1|CHZMRRNA Zea mays chloroplast rRNA-operon, 11%	-0.25	
5573	TC334655	RF|NP_910156.1|34897620|NM_185267 kinesin-like protein {Oryza sativa (japonica cultivar-group)}, 20%	-0.20	
5577	TC342504		-0.19	
5585	TC340240	UP|Q7XBA5_ORYSA (Q7XBA5) Drought-induced protein DI1, 36%	-0.19	
5648	-	-	-0.17	
5844	TC351115		-0.18	
5855	TC325355	RF|NP_198865.1|15242719|NM_123413 RNA binding {Arabidopsis thaliana}, 47%	-0.20	
6515	TC358925	UP|Q3IVG1_RHOS4 (Q3IVG1) Poly (3-hydroxybutyrate) depolymerase, 5%		-0.19
6846	TC343271	UP|O23646_ARATH (O23646) RSZp22 protein, 10%		-0.20
7471	TC342920	RF|NP_177494.1|15219486|NM_106011 RNA binding {Arabidopsis thaliana}, 11%		-0.18
7969	TC331925	GB|AAP68268.1|31711824|BT008829 At5g47680 {Arabidopsis thaliana}, 14%		-0.17
8017	TC327342			-0.21
8115	TC316108	UP|Q9XGU7_ORYSA (Q9XGU7) NADP-specific isocitrate dehydrogenase		-0.18
8117	TC331376			-0.29
8247	TC336584	UP|ZRP4_MAIZE (P47917) O-methyltransferase ZRP4, 67%		0.18
8539	TC333114	Zea mays clone EL01N0526H03.c mRNA sequence		-0.21
8762	TC334154	UP|IF4A3_NICPL (P41380) Eukaryotic initiation factor 4A-3 , 47%		-0.18
8911	TC329085	RF|XP_506513.1|51963608|XM_506513 P0503D09.102 gene product {Oryza sativa (japonica cultivar-group)}, 12%		-0.18
8997	TC317193	Zea mays clone EL01N0519D06.c mRNA sequence	0.22	
9229	TC317179	UP|BP73_ORYSA (Q8L4E7) SAP-like protein BP-73 (OsBP-73) (Riaa1), 69%	0.19	
9298	TC342479	UP|Q2L3E5_BRASY (Q2L3E5) Hap5-like protein, 50%		0.20

9465	TC348244	UP|Q2U6Q3_ASPOR (Q2U6Q3) Actin regulatory protein, 3%	-0.16	
9695	TC339225			0.18
9834	TC335363			0.18
9836	TC331017	UP|ABIL1_ORYSA (Q9AXA6) Probable protein ABIL1 (Abl interactor-like protein 1), 66%	0.18	
9911	TC352340	UP|O24343_SORBI (O24343) Serine/threonine kinase, 37%	0.23	
10005	TC323358	UP|Q7QXS8_GIALA (Q7QXS8) GLP_77_45985_45458, 9%	0.22	
10008	TC350656	RF|XP_507376.1|51978998|XM_507376 OJ1699_E05.18 gene product {Oryza sativa (japonica cultivar-group)}, 91%		-0.21
10018	TC335804	Zea mays clone Contig94.F mRNA sequence		-0.18
10103	TC341385	UP|Q6PSU8_ARATH (Q6PSU8) Formin homology 2 domain-containing protein 5, 3%	0.19	
10273	TC362231	UP|Q6YUL3_ORYSA (Q6YUL3) Nodulin-like protein, 84%		-0.28
10360	TC318699	UP|Q9FR79_ORYSA (Q9FR79) Pre-mRNA splicing factor, 65%		0.18
10369	TC360238	UP|Q5NAY5_ORYSA (Q5NAY5) Ubiquitin-conjugating enzyme-like, 13%	0.17	
10373	TC367235	UP|Q9FQ60_ARATH (Q9FQ60) Peroxisome biogenesis protein PEX1, 12%		-0.19
10399	TC328643	Zea mays clone EL01N0442D06.d mRNA sequence		-0.17
10408	TC333680		0.23	
10485	TC338211		0.20	0.19
10506	TC326831	UP|Q946Y2_ORYSA (Q946Y2) Succinate dehydrogenase subunit 4, 47%	0.19	
10527	TC318672	UP|Q9FG32_ARATH (Q9FG32) Protein phosphatase 2C-like, 89%		-0.19
10665	-	-		-0.24
10666	-	-	0.25	
11056	TC328238			0.17

11240	-	-		0.15
11278	TC343031	RF|XP_507607.1|51979721|XM_507607 P0562A06.14 gene product {Oryza sativa (japonica cultivar-group)}		0.18
11662	TC339225		0.19	
11866	TC360932	RF|NP_914820.1|34906946|NM_189931 cysteine proteinase inhibitor Scb-like protein {Oryza sativa (japonica cultivar-group)}, 5%		0.19
12301	TC331078	RF|NP_178108.1|15220023|NM_106639 binding {Arabidopsis thaliana}, 45%		0.17
12389	TC355914	UP|Q697H7_9HEMI (Q697H7) ATP synthase F0 subunit 8, 21%		-0.20
12426	TC339525	UP|Q9FUL7_MAIZE (Q9FUL7) 40S ribosomal protein S24, 80%	0.19	0.16
12635	TC356396	UP|Q9FDV6_FAGSY (Q9FDV6) Protein kinase, 29%		0.22
12700	TC357450	Zea mays clone Contig135 mRNA sequence		0.19
12794	TC341011			0.17
13349	TC349798	UP|Q9EXR3_CLOBO (Q9EXR3) Spore germination protein, 5%		-0.26
13544	TC322043	UP|Q5UDB5_MAIZE (Q5UDB5) INDETERMINATE-related protein 1		0.18
13716	TC357972	UP|T2AH_DROME (Q9W5B9) Transcription initiation factor IIA gamma-2 chain (TFIIA-gamma-2), 19%		0.20
14030	TC339680	UP|Q67VJ6_ORYSA (Q67VJ6) Aintegumenta-like protein, 9%		0.23
14096	TC353719	UP|Q9AVR1_9GENT (Q9AVR1) S-adenosyl-L-methionine:salicylic acid carboxyl methyltransferase, 7%		0.18
14124	TC318182	UP|Q3UZD5_MOUSE (Q3UZD5) 8 days embryo whole body cDNA, RIKEN full-length enriched library, clone:5730516M01 product:PR-domain zinc finger protein 6 homolog, 5%	0.21	
14196	TC330238	UP|Q6CML8_KLULA (Q6CML8) Kluyveromyces lactis strain NRRL Y-1140 chromosome E of strain NRRL Y-1140 of Kluyveromyces lactis, 3%	0.25	
14819	TC353442			0.19
14899	TC345276	UP|Q5VPC7_ORYSA (Q5VPC7) CTP synthetase-like, 9%		0.19
14910	TC363159	UP|Q336Y9_ORYSA (Q336Y9) New cDNA-based Gene, 51%	-0.19	

	14985	TC348906	UP|Q2G6W9_NOVAD (Q2G6W9) Fructose-bisphosphate aldolase, 5%		0.22
	15450	TC345781	UP|Q9ZTB1_MAIZE (Q9ZTB1) Scl1 protein, 29%		-0.29
	15456	TC335782	UP|Q461D5_MAIZE (Q461D5) Stress inducible protein coi6.1, 35%		-0.33
	15544	TC334920	UP|Q9LHA8_ARATH (Q9LHA8) 70 kDa heat shock protein (AT3g12580/T2E22_110), 75%		0.18
	15550	TC331378	UP|Q9XFE4_ORYSA (Q9XFE4) Peptidyl-prolyl cis-trans isomerase, 28%	0.22	
	15562	TC316645	Zea mays clone Contig769.F mRNA sequence		0.18
	15591	TC357336	UP|Q9I4J7_PSEAE (Q9I4J7) Probable transcriptional regulator, 5%		0.17
	15617	TC349814	RF|XP_507123.1|51964678|XM_507123 P0509D04.19 gene product {Oryza sativa (japonica cultivar-group)}, 14%		0.18
	15619	TC323045	RF|XP_507539.1|51979384|XM_507539 OJ1311_D08.9 gene product {Oryza sativa (japonica cultivar-group)}, 29%		0.18
	15677	TC351932	UP|Q6YSF3_ORYSA (Q6YSF3) Anp32/Acidic nuclear phosphoprotein-like protein, 20%		0.19

Appendix S6. Transcripts with significant abundance change in *Andropogon gerardii* and *Sorghastrum nutans* in response to variation in leaf water potential (LWP) (q-value<0.05).

**Gene ontology**		**SPOTID**	**Gene Index**	**Gene annotation**	**Slope estimate**	
					A. gerardii	***S. nutans***
C1-metabolism	S-(hydroxymethyl)glutathione dehydrogenase & S-(hydroxymethyl)glutathione synthase	1703	TC330451	UP|ADHX_MAIZE (P93629) Alcohol dehydrogenase class 3		-3.94
Cell	Cycle	8429	TC337886	UP|PIN1_DIGLA (Q9LEK8) Peptidyl-prolyl cis-trans isomerase 1, 97%		3.99
	Organisation	1207	TC352355	RF|NP_196094.1|15238305|NM_120556 amino acid binding {Arabidopsis thaliana}, 46%		3.83
Development	Unspecified	4813	TC316840	UP|Q84V71_MAIZE (Q84V71) M4 protein		3.75
		7559	TC354675	UP|O82787_ORYSA (O82787) Early nodulin, 98%		5.13
DNA	Repair	12572	TC351896	UP|Q6K9U2_ORYSA (Q6K9U2) Replication protein A2, 70%	-3.69	
	Synthesis/chromatin structure.histone	8561	TC365788	UP|H2A_MAIZE (P40280) Histone H2A		3.98
		11256	TC345967	UP|Q76N07_SOLME (Q76N07) Histone H4-like protein		3.98
Glycolysis	Glyceraldehyde 3-phosphate dehydrogenase	1615	TC351862	UP|G3PE_MAIZE (Q43247) Glyceraldehyde-3-phosphate dehydrogenase, cytosolic 3, 45%		-4.56
Hormone metabolism	Auxin	9283	TC318047	GB|AAM91657.1|22136676|AY133723 auxin response factor 1 {Arabidopsis thaliana}, 49%		4.27
	Brassinosteroid	9421	TC321776	UP|Q84YE6_SORBI (Q84YE6) Cytochrome P450-like protein, 93%		4.18
Lipid metabolism	‘Exotics' (steroids, squalene etc)	6204	TC342870		4.01	

	Lipid degradation	6605	TC338703	RF|XP_506842.1|51964118|XM_506842 OSJNBb0046O12.21 gene product {Oryza sativa (japonica cultivar-group)}, 17%		4.19
Major CHO metabolism	Degradation	6081	TC362321	UP|SUS1_MAIZE (P04712) Sucrose synthase 1, 85%	4.29	
Misc	Alcohol dehydrogenases	1703	TC328105	UP|ADHX_MAIZE (P93629) Alcohol dehydrogenase class 3 , 74%		-3.94
Protein	Degradation	8430	TC320859	UP|Q945B8_EUPES (Q945B8) Growth-on protein GRO10, 88%		3.86
		9542	TC320933	UP|FTSH1_ARATH (Q39102) Cell division protein ftsH homolog 1, chloroplast precursor, 66%		3.94
		6809	TC355466	UP|Q6LCT7_MAIZE (Q6LCT7) Ubiquitin fusion protein		3.96
		6766	TC324997	UP|Q45W76_ARAHY (Q45W76) Ubiquitin-conjugating enzyme 2, 91%		3.89
		7346	TC327651	UP|Q5K4G3_ORYSA (Q5K4G3) E3 ubiquitin ligase (Ubiquitin ligase E3), 78%		4.32
		10487	TC331372	UP|Q94AD8_ARATH (Q94AD8) T10F18_70/T10F18_70 (At5g21040/T10F18_70), 42%		4.04
		5235	TC316315	UP|PSB1_ORYSA (O64464) Proteasome subunit beta type 1	4.29	
		8634	TC318099	UP|PSD6_ORYSA (Q8W425) 26S proteasome non-ATPase regulatory subunit 6		4.15
	Synthesis.initiation	82	TC332064	UP|IF2B_WHEAT (O24473) Eukaryotic translation initiation factor 2 beta subunit		-4.10
	Synthesis.misc ribososomal protein	181	TC369722	UP|RL41_ARATH (P62120) 60S ribosomal protein L41		-3.71
		490	TC366592	UP|RL18A_ORYSA (Q943F3) 60S ribosomal protein L18a	-3.56	
		1641	TC326208	UP|Q7XBH6_ORYSA (Q7XBH6) Ribosomal L9-like protein		-4.07
		1653	TC323771	UP|RS10_ORYSA (Q9AYP4) 40S ribosomal protein S10, 95%		-3.59
		3387	TC363531	UP|Q9AV77_ORYSA (Q9AV77) 60S ribosomal protein L17		4.39
		8217	TC326476	UP|Q94AF6_ARATH (Q94AF6) AT5g20160/F5O24_50 (Ribosomal protein L7Ae-like), 97%		4.44
		9210	TC338085	UP|Q7XR19_ORYSA (Q7XR19) 60S ribosomal protein L6		4.26
		10017	TC346005	UP|Q9FUL7_MAIZE (Q9FUL7) 40S ribosomal protein S24		3.98

Redox	Dismutases and catalases	1821	TC331770	GB|AAA33511.1|168622|MZESOD2A SOD2 protein {Zea mays}		-4.12
RNA	Regulation of transcription	9283	TC318047	GB|AAM91657.1|22136676|AY133723 auxin response factor 1 {Arabidopsis thaliana}, 49%		4.27
		4813	TC325266	UP|Q84V71_MAIZE (Q84V71) M4 protein		3.75
		10150	TC358883	UP|Q8LK06_MAIZE (Q8LK06) Methyl binding domain protein MBD109, 83%		4.00
		10448	TC358883	UP|Q8LK06_MAIZE (Q8LK06) Methyl binding domain protein MBD109, 83%		4.32
		10462	TC358883	UP|Q8LK06_MAIZE (Q8LK06) Methyl binding domain protein MBD109, 83%		4.43
		9202	TC332546	Zea mays clone Contig290 mRNA sequence		4.29
	RNA binding	10525	TC340045	UP|Q58T16_ARATH (Q58T16) FLK, 40%		4.12
Signaling	In sugar and nutrient physiology	8764	TC334913	UP|O82423_MAIZE (O82423) Pyruvate dehydrogenase kinase isoform 1, 47%		3.86
Stress	Abiotic.heat	636	TC318189	GB|CAB45063.1|5051770|ATT22A6 hsp 70-like protein {Arabidopsis thaliana}, 88%	-3.72	
TCA / org	Transformation	6758	TC331458	UP|Q6ZI55_ORYSA (Q6ZI55) NAD-dependent isocitrate dehydrogenase c 2, 92%		3.87
Transport	Major Intrinsic Proteins	12470	TC370281	UP|PTH2_DROME (O97067) Probable peptidyl-tRNA hydrolase 2 (PTH 2) , 22%	-3.87	
Not assigned	No ontology	1207	TC364898	UP|TBB1_MAIZE (P18025) Tubulin beta-1 chain		3.83
		6532	TC319799	GB|1604369A|226743|1604369A sulfated surface glycoprotein SSG185. {Volvox carteri}, 9%		3.69
		8240	TC362831	Zea mays clone EL01N0317G06.c mRNA sequence		3.78
		8719	TC341712			4.30
		10146	TC323380	UP|Q41719_ZEADI (Q41719) Hydroxyproline-rich glycoprotein precursor, 98%		4.21
	Unknown	6455	TC316662	UP|Q84N07_ORYSA (Q84N07) Replication factor C 38 kDa subunit		4.39
		6496	TC321853	Zea mays clone EL01N0553G07.c mRNA sequence		4.02
		6948	TC331989			4.07

		8193	TC337322	UP|Q9LW02_ARATH (Q9LW02) Arabidopsis thaliana genomic DNA, chromosome 3, P1 clone: MSJ11, 75%		3.99
		8763	TC341167	UP|Q6NLE1_ARATH (Q6NLE1) At5g58250, 39%		4.30
		9426	TC341115			5.48
No homology in Arabidopsis		726	TC361870	UP|Q5JZR1_SOYBN (Q5JZR1) N-rich protein, 20%	-3.98	
		2289	TC320965	RF|XP_506750.1|51963934|XM_506750 P0419A09.8 gene product {Oryza sativa (japonica cultivar-group)}, 47%		-4.60
		3753	-	-		3.97
		5733	-	-	4.21	
		6644	TC368618	RF|NP_179899.1|15227788|NM_127882 CYP96A1 heme binding {Arabidopsis thaliana}, 5%		4.00
		7170	TC354354	UP|Q550K7_DICDI (Q550K7) Spore coat protein sp96, 5%		4.03
		7428	TC339923	UP|Q3X570_9ACTN (Q3X570) DedA precursor, 13%		4.12
		7437	TC356642			3.92
		7502	TC334340	UP|HEXA_BLADI (Q17127) Hexamerin precursor, 7%		5.46
		7567	TC330120	UP|Q4C3T1_CROWT (Q4C3T1) ABC-1, 6%		3.83
		8342	TC339295	GB|AAA39391.1|387397|MUSKTEPI2 epidermal keratin subunit I {Mus musculus}, 5%		4.52
		10035	TC369322	UP|HD2A_MAIZE (O24591) Histone deacetylase 2a, 46%		4.44
		10379	TC321506	UP|Q93VH6_ARATH (Q93VH6) AT4g00850/A_TM018A10_22 (GRF1-interacting factor 3), 28%		3.89
		10567	TC318355	UP|Q1WD14_SHEEP (Q1WD14) Polymorphic epithelial mucin, 13%		4.07
		10753	TC356236			3.90
		11229	TC339716	UP|Q9ZRQ5_MAIZE (Q9ZRQ5) Phytase, 43%		3.82
		14302	TC330215	UP|Q552N9_DICDI (Q552N9) RNA-binding region-containing protein (RNP-1), 5%		3.83

Appendix S7. Transcripts with significant abundance change in *Andropogon gerardii* and *Sorghastrum nutans* in response to the interaction of leaf temperature (T_{leaf}) and leaf water potential (LWP) (q-value<0.05). Light grey shaded SPOTID's were transcripts changed abundance in both species.

**Gene ontology**		**SPOTID**	**Gene index**	**Gene annotation**	**Slope estimate**	
					A. gerardii	***S. nutans***
Amino acid metabolism	Degradation.glutamate family.proline	8515	TC319043	GB|AAZ91461.1|73913047|DQ154923 delta-1-pyrroline-5-carboxylate dehydrogenase {Hordeum vulgare}, 97%	0.09	
Cell wall	Cellulose synthesis	4827	TC320894	RF|NP_174697.1|15218606|NM_103160 nucleic acid binding {Arabidopsis thaliana}, 40%	-0.10	
	Degradation	6286	TC357953	UP|Q68UW0_PYRCO (Q68UW0) Beta-galactosidase, 14%	-0.09	
		10624	TC319938	Zea mays clone Contig523 mRNA sequence		-0.19
	Vesicle transport	3280	TC317682	Zea mays clone Contig617.F mRNA sequence	-0.10	
		6828	TC333972	RF|XP_506688.1|51963810|XM_506688 OJ1217_F02.19 gene product {Oryza sativa (japonica cultivar-group)}		-0.11
Cell	Cycle	8457	-	-	0.09	
	Division	4741	TC316325	UP|Q84YE5_SORBI (Q84YE5) Cyclin-dependent kinase-like protein	-0.16	
		6992	TC339630	UP|Q9FHX1_ARATH (Q9FHX1) TMV resistance protein-like, 26%		-0.18
	Organisation	2034	TC364640	UP|MNB1B_MAIZE (P27347) DNA-binding protein MNB1B	0.10	
		2980	TC333073	UP|Q9SAF1_ARATH (Q9SAF1) F3F19.20 protein (Actin-related protein 3) (At1g13180), 59%	0.09	
		3958	TC318234	UP|TBB2_MAIZE (P18026) Tubulin beta-2 chain	-0.11	
		6229	TC334040	UP|Q9FK11_ARATH (Q9FK11) Arabidopsis thaliana genomic DNA, chromosome 5, TAC clone:K19E1, 66%	-0.12	
		8900	TC362989	UP|TBA1_MAIZE (P14640) Tubulin alpha-1 chain	0.09	

Development	Unspecified	12660	TC315884	UP|Q4R0U0_MAIZE (Q4R0U0) Homeobox protein OCL1	0.11	
DNA	Synthesis/chromatin structure	479	TC352342	UP|Q93VJ8_ARATH (Q93VJ8) AT5g11200/F2I11_90 (AT5g11170/F2I11_60), 57%	0.10	
		6802	TC317817	GB|AAU89137.1|53370642|AC145388 DEAD/DEAH box helicase domain containing protein {Oryza sativa (japonica cultivar-group)}, 88%		-0.12
		7860	TC336713	UP|Q945C8_MAIZE (Q945C8) Origin recognition complex subunit 1, 13%	0.13	
		27	TC365788	UP|H2A_MAIZE (P40280) Histone H2A	0.12	
		59	TC329978	UP|H2B2_MAIZE (P30756) Histone H2B.2	0.09	
		1841	TC341191	UP|Q6LB28_LYCES (Q6LB28) Histone H3 variant H3.3		0.12
		4987	TC327808	UP|Q76MV0_TOBAC (Q76MV0) H3 histone	-0.09	
		6914	TC365788	UP|H2A_MAIZE (P40280) Histone H2A		-0.11
		7635	TC365788	UP|H2A_MAIZE (P40280) Histone H2A	0.10	
		8006	TC355183	UP|H2B2_MAIZE (P30756) Histone H2B.2		-0.10
		8048	TC316010	UP|PSD6_ORYSA (Q8W425) 26S proteasome non-ATPase regulatory subunit 6	0.10	
		8578	TC324223	UP|H2A_MAIZE (P40280) Histone H2A, 96%	0.11	
		8975	TC365603	UP|H2A2_ORYSA (Q6ZL43) Probable histone H2A.2	0.17	
		9224	TC338271	UP|Q76N07_SOLME (Q76N07) Histone H4-like protein		-0.11
		9700	TC329978	UP|H2B2_MAIZE (P30756) Histone H2B.2	0.11	
		10541	TC327070	UP|Q6LB28_LYCES (Q6LB28) Histone H3 variant H3.3	0.12	
		12018	TC363966	UP|Q76N07_SOLME (Q76N07) Histone H4-like protein	0.09	
		12079	TC333345	UP|Q76N07_SOLME (Q76N07) Histone H4-like protein	0.10	
		12401	TC365788	UP|H2A_MAIZE (P40280) Histone H2A	0.10	
		12464	TC357259	UP|H2A_MAIZE (P40280) Histone H2A	0.09	

		12515	TC329978	UP|H2B2_MAIZE (P30756) Histone H2B.2	0.12	
		13591	TC342976	UP|H2AV3_ORYSA (Q84MP7) Probable histone H2A variant 3	0.12	
Glycolysis	Aldolase	8176	TC318980	UP|ALF_MAIZE (P08440) Fructose-bisphosphate aldolase, cytoplasmic isozyme	0.12	
	Enolase	5708	TC369719	UP|ENO2_MAIZE (P42895) Enolase 2	-0.11	
	G6PIsomerase	13433	TC332925	UP|Q76E42_ORYSA (Q76E42) Glucose-6-phosphate isomerase, 29%	0.11	
	Glyceraldehyde 3-phosphate dehydrogenase	5823	TC316013	Zea mays clone Contig296 mRNA sequence	-0.11	
		9555	TC363412	UP|G3PC_MAIZE (P08735) Glyceraldehyde-3-phosphate dehydrogenase, cytosolic 1	0.10	
	PEPCK	10155	TC325108	UP|Q5EC59_MAIZE (Q5EC59) Phosphoenolpyruvate carboxylase kinase 1	0.10	
	Pyrophosphate-fructose-6-P phosphotransferase	5048	TC323414	Zea mays clone Contig720.F mRNA sequence	-0.09	
Hormone metabolism	Abscisic acid	6594	TC335035	RF|XP_506479.1|51963562|XM_506479 P0594D10.123 gene product {Oryza sativa (japonica cultivar-group)}, 91%		-0.11
	Auxin	6194	TC320382	GB|AAA33436.1|168422|MZEAUX auxin-binding protein precursor {Zea mays}	-0.11	
		8105	TC324140	UP|Q8S985_ORYSA (Q8S985) Arabidopsis ETTIN-like protein 1, 21%		-0.13
Lipid metabolism	Phospholipid synthesis	55	TC321870	UP|Q2QN70_ORYSA (Q2QN70) Diacylglycerol kinase 1, 87%	0.10	
Metal handling	Binding, chelation and storage	3389	TC357882	UP|Q5U7K6_9POAL (Q5U7K6) Metallothionein-like protein, 98%	-0.09	
Misc	Beta 1,3 glucan hydrolases	3946	TC349135	UP|Q9C5B4_ARATH (Q9C5B4) Alpha-glucosidase 1, 15%	-0.10	
	Cytochrome P450	13800	TC351588	UP|Q2QYH8_ORYSA (Q2QYH8) Cytochrome P450 monooxygenase CYP72A5, 62%	0.09	
	Gluco-, galacto- and mannosidases	3946	TC349135	UP|Q9C5B4_ARATH (Q9C5B4) Alpha-glucosidase 1, 15%	-0.10	
		6286	TC357953	UP|Q68UW0_PYRCO (Q68UW0) Beta-galactosidase, 14%	-0.09	

		10624	TC319938	Zea mays clone Contig523 mRNA sequence		-0.19
	Myrosinases-lectin-jacalin	5713	TC318654	UP|Q9LL87_MAIZE (Q9LL87) Beta-glucosidase aggregating factor, 13%	-0.11	
		8175	TC318654	UP|Q9LL87_MAIZE (Q9LL87) Beta-glucosidase aggregating factor, 13%		-0.12
	Protease inhibitor/seed storage/lipid transfer protein (LTP) family protein	1999	TC333824	UP|O24556_MAIZE (O24556) Physical impedance induced protein	-0.11	
	Short chain dehydrogenase/reductase (SDR)	5826	TC326786	UP|Q7QJE4_ANOGA (Q7QJE4) ENSANGP00000019038, 64%	-0.15	
Mitochondrial electron transport / ATP synthesis	Cytochrome c oxidase	12775	TC320634	UP|Q9SXV0_ORYSA (Q9SXV0) Cytochrome c oxidase subunit 6b-1 (Cytochrome c oxidase subunit 6b), 77%	0.11	
Nucleotide metabolism	.degradation	6741	TC323967	RF|XP_506591.1|51963676|XM_506591 P0034A04.129 gene product {Oryza sativa (japonica cultivar-group)}, 81%		-0.11
	Phosphotransfer and pyrophosphatases.misc	418	TC316547	UP|Q6YVH9_ORYSA (Q6YVH9) Inorganic pyrophosphatase, 92%	0.10	
		7378	TC332430	UP|NDK1_SACOF (P93554) Nucleoside diphosphate kinase 1	0.09	
		10143	TC332430	UP|NDK1_SACOF (P93554) Nucleoside diphosphate kinase 1	0.12	
		11238	TC316877	UP|Q9SP13_PEA (Q9SP13) Nucleoside diphosphate kinase, 76%	0.12	
		2000	TC316766	UP|Q9SE48_ORYSA (Q9SE48) UMP/CMP kinase a, 95%	-0.11	
Protein	Amino acid activation	5917	TC318593	UP|O82108_MAIZE (O82108) Seryl-tRNA synthetase	-0.15	
		1049	TC319527	UP|Q8LPC9_ORYSA (Q8LPC9) Threonyl-tRNA synthetase	0.10	
	Degradation	8648	TC317033	Zea mays clone EL01N0509G01.c mRNA sequence	0.12	
		9162	TC316720	UP|Q8SBA4_ORYSA (Q8SBA4) Autophagocytosis protein AUT1-like, 94%	0.09	
		5647	TC344876	UP|Q655R7_ORYSA (Q655R7) OTU-like cysteine protease-like, 80%	-0.10	

	3846	TC332198	GB|AAP49525.1|31376397|BT008763 At1g28110 {Arabidopsis thaliana}, 61%	-0.11	
	7714	TC322656	UP|O48555_MAIZE (O48555) Ubiquitin conjugating enzyme	0.10	
	11419	TC364608	UP|Q5XUV4_WHEAT (Q5XUV4) Ubiquitin-conjugating enzyme	0.11	
	12039	TC343341	UP|O48555_MAIZE (O48555) Ubiquitin conjugating enzyme		-0.11
	45	TC318087	UP|Q6IFZ5_RAT (Q6IFZ5) Type II keratin Kb9, 3%		0.11
	3390	TC327047	RF|NP_171642.1|15223384|NM_100017 nucleic acid binding {Arabidopsis thaliana}, 33%	-0.10	
	10001	TC323093	GB|AAH25374.1|19263501|BC025374 ring finger protein 126, isoform 2 {Homo sapiens}, 19%	0.09	
	10101	-	-	0.12	
	10100	TC370145	UP|Q6H678_ORYSA (Q6H678) F-box protein-like, 41%		-0.11
	8094	TC322609	UP|Q7Q9J7_ANOGA (Q7Q9J7) ENSANGP00000010437, 91%	0.15	
Folding	149	TC316727	UP|Q53NM9_ORYSA (Q53NM9) DnaK-type molecular chaperone hsp70-rice		0.09
Postranslational modification	10	TC323065	UP|Q8S7U0_ORYSA (Q8S7U0) Serine/threonine protein phosphatase PP2A-4 catalytic subunit, 90%	0.17	
	11	TC323949	Zea mays clone Contig73 mRNA sequence	0.09	
	3670	-	-	-0.11	
	5947	TC333291	RF|NP_174401.1|15221629|NM_102854 protein kinase C binding {Arabidopsis thaliana}, 70%	-0.14	
	8806	TC339077	UP|Q5Z7K2_ORYSA (Q5Z7K2) Serine/threonine protein phosphatase PP2A-1 catalytic subunit		-0.11
	12096	TC318284	UP|Q8LSN3_PEA (Q8LSN3) Serine/threonine protein phosphatase 2A	0.10	
Synthesis.elongation	6194	TC336843	UP|Q9FYV3_SACOF (Q9FYV3) Elongation factor	-0.11	
	8066	TC358596	UP|O50018_MAIZE (O50018) Elongation factor 1-alpha	0.12	
	11241	TC365231	Zea mays clone Contig598 mRNA sequence		-0.12
	11381	TC369772	Zea mays clone Contig257 mRNA sequence	0.12	

	11420	TC346618	Zea mays clone Contig308 mRNA sequence		-0.11
Synthesis.initiation	10918	TC333592	UP|SUI1_ORYSA (P33278) Protein translation factor SUI1 homolog	0.11	
	11085	TC334370	UP|SUI1_MAIZE (P56330) Protein translation factor SUI1 homolog	0.09	
	12102	TC346232	Zea mays clone Contig468 mRNA sequence	0.13	
	15519	TC316808	UP|Q3ECQ8_ARATH (Q3ECQ8) Protein At1g53880 (Protein At1g53900), 57%	0.15	
Synthesis.misc ribososomal protein	9	TC330969	UP|RS11_MAIZE (P25460) 40S ribosomal protein S11	0.16	
	32	TC346170	Zea mays clone Contig334 mRNA sequence	0.10	
	38	TC324399	UP|Q9FJA6_ARATH (Q9FJA6) 40S ribosomal protein S3 (AT5g35530/MOK9_14), 88%	0.14	
	65	TC364357	Zea mays clone Contig30.F mRNA sequence	0.09	
	843	TC321886	UP|Q9FSF6_TOBAC (Q9FSF6) Ribosomal protein L11-like, 97%	0.09	
	856	TC324341	GB|BAA08264.1|1321661|D45423 ascorbate peroxidase {Oryza sativa}	0.10	
	1219	TC343216	UP|RL39_MAIZE (P51425) 60S ribosomal protein L39	-0.09	
	3761	TC334274	UP|RS4_MAIZE (O22424) 40S ribosomal protein S4	-0.13	
	3800	TC344372	RF|XP_507427.1|51979104|XM_507427 P0523B07.46 gene product {Oryza sativa (japonica cultivar-group)}	-0.09	
	5143	TC317738	UP|Q948T0_WHEAT (Q948T0) Mitochondrial ribosomal protein L11	-0.13	
	5546	TC330862	RF|XP_507356.1|51978958|XM_507356 OJ1014_E09.28 gene product {Oryza sativa (japonica cultivar-group)}, 97%	-0.09	
	7651	-	-	-0.13	
	8824	TC343035	UP|RL17_MAIZE (O48557) 60S ribosomal protein L17	0.10	
	8886	TC333590	UP|Q3MST7_ORYSA (Q3MST7) Ribosomal L32	0.10	
	8903	TC337033	UP|Q5WMY3_ORYSA (Q5WMY3) Cytoplasmic ribosomal protein L18	0.11	

		9165	TC363169	UP|Q7XY20_WHEAT (Q7XY20) Ribosomal protein L19, 89%	0.10	
		9773	TC334532	UP|Q7XC31_ORYSA (Q7XC31) 60S ribosomal protein L27	0.11	
		9857	TC346170	Zea mays clone Contig334 mRNA sequence	0.13	
		10189	TC324855	UP|Q7XR19_ORYSA (Q7XR19) 60S ribosomal protein L6	0.10	
		10503	TC340118	UP|RLA1_MAIZE (P52855) 60S acidic ribosomal protein P1 (L12)	0.09	
		10773	TC329124	UP|RL24_HORVU (P50888) 60S ribosomal protein L24	0.09	
		10971	TC324387	UP|Q7XC31_ORYSA (Q7XC31) 60S ribosomal protein L27	0.14	
		11973	TC371087	UP|Q5GMM4_CAPCH (Q5GMM4) 60S ribosomal protein L37a	0.11	
		12189	TC367575	UP|Q9AVU0_PICAB (Q9AVU0) 40S ribosomal protein S2, 78%	0.11	
		12408	TC331279	RF|XP_506724.1|51963882|XM_506724 OJ9003_G05.34 gene product {Oryza sativa (japonica cultivar-group)}	0.09	
		12416	TC348875	UP|Q6K853_ORYSA (Q6K853) 40S ribosomal protein S30-like	0.12	
		12434	TC325255	UP|Q2R1J8_ORYSA (Q2R1J8) Ribosomal protein S4, 98%	0.10	
		12477	TC325572	UP|RS141_MAIZE (P19950) 40S ribosomal protein S14 (Clone MCH1)	0.10	
		12617	TC358005	UP|RS11_MAIZE (P25460) 40S ribosomal protein S11	0.10	
	Targeting	10257	TC344686	UP|Q32Y71_ORYSA (Q32Y71) Peroxisomal targeting signal 1 receptor long form, 48%	0.11	
		6821	TC327724	RF|XP_507057.2|51978887|XM_507057 P0474F11.16-1 gene product {Oryza sativa (japonica cultivar-group)}, 94%	-0.10	
		8521	TC318641	UP|Q8W403_ORYSA (Q8W403) Sec13p	0.09	
		31	TC326586	UP|Q6UCJ1_CUCSA (Q6UCJ1) Signal recognition particle receptor protein, 66%	0.14	
		48	TC362978	UP|Q7XZG0_ORYSA (Q7XZG0) ADP-ribosylation factor		0.11
		8465	TC324392	UP|Q2R135_ORYSA (Q2R135) Expressed protein, 97%	0.10	

Photosynthesis	Calvin cyle	10901	TC343644	UP|PGKH_WHEAT (P12782) Phosphoglycerate kinase, chloroplast precursor, 33%	0.10	
Redox	Ascorbate and glutathione	4691	TC324531	UP|Q84KI3_SORBI (Q84KI3) Cytochrome b5	-0.12	
		13174	TC316473	UP|Q6I681_MAIZE (Q6I681) Cytochrome b561	-0.13	
	Dismutases and catalases	416	TC325282	UP|Q94L33_ARATH (Q94L33) Ania-6a type cyclin, 15%	0.11	
	Thioredoxin	6107	TC351539	UP|Q8LCH9_ARATH (Q8LCH9) Thioredoxin-like protein, 72%	-0.12	
RNA	Processing	10172	TC357187	UP|Q84LL7_BETVU (Q84LL7) Salt tolerance protein 6, 67%	0.09	
		903	TC344697	RF|XP_507274.1|51964980|XM_507274 P0481F05.17 gene product {Oryza sativa (japonica cultivar-group)}, 51%	0.09	
		3330	TC339403	UP|Q69K06_ORYSA (Q69K06) Pre-mRNA splicing factor PRP38 protein-like, 97%	-0.10	
		4496	TC316321	Zea mays clone Contig631.F mRNA sequence	0.09	
		10431	TC354282	UP|Q2QKC2_WHEAT (Q2QKC2) Pre-mRNA processing factor, 96%	0.13	
		12785	TC325146	UP|Q2QKC2_WHEAT (Q2QKC2) Pre-mRNA processing factor, 97%	0.11	
	Regulation of transcription	8780	TC322691	UP|Q6ZD92_ORYSA (Q6ZD92) Proline-rich protein-like, 48%	0.10	
		9717	TC327557	Zea mays clone EL01N0450G11.d mRNA sequence	0.10	
		8105	TC334681	UP|Q8S985_ORYSA (Q8S985) Arabidopsis ETTIN-like protein 1, 48%		-0.13
		5828	TC338589	UP|Q8S490_ORYSA (Q8S490) Transcription factor RAU1, 71%	-0.11	
		10436	TC348661	UP|Q5JK17_ORYSA (Q5JK17) Transcription factor ICE1-like, 63%	0.11	
		6619	TC318347	UP|YABDL_ORYSA (Q76EJ0) Protein DROOPING LEAF, 92%		-0.12
		5652	TC359677	UP|Q5Z9H7_ORYSA (Q5Z9H7) Zinc finger protein-like, 89%	-0.09	
		4944	TC331266	UP|Q8W0W6_MAIZE (Q8W0W6) Repressor protein, 78%	-0.10	
		4353	TC360477	UP|Q5BXG0_SCHJA (Q5BXG0) SJCHGC03664 protein, 14%	-0.11	

		12512	TC345060	Zea mays clone Contig141 mRNA sequence	0.10	
		12660	TC315884	UP|Q4R0U0_MAIZE (Q4R0U0) Homeobox protein OCL1	0.11	
		10099	TC364640	UP|MNB1B_MAIZE (P27347) DNA-binding protein MNB1B	0.10	
		12423	TC364640	UP|MNB1B_MAIZE (P27347) DNA-binding protein MNB1B	0.08	
		71	TC342665	UP|Q6ZLD8_ORYSA (Q6ZLD8) Fiber protein-like, 48%	0.10	
		4629	TC357075	UP|Q9M7F3_MAIZE (Q9M7F3) LIM transcription factor homolog	-0.10	
		7403	TC354762	UP|Q9M7F3_MAIZE (Q9M7F3) LIM transcription factor homolog, 52%	-0.12	
		8220	TC345401			-0.12
		12512	TC345060	Zea mays clone Contig141 mRNA sequence	0.10	
		56	TC322645	RF|NP_564993.1|18409643|NM_105733 binding {Arabidopsis thaliana}, 28%	0.10	
		5739	-	-		-0.11
		6992	TC339630	UP|Q9FHX1_ARATH (Q9FHX1) TMV resistance protein-like, 26%		-0.18
		7860	TC336713	UP|Q945C8_MAIZE (Q945C8) Origin recognition complex subunit 1, 13%	0.13	
		10172	TC316815	UP|Q9LEB4_NICPL (Q9LEB4) RNA Binding Protein 45, 76%	0.09	
		12039	TC320216	GB|AAP37853.1|30725662|BT008494 At1g11650 {Arabidopsis thaliana}, 45%		-0.11
	RNA binding	56	TC321032	UP|Q2PCD1_WHEAT (Q2PCD1) Type 1 non specific lipid transfer protein precursor, 95%	0.10	
	Transcription	5439	TC319720	UP|Q4ABR3_BRARP (Q4ABR3) 80A08_4, 39%	-0.14	
		7857	TC325072	RF|NP_175827.1|15221759|NM_104303 ATRPABC16.5 DNA-directed RNA polymerase {Arabidopsis thaliana}, 91%	0.11	
Signaling	14-3-3 proteins	2903	TC367395	UP|14331_MAIZE (P49106) 14-3-3-like protein GF14-6	0.09	
	Calcium	13	TC324214	UP|Q7DLR7_MAIZE (Q7DLR7) Calmodulin	0.10	
		7007	TC368414	RF|NP_180187.1|15225258|NM_128176 calmodulin binding {Arabidopsis thaliana}, 48%		-0.11

	Light	16	TC337635	GB|AAT85270.1|50881425|AC133335 expressed protein {Oryza sativa (japonica cultivar-group)}, 96%	0.14	
	MAP kinases	3950	TC316110	UP|O49975_MAIZE (O49975) Protein kinase ZmMEK1	-0.11	
		5516	TC324244	UP|Q9MB22_ARATH (Q9MB22) ATMPK9, 43%	-0.19	
	Receptor kinases	719	TC350209	RF|NP_174673.2|30692999|NM_103134 ATP binding {Arabidopsis thaliana}, 8%	0.10	
Stress	Abiotic.drought/salt	10180	TC336873	GB|ABB47874.1|78708899|AE016959 expressed protein {Oryza sativa (japonica cultivar-group)}, 14%	0.11	
	Abiotic.heat	149	TC316727	UP|Q53NM9_ORYSA (Q53NM9) DnaK-type molecular chaperone hsp70-rice		0.09
		171	TC347913	RF|NP_680194.1|22326960|NM_147889 heat shock protein binding {Arabidopsis thaliana}, 47%	0.10	
		4796	TC328048	RF|XP_506783.1|51964000|XM_506783 P0543C11.34 gene product {Oryza sativa (japonica cultivar-group)}	-0.10	
		5802	TC327823	UP|Q7F1J8_ORYSA (Q7F1J8) DnaJ protein family-like, 88%	-0.13	
		6311	TC368947	UP|Q38HT9_SOLTU (Q38HT9) DnaJ-like protein, 44%	-0.17	
	Abiotic.unspecified	10759	TC325848	UP|Q8S3R7_ORYSA (Q8S3R7) Ethylene-responsive protein-like	0.09	
		13697	TC342426	UP|Q6TM44_MAIZE (Q6TM44) Germin-like protein, 66%	0.09	
	Biotic	13402	TC339098	RF|NP_195056.2|30689664|NM_119484 ADR1-L1 ATP binding {Arabidopsis thaliana}, 32%	0.12	
TCA / org	Transformation	13397	TC356756	UP|Q9SIB9_ARATH (Q9SIB9) Cytoplasmic aconitate hydratase, 25%	0.10	
		2857	-	-	-0.10	
Transport	Major Intrinsic Proteins	15022	TC316339	UP|Q9AQU5_MAIZE (Q9AQU5) Plasma membrane integral protein ZmPIP1-4	0.10	
	Metabolite transporters at the mitochondrial membrane	10010	TC361986	UP|ADT1_MAIZE (P04709) ADP,ATP carrier protein 1, mitochondrial precursor	0.10	
	Metal	2334	TC346872	UP|Q2R041_ORYSA (Q2R041) Magnesium/proton exchanger AtMHX, 41%	-0.12	

		13437	TC337431	UP|Q7XI43_ORYSA (Q7XI43) Metal-transporting P-type ATPase-like protein, 48%	0.13	
	P- and v-ATPases	9256	TC353176	UP|Q945E8_PENAM (Q945E8) Vacuolar H+-ATPase 16 kDa proteolipid subunit c	0.10	
	Sugars	1608	TC316506	UP|Q6PST5_MAIZE (Q6PST5) Sucrose transporter 2	-0.09	
Not assigned	No ontology	438	TC316482		0.09	
		3922	TC346257	UP|Q75KV5_ORYSA (Q75KV5) Expressed protein, 30%	-0.11	
		3978	TC322802	UP|Q94C36_ARATH (Q94C36) At3g13062, 39%	-0.12	
		6802	TC332211	UP|Q3EBI6_ARATH (Q3EBI6) Protein At2g40935, 61%		-0.12
		7552	TC319031	UP|Q9LR62_ARATH (Q9LR62) F21B7.23, 72%		-0.15
		8104	TC336183	UP|Q9MAL9_ARATH (Q9MAL9) T25K16.15 (At1g01160/F6F3_1) (GRF1-interacting factor 2), 37%		-0.11
		9260	TC318488	UP|Q7XI46_ORYSA (Q7XI46) Hydrolase-like protein, 72%	0.11	
		9854	TC362975	UP|Q8H3G8_ORYSA (Q8H3G8) Myosin heavy chain-like protein, 6%	0.12	
		10239	TC317205	Zea mays clone EL01N0426H04.c mRNA sequence		-0.12
		12648	-	-	0.11	
		13378	TC327182	UP|Q2EN88_MAIZE (Q2EN88) Guanylyl cyclase-like protein, 43%	0.10	
		13436	TC323254	RF|NP_850385.1|30689259|NM_180054 calmodulin binding {Arabidopsis thaliana}, 72%	0.11	
		14	TC365456	UP|Q9SDN6_TOBAC (Q9SDN6) FH protein NFH1, 12%	0.12	
		10528	TC323380	UP|Q41719_ZEADI (Q41719) Hydroxyproline-rich glycoprotein precursor, 98%		-0.13
		13430	TC358875	UP|Q40705_ORYSA (Q40705) Osr40c1 protein, 41%	-0.13	-0.23
	Unknown	28	TC334537		0.11	
		30	TC339858	UP|Q9LK32_ARATH (Q9LK32) Gb|AAF03438.1 (AT3g27210/K17E12_3), 16%	0.14	
		1674	TC332810	UP|Q27A62_MYCFV (Q27A62) Peptidase M24, 4%		0.11

		1975	TC360005		-0.09	
		3346	TC316925	Zea mays clone Contig451.F mRNA sequence	-0.11	
		3947	TC344292	UP|Q6CHT8_YARLI (Q6CHT8) Yarrowia lipolytica chromosome A of strain CLIB122 of Yarrowia lipolytica, 17%	-0.15	
		4705	TC339831		-0.13	
		5146	TC327522		-0.12	
		6455	TC316662	UP|Q84N07_ORYSA (Q84N07) Replication factor C 38 kDa subunit	-0.11	
		7157	TC340400	GB|AAL16180.1|16226487|AF428412 At3g07760/F17A17.10 {Arabidopsis thaliana}, 94%	-0.14	
		8501	TC342440	UP|YB1E_SCHPO (P87179) Serine-rich protein C30B4.01c precursor, 7%	0.12	
		8549	TC322706		0.09	
		8844	TC337322	UP|Q9LW02_ARATH (Q9LW02) Arabidopsis thaliana genomic DNA, chromosome 3, P1 clone: MSJ11, 75%	0.11	
		8969	TC347581	GB|BAD43484.1|51969584|AK175721 predicted GPI-anchored protein {Arabidopsis thaliana}, 26%	0.09	
		8983	TC329841		0.10	
		9385	TC337322	UP|Q9LW02_ARATH (Q9LW02) Arabidopsis thaliana genomic DNA, chromosome 3, P1 clone: MSJ11, 75%	0.10	
		9426	TC341115		-0.11	
		9840	TC332253	UP|Q8RY12_ARATH (Q8RY12) AT5g20600/F7C8_190, 9%	0.10	
		10096	TC344393	UP|Q8IQY7_DROME (Q8IQY7) CG32552-PA, 9%	0.09	
		11768	TC324604	Zea mays clone Contig497.F mRNA sequence	0.09	
		11775	TC331134	UP|Q2IMJ3_ANADE (Q2IMJ3) LigA, 6%	0.11	
		11969	TC345911	UP|Q75H93_ORYSA (Q75H93) Expressed protein, 90%	0.13	
		15602	TC317500	UP|Q3S4H5_MAIZE (Q3S4H5) Dual-specificity protein-like phosphatase 3	0.09	

No homology in Arabidopsis	256	TC332825	RF|XP_507494.1|51979265|XM_507494 OJ1476_F05.20 gene product {Oryza sativa (japonica cultivar-group)}, 68%	0.09	
	260	TC368894		0.10	
	313	TC346912	UP|O01860_CAEEL (O01860) Collagen protein 55, 9%	0.11	
	1168	TC364327	UP|Q2V2X8_ARATH (Q2V2X8) Protein At5g57000, 16%	-0.10	
	1859	TC343192	UP|Q5U8L4_GOSHI (Q5U8L4) SBP transcription factor, 50%	0.09	
	2793	TC349563			-0.18
	3371	TC325899	GB|AAB23484.1|256638|S45168 15 kda organ-specific salt-induced protein Method: conceptual translation with partial peptide sequencing {Oryza sativa}, 10%	-0.09	
	3372	TC348792	Zea mays clone EL01N0519B08.d mRNA sequence	-0.12	
	3507	TC341027		-0.09	
	3753	-	-	-0.10	
	3858	TC343616	UP|Q67W96_ORYSA (Q67W96) Kelch repeat containing F-box protein-like, 19%	0.10	
	3861	TC336124	UP|Q84JQ1_HORVD (Q84JQ1) Phosphate transporter HvPT4, 4%	-0.10	
	3980	TC343291		-0.11	
	4239	TC349467	UP|O18515_HYDAT (O18515) Nucleoporin p62, 5%	-0.11	
	4354	TC339242	UP|Q8S566_ORYSA (Q8S566) Guanine nucleotide-exchange protein GEP1, 5%	-0.12	
	4688	TC319764	UP|Q3ANJ1_SYNSC (Q3ANJ1) DNA-directed DNA polymerase, 6%	-0.10	
	4689	TC324011	UP|Q84U00_GOSHI (Q84U00) Ser-thr protein kinase, 35%	-0.13	
	4757	TC340406	UP|Q2QLY7_ORYSA (Q2QLY7) Expressed protein, 40%	-0.14	
	4907	TC357883		-0.11	
	5177	TC360627		-0.11	

5199	TC336887	Zea mays clone Contig189 mRNA sequence	-0.09	
5359	TC321156	UP|Q69WH1_ORYSA (Q69WH1) RNA-binding protein-like, 70%	-0.11	
5458	-	-	0.12	
5960	TC345844	UP|Q8SA85_MAIZE (Q8SA85) Prpol, 12%	-0.13	
6164	TC341226	Zea mays clone cho1c.pk003.k1, mRNA sequence	-0.10	
6225	TC341818	UP|Q8H349_ORYSA (Q8H349) VP1/ABI3 family regulatory protein-like protein, 25%	-0.12	
6335	TC357421		-0.10	
6688	TC352800	UP|Q4S436_TETNG (Q4S436) Chromosome 20 SCAF14744, whole genome shotgun sequence, 5%	0.09	
7030	TC333381	RF|NP_195678.1|15235982|NM_120129 DNA binding {Arabidopsis thaliana}, 7%	-0.12	
7096	TC341847			-0.16
7284	TC337513	UP|Q2QNR7_ORYSA (Q2QNR7) CTP synthase, 4%	-0.12	
7428	TC339923	UP|Q3X570_9ACTN (Q3X570) DedA precursor, 13%	-0.16	
7435	TC342168	UP|Q867T7_DICDI (Q867T7) P67-like superoxide-generating NADPH oxidase, 4%		-0.12
7600	TC347817			-0.12
7891	TC338415	UP|Q8VYU6_ARATH (Q8VYU6) At2g46180/T3F17.17, 14%	-0.11	
8114	TC364640	UP|MNB1B_MAIZE (P27347) DNA-binding protein MNB1B		-0.10
8255	TC334552	UP|Q209B2_MYXXA (Q209B2) Phosphoglucoisomerase, 7%	0.12	
8332	TC357066	UP|Q8GU81_ORYSA (Q8GU81) MDR-like ABC transporter (MDR-like p-glycoprotein-like), 6%		-0.16
8558	TC325434			-0.11
8639	TC343064			-0.10
8641	-	-	0.11	

8747	TC336887	Zea mays clone Contig189 mRNA sequence	0.12	
8752	TC365366			-0.12
8837	TC318428	Zea mays clone EL01N0360D09.c mRNA sequence	0.10	
9154	TC332719	UP|PRP39_SCHPO (O74970) Pre-mRNA-processing protein prp39, 3%	-0.12	
9565	TC360292		0.10	
9747	TC327342		0.09	
9830	TC361245	UP|Q5FIE0_LACAC (Q5FIE0) Transposase, 5%	0.16	
10281	TC363555	UP|Q9LNA7_ARATH (Q9LNA7) F5O11.11, 22%	0.10	
10408	TC333680			-0.11
10874	TC341226	Zea mays clone cho1c.pk003.k1, mRNA sequence	0.09	
10973	TC334996	UP|Q2IMJ3_ANADE (Q2IMJ3) LigA, 5%	0.09	
11604	TC318240	Zea mays clone EL01N0407E11.d mRNA sequence	0.10	
11869	TC335726	UP|Q285R4_HORVD (Q285R4) ABA 8'-hydroxylase 2, 11%	0.11	
11970	TC353607	UP|Q4ABM1_BRARP (Q4ABM1) 80C09_15, 28%	0.09	
12122	TC324474	UP|MAD32_ORYSA (Q8S151) MADS-box transcription factor 32 (OsMADS32), 88%	0.09	
12373	TC363625		0.12	
12694	TC357114	UP|Q88TX4_LACPL (Q88TX4) Phosphoglycerate mutase, 6%	0.09	
12771	TC330074	UP|Q93ZM0_ARATH (Q93ZM0) AT3g18370/MYF24_8, 12%	0.09	
12794	TC341011		0.14	
12797	TC349111	RF|XP_507406.1|51979058|XM_507406 P0450A04.131 gene product {Oryza sativa (japonica cultivar-group)}, 12%	0.16	
13041	TC365874			-0.12

	13407	TC320322	UP|TKTC_MAIZE (Q7SIC9) Transketolase, chloroplast (TK) , 60%		-0.12
	13429	TC343148		0.11	
	13495	TC319203	Zea mays clone Contig58 mRNA sequence	0.14	
	13586	TC354792	UP|P93443_ORYSA (P93443) Photosystem II 10 kDa polypeptide, 21%		-0.12
	14151	TC364844		0.10	
	14213	TC316406	Zea mays clone EL01N0515E02.c mRNA sequence	0.09	
	14843	TC345974	UP|Q2R3W9_ORYSA (Q2R3W9) Abscisic acid-induced-like protein, 91%	0.11	

Appendix S8. Transcripts with significant abundance change in *Andropogon gerardii* and *Sorghastrum nutans* in response to sampling date (q-value<0.05). Light grey shaded spotID's are transcripts with changed abundance in both species.

**Gene ontology**		**SPOTID**	**Gene index**	**Gene annotation**	**Slope estimate**	
					A. gerardii	***S. nutans***
Amino acid metabolism	Degradation	2268	TC327209	UP|Q9LK08_ARATH (Q9LK08) 3-hydroxyisobutyryl-coenzyme A hydrolase-like protein, 77%	0.02	
		2899	TC353305	UP|Q56XU5_ARATH (Q56XU5) 3-hydroxyisobutyryl-coenzyme A hydrolase (At1g06550), 87%	-0.01	
		6870	TC338688	UP|O04469_ARATH (O04469) F5I14.5 protein (At1g65520), 58%		0.01
		10712	TC339190	UP|Q9LKJ1_ARATH (Q9LKJ1) CoA-thioester hydrolase CHY1 (3-hydroxyisobutyryl-coenzyme A hydrolase), 70%		0.01
		4247	TC368889	UP|Q45W70_ARAHY (Q45W70) Isomerase-like protein, 65%		0.01
		5064	TC346718	UP|Q45W70_ARAHY (Q45W70) Isomerase-like protein, 56%		-0.02
		14547	TC361093	Zea mays clone EL01N0531D09.c mRNA sequence	0.01	
		8290	-	-		-0.01
		2268	TC327209	UP|Q9LK08_ARATH (Q9LK08) 3-hydroxyisobutyryl-coenzyme A hydrolase-like protein, 77%	0.02	
		2899	TC353305	UP|Q56XU5_ARATH (Q56XU5) 3-hydroxyisobutyryl-coenzyme A hydrolase (At1g06550), 87%	-0.01	
		6870	TC338688	UP|O04469_ARATH (O04469) F5I14.5 protein (At1g65520), 58%		0.01
		10712	TC339190	UP|Q9LKJ1_ARATH (Q9LKJ1) CoA-thioester hydrolase CHY1 (3-hydroxyisobutyryl-coenzyme A hydrolase), 70%		0.01
		2444	TC367225	UP|Q84VE1_ORYSA (Q84VE1) Adenosylhomocysteinase-like protein	-0.02	
		11745	TC367225	UP|Q84VE1_ORYSA (Q84VE1) Adenosylhomocysteinase-like protein		-0.01
		12516	TC367225	UP|Q84VE1_ORYSA (Q84VE1) Adenosylhomocysteinase-like protein		-0.01
		2583	TC316141	UP|Q6XC06_MAIZE (Q6XC06) Glyoxalase I	-0.01	0.01
		4427	TC337595	UP|LGUL_CICAR (O49818) Lactoylglutathione lyase, 90%	-0.01	

	7653	TC355817	UP|Q5I7K2_WHEAT (Q5I7K2) Ribosomal protein S7		-0.01
	13873	TC316141	UP|Q6XC06_MAIZE (Q6XC06) Glyoxalase I		-0.01
	7814	TC347710	UP|Q2RAU5_ORYSA (Q2RAU5) Hydroxymethylglutaryl-coa lyase, 37%		0.01
	11474	TC325321	UP|MCCB_ARATH (Q9LDD8) Methylcrotonoyl-CoA carboxylase beta chain, mitochondrial precursor, 44%	0.01	
	2268	TC327209	UP|Q9LK08_ARATH (Q9LK08) 3-hydroxyisobutyryl-coenzyme A hydrolase-like protein, 77%	0.02	
	6870	TC338688	UP|O04469_ARATH (O04469) F5I14.5 protein (At1g65520), 58%		0.01
	10712	TC339190	UP|Q9LKJ1_ARATH (Q9LKJ1) CoA-thioester hydrolase CHY1 (3-hydroxyisobutyryl-coenzyme A hydrolase), 70%		0.01
	5022	TC348477	UP|ARGI1_ARATH (P46637) Arginase, 54%		0.01
	4844	TC355203	UP|Q94F76_MAIZE (Q94F76) Silencing group B protein	-0.01	
	1782	TC343761	RF|NP_566838.1|18405808|NM_113768 oxidoreductase, acting on paired donors, with incorporation or reduction of molecular oxygen, 2-oxoglutarate as one donor, and incorporation of one atom each of oxygen into both donors {Arabidopsis thaliana}, 78%	-0.02	
	6006	TC319043	GB|AAZ91461.1|73913047|DQ154923 delta-1-pyrroline-5-carboxylate dehydrogenase {Hordeum vulgare}, 97%	-0.01	
	8058	TC343761	RF|NP_566838.1|18405808|NM_113768 oxidoreductase, acting on paired donors, with incorporation or reduction of molecular oxygen, 2-oxoglutarate as one donor, and incorporation of one atom each of oxygen into both donors {Arabidopsis thaliana}, 78%		-0.02
	14909	TC343761	RF|NP_566838.1|18405808|NM_113768 oxidoreductase, acting on paired donors, with incorporation or reduction of molecular oxygen, 2-oxoglutarate as one donor, and incorporation of one atom each of oxygen into both donors {Arabidopsis thaliana}, 78%	0.01	
	6349	TC325870	UP|Q9ZPK0_DATGL (Q9ZPK0) Thiosulfate sulfurtransferase, 79%		0.01
	3112	TC316310	UP|Q6V9T1_ORYSA (Q6V9T1) Glycine dehydrogenase P protein, 56%		0.01
	13565	TC319428	UP|GCST_FLAPR (P49363) Aminomethyltransferase, mitochondrial precursor, 93%		-0.02
	4425	TC336699	UP|Q8GUG5_ARATH (Q8GUG5) Threonine dehydratase/deaminase (OMR1), 57%	-0.01	0.01

Synthesis	10736	TC367699	UP|O24566_MAIZE (O24566) 3-phosphoshikimate 1-carboxyvinyltransferase		0.01
	13675	TC332434	RF|NP_187286.1|15230703|NM_111510 EMB3004 3-dehydroquinate dehydratase/ shikimate 5-dehydrogenase {Arabidopsis thaliana}, 70%		0.01
	3326	TC316746	UP|Q5NTH4_ORYSA (Q5NTH4) Shikimate kinase 1, 95%		-0.01
	13366	TC354828	UP|Q9XF60_ARATH (Q9XF60) Chorismate mutase 3, 17%		0.01
	6331	TC334806	GB|BAD11024.1|42543941|AB116722 anthranilate synthase beta 2 subunit {Oryza sativa (japonica cultivar-group)}, 67%		0.01
	5417	TC327456	UP|Q8W0Q7_SORBI (Q8W0Q7) Methionine synthase protein		0.01
	109	TC323570	UP|O04981_MAIZE (O04981) Cystathionine gamma-synthase		0.01
	3335	TC316943	UP|Q8W0Q7_SORBI (Q8W0Q7) Methionine synthase protein, 61%		0.01
	6272	TC332230	UP|METK_ORYSA (P46611) S-adenosylmethionine synthetase 1, 82%		0.01
	7103	TC365565	UP|METK_ORYSA (P46611) S-adenosylmethionine synthetase 1	0.01	
	14378	TC320463	UP|AKH2_MAIZE (P49080) Bifunctional aspartokinase/homoserine dehydrogenase 2, chloroplast precursor [Includes: Aspartokinase Homoserine dehydrogenase] , 23%	0.01	
	6745	TC356062	UP|Q41768_MAIZE (Q41768) Acetohydroxyacid synthase	0.01	
	7000	TC356062	UP|Q41768_MAIZE (Q41768) Acetohydroxyacid synthase		-0.01
	9402	TC315919	Zea mays clone Contig499 mRNA sequence	0.01	
	14820	TC346965	UP|Q2QXY9_ORYSA (Q2QXY9) 2-isopropylmalate synthase, 19%		0.01
	3314	TC316318	Zea mays clone EK07D2310G02.c mRNA sequence	-0.01	
	270	TC365751	UP|ALA2_PANMI (P34106) Alanine aminotransferase 2		-0.01
	9111	TC365751	UP|ALA2_PANMI (P34106) Alanine aminotransferase 2		0.01
	4256	TC328550	UP|AGT23_ARATH (Q9SR86) Alanine--glyoxylate aminotransferase 2 homolog 3, mitochondrial precursor, 65%		0.01

		760	TC344923	UP|Q43305_PANMI (Q43305) Aspartate aminotransferase precursor	-0.01	-0.01
		1291	TC318365	UP|Q5F4K8_PINPS (Q5F4K8) Aspartate aminotransferase, 77%		-0.01
		10570	TC353544	Zea mays clone EL01N0518A07.d mRNA sequence		0.01
		5945	TC332178	UP|HIS2_ARATH (O82768) Histidine biosynthesis bifunctional protein hisIE, chloroplast precursor [Includes: Phosphoribosyl-AMP cyclohydrolase (PRA-CH) Phosphoribosyl-ATP pyrophosphatase (PRA-PH)] , 78%	0.01	0.01
		11979	TC341041	UP|KPRS3_ORYSA (Q8S2E5) Ribose-phosphate pyrophosphokinase 3, 26%		-0.01
		29	TC317395	UP|CYSK_MAIZE (P80608) Cysteine synthase		-0.02
		6597	TC326159	UP|Q9XEA7_ORYSA (Q9XEA7) Cysteine synthase, 90%		0.01
		11540	TC323544	UP|Q9XEA9_ORYSA (Q9XEA9) Cysteine synthase, 94%	0.01	
		528	TC317782	UP|Q8GZP9_MAIZE (Q8GZP9) Satase isoform III		-0.01
		8136	TC368200	UP|Q9FPJ3_ARATH (Q9FPJ3) AT4g13930, 31%		0.02
		3104	TC351802	Zea mays clone EL01N0552F04.c mRNA sequence		0.01
Biodegradation of Xenobiotics	Lactoylglutathione lyase	2583	TC335036	UP|Q6XC06_MAIZE (Q6XC06) Glyoxalase I, 49%	-0.01	0.01
		4427	TC337595	UP|LGUL_CICAR (O49818) Lactoylglutathione lyase, 90%	-0.01	
		7653	TC356842	UP|Q6XC06_MAIZE (Q6XC06) Glyoxalase I, 22%		-0.01
		13873	TC316141	UP|Q6XC06_MAIZE (Q6XC06) Glyoxalase I		-0.01
C1-metabolism	Formate dehydrogenase	2947	TC316276	UP|FDH1_ORYSA (Q9SXP2) Formate dehydrogenase 1, mitochondrial precursor		0.01
	Glycine hydroxymethyltransferase	8136	TC368200	UP|Q9FPJ3_ARATH (Q9FPJ3) AT4g13930, 31%		0.02

	Methylenetetrahydrofolate dehydrogenase & Methenyltetrahydrofolate cyclohydrolase	12580	TC330600	Zea mays clone EK07D2304H10.c mRNA sequence		-0.01
	S-(hydroxymethyl)glutathione dehydrogenase & S-(hydroxymethyl)glutathione synthase	6744	TC330451	UP|ADHX_MAIZE (P93629) Alcohol dehydrogenase class 3	0.01	
Cell wall	Cell wall proteins	9693	TC365415	Zea mays clone EL01N0516D04.c mRNA sequence		-0.01
		5160	TC327388	UP|UPTG_MAIZE (P80607) Alpha-1, 4-glucan-protein synthase, 51%	0.01	
	Cellulose synthesis	1633	TC315858	UP|Q9LLI2_MAIZE (Q9LLI2) Cellulose synthase-8		-0.01
		8732	TC316442	UP|Q9LLI5_MAIZE (Q9LLI5) Cellulose synthase-5		0.01
		11228	TC320894	RF|NP_174697.1|15218606|NM_103160 nucleic acid binding {Arabidopsis thaliana}, 40%	0.01	
		12093	TC315858	UP|Q9LLI2_MAIZE (Q9LLI2) Cellulose synthase-8	-0.01	
		5938	TC318468	UP|Q5YLM2_MAIZE (Q5YLM2) Roothairless 3		0.01
	Degradation	2902	TC354379	UP|Q8L3P5_ORYSA (Q8L3P5) Beta-galactosidase, 52%	-0.01	
		6286	TC357953	UP|Q68UW0_PYRCO (Q68UW0) Beta-galactosidase, 14%		0.01
		8778	TC354379	UP|Q8L3P5_ORYSA (Q8L3P5) Beta-galactosidase, 52%		0.02
	Modification	3293	TC322932	UP|Q94KT6_MAIZE (Q94KT6) Alpha-expansin 2		-0.01
		4149	TC328174	UP|Q4F986_LYCES (Q4F986) Xyloglucan endotransglycosylase/hydrolase 16 protein, 65%	-0.01	
		12495	TC320186	UP|P93671_HORVU (P93671) Xyloglucan endotransglycosylase (XET), 92%	-0.01	-0.01
	Pectin*esterases	12184	TC316093	Zea mays clone Contig500 mRNA sequence	-0.02	
		4318	TC342734	RF|NP_181209.1|15228023|NM_129226 pectinesterase {Arabidopsis thaliana}, 22%	-0.01	

		4998	TC347297	UP|Q8LQ65_ORYSA (Q8LQ65) Pectin methyl esterase-like, 95%	0.01	
	Precursor synthesis	2762	TC358452	UP|Q6DW08_MEDSA (Q6DW08) GMPase, 24%	-0.02	
		14341	TC315994	UP|Q84TT8_COLES (Q84TT8) UDP-glucose dehydrogenase		-0.02
		11111	TC352521	UP|Q58IJ5_HORVU (Q58IJ5) UDP-D-glucose epimerase 2, 53%		-0.01
		12953	TC364281	UP|Q9FSE2_PHRAU (Q9FSE2) D-TDP-glucose dehydratase		0.01
Cell	Cycle	1255	TC326770	UP|Q6PS57_ORYSA (Q6PS57) Cyclin-dependent kinase subunit, 91%	0.02	
		2523	TC320136	UP|O65065_PICMA (O65065) PREG-like protein, 40%		0.01
		5634	TC316094	UP|Q41734_MAIZE (Q41734) Cyclin IaZm	-0.01	
		8971	TC363585	UP|Q41734_MAIZE (Q41734) Cyclin IaZm, 88%	-0.01	
		11674	TC316210	UP|CDC22_ORYSA (P29619) Cell division control protein 2 homolog 2		-0.02
		11680	TC316094	UP|Q41734_MAIZE (Q41734) Cyclin IaZm	0.01	
		12507	TC326770	UP|Q6PS57_ORYSA (Q6PS57) Cyclin-dependent kinase subunit, 91%	-0.01	
		808	TC371041	UP|CYPH_MAIZE (P21569) Peptidyl-prolyl cis-trans isomerase		-0.01
		2017	TC371041	UP|CYPH_MAIZE (P21569) Peptidyl-prolyl cis-trans isomerase	-0.01	
		3629	TC371041	UP|CYPH_MAIZE (P21569) Peptidyl-prolyl cis-trans isomerase	-0.01	
		5239	TC371041	UP|CYPH_MAIZE (P21569) Peptidyl-prolyl cis-trans isomerase	-0.01	
		6023	TC371041	UP|CYPH_MAIZE (P21569) Peptidyl-prolyl cis-trans isomerase		0.01
		6154	TC371041	UP|CYPH_MAIZE (P21569) Peptidyl-prolyl cis-trans isomerase		0.01
		6829	TC371041	UP|CYPH_MAIZE (P21569) Peptidyl-prolyl cis-trans isomerase	-0.01	
		7477	TC347403	RF|NP_914824.1|34906954|NM_189935 rapamycin-binding protein-like {Oryza sativa (japonica cultivar-group)}, 86%		0.01
		10182	TC323894	UP|FKB70_WHEAT (Q43207) 70 kDa peptidyl-prolyl isomerase, 97%	-0.01	

	10840	TC371041	UP|CYPH_MAIZE (P21569) Peptidyl-prolyl cis-trans isomerase		0.01
	11211	TC330032	GB|AAC49390.1|1272406|ATU52046 immunophilin {Arabidopsis thaliana}, 83%	0.01	
	11756	TC316397	Zea mays clone EL01N0530F05.c mRNA sequence		0.01
Division	815	TC328183	Zea mays clone EL01N0526G04.d mRNA sequence	-0.01	-0.01
	851	TC369924	UP|Q84YE5_SORBI (Q84YE5) Cyclin-dependent kinase-like protein, 45%		-0.01
	3770	TC316332	UP|Q6Z8N6_ORYSA (Q6Z8N6) Protein cdc2 kinase	-0.01	
	3917	TC320602	UP|APC10_ARATH (Q9ZPW2) Anaphase-promoting complex subunit 10, 92%		-0.01
	5274	TC316332	UP|Q6Z8N6_ORYSA (Q6Z8N6) Protein cdc2 kinase		0.01
	6055	TC359645	UP|Q7XE16_ORYSA (Q7XE16) AAA family ATPase, CDC48 subfamily, 98%	-0.01	
	6098	TC346200	UP|Q3LXA7_MAIZE (Q3LXA7) Retinoblastoma-related 3, 46%		0.01
	8108	TC360599	UP|Q9LTY1_ARATH (Q9LTY1) Mitotic checkpoint protein-like, 7%	0.01	0.01
	8833	TC316332	UP|Q6Z8N6_ORYSA (Q6Z8N6) Protein cdc2 kinase		0.01
	10447	TC340599			0.01
	10450	TC359645	UP|Q7XE16_ORYSA (Q7XE16) AAA family ATPase, CDC48 subfamily, 98%	-0.01	
	13538	TC329359	UP|Q2HSK8_MEDTR (Q2HSK8) Regulator of chromosome condensation/beta-lactamase-inhibitor protein II, 18%		0.02
Organization	148	TC367492	UP|TBA1_MAIZE (P14640) Tubulin alpha-1 chain		-0.01
	433	TC334942	UP|Q811M0_MOUSE (Q811M0) Hist1h4h protein, 97%		-0.01
	718	TC329678	UP|Q8S912_TOBAC (Q8S912) Microtubule bundling polypeptide TMBP200, 11%		-0.01
	840	-	-	-0.01	
	876	TC364898	UP|TBB1_MAIZE (P18025) Tubulin beta-1 chain		-0.01
	1149	TC320110	UP|Q7XI08_ORYSA (Q7XI08) Auxin-regulated protein-like protein, 94%	-0.01	

1222	TC347779	GB|BAC78565.1|32352144|AB110173 ankyrin {Oryza sativa (japonica cultivar-group)}, 80%		-0.01
1255	TC326770	UP|Q6PS57_ORYSA (Q6PS57) Cyclin-dependent kinase subunit, 91%	0.02	
1296	TC321524	UP|Q6K5G3_ORYSA (Q6K5G3) Ankyrin repeat-like protein, 87%		-0.01
1341	TC361943	UP|Q6Z2W0_ORYSA (Q6Z2W0) Chromosome-associated kinesin-like, 58%		-0.02
1505	TC318412	GB|CAJ31078.1|76058012|AM086438 70 kDa microtubule associated protein Type 1 {Arabidopsis thaliana}, 72%		0.01
1627	TC357284	UP|Q9ZVV2_ARATH (Q9ZVV2) T5A14.3 protein, 30%		-0.01
1734	TC356501	UP|TBA3_MAIZE (P22275) Tubulin alpha-3 chain		-0.01
2034	TC364640	UP|MNB1B_MAIZE (P27347) DNA-binding protein MNB1B		-0.01
2037	TC319386	UP|ADF3_MAIZE (Q41764) Actin-depolymerizing factor 3	-0.01	
2626	TC363214	UP|Q5ZEA0_ORYSA (Q5ZEA0) Kinesin heavy chain-like, 48%		0.01
2736	TC338133	UP|Q9SQH4_LILLO (Q9SQH4) Actin bundling protein ABP135, 22%		0.01
2859	TC330938	UP|Q9SAF1_ARATH (Q9SAF1) F3F19.20 protein (Actin-related protein 3) (At1g13180), 79%	-0.01	0.01
2878	TC321524	UP|Q6K5G3_ORYSA (Q6K5G3) Ankyrin repeat-like protein, 87%		0.02
2980	TC333073	UP|Q9SAF1_ARATH (Q9SAF1) F3F19.20 protein (Actin-related protein 3) (At1g13180), 59%		-0.01
3147	TC366766	UP|TBA2_MAIZE (P14641) Tubulin alpha-2 chain	-0.01	
3172	TC342944	UP|Q9FW70_ORYSA (Q9FW70) Kinesin-like protein, 33%		0.01
3455	TC344584	UP|Q5XPX5_SACOF (Q5XPX5) Actin	-0.01	
3601	TC327546	UP|Q9SXG0_ORYSA (Q9SXG0) F1F0-ATPase inhibitor protein, 93%		-0.02
3641	TC335773	UP|TBB7_MAIZE (Q41784) Tubulin beta-7 chain, 52%	-0.02	
4016	TC331931	UP|Q2RBQ8_ORYSA (Q2RBQ8) Expressed protein, 98%	-0.01	
4200	TC333132	UP|Q43863_MAIZE (Q43863) Annexin p33	-0.01	

4252	TC348819	UP|Q93XF4_MAIZE (Q93XF4) Kinesin heavy chain	-0.01	
4516	TC316076	UP|Q4A190_MAIZE (Q4A190) Beclin 1 protein	-0.01	
4845	TC364898	UP|TBB1_MAIZE (P18025) Tubulin beta-1 chain	-0.01	
5679	TC348819	UP|Q93XF4_MAIZE (Q93XF4) Kinesin heavy chain		0.01
5684	TC327439	UP|PROF5_MAIZE (Q9FR39) Profilin-5 (ZmPRO5)		0.01
5751	TC356501	UP|TBA3_MAIZE (P22275) Tubulin alpha-3 chain		0.01
6805	TC317247	Zea mays clone Contig385.F mRNA sequence		-0.01
7138	TC342814	UP|ACT1_ORYSA (P13362) Actin-1		0.01
8143	TC324564	UP|Q651Z1_ORYSA (Q651Z1) TMV-MP30 binding protein 2C-like, 94%		-0.01
8446	TC361063	RF|XP_506618.1|51963694|XM_506618 P0015C07.29 gene product {Oryza sativa (japonica cultivar-group)}, 37%		0.02
8516	TC362989	UP|TBA1_MAIZE (P14640) Tubulin alpha-1 chain		0.01
8670	TC345256	UP|Q4SKJ3_TETNG (Q4SKJ3) Chromosome undetermined SCAF14565, whole genome shotgun sequence, 47%		0.01
8713	TC326972	UP|PROF5_MAIZE (Q9FR39) Profilin-5 (ZmPRO5)	0.01	
8819	TC362989	UP|TBA1_MAIZE (P14640) Tubulin alpha-1 chain		-0.01
8871	TC359347	UP|TBB5_MAIZE (Q43697) Tubulin beta-5 chain	-0.01	-0.01
8888	TC342814	UP|ACT1_ORYSA (P13362) Actin-1		0.01
8913	TC335773	UP|TBB7_MAIZE (Q41784) Tubulin beta-7 chain, 52%	-0.01	-0.01
9486	TC333132	UP|Q43863_MAIZE (Q43863) Annexin p33	0.01	
9673	TC327509	UP|TBB3_WHEAT (Q9ZRB0) Tubulin beta-3 chain, 71%		-0.01
10079	TC332697	UP|Q65C76_SETVI (Q65C76) Beta tubulin, 49%	-0.01	-0.02

	10084	TC367492	UP|TBA1_MAIZE (P14640) Tubulin alpha-1 chain	-0.01	-0.01
	10086	TC332697	UP|Q65C76_SETVI (Q65C76) Beta tubulin, 49%		0.01
	10147	TC336624	UP|Q6H7Q8_ORYSA (Q6H7Q8) Tubulin-specific chaperone C-like protein, 22%		-0.01
	10227	TC359833	UP|Q2QLT8_ORYSA (Q2QLT8) Cofilin/tropomyosin-type actin-binding protein		0.01
	10463	TC339557	UP|TBB7_MAIZE (Q41784) Tubulin beta-7 chain		0.01
	10474	-	-		0.01
	10533	TC329923	UP|Q9FQL7_MAIZE (Q9FQL7) Kinesin-like calmodulin binding protein	-0.01	
	10535	TC356501	UP|TBA3_MAIZE (P22275) Tubulin alpha-3 chain	-0.01	
	10898	TC347779	GB|BAC78565.1|32352144|AB110173 ankyrin {Oryza sativa (japonica cultivar-group)}, 80%		-0.01
	10974	TC362989	UP|TBA1_MAIZE (P14640) Tubulin alpha-1 chain	-0.01	
	11230	TC365565	UP|METK_ORYSA (P46611) S-adenosylmethionine synthetase 1		-0.01
	11264	TC332697	UP|Q65C76_SETVI (Q65C76) Beta tubulin, 49%	0.02	
	12190	TC367492	UP|TBA1_MAIZE (P14640) Tubulin alpha-1 chain		-0.01
	12329	TC324564	UP|Q651Z1_ORYSA (Q651Z1) TMV-MP30 binding protein 2C-like, 94%		0.01
	12447	TC359347	UP|TBB5_MAIZE (Q43697) Tubulin beta-5 chain		-0.01
	12507	TC326770	UP|Q6PS57_ORYSA (Q6PS57) Cyclin-dependent kinase subunit, 91%	-0.01	
	12573	TC354205	UP|ADF3_MAIZE (Q41764) Actin-depolymerizing factor 3		0.01
	12641	TC338056	UP|Q76N07_SOLME (Q76N07) Histone H4-like protein		0.02
	13651	TC338073	UP|Q5ZB07_ORYSA (Q5ZB07) Myosin heavy chain-like, 20%	0.02	
	13802	TC342814	UP|ACT1_ORYSA (P13362) Actin-1	0.01	
	15107	TC337187	UP|Q6K5K0_ORYSA (Q6K5K0) Myosin-like protein, 24%	0.01	0.01

	Vesicle transport	205	TC358218	UP|Q9M640_MAIZE (Q9M640) Delta-COP, 24%		-0.01
		302	TC366946	UP|Q9SV20_ARATH (Q9SV20) Beta-COP-like protein, 21%	0.01	
		1764	TC323514	UP|Q69WS1_ORYSA (Q69WS1) Synaptobrevin-like protein (Synaptobrevin 1)	-0.01	
		1921	TC358944	UP|Q2QW51_ORYSA (Q2QW51) Clathrin assembly protein AP17-like protein	-0.01	
		2526	TC371073	UP|Q9M639_MAIZE (Q9M639) Epsilon-COP	-0.01	
		5385	TC323514	UP|Q69WS1_ORYSA (Q69WS1) Synaptobrevin-like protein (Synaptobrevin 1)		0.01
		6080	TC317682	Zea mays clone Contig617.F mRNA sequence		0.01
		6404	TC317682	Zea mays clone Contig617.F mRNA sequence		0.01
		6523	TC358218	UP|Q9M640_MAIZE (Q9M640) Delta-COP, 24%		0.01
		7140	TC316498	UP|Q8S0N4_ORYSA (Q8S0N4) Vesicle transport v-SNARE (Vesicle soluble NSF attachment protein receptor) protein-like		0.01
		7831	TC349957	UP|Q9SV20_ARATH (Q9SV20) Beta-COP-like protein, 7%		0.01
		12519	TC326053	UP|Q69WS1_ORYSA (Q69WS1) Synaptobrevin-like protein (Synaptobrevin 1)		0.01
		12590	TC323018	RF|NP_564138.1|18394983|NM_101990 protein transporter {Arabidopsis thaliana}, 51%	-0.01	
Co-factor and vitamine metabolism	Biotin	5507	TC346678	UP|Y1280_ARATH (O80543) Protein At1g22800, 76%	0.01	
		5726	-	-		0.01
	Folate & vitamine K.folate	12830	TC328784	GB|AAC83041.1|3834325|F9K20 Strong similarity to gb|AF067141 gamma-glutamyl hydrolase from ESTs gb|R83955, gb|T45062, gb|T22220, gb|AA586207, gb|AI099851 and gb|AI00672 come from this gene. {Arabidopsis thaliana}, 83%	0.01	
Development	Late embryogenesis abundant	7340	TC324387	UP|Q7XC31_ORYSA (Q7XC31) 60S ribosomal protein L27		-0.02
	Multitarget	5821	TC329152	UP|Q5EFI5_MAIZE (Q5EFI5) Target of rapamycin, 10%		0.01
	Storage proteins	1146	TC364534	UP|Q946V6_MAIZE (Q946V6) 19kD alpha zein B1	-0.01	

	9974	TC356334	UP|Q41883_MAIZE (Q41883) Zein		-0.02
	10746	TC364534	UP|Q946V6_MAIZE (Q946V6) 19kD alpha zein B1		0.01
	15086	TC364534	UP|Q946V6_MAIZE (Q946V6) 19kD alpha zein B1	0.02	
Unspecified	199	TC345741	UP|Q6K972_ORYSA (Q6K972) AGO1 homologous protein, 28%		-0.01
	252	TC328398	Zea mays clone EL01N0551E04.c mRNA sequence		-0.02
	1022	TC332106	Zea mays clone EL01N0526B05.d mRNA sequence		0.01
	2404	TC325067	UP|Q3HVK6_SOLTU (Q3HVK6) Drm3-like protein, 48%	-0.01	
	2764	TC324694	Zea mays clone E04912704G01.c mRNA sequence	-0.01	
	2836	TC341177	UP|Q2LFC4_NICBE (Q2LFC4) AGO1-1, 43%		-0.02
	3056	-	-		-0.01
	3151	TC332072	UP|O82787_ORYSA (O82787) Early nodulin	-0.01	
	3671	TC335389	UP|Q656P7_ORYSA (Q656P7) Root hair defective 3 GTP-binding protein-like, 40%		0.01
	3732	TC345741	UP|Q6K972_ORYSA (Q6K972) AGO1 homologous protein, 28%	0.01	
	4156	TC327894	UP|Q8LQG0_ORYSA (Q8LQG0) Leaf senescence protein-like, 36%	-0.01	
	5266	TC358593			0.01
	5506	TC351999	UP|Q67VC5_ORYSA (Q67VC5) DeliriumA-like, 78%		0.01
	5911	TC347801	UP|Q2LFC3_NICBE (Q2LFC3) AGO1-2, 6%		0.01
	5964	TC332381	UP|Q56YT3_ARATH (Q56YT3) Squamosa promoter binding protein-like 1, 25%		0.01
	7128	TC342304	RF|NP_914460.1|34906226|NM_189571 gigantea-like protein {Oryza sativa (japonica cultivar-group)}, 17%	0.01	
	7559	TC354675	UP|O82787_ORYSA (O82787) Early nodulin, 98%	-0.01	
	8170	TC318199	UP|Q5ZCB3_ORYSA (Q5ZCB3) Seven in absentia protein-like, 17%	0.01	

		8436	TC360988	UP|Q654W0_ORYSA (Q654W0) G-box binding protein-like, 94%		-0.01
		8566	TC327894	UP|Q8LQG0_ORYSA (Q8LQG0) Leaf senescence protein-like, 36%	0.01	
		9975	TC329769	UP|Q4QWQ6_SACOF (Q4QWQ6) NAC23, 98%		-0.01
		10637	TC325826	UP|Q9LKY2_MAIZE (Q9LKY2) WD-repeat protein RBAP1, 88%		-0.01
		10816	TC321315	RF|NP_564995.1|18409649|NM_105735 calcium ion binding {Arabidopsis thaliana}, 83%		0.01
		11752	TC360988	UP|Q654W0_ORYSA (Q654W0) G-box binding protein-like, 94%	0.01	
		12174	TC353700	UP|Q400H8_ELAGV (Q400H8) AP1-like MADS box transcription factor, 35%		-0.01
		12902	TC328313	UP|Q6Z663_ORYSA (Q6Z663) WD-40 repeat protein-like	0.02	
		15568	TC320419	GB|AAP13420.1|30023774|BT006312 At3g45600 {Arabidopsis thaliana}, 65%		-0.01
DNA	Repair	488	TC356105	RF|XP_506511.1|51963604|XM_506511 OJ1458_B07.103 gene product {Oryza sativa (japonica cultivar-group)}, 21%		-0.01
		4653	TC347035	UP|Q9FQ08_ARATH (Q9FQ08) Ku70-like protein, 75%		0.01
		12191	TC337580	GB|ABB86293.1|82621223|DQ284987 DNA replication protein A2 subunit {Arabidopsis thaliana}, 14%	0.01	
		12931	TC329032	UP|Q69KV4_ORYSA (Q69KV4) Trad-like protein, 69%		0.01
		13798	TC328654	GB|AAL87405.1|19548081|AY081835 At5g38470/At5g38470 {Arabidopsis thaliana}, 40%		0.01
	Synthesis/chromatin structure	479	TC352342	UP|Q93VJ8_ARATH (Q93VJ8) AT5g11200/F2I11_90 (AT5g11170/F2I11_60), 57%		-0.02
		594	TC355960	UP|Q5I285_MAIZE (Q5I285) Minichromosome maintenance protein, 98%		0.01
		975	TC342285	RF|NP_190333.1|15232817|NM_114617 endonuclease/ nucleic acid binding {Arabidopsis thaliana}, 55%		-0.01
		1057	TC352008	RF|NP_179236.3|42569072|NM_127197 ATP binding {Arabidopsis thaliana}, 73%	-0.01	
		1692	TC354035	UP|Q84UD2_HORVD (Q84UD2) GAMYB-binding protein, 47%	-0.01	

3548	TC344117	UP|DKC1_ARATH (Q9LD90) H/ACA ribonucleoprotein complex subunit 4 (Nucleolar protein NAP57 homolog) (Nopp-140-associated protein of 57 kDa homolog) (AtNAP57) , 48%	0.01	
3579	TC345216	UP|DPOD1_ORYSA (Q9LRE6) DNA polymerase delta catalytic subunit, 26%	-0.01	
4600	-	-		0.01
5366	TC358511	UP|Q8GSC4_TOBAC (Q8GSC4) DNA topoisomerase II, 19%		0.01
6063	TC322529	UP|Q9FJW0_ARATH (Q9FJW0) RuvB DNA helicase-like protein (AT5g67630/K9I9_20), 94%		0.01
6093	TC320791	UP|Q5ZPI9_ORYSA (Q5ZPI9) Topoisomerase 6 subunit A, 98%		0.01
6941	TC324617	UP|Q9LTV0_ARATH (Q9LTV0) Nucleolar protein, 86%		-0.01
8122	-	-		0.01
9403	TC324134	UP|Q94F78_MAIZE (Q94F78) Nucleosome/chromatin assembly factor A		-0.01
9953	TC333500	Zea mays clone EL01T0205D01.c mRNA sequence		-0.01
10976	TC324617	UP|Q9LTV0_ARATH (Q9LTV0) Nucleolar protein, 86%	-0.01	-0.01
11260	TC337427			0.01
12225	TC325043		-0.01	
15083	TC357068	UP|Q84UX6_MAIZE (Q84UX6) Nucleosome/chromatin assembly factor group A	0.01	
5	TC345524	UP|H2B2_MAIZE (P30756) Histone H2B.2		-0.01
88	TC329978	UP|H2B2_MAIZE (P30756) Histone H2B.2		-0.01
112	TC327070	UP|Q6LB28_LYCES (Q6LB28) Histone H3 variant H3.3		-0.01
433	TC334942	UP|Q811M0_MOUSE (Q811M0) Hist1h4h protein, 97%		-0.01
437	TC365360	UP|H2B3_MAIZE (Q43261) Histone H2B.3		-0.01
446	TC318413	UP|H2B1_MAIZE (P30755) Histone H2B.1		-0.01
485	TC357259	UP|H2A_MAIZE (P40280) Histone H2A		-0.02

534	TC341422	UP|Q811M0_MOUSE (Q811M0) Hist1h4h protein, 97%		-0.01
577	TC336265	UP|H2B1_WHEAT (P27807) Histone H2B, 98%	-0.01	
592	TC349142	UP|H2B5_MAIZE (P54348) Histone H2B		-0.01
657	TC325723	UP|H2AV3_ORYSA (Q84MP7) Probable histone H2A variant 3		-0.01
830	TC327808	UP|Q76MV0_TOBAC (Q76MV0) H3 histone		-0.01
1293	TC331139	UP|Q76MV0_TOBAC (Q76MV0) H3 histone		-0.02
1691	TC339929	UP|Q8W120_MAIZE (Q8W120) Histone H1-like protein		-0.02
1717	TC359088	UP|Q76MV0_TOBAC (Q76MV0) H3 histone	-0.02	
1841	TC341191	UP|Q6LB28_LYCES (Q6LB28) Histone H3 variant H3.3	0.01	
2026	TC359346	UP|Q4ABW1_BRARP (Q4ABW1) 4D11_26, 37%	-0.01	
2438	TC357608	UP|Q76MV0_TOBAC (Q76MV0) H3 histone	-0.01	-0.01
2818	TC358538	UP|Q76MV0_TOBAC (Q76MV0) H3 histone	-0.01	
2835	TC331576	UP|Q30DN4_ORYSA (Q30DN4) Hd1, 12%		-0.01
2967	TC336965	UP|Q8CGN9_MOUSE (Q8CGN9) Histone protein Hist2h3c1 (H3 histone, family 2), 78%		0.01
2970	TC316760	UP|H1_MAIZE (P23444) Histone H1		0.01
3154	TC341323	UP|Q76N07_SOLME (Q76N07) Histone H4-like protein	-0.01	
3265	TC364001	UP|H2A_MAIZE (P40280) Histone H2A, 57%	-0.01	
3696	TC335618	UP|H2A_MAIZE (P40280) Histone H2A, 93%		0.01
4413	TC347766	UP|Q76N07_SOLME (Q76N07) Histone H4-like protein	-0.01	
4553	TC365788	UP|H2A_MAIZE (P40280) Histone H2A	-0.01	
4557	TC342976	UP|H2AV3_ORYSA (Q84MP7) Probable histone H2A variant 3	-0.01	

4591	TC347498	UP|Q76MV0_TOBAC (Q76MV0) H3 histone		0.02
4898	TC327808	UP|Q76MV0_TOBAC (Q76MV0) H3 histone		0.01
4993	TC363966	UP|Q76N07_SOLME (Q76N07) Histone H4-like protein		0.01
5337	TC321087	UP|H2A_MAIZE (P40280) Histone H2A, 81%		0.01
5616	TC359346	UP|Q4ABW1_BRARP (Q4ABW1) 4D11_26, 37%		0.01
5704	TC329978	UP|H2B2_MAIZE (P30756) Histone H2B.2		0.01
6026	TC349142	UP|H2B5_MAIZE (P54348) Histone H2B	-0.01	
6155	TC365788	UP|H2A_MAIZE (P40280) Histone H2A		0.01
6166	TC355183	UP|H2B2_MAIZE (P30756) Histone H2B.2		0.01
6183	TC357259	UP|H2A_MAIZE (P40280) Histone H2A		0.01
6198	TC341774	UP|Q76N07_SOLME (Q76N07) Histone H4-like protein	0.01	
6284	TC366521	UP|Q9SGE3_ARATH (Q9SGE3) T23G18.3 (T6D22.26) (Histone H2B family protein), 34%	0.01	0.02
6487	TC331139	UP|Q76MV0_TOBAC (Q76MV0) H3 histone	-0.01	
6590	TC327808	UP|Q76MV0_TOBAC (Q76MV0) H3 histone	-0.01	
6878	TC333345	UP|Q76N07_SOLME (Q76N07) Histone H4-like protein	0.01	
7366	TC334942	UP|Q811M0_MOUSE (Q811M0) Hist1h4h protein, 97%	0.01	
7395	TC349142	UP|H2B5_MAIZE (P54348) Histone H2B		0.01
7633	TC363966	UP|Q76N07_SOLME (Q76N07) Histone H4-like protein	0.01	
7643	TC349142	UP|H2B5_MAIZE (P54348) Histone H2B		0.01
7687	TC363966	UP|Q76N07_SOLME (Q76N07) Histone H4-like protein		0.01
7740	TC321585	UP|H2B1_WHEAT (P27807) Histone H2B, 97%		-0.02

7794	TC341774	UP|Q76N07_SOLME (Q76N07) Histone H4-like protein	0.01	
8060	TC327070	UP|Q6LB28_LYCES (Q6LB28) Histone H3 variant H3.3	0.01	
8076	TC336340	UP|H2B4_MAIZE (P49120) Histone H2B.4	-0.01	
8109	TC342976	UP|H2AV3_ORYSA (Q84MP7) Probable histone H2A variant 3	-0.01	
8414	TC336265	UP|H2B1_WHEAT (P27807) Histone H2B, 98%		-0.01
8471	TC345967	UP|Q76N07_SOLME (Q76N07) Histone H4-like protein	0.01	
8670	TC345256	UP|Q4SKJ3_TETNG (Q4SKJ3) Chromosome undetermined SCAF14565, whole genome shotgun sequence, 47%		0.01
8869	TC324223	UP|H2A_MAIZE (P40280) Histone H2A, 96%	-0.01	
9046	TC338967	UP|Q76MV0_TOBAC (Q76MV0) H3 histone		0.01
9067	TC336340	UP|H2B4_MAIZE (P49120) Histone H2B.4		-0.01
9244	TC336965	UP|Q8CGN9_MOUSE (Q8CGN9) Histone protein Hist2h3c1 (H3 histone, family 2), 78%		0.02
9258	TC355183	UP|H2B2_MAIZE (P30756) Histone H2B.2		0.01
9306	TC359088	UP|Q76MV0_TOBAC (Q76MV0) H3 histone	0.01	
9383	TC336265	UP|H2B1_WHEAT (P27807) Histone H2B, 98%		-0.01
9393	TC359346	UP|Q4ABW1_BRARP (Q4ABW1) 4D11_26, 37%		0.01
9601	TC338511	UP|H2A_MAIZE (P40280) Histone H2A, 92%		0.01
9704	TC318102	UP|Q8LK07_MAIZE (Q8LK07) Histone H1-like protein HON101	0.01	
9715	TC327808	UP|Q76MV0_TOBAC (Q76MV0) H3 histone	0.01	
10024	TC318102	UP|Q8LK07_MAIZE (Q8LK07) Histone H1-like protein HON101	-0.01	
10169	TC336340	UP|H2B4_MAIZE (P49120) Histone H2B.4	-0.01	
10479	TC355183	UP|H2B2_MAIZE (P30756) Histone H2B.2		0.01

10510	TC331031	UP|H2A_MAIZE (P40280) Histone H2A	0.01	
10524	TC359346	UP|Q4ABW1_BRARP (Q4ABW1) 4D11_26, 37%		0.01
10653	TC330119	UP|H2B1_WHEAT (P27807) Histone H2B, 97%		0.01
10813	TC342976	UP|H2AV3_ORYSA (Q84MP7) Probable histone H2A variant 3	-0.01	
10824	TC341422	UP|Q811M0_MOUSE (Q811M0) Hist1h4h protein, 97%		-0.01
10848	TC328193	UP|H2A_MAIZE (P40280) Histone H2A	0.01	
10864	TC341422	UP|Q811M0_MOUSE (Q811M0) Hist1h4h protein, 97%	0.01	
10891	TC327808	UP|Q76MV0_TOBAC (Q76MV0) H3 histone	0.01	
10938	TC329978	UP|H2B2_MAIZE (P30756) Histone H2B.2	0.01	
10977	TC336265	UP|H2B1_WHEAT (P27807) Histone H2B, 98%	-0.01	
11254	TC341533	UP|Q76N07_SOLME (Q76N07) Histone H4-like protein	0.01	
11268	TC338056	UP|Q76N07_SOLME (Q76N07) Histone H4-like protein		-0.02
11660	TC329978	UP|H2B2_MAIZE (P30756) Histone H2B.2		0.01
11679	TC336965	UP|Q8CGN9_MOUSE (Q8CGN9) Histone protein Hist2h3c1 (H3 histone, family 2), 78%	0.01	
11721	TC327808	UP|Q76MV0_TOBAC (Q76MV0) H3 histone	0.01	
11740	TC365788	UP|H2A_MAIZE (P40280) Histone H2A	0.01	
11757	TC327808	UP|Q76MV0_TOBAC (Q76MV0) H3 histone	0.01	
12028	TC335618	UP|H2A_MAIZE (P40280) Histone H2A, 93%		0.01
12157	TC335527	UP|H2B5_MAIZE (P54348) Histone H2B, 98%		0.01
12176	TC355183	UP|H2B2_MAIZE (P30756) Histone H2B.2		0.01
12515	TC329978	UP|H2B2_MAIZE (P30756) Histone H2B.2		-0.01

		12532	TC349142	UP|H2B5_MAIZE (P54348) Histone H2B		0.01
		12641	TC338056	UP|Q76N07_SOLME (Q76N07) Histone H4-like protein		0.02
		13591	TC342976	UP|H2AV3_ORYSA (Q84MP7) Probable histone H2A variant 3		-0.01
		13837	TC337000	UP|H2B4_MAIZE (P49120) Histone H2B.4	0.01	
		14434	TC351320	UP|H2B4_MAIZE (P49120) Histone H2B.4		-0.02
		15020	TC349142	UP|H2B5_MAIZE (P54348) Histone H2B	0.02	
		11570	TC341955	UP|Q3LAG0_WHEAT (Q3LAG0) Transposase-related protein w-gary2, 10%		0.01
	Unspecified	759	TC329389	Zea mays clone EL01T0403F08.c mRNA sequence	-0.01	
		981	TC365295			-0.01
		2406	TC324243	GB|AAQ65156.1|34365689|BT010533 At1g03360 {Arabidopsis thaliana}, 16%		-0.01
		4075	TC323772	RF|XP_506641.1|51963722|XM_506641 P0523B07.38-1 gene product {Oryza sativa (japonica cultivar-group)}		0.01
		5129	TC333034	UP|Q6ZCZ7_ORYSA (Q6ZCZ7) Phosphatidylinositol transfer-like, 57%		0.01
		5148	TC317455	UP|Q9FMK4_ARATH (Q9FMK4) Topoisomerase-like protein, 70%	0.01	
		5859	TC330704	UP|Q3EAL4_ARATH (Q3EAL4) Protein At3g52050, 44%		0.01
		7667	TC338556	RF|NP_177653.1|15222153|NM_106173 transporter {Arabidopsis thaliana}, 68%		-0.01
Fermentation	Aldehyde dehydrogenase	2547	TC316041	UP|Q8S532_MAIZE (Q8S532) Cytosolic aldehyde dehydrogenase RF2C	-0.01	
		4452	TC315983	UP|Q7FWR0_MAIZE (Q7FWR0) Mitochondrial aldehyde dehydrogenase RF2B	-0.01	
		6046	TC316041	UP|Q8S532_MAIZE (Q8S532) Cytosolic aldehyde dehydrogenase RF2C	-0.01	0.01
		11248	TC315983	UP|Q7FWR0_MAIZE (Q7FWR0) Mitochondrial aldehyde dehydrogenase RF2B		0.01
		12310	TC316400	UP|Q67B30_MAIZE (Q67B30) Fatty aldehyde dehydrogenase 1	0.01	0.01
Glycolysis	Aldolase	2590	TC332540	UP|ALF_MAIZE (P08440) Fructose-bisphosphate aldolase, cytoplasmic isozyme, 49%	-0.01	

Enolase	5254	TC335086	UP|ENO2_MAIZE (P42895) Enolase 2		0.01
G6PIsomerase	10841	TC345791	UP|Q84P59_ORYSA (Q84P59) Glucose-6-phosphate isomerase-like protein, 73%	-0.01	
Glyceraldehyde 3-phosphate dehydrogenase	444	TC367854	UP|G3PD_MAIZE (Q09054) Glyceraldehyde-3-phosphate dehydrogenase, cytosolic 2		-0.01
	511	TC334343	UP|Q37265_PINSY (Q37265) Glyceraldehyde-3-phosphate dehydrogenase precursor, 35%		-0.01
	4301	TC367854	UP|G3PD_MAIZE (Q09054) Glyceraldehyde-3-phosphate dehydrogenase, cytosolic 2		0.01
	4597	TC367859	UP|Q43359_MAIZE (Q43359) Cytosolic glyceroldehyde-3-phosphate dehydrogenase GAPC4		0.01
	4798	TC361983	UP|Q43359_MAIZE (Q43359) Cytosolic glyceroldehyde-3-phosphate dehydrogenase GAPC4, 55%		0.01
	5686	TC316142	UP|G3PE_MAIZE (Q43247) Glyceraldehyde-3-phosphate dehydrogenase, cytosolic 3	-0.01	
PEPCase	494	TC339812	UP|Q9SAZ6_MAIZE (Q9SAZ6) Phosphoenolpyruvate carboxylase		-0.01
	10422	TC339812	UP|Q9SAZ6_MAIZE (Q9SAZ6) Phosphoenolpyruvate carboxylase		0.01
	10526	TC339812	UP|Q9SAZ6_MAIZE (Q9SAZ6) Phosphoenolpyruvate carboxylase		0.01
	11127	TC361054	UP|Q43267_MAIZE (Q43267) PEP carboxylase		0.01
PGM	2498	TC315904	UP|PGMC2_MAIZE (P93805) Phosphoglucomutase, cytoplasmic 2		-0.01
	9115	TC352484	UP|PGM_SCHPO (O74374) Probable phosphoglucomutase (Glucose phosphomutase) (PGM) , 14%		0.01
Phosphoglycerate kinase	7418	TC318427	UP|PGKY_WHEAT (P12783) Phosphoglycerate kinase, cytosolic		0.01
	9145	TC319599	UP|PGKY_WHEAT (P12783) Phosphoglycerate kinase, cytosolic		0.01
PK	439	TC365959	UP|Q2RAK2_ORYSA (Q2RAK2) Pyruvate kinase		-0.02
Pyrophosphate-fructose-6-P phosphotransferase	282	TC361794	RF|XP_507199.1|51964830|XM_507199 P0410E11.122 gene product {Oryza sativa (japonica cultivar-group)}, 12%		-0.01
	4897	TC330834	RF|NP_192313.2|30679628|NM_116642 6-phosphofructokinase {Arabidopsis thaliana}, 27%		0.01
	5048	TC323414	Zea mays clone Contig720.F mRNA sequence		0.01
TPI	170	TC367984	GB|AAB81110.1|168647|MZETPI2 triosephosphate isomerase 1 {Zea mays}		-0.01

		423	TC344569	GB|AAB81110.1|168647|MZETPI2 triosephosphate isomerase 1 {Zea mays}		-0.01
Hormone metabolism	Abscisic acid	12422	TC352711	RF|XP_463364.1|50901862|XM_463364 protein phosphatase 2C-like protein {Oryza sativa (japonica cultivar-group)}, 48%		-0.01
		3161	TC327991	UP|Q8H764_WHEAT (Q8H764) Zeaxanthin epoxidase, 82%	-0.01	0.01
	Auxin	13537	TC371167	UP|IAA3_ORYSA (Q5NB25) Auxin-responsive protein IAA3, 89%		0.01
		15109	TC332896	GB|AAD32773.1|4895186|AC007661 axi 1-like protein {Arabidopsis thaliana}, 29%		-0.02
		460	TC354000	UP|PIN1C_ORYSA (Q67UL3) Probable auxin efflux carrier component 1c (OsPIN1c), 13%		0.01
		8442	TC363330	GB|AAA33436.1|168422|MZEAUX auxin-binding protein precursor {Zea mays}, 82%	-0.01	
		8867	TC361801	UP|Q8S980_ORYSA (Q8S980) Auxin response factor 6b, 48%	-0.01	
		10948	TC327734	UP|Q8GST0_ORYSA (Q8GST0) Auxin response factor 1, 62%		0.01
		2014	TC320512		-0.01	
	Brassinosteroid.signal transduction	7935	TC346307	UP|Q942F3_ORYSA (Q942F3) Extra sporogenous cells-like, 45%		0.01
		188	TC353475	RF|XP_507025.1|51964480|XM_507025 P0700F06.34-2 gene product {Oryza sativa (japonica cultivar-group)}, 21%		-0.01
		12328	TC323536	Zea mays clone EL01N0532H06.c mRNA sequence		0.01
		3955	TC354772	UP|C90D2_ORYSA (Q94IW5) Cytochrome P450 90D2 (C6-oxidase) , 24%		0.01
		11297	TC327643	UP|Q5YFA2_MAIZE (Q5YFA2) Brassinosteroid biosynthesis-like protein	-0.01	
		320	TC316709	Zea mays clone Contig854.F mRNA sequence		-0.01
		13033	TC337966	Zea mays clone Contig708.F mRNA sequence	0.01	
		12534	-	-		0.01
		1256	TC317861	Zea mays clone EL01N0563E07.d mRNA sequence		0.01
		4164	TC318300	UP|SMT2_ORYSA (O82427) 24-methylenesterol C-methyltransferase 2		0.01

	Cytokinin	8068	-	-		-0.01
	Ethylene	2350	TC345632	UP|Q9LXT3_ARATH (Q9LXT3) Transcriptional coactivator-like protein (AT3g58680)		-0.01
		2828	TC328570	UP|Q9FQ93_TOBAC (Q9FQ93) Anther ethylene-upregulated protein ER1, 50%	-0.01	
		12668	TC317123	UP|Q5MFV1_ORYSA (Q5MFV1) BTH-induced ERF transcriptional factor 3, 55%		0.01
	Gibberelin	2105	TC327992	UP|Q38939_ARATH (Q38939) GASA5, 37%	-0.01	
		2178	TC339892	RF|XP_507233.1|51964898|XM_507233 P0433E10.28 gene product {Oryza sativa (japonica cultivar-group)}, 10%	-0.01	
		12654	TC339081	UP|GAOX2_ORYSA (Q8RVF5) Gibberellin 20 oxidase 2, 40%	-0.01	
	Jasmonate	5171	TC318590	UP|Q6RW09_MAIZE (Q6RW09) Allene oxide cyclase		0.01
Lipid metabolism	Exotics' (steroids, squalene etc).cycloartenol synthase	259	TC341351	UP|Q6IWA6_9POAL (Q6IWA6) Cycloartenol synthase, 17%	-0.01	
		1256	TC317861	Zea mays clone EL01N0563E07.d mRNA sequence		0.01
		9248	TC323725	UP|Q9ZTU8_WHEAT (Q9ZTU8) S276, 95%		0.01
		4034	TC333317	UP|O22106_MAIZE (O22106) Squalene synthase	-0.01	
	Fatty acid desaturation	5032	TC328081	UP|FAD6C_BRANA (P48627) Omega-6 fatty acid desaturase, chloroplast precursor, 82%		0.01
	FA synthesis and FA elongation	12542	TC325121	UP|Q2VA66_SOYBN (Q2VA66) Malonyltransferase, 91%		-0.02
		3318	TC357816	Zea mays clone cr1n.pk0042.b5, mRNA sequence	0.01	
		2140	-	-	-0.01	
		8510	TC328449	UP|Q41765_MAIZE (Q41765) Acyl carrier protein, 96%		0.01
		14304	TC333357	UP|Q41765_MAIZE (Q41765) Acyl carrier protein, 96%		-0.02
		1376	TC340273	UP|Q8RVT5_PANGI (Q8RVT5) Acyl-CoA-binding protein		-0.01
		3068	TC354079	UP|Q8RVT5_PANGI (Q8RVT5) Acyl-CoA-binding protein		-0.01

	6138	-	-		0.01
	6680	TC340273	UP|Q8RVT5_PANGI (Q8RVT5) Acyl-CoA-binding protein	0.01	
	6939	TC327213	GB|AAA39391.1|387397|MUSKTEPI2 epidermal keratin subunit I {Mus musculus}, 6%	-0.01	-0.01
	11258	-	-		0.01
	439	TC365959	UP|Q2RAK2_ORYSA (Q2RAK2) Pyruvate kinase		-0.02
	4444	TC331051	RF|XP_506198.1|51963302|XM_506198 OJ1014_E09.29 gene product {Oryza sativa (japonica cultivar-group)}	-0.01	
	11538	TC367406	Zea mays clone EL01N0406B10.c mRNA sequence		-0.02
Glyceral metabolism	14271	TC357906	UP|GPDA_CUPLA (P52425) Glycerol-3-phosphate dehydrogenase [NAD+], 22%		-0.01
	210	TC371994			-0.01
Lipid degradation	10712	TC339190	UP|Q9LKJ1_ARATH (Q9LKJ1) CoA-thioester hydrolase CHY1 (3-hydroxyisobutyryl-coenzyme A hydrolase), 70%		0.01
	2268	TC327209	UP|Q9LK08_ARATH (Q9LK08) 3-hydroxyisobutyryl-coenzyme A hydrolase-like protein, 77%	0.02	
	2899	TC353305	UP|Q56XU5_ARATH (Q56XU5) 3-hydroxyisobutyryl-coenzyme A hydrolase (At1g06550), 87%	-0.01	
	6870	TC338688	UP|O04469_ARATH (O04469) F5I14.5 protein (At1g65520), 58%		0.01
	7104	TC332737	GB|AAL15341.1|16323214|AY057711 At2g47630/F17A22.2 {Arabidopsis thaliana}, 69%	0.01	0.01
	3256	-	-		0.01
	5625	TC362864	UP|Q5U7K9_9POAL (Q5U7K9) Stem-specific protein, 61%		0.01
	12085	TC336141	UP|Q69SU0_ORYSA (Q69SU0) Inositol 5-phosphatase 3-like protein, 29%		-0.01
	527	TC353798	UP|Q8SAG7_ORYSA (Q8SAG7) Phospholipase D beta 2, 14%		-0.02
Lipid transfer proteins etc	7072	TC321032	UP|Q2PCD1_WHEAT (Q2PCD1) Type 1 non specific lipid transfer protein precursor, 95%		0.01
	9716	TC321032	UP|Q2PCD1_WHEAT (Q2PCD1) Type 1 non specific lipid transfer protein precursor, 95%		-0.01

		14642	TC321032	UP|Q2PCD1_WHEAT (Q2PCD1) Type 1 non specific lipid transfer protein precursor, 95%		0.01
	Phospholipid synthesis	5051	TC345475	UP|O82359_ARATH (O82359) Expressed protein, 34%		0.01
		13300	TC342051	UP|Q6QA26_ORYSA (Q6QA26) Phosphoethanolamine N-methyltransferase, 21%	0.01	
		2403	TC337701	GB|AAD29709.2|71164865|AF140496 cholinephosphate cytidylyltransferase {Oryza sativa (japonica cultivar-group)}, 57%	-0.01	
	TAG synthesis	6370	TC336216	UP|Q5I396_ORYSA (Q5I396) Diacylglycerol acyltransferase, 12%		0.02
Major CHO metabolism	Degradation	12873	TC330885	UP|TPT_MAIZE (P49133) Triose phosphate/phosphate translocator, chloroplast precursor (cTPT), 33%	0.01	0.01
		6585	TC351418	UP|Q2KNB4_ORYSA (Q2KNB4) Hexokinase 8, 88%	-0.01	
		1847	TC363131	UP|SUS1_MAIZE (P04712) Sucrose synthase 1		-0.01
		2748	TC338178	UP|SUS1_MAIZE (P04712) Sucrose synthase 1, 39%		0.01
		4519	TC363131	UP|SUS1_MAIZE (P04712) Sucrose synthase 1	-0.02	
		5360	TC315871	UP|SUS2_MAIZE (P49036) Sucrose synthase 2	-0.01	
	Synthesis	214	TC315909	UP|GLGL2_MAIZE (P55234) Glucose-1-phosphate adenylyltransferase large subunit 2, chloroplast precursor, 98%	-0.01	
		7902	TC316411	UP|Q941P2_MAIZE (Q941P2) ADP-glucose pyrophosphorylase small subunit, 52%		0.01
		10875	TC315909	UP|GLGL2_MAIZE (P55234) Glucose-1-phosphate adenylyltransferase large subunit 2, chloroplast precursor, 98%		-0.01
		6896	TC317834	UP|ADT_ORYSA (P31691) ADP,ATP carrier protein, mitochondrial precursor, 82%		0.01
		9266	TC361986	UP|ADT1_MAIZE (P04709) ADP,ATP carrier protein 1, mitochondrial precursor	0.01	
		12066	TC361986	UP|ADT1_MAIZE (P04709) ADP,ATP carrier protein 1, mitochondrial precursor		-0.01
Metal handling	Binding, chelation and storage	18	TC357882	UP|Q5U7K6_9POAL (Q5U7K6) Metallothionein-like protein, 98%		-0.01
		516	TC357882	UP|Q5U7K6_9POAL (Q5U7K6) Metallothionein-like protein, 98%		-0.01

		776	TC340070	UP|Q6H759_ORYSA (Q6H759) Copper chaperone homolog CCH, 78%	-0.01	
		1787	TC357882	UP|Q5U7K6_9POAL (Q5U7K6) Metallothionein-like protein, 98%	-0.02	
		2328	TC316627	UP|FRI1_MAIZE (P29036) Ferritin-1, chloroplast precursor (ZmFer1)	0.01	
		2436	TC357882	UP|Q5U7K6_9POAL (Q5U7K6) Metallothionein-like protein, 98%		-0.01
		2513	TC357882	UP|Q5U7K6_9POAL (Q5U7K6) Metallothionein-like protein, 98%	-0.01	
		5819	TC357882	UP|Q5U7K6_9POAL (Q5U7K6) Metallothionein-like protein, 98%		-0.01
		5926	TC351419	UP|Q6J338_9ROSI (Q6J338) Copper chaperone, 84%		0.01
		7103	TC365565	UP|METK_ORYSA (P46611) S-adenosylmethionine synthetase 1	0.01	
		10831	TC357882	UP|Q5U7K6_9POAL (Q5U7K6) Metallothionein-like protein, 98%		-0.01
	Regulation	1000	TC350429	UP|ARD2_ORYSA (Q58FK4) 1, 2-dihydroxy-3-keto-5-methylthiopentene dioxygenase 2		-0.01
Minor CHO metabolism	Callose	9850	-	-		-0.02
	Myo-inositol	1679	TC336571	GB|AAG17824.1|10444261|AF289633 inositol polyphosphate 5-phosphatase I {Arabidopsis thaliana}, 37%	-0.01	
	Others	758	TC316437	UP|Q5SMZ1_ORYSA (Q5SMZ1) Aldose 1-epimerase-like, 97%	-0.01	0.01
		956	TC319785	RF|XP_506697.1|51963828|XM_506697 P0575F10.14 gene product {Oryza sativa (japonica cultivar-group)}, 82%	-0.01	-0.01
		6082	TC330920	UP|Q657Z1_ORYSA (Q657Z1) Carbohydrate kinase-like, 92%		0.01
		7854	TC315959	Zea mays clone Contig515 mRNA sequence	0.01	
		8884	TC320523	UP|Q5SN59_ORYSA (Q5SN59) Ribokinase-like, 88%		0.01
		9875	TC349821	RF|NP_974931.1|42573670|NM_203202 catalytic/ hydrolase/ phosphoglycolate phosphatase {Arabidopsis thaliana}, 24%	0.01	
		11285	TC333922	UP|Q657Z1_ORYSA (Q657Z1) Carbohydrate kinase-like, 61%		-0.03
	Trehalose	899	TC344391	UP|Q1W5S5_PENAM (Q1W5S5) Ramosa 3, 98%		-0.01

		13405	TC326631	UP|Q1W5S7_MAIZE (Q1W5S7) Sister of ramosa 3	-0.01	
		641	TC346233	UP|Q9XEY7_SOYBN (Q9XEY7) Trehalase 1 GMTRE1, 51%		-0.01
Misc	Other Ferredoxins and Rieske domain	9498	TC327201	UP|FER3_MAIZE (P27788) Ferredoxin-3, chloroplast precursor , 64%	0.02	
	Acid and other phosphatases	1602	-	-	-0.01	
		4370	TC318380	UP|Q6J5M7_SOLTU (Q6J5M7) Purple acid phosphatase 1, 84%		0.01
		11908	TC365709		0.01	
	Alcohol dehydrogenases	6744	TC330451	UP|ADHX_MAIZE (P93629) Alcohol dehydrogenase class 3	0.01	
	Calcineurin-like phosphoesterase family protein	9043	TC334862	UP|Q3EAN1_ARATH (Q3EAN1) Protein At3g47810, 77%	0.01	
	Cytochrome P450	885	TC320192	UP|C78A1_MAIZE (P48420) Cytochrome P450 78A1		-0.01
		10126	TC356473	UP|C71C2_MAIZE (Q43255) Cytochrome P450 71C2 (Benzoxazineless 3)		0.01
		10247	TC361980	UP|Q5QIS9_HORLE (Q5QIS9) Bx2-like protein, 14%	0.01	
		10853	TC318677	RF|XP_507177.1|51964786|XM_507177 OSJNBb0070J06.25 gene product {Oryza sativa (japonica cultivar-group)}	-0.01	
	Dynamin	4549	TC340358	Zea mays clone Contig726 mRNA sequence		0.01
	GCN5-related N-acetyltransferase	4877	TC328698	Zea mays clone EK07D2304B01.c mRNA sequence		-0.01
	GDSL-motif lipase	4294	TC347899	UP|Q8RZ61_ORYSA (Q8RZ61) Lipase-like, 50%		0.01
		4304	TC340462	UP|Q5ZBI0_ORYSA (Q5ZBI0) Lanatoside 15'-O-acetylesterase-like, 86%	0.02	
		5081	TC331972	UP|ENAH_MOUSE (Q03173) Protein enabled homolog, 3%		0.01
		5093	TC324147	RF|XP_507548.1|51979502|XM_507548 P0643F09.14 gene product {Oryza sativa (japonica cultivar-group)}, 92%	-0.02	

	5909	TC321327	UP|Q69Y47_ORYSA (Q69Y47) EREBP-like protein, 62%		0.01
Gluco-, galacto- and mannosidases	2775	TC329285	RF|XP_469436.1|50918079|XM_469436 beta-glucosidase (with alternative splicing) {Oryza sativa (japonica cultivar-group)}, 44%		0.01
	2902	TC354379	UP|Q8L3P5_ORYSA (Q8L3P5) Beta-galactosidase, 52%	-0.01	
	6286	TC357953	UP|Q68UW0_PYRCO (Q68UW0) Beta-galactosidase, 14%		0.01
	8778	TC354379	UP|Q8L3P5_ORYSA (Q8L3P5) Beta-galactosidase, 52%		0.02
	12718	TC349064	UP|Q5K3Q1_TRIMO (Q5K3Q1) Beta-galactosidase, 18%		0.01
Glutathione S transferases	2635	TC336687	PRF|1303351A|225458|1303351A transferase,glutathione S. {Zea mays}		0.01
	4490	TC317007	UP|Q9FQC9_MAIZE (Q9FQC9) Glutathione S-transferase GST 10	-0.02	-0.01
	5138	TC336687	PRF|1303351A|225458|1303351A transferase,glutathione S. {Zea mays}	0.01	
	5290	TC336687	PRF|1303351A|225458|1303351A transferase,glutathione S. {Zea mays}		0.01
	5338	TC336687	PRF|1303351A|225458|1303351A transferase,glutathione S. {Zea mays}		0.01
	5637	TC316394	UP|Q9FQA9_MAIZE (Q9FQA9) Glutathione S-transferase GST 30	-0.01	
	6099	TC316394	UP|Q9FQA9_MAIZE (Q9FQA9) Glutathione S-transferase GST 30		0.01
	8078	TC322201	PRF|1303351A|225458|1303351A transferase,glutathione S. {Zea mays}	0.01	
	9040	TC327151	UP|Q9FQC1_MAIZE (Q9FQC1) Glutathione S-transferase GST 18, 93%		0.01
	9588	TC336687	PRF|1303351A|225458|1303351A transferase,glutathione S. {Zea mays}	0.01	
	12665	TC341967	UP|Q9FQC2_MAIZE (Q9FQC2) Glutathione S-transferase GST 17, 89%	-0.01	
Invertase/pectin methylesterase inhibitor family protein	3103	TC325396	UP|Q2IIH7_ANADE (Q2IIH7) PE-PGRS family protein, 4%		0.01
Myrosinases-lectin-	1010	TC318654	UP|Q9LL87_MAIZE (Q9LL87) Beta-glucosidase aggregating factor, 13%		-0.01

jacalin	2292	TC368374	GB|AAA18780.1|488466|MMU08339 GDF7 {Mus musculus}, 15%	-0.01	
	2805	TC318654	UP|Q9LL87_MAIZE (Q9LL87) Beta-glucosidase aggregating factor, 13%		-0.01
	5713	TC318654	UP|Q9LL87_MAIZE (Q9LL87) Beta-glucosidase aggregating factor, 13%		0.01
	9096	TC344912	UP|Q2QWD7_ORYSA (Q2QWD7) Jacalin homolog, 17%		0.01
Nitrilases, *nitrile lyases, berberine bridge enzymes, reticuline oxidases, troponine reductases	8452	TC317535	UP|Q3E9P0_ARATH (Q3E9P0) Protein At4g38220, 62%	-0.01	0.01
O- methyl transferases	9654	TC317094	UP|Q9SU94_ARATH (Q9SU94) Arginine methyltransferase, 83%		-0.01
Oxidases - copper, flavone etc.	1782	TC343761	RF|NP_566838.1|18405808|NM_113768 oxidoreductase, acting on paired donors, with incorporation or reduction of molecular oxygen, 2-oxoglutarate as one donor, and incorporation of one atom each of oxygen into both donors {Arabidopsis thaliana}, 78%	-0.02	
	8058	TC343761	RF|NP_566838.1|18405808|NM_113768 oxidoreductase, acting on paired donors, with incorporation or reduction of molecular oxygen, 2-oxoglutarate as one donor, and incorporation of one atom each of oxygen into both donors {Arabidopsis thaliana}, 78%		-0.02
	14909	TC343761	RF|NP_566838.1|18405808|NM_113768 oxidoreductase, acting on paired donors, with incorporation or reduction of molecular oxygen, 2-oxoglutarate as one donor, and incorporation of one atom each of oxygen into both donors {Arabidopsis thaliana}, 78%	0.01	
Protease inhibitor/seed storage/lipid transfer protein (LTP) family protein	192	TC332821	RF|NP_199660.1|15238989|NM_124225 lipid binding {Arabidopsis thaliana}, 45%		-0.01
	1999	TC333824	UP|O24556_MAIZE (O24556) Physical impedance induced protein		0.01
	4374	TC340827	Zea mays clone Contig198 mRNA sequence		0.01
	4695	TC334146	UP|Q40721_ORYSA (Q40721) RCc3 protein, 95%		0.01
	5147	TC352860	Zea mays clone Contig570.F mRNA sequence		0.01
	6639	TC318884	UP|Q9ST25_MAIZE (Q9ST25) ZmGR1b protein		0.01
	8454	TC324670	UP|Q43522_LYCES (Q43522) Tfm5 protein, 66%	-0.01	

		10242	TC341209	UP|O24556_MAIZE (O24556) Physical impedance induced protein, 98%	0.01	
		11759	TC332821	RF|NP_199660.1|15238989|NM_124225 lipid binding {Arabidopsis thaliana}, 45%		-0.01
	Short chain dehydrogenase/reductase (SDR)	5826	TC326786	UP|Q7QJE4_ANOGA (Q7QJE4) ENSANGP00000019038, 64%		0.01
		12692	TC319584	UP|DECR2_ARATH (Q9LTV6) Peroxisomal 2, 4-dienoyl-CoA reductase	0.01	
	UDP glucosyl and glucoronyl transferases	2640	TC321566	UP|Q652K2_ORYSA (Q652K2) Glycosyl transferase family 8 protein-like, 72%		0.01
		6201	TC371225	UP|P89473_HHV2 (P89473) RL2 protein, 3%		0.01
		12938	TC325786	UP|Q84SM6_ORYSA (Q84SM6) Exostosin family protein-like protein, 46%		0.01
		13740	TC346610			0.01
		14393	TC350659	RF|NP_193724.1|15235222|NM_118109 transferase, transferring glycosyl groups {Arabidopsis thaliana}, 7%	0.01	
Mitochondrial electron transport / ATP synthesis	Cytochrome c	3285	TC336065	UP|CYC_MAIZE (P00056) Cytochrome c		-0.01
		14193	TC345346	UP|O50041_CHLRE (O50041) CCS1 protein (C-type cytochrome synthesis 1), 19%	0.01	
		1713	TC319230	UP|Q37697_MAIZE (Q37697) Cytochrome oxidase subunit II	-0.01	
		3275	TC320634	UP|Q9SXV0_ORYSA (Q9SXV0) Cytochrome c oxidase subunit 6b-1 (Cytochrome c oxidase subunit 6b), 77%		0.01
		5941	TC356890	UP|SERB_ARATH (O82796) Phosphoserine phosphatase, chloroplast precursorase, 70%	0.01	
		9823	TC333230	RF|NP_172964.1|15218058|NM_101380 ubiquinol-cytochrome-c reductase {Arabidopsis thaliana}, 97%	0.01	
		10765	TC350900	RF|NP_172964.1|15218058|NM_101380 ubiquinol-cytochrome-c reductase {Arabidopsis thaliana}	-0.01	
	F1-ATPase	172	TC317181	Zea mays clone EL01N0523B01.d mRNA sequence		-0.01
		677	TC342196	UP|ATP5E_MAIZE (Q41898) ATP synthase epsilon chain, mitochondrial	-0.01	
		2134	TC316278	UP|ATPG3_IPOBA (P26360) ATP synthase gamma chain, mitochondrial precursor, 91%	-0.01	

	4306	TC349643	UP|Q3H3B3_9ACTO (Q3H3B3) Binding-protein-dependent transport systems inner membrane component precursor, 5%		0.01
	4997	TC316326	Zea mays clone Contig504 mRNA sequence		0.01
	5528	TC319963	UP|ATP4_IPOBA (Q40089) ATP synthase delta' chain, mitochondrial precursor, 88%		0.01
	6947	TC317812	UP|ATP4_IPOBA (Q40089) ATP synthase delta' chain, mitochondrial precursor, 88%		-0.02
NADH-DH	730	TC323979	UP|NUIM_SOLTU (P80269) NADH-ubiquinone oxidoreductase 23 kDa subunit, mitochondrial precursor, 78%	-0.01	
	884	TC363604	RF|XP_506513.1|51963608|XM_506513 P0503D09.102 gene product {Oryza sativa (japonica cultivar-group)}, 58%		-0.01
	1051	TC315950	RF|XP_506513.1|51963608|XM_506513 P0503D09.102 gene product {Oryza sativa (japonica cultivar-group)}		-0.01
	1395	TC366082	Zea mays clone EL01N0305F07.d mRNA sequence		-0.01
	3817	TC363891	UP|Q8LGE7_ARATH (Q8LGE7) NADH:ubiquinone oxidoreductase-like protein (At5g18800), 94%		0.01
	4326	TC346729	UP|Q9FIJ2_ARATH (Q9FIJ2) NADH dehydrogenase 10.5K chain-like protein, 92%		0.01
	4472	TC338452	UP|Q69WE3_ORYSA (Q69WE3) NADH-ubiquinone oxidoreductase-related-like protein	-0.01	
	6018	TC322006	UP|NUFM_ARATH (Q9FLX7) Probable NADH-ubiquinone oxidoreductase 18 kDa subunit, mitochondrial precursor, 79%	-0.02	
	6101	TC315950	RF|XP_506513.1|51963608|XM_506513 P0503D09.102 gene product {Oryza sativa (japonica cultivar-group)}		0.01
	6137	TC315950	RF|XP_506513.1|51963608|XM_506513 P0503D09.102 gene product {Oryza sativa (japonica cultivar-group)}		0.01
	6872	TC335018	UP|Q7XBW1_ORYSA (Q7XBW1) Expressed protein, 98%		0.01
	8860	TC330236	RF|XP_506513.1|51963608|XM_506513 P0503D09.102 gene product {Oryza sativa (japonica cultivar-group)}, 53%	0.01	
	11307	TC357528	UP|N7BM_ARATH (Q9M9M9) Probable NADH-ubiquinone oxidoreductase subunit B17.2, 92%	0.01	

		12057	TC363891	UP|Q8LGE7_ARATH (Q8LGE7) NADH:ubiquinone oxidoreductase-like protein (At5g18800), 94%		0.02
		14389	TC319656	UP|NUFM_ARATH (Q9FLX7) Probable NADH-ubiquinone oxidoreductase 18 kDa subunit, mitochondrial precursor, 79%	0.01	
Nitrogen-metabolism	Ammonia metabolism	3746	TC362143	UP|Q9ZNX7_ORYSA (Q9ZNX7) NADH dependent Glutamate Synthase precursor, 12%	-0.01	
		690	TC316759	GB|BAA03431.1|286124|MZEGS1B glutamine synthetase {Zea mays}		0.02
		5882	TC317023	UP|GLNA2_MAIZE (P38560) Glutamine synthetase root isozyme 2 (Glutamate--ammonia ligase)		0.01
	Misc	1193	TC357958	RF|NP_190533.1|15229183|NM_114824 FAD binding {Arabidopsis thaliana}, 30%		-0.02
Nucleotide metabolism		2876	TC343678	UP|Q84VV6_ARATH (Q84VV6) At4g16566 (MRNA, complete cds, clone: RAFL21-63-F01) (MRNA, complete cds, clone: RAFL23-10-N20), 70%		0.01
	Degradation	611	TC344846	RF|NP_563745.1|18390550|NM_100442 hydrolase {Arabidopsis thaliana}, 56%		-0.01
		14602	TC341275		0.01	
	Deoxynucleotide metabolism	5263	TC323824	GB|CAB51171.1|5541665|ATT6H20 dUTP pyrophosphatase-like protein {Arabidopsis thaliana} , 85%		0.01
		1223	TC329886	Zea mays clone EL01N0512A06.d mRNA sequence		0.01
		2816	TC329886	Zea mays clone EL01N0512A06.d mRNA sequence	-0.01	
		10569	TC332262	Zea mays clone EL01N0422F11.c mRNA sequence		0.01
	Phosphotransfer and pyrophosphatases	11627	TC351034	Zea mays clone EL01N0519H11.c mRNA sequence		-0.01
		2678	TC346988	UP|IPYR_MAIZE (O48556) Soluble inorganic pyrophosphatase (Pyrophosphate phospho-hydrolase) (PPase)	-0.01	
		4167	TC357469	UP|Q2P9V0_9MAGN (Q2P9V0) Soluble inorganic pyrophosphatase, 83%		0.01
		4219	TC367931	UP|IPYR_MAIZE (O48556) Soluble inorganic pyrophosphatase (Pyrophosphate phospho-hydrolase) (PPase)	-0.01	0.01
		829	TC332430	UP|NDK1_SACOF (P93554) Nucleoside diphosphate kinase 1		-0.02

		8908	TC360950	PDB|1PKU_A|61679782|1PKU_A Chain A, Crystal Structure Of Nucleoside Diphosphate Kinase From Rice. {Oryza sativa}		0.01
		2000	TC316766	UP|Q9SE48_ORYSA (Q9SE48) UMP/CMP kinase a, 95%		0.01
		3489	TC333171	UP|Q9SE48_ORYSA (Q9SE48) UMP/CMP kinase a, 94%		-0.01
		11309	TC316766	UP|Q9SE48_ORYSA (Q9SE48) UMP/CMP kinase a, 95%		-0.01
	Salvage	8866	TC318622	UP|Q71F76_LYCES (Q71F76) DCK/dGK-like deoxyribonucleoside kinase, 70%	-0.01	
		12770	TC350075	UP|UPP_TOBAC (P93394) Uracil phosphoribosyltransferase, 94%		0.01
	Synthesis	11979	TC341041	UP|KPRS3_ORYSA (Q8S2E5) Ribose-phosphate pyrophosphokinase 3, 26%		-0.01
		745	TC329593	UP|PYRC_ARATH (O04904) Dihydroorotase, mitochondrial precursor, 90%		-0.01
		11887	TC329593	UP|PYRC_ARATH (O04904) Dihydroorotase, mitochondrial precursor, 90%	-0.01	
		8088	TC317371	UP|Q9LKI4_MAIZE (Q9LKI4) UMP synthase		0.01
OPP	Electron transfer	9498	TC327201	UP|FER3_MAIZE (P27788) Ferredoxin-3, chloroplast precursor , 64%	0.02	
	Oxidative PP	8553	TC315976	UP|Q7FRX8_ORYSA (Q7FRX8) Cytosolic 6-phosphogluconate dehydrogenase, 98%		-0.01
		4426	TC316575	RF|XP_506916.1|51964262|XM_506916 OSJNBb0059G13.23 gene product {Oryza sativa (japonica cultivar-group)}, 86%	-0.01	
		5428	TC336517	UP|Q9LRI9_WHEAT (Q9LRI9) Glucose-6-phosphate dehydrogenase, 31%	0.01	
		9196	TC339413	UP|Q7X7I6_ORYSA (Q7X7I6) Glucose-6-phosphate 1-dehydrogenase (G6PD) , 34%	-0.01	
Polyamine metabolism	Degradation	8219	TC317890	UP|PAO_MAIZE (O64411) Polyamine oxidase precursor		0.01
	Synthesis	6979	TC368082	UP|DCAM_MAIZE (O24575) S-adenosylmethionine decarboxylase proenzyme		0.01
		7055	TC327880	UP|DCAM_MAIZE (O24575) S-adenosylmethionine decarboxylase proenzyme , 38%		0.02
		10530	TC368082	UP|DCAM_MAIZE (O24575) S-adenosylmethionine decarboxylase proenzyme		0.01
		12195	TC368082	UP|DCAM_MAIZE (O24575) S-adenosylmethionine decarboxylase proenzyme		0.02

		7700	TC321207	UP|SPD1_ORYSA (Q9SMB1) Spermidine synthase 1		-0.01
Protein	Assembly and cofactor ligation	5128	TC369755			0.01
		6769	TC324362	RF|NP_187678.1|15228351|NM_111903 ATNAP7 {Arabidopsis thaliana}, 65%		-0.01
		8479	TC321214	GB|BAC76603.1|30698492|AB096013 NifU1 {Oryza sativa (japonica cultivar-group)}, 73%	-0.01	
		12499	TC321813	UP|Q8L984_ARATH (Q8L984) NifU-like protein, 73%	0.02	
	Amino acid activation	5784	TC340968	RF|NP_180591.1|15227735|NM_128585 pseudouridylate synthase/ tRNA-pseudouridine synthase {Arabidopsis thaliana} , 44%		0.02
		9293	TC340968	RF|NP_180591.1|15227735|NM_128585 pseudouridylate synthase/ tRNA-pseudouridine synthase {Arabidopsis thaliana} , 44%		0.02
		14448	TC337529	UP|Q41754_MAIZE (Q41754) Ubiquitin, 34%	0.01	
		2478	TC318466	RF|NP_172433.2|79339972|NM_100834 ATP binding {Arabidopsis thaliana}, 59%	-0.01	
		756	TC320394	UP|Q7DLG4_ARATH (Q7DLG4) Arginyl-tRNA synthetase, 35%	-0.01	
		5736	TC329407	RF|XP_507519.1|51979332|XM_507519 OJ1717_A09.34 gene product {Oryza sativa (japonica cultivar-group)}, 31%		0.02
		3409	-	-	0.02	-0.01
		5834	TC337449	UP|Q8SA98_MAIZE (Q8SA98) Glycyl-tRNA synthetase, 41%		0.01
		642	TC362054	UP|Q5Z9M1_ORYSA (Q5Z9M1) Methionyl-tRNA synthetase, 16%	-0.01	
		4759	TC337318	UP|Q9ASP8_ARATH (Q9ASP8) AT3g55400/T22E16_60, 7%		0.01
		1223	TC335546	UP|Q9LY83_ARATH (Q9LY83) TRNA synthase-like protein, 26%		0.01
		2816	TC329886	Zea mays clone EL01N0512A06.d mRNA sequence	-0.01	
		5917	TC318593	UP|O82108_MAIZE (O82108) Seryl-tRNA synthetase		0.01
		1049	TC319527	UP|Q8LPC9_ORYSA (Q8LPC9) Threonyl-tRNA synthetase		-0.02
	Degradation	343	TC328083	UP|Q53PA1_ORYSA (Q53PA1) Serine carboxypeptidase, 64%		0.01

1203	TC333592	UP|SUI1_ORYSA (P33278) Protein translation factor SUI1 homolog		-0.01
1411	TC317913	UP|Q6YT00_ORYSA (Q6YT00) Proteasome alpha subunit		-0.01
1416	TC321052	RF|NP_194117.3|42567081|NM_118517 protein binding {Arabidopsis thaliana}, 35%		-0.01
2774	TC353142	UP|Q7GBC8_ORYSA (Q7GBC8) Ubiquitin-related modifier-1, 97%	-0.01	
3730	TC369678	UP|Q945B8_EUPES (Q945B8) Growth-on protein GRO10, 24%	-0.01	
4625	TC339421	RF|NP_172401.2|30681070|NM_100800 metalloexopeptidase/ metallopeptidase/ methionyl aminopeptidase/ peptidase {Arabidopsis thaliana}, 51%		0.01
5750	TC320242	RF|XP_507533.1|51979370|XM_507533 OJ1175_B01.8-1 gene product {Oryza sativa (japonica cultivar-group)}, 56%		0.01
5774	TC348062	UP|UBC12_SCHPO (O74549) NEDD8-conjugating enzyme ubc12, 29%	-0.01	
6599	TC320398	UP|Q8GVF6_ORYSA (Q8GVF6) Pyrrolidone carboxyl peptidase-like protein, 97%		-0.01
7921	TC341113	RF|XP_507338.1|51965108|XM_507338 P0562A06.41 gene product {Oryza sativa (japonica cultivar-group)}, 84%		0.01
8290	-	-		-0.01
8524	TC347585	UP|PSA2_ORYSA (Q9LSU2) Proteasome subunit alpha type 2	0.01	
10191	TC319648	UP|Q5JKW4_ORYSA (Q5JKW4) ATP-dependent protease La (LON) domain-containing protein-like, 69%		0.01
10201	TC319964	UP|Q67V39_ORYSA (Q67V39) Phosphatase-like, 39%		-0.01
10351	TC315930	Zea mays clone Contig452 mRNA sequence	0.01	0.01
11161	TC341342	UP|PSA2_ORYSA (Q9LSU2) Proteasome subunit alpha type 2 , 81%		0.01
11438	TC335376	RF|XP_507579.1|51979665|XM_507579 P0690C12.28 gene product {Oryza sativa (japonica cultivar-group)}	0.01	
12085	TC336141	UP|Q69SU0_ORYSA (Q69SU0) Inositol 5-phosphatase 3-like protein, 29%		-0.01
12509	TC347585	UP|PSA2_ORYSA (Q9LSU2) Proteasome subunit alpha type 2		-0.01

12830	TC328784	GB|AAC83041.1|3834325|F9K20 Strong similarity to gb|AF067141 gamma-glutamyl hydrolase from ESTs gb|R83955, gb|T45062, gb|T22220, gb|AA586207, gb|AI099851 and gb|AI00672 come from this gene. {Arabidopsis thaliana}, 83%	0.01	
14257	TC347585	UP|PSA2_ORYSA (Q9LSU2) Proteasome subunit alpha type 2		-0.01
6942	TC337914			-0.01
7840	TC318502	Zea mays clone EL01N0324E07.c mRNA sequence		0.01
13722	TC348733	Zea mays clone EL01N0523D02.c mRNA sequence	0.02	
4516	TC316076	UP|Q4A190_MAIZE (Q4A190) Beclin 1 protein	-0.01	
2120	TC316118	UP|CYSP1_MAIZE (Q10716) Cysteine proteinase 1 precursor	-0.01	
2703	TC348051	UP|Q9SX19_MAIZE (Q9SX19) Cysteine protease Mir1		0.01
3627	TC316642	UP|Q1XHC6_WHEAT (Q1XHC6) Multidomain cystatin, 93%	-0.01	
4292	TC321186	UP|Q30KW0_ZEAMP (Q30KW0) Cysteine proteinase inhibitor	-0.01	
7820	TC359551	UP|Q655R7_ORYSA (Q655R7) OTU-like cysteine protease-like		0.01
9316	TC318453	UP|Q6Z3T8_ORYSA (Q6Z3T8) Auxin-regulated protein-like, 55%		0.01
9358	TC316642	UP|Q1XHC6_WHEAT (Q1XHC6) Multidomain cystatin, 93%		0.01
9497	-	-		-0.01
12415	TC359551	UP|Q655R7_ORYSA (Q655R7) OTU-like cysteine protease-like		0.01
7904	TC331803	Zea mays clone E04912707E08.c mRNA sequence		0.01
2143	TC342859	UP|CLPAB_LYCES (P31542) ATP-dependent Clp protease ATP-binding subunit clpA homolog CD4B, chloroplast precursor, 19%		-0.01
2415	TC353826	UP|CLPAB_LYCES (P31542) ATP-dependent Clp protease ATP-binding subunit clpA homolog CD4B, chloroplast precursor, 37%	-0.01	
3264	TC318915	GB|AAP49525.1|31376397|BT008763 At1g28110 {Arabidopsis thaliana}, 47%	-0.01	

8536	TC357331	GB|AAP49525.1|31376397|BT008763 At1g28110 {Arabidopsis thaliana}, 54%		0.01
9045	TC328292	UP|CBP3_ORYSA (P37891) Serine carboxypeptidase 3 precursor, 47%		0.01
10534	TC317386	GB|AAP54414.1|31432827|AE016959 expressed protein {Oryza sativa (japonica cultivar-group)}, 87%	-0.01	0.01
10772	TC316373	Zea mays clone EL01N0361F06.c mRNA sequence		0.01
13294	TC332407	UP|Q8LKH2_ORYSA (Q8LKH2) ATP-dependent Clp protease, 93%		0.01
6042	TC365983	RF|NP_565330.1|18396193|NM_126605 peptidase/ subtilase {Arabidopsis thaliana}, 35%	-0.01	0.01
440	TC327815	UP|Q9FGS7_ARATH (Q9FGS7) Protein kinase ATN1-like protein (At5g50180), 37%		-0.01
1680	TC329112	UP|Q6LCT7_MAIZE (Q6LCT7) Ubiquitin fusion protein	-0.01	
2181	TC317790	UP|Q2V986_SOLTU (Q2V986) Ubiquitin-conjugating protein-like, 79%	-0.01	
3250	TC329112	UP|Q6LCT7_MAIZE (Q6LCT7) Ubiquitin fusion protein	-0.01	
4223	TC327935	UP|Q8VY32_MAIZE (Q8VY32) Ubiquitin conjugating enzyme 2	0.02	
4491	TC317790	UP|Q2V986_SOLTU (Q2V986) Ubiquitin-conjugating protein-like, 79%	-0.01	
4856	TC348584	UP|Q6LCT7_MAIZE (Q6LCT7) Ubiquitin fusion protein	-0.01	
7796	TC332420	UP|O82143_ORYSA (O82143) OsS5a (26S proteasome regulatory particle non-ATPase subunit10), 54%		0.01
10559	TC321158	GB|AAB60914.1|2190550|F5I14 ESTs gb|T45673,gb|N37512 come from this gene. {Arabidopsis thaliana}, 91%	-0.01	
11620	TC344991	UP|Q5ZCB9_ORYSA (Q5ZCB9) Ubiquitin-conjugating enzyme-like, 15%		0.01
12032	TC355466	UP|Q6LCT7_MAIZE (Q6LCT7) Ubiquitin fusion protein		0.01
1308	TC338357	GB|CAA48378.1|22658|ATUBCJEA ubiquitin-conjugating enzyme {Arabidopsis thaliana}		-0.01
5959	TC362594	UP|Q5XUV4_WHEAT (Q5XUV4) Ubiquitin-conjugating enzyme, 91%		0.01

6867	TC335249	RF|NP_565834.1|18404032|NM_129165 ubiquitin conjugating enzyme/ ubiquitin-like activating enzyme {Arabidopsis thaliana}, 98%	0.01	
8458	TC326342	UP|Q70I24_NICBE (Q70I24) SUMO E2 conjugating enzyme SCE1		0.02
9638	TC316566	RF|NP_001031939.1|79328676|NM_001036862 ubiquitin conjugating enzyme/ ubiquitin-like activating enzyme {Arabidopsis thaliana}	0.01	
10430	TC316566	RF|NP_001031939.1|79328676|NM_001036862 ubiquitin conjugating enzyme/ ubiquitin-like activating enzyme {Arabidopsis thaliana}	-0.01	
12039	TC343341	UP|O48555_MAIZE (O48555) Ubiquitin conjugating enzyme	-0.01	
12947	TC350852	UP|Q9M551_9ROSI (Q9M551) Polyubiquitin	0.01	
13421	TC318336	UP|O48555_MAIZE (O48555) Ubiquitin conjugating enzyme		0.02
14669	TC330934	RF|NP_565834.1|18404032|NM_129165 ubiquitin conjugating enzyme/ ubiquitin-like activating enzyme {Arabidopsis thaliana}, 98%	0.01	
14900	TC318336	UP|O48555_MAIZE (O48555) Ubiquitin conjugating enzyme		0.01
15059	TC325992	UP|Q8S920_ORYSA (Q8S920) Ubiquitin-conjugating enzyme OsUBC5a		-0.01
15350	TC341572	UP|Q8S920_ORYSA (Q8S920) Ubiquitin-conjugating enzyme OsUBC5a	0.01	
629	TC319185	Zea mays clone Contig551 mRNA sequence		-0.01
4366	TC337100	UP|Q69U52_ORYSA (Q69U52) POZ domain protein family-like, 82%		0.01
645	TC353211	UP|Q7ZTZ6_BRARE (Q7ZTZ6) STIP1 homology and U-box containing protein 1, 33%	0.02	
1149	TC320110	UP|Q7XI08_ORYSA (Q7XI08) Auxin-regulated protein-like protein, 94%	-0.01	
2066	TC363466	UP|Q8VYW5_ARATH (Q8VYW5) AT5g45290/K9E15_7, 23%	-0.01	
4029	TC326909	RF|NP_176574.2|30696917|NM_105064 zinc ion binding {Arabidopsis thaliana}, 49%	-0.01	
4214	TC327047	RF|NP_171642.1|15223384|NM_100017 nucleic acid binding {Arabidopsis thaliana}, 33%		0.02
5024	TC337154			0.02

5421	TC340974	GB|AAP37763.1|30725482|BT008404 At1g04790 {Arabidopsis thaliana}, 6%	0.01	
5932	TC355375	UP|Q9M2V1_ARATH (Q9M2V1) RING finger-like protein (At3g54360/T12E18_50), 67%		0.02
6283	TC341780	UP|Q5NAS8_ORYSA (Q5NAS8) C3H2C3 RING-finger protein-like, 23%		0.01
7509	TC316188	Zea mays clone EL01N0521E02.d mRNA sequence	0.01	0.01
7878	-	-		0.01
8050	-	-		0.01
8883	TC317047	Zea mays clone Contig284 mRNA sequence		-0.01
9944	TC316949	UP|Q75I59_ORYSA (Q75I59) Expressed protein, 64%		-0.02
10358	TC318125	UP|Q6YW60_ORYSA (Q6YW60) Zinc finger (C3HC4-type RING finger) protein-like, 98%	0.01	
11661	TC359015	RF|XP_506706.1|51963846|XM_506706 OJ1007_D04.4-2 gene product {Oryza sativa (japonica cultivar-group)}, 45%	0.01	
12402	TC322618	UP|ATL1D_ARATH (Q8GW38) RING-H2 finger protein ATL1D, 47%		-0.01
15432	TC342407	UP|Q93VN2_ARATH (Q93VN2) At2g14841/At2g14841, 22%		0.01
11333	TC362648	UP|Q5ZC88_ORYSA (Q5ZC88) CUL1, 64%	0.01	
13703	TC318589	UP|Q93VH7_ORYSA (Q93VH7) Cullin-like protein (CUL1), 49%	0.01	
567	-	-		-0.02
2162	TC322500	UP|SMD1_HUMAN (P62314) Small nuclear ribonucleoprotein Sm D1, 11%	-0.01	
6805	TC317247	Zea mays clone Contig385.F mRNA sequence		-0.01
8446	TC361063	RF|XP_506618.1|51963694|XM_506618 P0015C07.29 gene product {Oryza sativa (japonica cultivar-group)}, 37%		0.02
10156	TC322136			-0.01
10553	TC361897	Zea mays clone Contig523.F mRNA sequence		0.01

10651	TC333492	UP|Q2R0L6_ORYSA (Q2R0L6) F-box protein family, AtFBL4, 25%		-0.01
13841	TC316984	UP|Q5QM27_ORYSA (Q5QM27) Chain A, C-Terminal Domain Of Mouse Brain Tubby Protein-like, 93%	0.01	
5122	TC328600	UP|Q2PYP4_ARAHY (Q2PYP4) RING-box protein, 79%		0.01
12493	TC331345	UP|RBX1A_ARATH (Q940X7) RING-box protein 1a, 91%	-0.01	-0.02
10641	TC369780	UP|Q6PL11_ORYSA (Q6PL11) Skp1 protein, 98%	0.01	
12014	TC369780	UP|Q6PL11_ORYSA (Q6PL11) Skp1 protein, 98%		-0.01
12912	TC324371	UP|Q2BBE2_9BACI (Q2BBE2) Drug/metabolite transporter (DMT) superfamily protein, 6%		-0.01
13911	TC369780	UP|Q6PL11_ORYSA (Q6PL11) Skp1 protein, 98%	0.01	0.01
991	TC358772	UP|SEM11_ARATH (Q9XIR8) Probable 26 proteasome complex subunit sem1-1, 92%		-0.01
1411	TC356537	UP|Q6YT00_ORYSA (Q6YT00) Proteasome alpha subunit, 62%		-0.01
1930	TC317267	UP|Q84ZC7_ORYSA (Q84ZC7) Proteasome inhibitor-like protein, 91%	-0.01	
2015	TC331359	Zea mays clone Contig259 mRNA sequence	-0.02	
2186	TC358997	UP|PRS7_ORYSA (Q9FXT9) 26S protease regulatory subunit 7, 61%	-0.01	
3678	TC316420	UP|Q5XUV7_WHEAT (Q5XUV7) 20S proteasome beta 4 subunit, 93%		-0.01
5942	TC325458	UP|Q8W423_ORYSA (Q8W423) 26S proteasome regulatory particle non-ATPase subunit12	0.01	0.01
6005	TC316315	UP|PSB1_ORYSA (O64464) Proteasome subunit beta type 1	-0.01	
6516	TC334384	GB|AAM20712.1|20466790|AY099861 26S proteasome regulatory subunit {Arabidopsis thaliana}, 35%		0.01
8181	TC333080	UP|Q9LST4_ORYSA (Q9LST4) Beta 7 subunit of 20S proteasome, 68%	-0.01	
9370	TC316010	UP|PSD6_ORYSA (Q8W425) 26S proteasome non-ATPase regulatory subunit 6		0.01
9949	TC327419	UP|Q5VRG3_ORYSA (Q5VRG3) Alpha 3 subunit of 20S proteasome	-0.01	-0.01

	10045	TC320614	UP|Q8RWF0_ARATH (Q8RWF0) 26S proteasome subunit-like protein (26S proteasome subunit RPN9a)	0.01	
	10131	TC346913	UP|PSA3_ORYSA (Q9LSU0) Proteasome subunit alpha type 3	0.01	
	10590	TC316420	UP|Q5XUV7_WHEAT (Q5XUV7) 20S proteasome beta 4 subunit, 93%		-0.01
	10648	TC345907	UP|Q8W3M9_ORYSA (Q8W3M9) 26S proteasome regulatory particle triple-A ATPase subunit5b, 54%		-0.01
	11217	TC317822	UP|Q9FER4_MAIZE (Q9FER4) 20S proteasome alpha subunit		-0.01
	11782	TC332520	UP|Q9FXT8_ORYSA (Q9FXT8) 26S proteasome regulatory particle triple-A ATPase subunit4		-0.01
	12022	TC349337	UP|PRS6B_ARATH (Q9SEI4) 26S protease regulatory subunit 6B homolog, 94%		-0.01
	14010	TC337789	Zea mays clone EL01N0508G06.d mRNA sequence		0.01
	14978	TC329850	UP|Q8W422_ORYSA (Q8W422) 26S proteasome regulatory particle triple-A ATPase subunit2b, 45%		-0.02
	63	TC343327	UP|SMT3_ORYSA (P55857) Ubiquitin-like protein SMT3, 93%		-0.01
	2824	TC341370	UP|Q8RZ90_ORYSA (Q8RZ90) Ribosomal protein L18a-like, 81%	-0.01	-0.02
	7936	TC356517	UP|Q3EAA5_ARATH (Q3EAA5) Protein At4g05320, 33%		0.01
	10547	TC334620	UP|Q6K5J0_ORYSA (Q6K5J0) Ubiquitin-like protein, 83%		-0.02
	12414	TC334620	UP|Q6K5J0_ORYSA (Q6K5J0) Ubiquitin-like protein, 83%	-0.01	-0.01
	15265	TC335629	UP|Q5KQC2_ORYSA (Q5KQC2) Polyubiquitin, 82%	0.01	
	6079	TC355523	UP|Q2QQM3_ORYSA (Q2QQM3) Ubiquitin-specific protease 12, 11%	-0.01	
	8757	TC325172	UP|Q9FPT1_ARATH (Q9FPT1) Ubiquitin-specific protease 12, 13%	0.01	
	11892	TC322365	RF|NP_566680.2|42565077|NM_113023 UBP7 (UBIQUITIN-SPECIFIC PROTEASE 7) cysteine-type endopeptidase/ ubiquitin thiolesterase/ ubiquitin-specific protease {Arabidopsis thaliana}, 78%		-0.02
	14282	TC325930	UP|Q9FPS3_ARATH (Q9FPS3) Ubiquitin-specific protease 24, 37%	0.01	0.02

Folding	1169	TC361384	UP|Q7XUX5_ORYSA (Q7XUX5) Peptidyl-prolyl cis-trans isomerase, 25%	-0.01	
	1373	TC336547	UP|Q8H9B2_9ROSI (Q8H9B2) T-complex polypeptide 1		-0.02
	1669	-	-		-0.01
	2895	TC329860	RF|NP_186902.1|15232923|NM_111120 ATP binding {Arabidopsis thaliana}, 66%		-0.02
	3944	TC335732	RF|XP_506305.1|51963400|XM_506305 OSJNBb0062P14.116-1 gene product {Oryza sativa (japonica cultivar-group)}, 95%	0.01	
	4095	TC329860	RF|NP_186902.1|15232923|NM_111120 ATP binding {Arabidopsis thaliana}, 66%	-0.01	
	4499	TC316727	UP|Q53NM9_ORYSA (Q53NM9) DnaK-type molecular chaperone hsp70-rice	-0.02	
	6049	TC318970	Zea mays clone EL01N0556C01.c mRNA sequence		0.01
	6462	TC327373	UP|Q6B4V4_VITVI (Q6B4V4) Chloroplast chaperonin 21, 80%	0.01	
	10349	TC320767	RF|XP_506305.1|51963400|XM_506305 OSJNBb0062P14.116-1 gene product {Oryza sativa (japonica cultivar-group)}, 95%	0.01	
	11234	TC332928	GB|AAB72097.1|2465428|AF021257 32 kDa protein {Hordeum vulgare subsp. vulgare}, 34%	0.01	
	11722	TC336547	UP|Q8H9B2_9ROSI (Q8H9B2) T-complex polypeptide 1	0.01	
Glycosylation	7147	TC331787	Zea mays clone EL01N0324C05.d mRNA sequence	0.01	
Postranslational modification	10	TC323065	UP|Q8S7U0_ORYSA (Q8S7U0) Serine/threonine protein phosphatase PP2A-4 catalytic subunit, 90%		-0.01
	790	TC359878	PRF|2206327A|1587206|2206327A T complex protein. {Cucumis sativus}	-0.02	
	800	TC337864	UP|Q9FQF5_MAIZE (Q9FQF5) Protein kinase CK2 catalytic subunit CK2 alpha-3, 30%	-0.01	
	958	TC341559		-0.01	
	1255	TC326770	UP|Q6PS57_ORYSA (Q6PS57) Cyclin-dependent kinase subunit, 91%	0.02	
	1375	TC334319	UP|Q5SN75_ORYSA (Q5SN75) Protein phosphatase 2C-like, 43%		-0.02
	1536	TC350804	UP|Q41297_9SOLN (Q41297) Protein kinase, 29%		-0.01

1587	TC330433	UP|Q2QQX0_ORYSA (Q2QQX0) Protein kinase KIPK, 20%	-0.01	
1607	TC322951	RF|NP_849370.1|30682312|NM_179039 MHK ATP binding {Arabidopsis thaliana}, 20%		-0.01
2008	TC330951	UP|SAPK8_ORYSA (Q7Y0B9) Serine/threonine-protein kinase SAPK8 (Osmotic stress/abscisic acid-activated protein kinase 8) , 98%	-0.01	
2018	TC320160	UP|Q8GZT8_ARATH (Q8GZT8) PTEN-like protein, 41%		-0.01
2829	TC354054	RF|XP_507389.1|51979024|XM_507389 OJ1003_H02.130 gene product {Oryza sativa (japonica cultivar-group)}, 4%	-0.01	
2994	TC318499	UP|Q67UH1_ORYSA (Q67UH1) Mitochondrial transcription termination factor-like, 86%		0.02
3258	TC318355	UP|Q1WD14_SHEEP (Q1WD14) Polymorphic epithelial mucin, 13%		0.01
4043	TC326636	UP|Q32SG2_MAIZE (Q32SG2) Protein phosphatase 2A regulatory subunit A		0.01
4102	TC319808	UP|O82469_MESCR (O82469) Protein phosphatase-2C, 70%	-0.01	
4585	TC317577	RF|XP_506498.1|51963590|XM_506498 OJ1136_D11.123 gene product {Oryza sativa (japonica cultivar-group)}, 86%		0.02
4901	TC367831	GB|BAB91129.1|20302604|AB084270 Ser/Thr kinase {Arabidopsis thaliana}, 31%	0.01	
4922	TC341559			0.01
5247	TC326787	UP|O24186_ORYSA (O24186) 10 kDa chaperonin	0.01	
5436	TC330419	UP|Q6J2K6_ORYSA (Q6J2K6) BTH-induced protein phosphatase 1, 49%		0.01
5683	TC316351	GB|AAP13377.1|30023688|BT006269 At5g10480 {Arabidopsis thaliana}		0.01
6007	TC345332	UP|Q7GC12_ORYSA (Q7GC12) Shaggy-related protein kinase gamma, 27%		0.01
6502	TC364264	UP|ARF5_ARATH (Q9ZPX1) Probable ADP-ribosylation factor At2g18390, 43%	0.01	
6505	TC316580	UP|Q9SEG2_MAIZE (Q9SEG2) Protein kinase PK4	0.01	
7394	TC347831	RF|NP_171964.1|15219796|NM_100350 ATP binding {Arabidopsis thaliana}, 11%		0.01
7401	TC343389	RF|NP_177573.2|79379990|NM_106093 ATP binding {Arabidopsis thaliana}, 9%		0.01

7684	TC333099	RF|NP_179594.1|15225287|NM_127562 ATP binding {Arabidopsis thaliana}, 24%		0.01
8419	TC336577	UP|Q4R1K7_ORYSA (Q4R1K7) Aurora kinase, 66%	-0.01	
8806	TC339077	UP|Q5Z7K2_ORYSA (Q5Z7K2) Serine/threonine protein phosphatase PP2A-1 catalytic subunit	-0.01	
8875	TC338344	UP|Q7F270_ORYSA (Q7F270) ADP-ribosylation factor 1, 95%		0.01
8980	TC316122	UP|Q9FQF6_MAIZE (Q9FQF6) Protein kinase CK2 regulatory subunit CK2B3		-0.01
9246	TC323257	UP|Q84WJ9_ARATH (Q84WJ9) At5g19680, 93%		0.01
9303	TC322593	GB|AAK73969.1|15010620|AY045611 AT4g08960/T3H13_2 {Arabidopsis thaliana}, 44%		0.01
9339	TC340380	UP|Q94CI5_ARATH (Q94CI5) Protein kinase AtSIK, 47%		0.01
9398	TC316748	UP|Q8LF96_ARATH (Q8LF96) PRL1 protein, 78%	0.01	
9923	TC316511	UP|Q8S7U0_ORYSA (Q8S7U0) Serine/threonine protein phosphatase PP2A-4 catalytic subunit, 90%	0.01	-0.01
10108	TC330244	RF|NP_850057.1|30682545|NM_179726 GTP binding {Arabidopsis thaliana}		-0.01
10512	TC316305	UP|PP1_MAIZE (P22198) Serine/threonine-protein phosphatase PP1		0.01
11687	TC333099	RF|NP_179594.1|15225287|NM_127562 ATP binding {Arabidopsis thaliana}, 24%	0.01	
11753	TC341559		0.01	
11937	TC325980	UP|O23334_ARATH (O23334) Kinase like protein, 73%		0.01
11962	TC356350	UP|Q94KC3_ORYSA (Q94KC3) CaMK1, 69%	-0.01	
12229	TC351011	UP|Q7XUF4_ORYSA (Q7XUF4) OJ991113_30.14 protein, 44%	0.01	
12475	TC354630	UP|Q9LS26_ARATH (Q9LS26) Protein kinase-like protein (At5g46570), 92%		-0.01
12507	TC326770	UP|Q6PS57_ORYSA (Q6PS57) Cyclin-dependent kinase subunit, 91%	-0.01	
12778	TC330850	UP|Q5Z7J0_ORYSA (Q5Z7J0) Shaggy-like kinase etha, 33%		0.01
13789	TC316305	UP|PP1_MAIZE (P22198) Serine/threonine-protein phosphatase PP1	0.01	

	14417	TC317850	UP|PP1_ORYSA (P48489) Serine/threonine-protein phosphatase PP1, 98%		0.01
	15194	TC322582	UP|Q8LK43_WHEAT (Q8LK43) GSK-like kinase		0.01
	15517	TC326419	UP|SAPK4_ORYSA (Q5N942) Serine/threonine-protein kinase SAPK4 (Osmotic stress/abscisic acid-activated protein kinase 4) , 87%		-0.02
	2052	TC324529	RF|NP_192172.1|15235432|NM_116497 ATP binding {Arabidopsis thaliana}, 69%	-0.01	
	12132	TC325152			0.01
	1013	TC347299	RF|NP_173489.1|15217992|NM_101916 ATP binding {Arabidopsis thaliana}, 13%	-0.01	
	12467	TC324525	RF|NP_186779.1|15232085|NM_110996 ATP binding {Arabidopsis thaliana}, 53%	-0.02	
	655	TC331535	RF|NP_182083.1|15225518|NM_130121 ATP binding {Arabidopsis thaliana}, 24%	-0.01	-0.01
Synthesis.elongation	193	TC317846	UP|Q9ASR1_ARATH (Q9ASR1) At1g56070/T6H22_13 (Elongation factor EF-2)		-0.01
	425	TC367625	Zea mays clone EL01N0413C10.c mRNA sequence		-0.02
	910	TC346618	Zea mays clone Contig308 mRNA sequence		-0.01
	1370	TC369772	Zea mays clone Contig257 mRNA sequence		-0.01
	2117	TC324535	Zea mays clone Contig365 mRNA sequence		-0.01
	2171	TC324535	Zea mays clone Contig365 mRNA sequence	-0.01	
	2420	TC336397	UP|EF1A_MAIZE (Q41803) Elongation factor 1-alpha, 45%		-0.01
	2603	TC348770	RF|NP_181390.1|15224901|NM_129413 DNA binding {Arabidopsis thaliana}, 57%		0.01
	2714	TC339495	UP|EF2_BETVU (O23755) Elongation factor 2 (EF-2), 28%		0.01
	3153	TC367625	Zea mays clone EL01N0413C10.c mRNA sequence	-0.01	
	3279	TC336397	UP|EF1A_MAIZE (Q41803) Elongation factor 1-alpha, 45%		-0.01
	3319	TC336397	UP|EF1A_MAIZE (Q41803) Elongation factor 1-alpha, 45%		-0.01
	4404	TC369304	UP|Q9SGT4_ARATH (Q9SGT4) Elongation factor EF-2, 79%	0.01	

	4556	TC369772	Zea mays clone Contig257 mRNA sequence	-0.01	
	4709	TC349287	GB|AAL10483.1|15983773|AY056792 AT4g29060/F19B15_90 {Arabidopsis thaliana}, 4%		0.01
	5662	TC340493	UP|Q9M7E6_MAIZE (Q9M7E6) Elongation factor 1 alpha		0.01
	6169	TC337809	UP|O50018_MAIZE (O50018) Elongation factor 1-alpha		0.01
	6238	TC331966	UP|Q9M7E6_MAIZE (Q9M7E6) Elongation factor 1 alpha, 67%	0.01	
	6387	TC324246	UP|Q9SWW0_LYCES (Q9SWW0) Ethylene-responsive elongation factor EF-Ts precursor, 42%		0.01
	8137	TC364493	GB|AAO72574.1|29367403|AY224455 elongation factor 1 gamma-like protein {Oryza sativa (japonica cultivar-group)}, 60%	0.01	
	10178	TC340493	UP|Q9M7E6_MAIZE (Q9M7E6) Elongation factor 1 alpha		-0.02
	11420	TC346618	Zea mays clone Contig308 mRNA sequence	-0.01	
	11700	TC369772	Zea mays clone Contig257 mRNA sequence		-0.01
	11894	TC364785	GB|BAC16499.1|23237926|AP005198 elongation factor 1 beta {Oryza sativa (japonica cultivar-group)}, 92%	0.02	
	13642	TC316813	Zea mays clone Contig596 mRNA sequence	0.01	0.01
	13954	TC369304	UP|Q9SGT4_ARATH (Q9SGT4) Elongation factor EF-2, 79%		0.01
	14662	TC350451	UP|Q9M7E5_MAIZE (Q9M7E5) Elongation factor 1 alpha	0.01	
Synthesis.initiation	2	TC333592	UP|SUI1_ORYSA (P33278) Protein translation factor SUI1 homolog	-0.01	
	15	TC321218	UP|IF5A_MAIZE (P80639) Eukaryotic translation initiation factor 5A		-0.01
	146	TC318770	UP|SUI1_MAIZE (P56330) Protein translation factor SUI1 homolog		-0.01
	530	TC346232	Zea mays clone Contig468 mRNA sequence		-0.02

574	TC346232	Zea mays clone Contig468 mRNA sequence		-0.02
825	TC321218	UP|IF5A_MAIZE (P80639) Eukaryotic translation initiation factor 5A	-0.01	-0.01
831	TC333592	UP|SUI1_ORYSA (P33278) Protein translation factor SUI1 homolog		-0.01
833	TC320480	UP|Q2QME5_ORYSA (Q2QME5) Translation initiation factor eIF-2 gamma chain F20D22.6		-0.02
901	TC366376	UP|O24449_MAIZE (O24449) Translational initiation factor eIF-4A		-0.01
1203	TC347585	UP|PSA2_ORYSA (Q9LSU2) Proteasome subunit alpha type 2		-0.01
1650	TC318899	UP|IF2B_WHEAT (O24473) Eukaryotic translation initiation factor 2 beta subunit	-0.01	
2092	TC345731	UP|IF1A_WHEAT (P47815) Eukaryotic translation initiation factor 1A	-0.01	
2401	TC345052	UP|Q2LFC3_NICBE (Q2LFC3) AGO1-2, 17%	-0.01	
2543	TC331710	UP|IF1A_WHEAT (P47815) Eukaryotic translation initiation factor 1A	-0.01	0.01
2806	TC333592	UP|SUI1_ORYSA (P33278) Protein translation factor SUI1 homolog		-0.02
2817	TC345731	UP|IF1A_WHEAT (P47815) Eukaryotic translation initiation factor 1A	-0.01	
3369	TC346232	Zea mays clone Contig468 mRNA sequence	0.01	-0.01
4036	TC333592	UP|SUI1_ORYSA (P33278) Protein translation factor SUI1 homolog		-0.01
4039	TC318770	UP|SUI1_MAIZE (P56330) Protein translation factor SUI1 homolog	-0.01	
4143	TC321218	UP|IF5A_MAIZE (P80639) Eukaryotic translation initiation factor 5A		0.01
4611	TC360749	RF|XP_506503.1|51963598|XM_506503 OJ1340_C08.131 gene product {Oryza sativa (japonica cultivar-group)}		0.01
5699	TC333592	UP|SUI1_ORYSA (P33278) Protein translation factor SUI1 homolog		0.01
5781	TC318064	RF|XP_506503.1|51963598|XM_506503 OJ1340_C08.131 gene product {Oryza sativa (japonica cultivar-group)}		0.01
6507	TC346232	Zea mays clone Contig468 mRNA sequence		0.01

	7560	TC316638	Zea mays clone EL01N0554D04.c mRNA sequence		0.01
	8074	TC318064	RF|XP_506503.1|51963598|XM_506503 OJ1340_C08.131 gene product {Oryza sativa (japonica cultivar-group)}		0.01
	8944	TC346232	Zea mays clone Contig468 mRNA sequence	-0.01	
	8968	TC316851	UP|IF4E2_MAIZE (O81482) Eukaryotic translation initiation factor 4E-2		0.01
	10835	TC338372	UP|IF5_MAIZE (P55876) Eukaryotic translation initiation factor 5 (eIF-5), 39%		-0.01
	13801	TC344354	UP|SUI1_MAIZE (P56330) Protein translation factor SUI1 homolog	0.02	
	14121	TC318770	UP|SUI1_MAIZE (P56330) Protein translation factor SUI1 homolog	0.01	
	14650	TC322920	UP|Q9C5Y8_ARATH (Q9C5Y8) Initiation factor 3d, 65%	0.01	
	14914	TC340575	UP|Q41583_WHEAT (Q41583) Initiation factor (Iso)4f p82 subunit, 15%		0.01
	10858	-	-		0.01
Synthesis.misc	3	TC331114		-0.01	
	9	TC330969	UP|RS11_MAIZE (P25460) 40S ribosomal protein S11		-0.01
	25	TC344993	GB|AAP85547.1|32493112|AY323130 ribosomal protein large subunit 13 {Oryza sativa (japonica cultivar-group)}	-0.01	
	32	TC346170	Zea mays clone Contig334 mRNA sequence		-0.01
	38	TC324399	UP|Q9FJA6_ARATH (Q9FJA6) 40S ribosomal protein S3 (AT5g35530/MOK9_14), 88%		-0.01
	46	TC322766	UP|Q5WMY3_ORYSA (Q5WMY3) Cytoplasmic ribosomal protein L18	-0.01	
	65	TC364357	Zea mays clone Contig30.F mRNA sequence		-0.01
	137	TC318175	UP|Q7XY20_WHEAT (Q7XY20) Ribosomal protein L19, 89%		-0.01
	167	TC347888	RF|NP_916542.1|34910390|NM_191653 ribosomal protein L28-like {Oryza sativa (japonica cultivar-group)}, 95%		-0.01
	218	TC346815	GB|BAA02155.1|303853|RICRPL3A ribosomal protein L3 {Oryza sativa (japonica cultivar-group)}	0.01	

323	TC331601	RF|XP_506724.1|51963882|XM_506724 OJ9003_G05.34 gene product {Oryza sativa (japonica cultivar-group)}		-0.01
369	TC359495	RF|XP_506775.1|51963984|XM_506775 OJ1115_D03.49 gene product {Oryza sativa (japonica cultivar-group)}		-0.01
374	TC347034	UP|RL31_PERFR (Q9M573) 60S ribosomal protein L31, 96%	-0.02	
409	TC341226	Zea mays clone cho1c.pk003.k1, mRNA sequence		-0.01
426	TC337820	Zea mays clone EL01N0425H11.c mRNA sequence		-0.01
427	TC324315	UP|RS19_ORYSA (P40978) 40S ribosomal protein S19, 98%		-0.02
434	TC331642	UP|RL7A_ORYSA (P35685) 60S ribosomal protein L7a		-0.02
436	TC326383	UP|Q8S2Y8_MAIZE (Q8S2Y8) Glycine-rich RNA binding protein		-0.02
445	TC350465	UP|Q8GTE2_CICAR (Q8GTE2) Ribosomal protein RL5		-0.01
466	TC347350	RF|XP_507392.1|51979030|XM_507392 B1056G08.113 gene product {Oryza sativa (japonica cultivar-group)}, 72%		-0.01
473	TC330969	UP|RS11_MAIZE (P25460) 40S ribosomal protein S11		-0.01
486	TC337840	UP|Q7XC31_ORYSA (Q7XC31) 60S ribosomal protein L27		-0.01
495	TC332929	UP|RS15D_ARATH (Q9FY64) 40S ribosomal protein S15-4, 98%		-0.02
505	TC368426	UP|Q8W1C9_MAIZE (Q8W1C9) Ribosomal protein L35A		-0.01
552	TC343230	UP|Q9AV77_ORYSA (Q9AV77) 60S ribosomal protein L17		-0.01
554	TC326068	UP|Q9SM26_MAIZE (Q9SM26) Acidic ribosomal protein P2a-2		-0.01
588	TC332529	RF|XP_507485.1|51979244|XM_507485 OJ1126_B06.24 gene product {Oryza sativa (japonica cultivar-group)}	-0.01	-0.01
605	TC363531	UP|Q9AV77_ORYSA (Q9AV77) 60S ribosomal protein L17		-0.01
606	TC362382	UP|RS19_ORYSA (P40978) 40S ribosomal protein S19, 98%		-0.01

701	TC345470	UP|Q7XY20_WHEAT (Q7XY20) Ribosomal protein L19, 73%	0.01	
738	TC362238	UP|Q4GXM3_BIPLU (Q4GXM3) Ribosomal protein L7e, 39%	-0.01	0.01
754	TC316461	UP|Q2R4A1_ORYSA (Q2R4A1) Ribosomal protein S7	-0.01	
804	TC342119	UP|Q8GTE2_CICAR (Q8GTE2) Ribosomal protein RL5		-0.01
811	TC316836	UP|Q6RJY1_CAPAN (Q6RJY1) 60S ribosomal protein L12		-0.02
813	TC329251	UP|Q7XY20_WHEAT (Q7XY20) Ribosomal protein L19, 70%		-0.01
827	TC341226	Zea mays clone cho1c.pk003.k1, mRNA sequence		-0.02
836	TC317715	Zea mays clone cho1c.pk003.i13, mRNA sequence		-0.01
841	TC347350	RF|XP_507392.1|51979030|XM_507392 B1056G08.113 gene product {Oryza sativa (japonica cultivar-group)}, 72%		-0.01
843	TC321886	UP|Q9FSF6_TOBAC (Q9FSF6) Ribosomal protein L11-like, 97%		-0.01
846	TC355817	UP|Q5I7K2_WHEAT (Q5I7K2) Ribosomal protein S7		-0.01
849	TC345147	UP|Q5WMY3_ORYSA (Q5WMY3) Cytoplasmic ribosomal protein L18		-0.01
859	TC341880	UP|Q2R1J8_ORYSA (Q2R1J8) Ribosomal protein S4, 98%		-0.01
864	TC343031	RF|XP_507607.1|51979721|XM_507607 P0562A06.14 gene product {Oryza sativa (japonica cultivar-group)}		-0.01
867	TC323766	UP|Q9FUL7_MAIZE (Q9FUL7) 40S ribosomal protein S24		-0.01
908	TC334634	Zea mays clone Contig717.F mRNA sequence		-0.01
954	TC337820	Zea mays clone EL01N0425H11.c mRNA sequence		-0.01
1011	TC333637	UP|Q8W1C9_MAIZE (Q8W1C9) Ribosomal protein L35A		-0.01
1180	TC338421	RF|XP_507607.1|51979721|XM_507607 P0562A06.14 gene product {Oryza sativa (japonica cultivar-group)}		-0.01
1281	TC343254			-0.02

1288	TC336888	RF|XP_507392.1|51979030|XM_507392 B1056G08.113 gene product {Oryza sativa (japonica cultivar-group)}, 73%	-0.01	-0.01
1323	TC364858	UP|RS8_MAIZE (Q08069) 40S ribosomal protein S8		-0.01
1336	TC367265	RF|XP_506775.1|51963984|XM_506775 OJ1115_D03.49 gene product {Oryza sativa (japonica cultivar-group)}, 89%	0.01	-0.01
1369	TC370785	UP|Q3MST7_ORYSA (Q3MST7) Ribosomal L32		-0.01
1385	TC329253			-0.02
1405	TC363721	UP|Q7XR19_ORYSA (Q7XR19) 60S ribosomal protein L6		-0.01
1420	TC368507	UP|Q6L5M1_BROIN (Q6L5M1) Glycoprotein, 97%	0.01	
1606	TC329095	UP|ZEB2_MAIZE (P08031) Zein-beta precursor (16 kDa)	-0.01	
1623	TC324946	UP|RL7A_ORYSA (P35685) 60S ribosomal protein L7a		-0.01
1627	TC342119	UP|Q8GTE2_CICAR (Q8GTE2) Ribosomal protein RL5		-0.01
1711	TC316271	UP|O22453_MAIZE (O22453) Ribsomal protein S4		-0.01
1773	TC326729	UP|RL111_ARATH (P42795) 60S ribosomal protein L11-1 (L16A), 98%		-0.01
2005	TC330576	UP|Q5I7L3_WHEAT (Q5I7L3) Ribosomal protein L10A		-0.01
2009	TC335247	UP|RL24_HORVU (P50888) 60S ribosomal protein L24	-0.01	
2019	TC361544	UP|Q5I7L5_WHEAT (Q5I7L5) Ribosomal protein L36, 93%	-0.01	
2050	TC317715	Zea mays clone cho1c.pk003.i13, mRNA sequence		-0.01
2070	TC323112	UP|RL7A_ORYSA (P35685) 60S ribosomal protein L7a		-0.01
2098	TC328924	UP|O82579_MAIZE (O82579) Ribosomal protein L26, 98%	-0.01	
2099	TC335017	UP|Q9AV87_ORYSA (Q9AV87) 60S ribosomal protein L21	-0.01	
2108	TC321719	Zea mays clone EL01N0308D08.c mRNA sequence	-0.01	

2112	TC357012	UP|P93626_MAIZE (P93626) 40S ribosomal subunit protein S21	-0.01	
2172	TC335017	UP|Q9AV87_ORYSA (Q9AV87) 60S ribosomal protein L21		-0.01
2304	TC342218	RF|XP_506724.1|51963882|XM_506724 OJ9003_G05.34 gene product {Oryza sativa (japonica cultivar-group)}	0.02	
2412	TC362382	UP|RS19_ORYSA (P40978) 40S ribosomal protein S19, 98%		-0.01
2430	TC369722	UP|RL41_ARATH (P62120) 60S ribosomal protein L41		-0.01
2473	TC332529	RF|XP_507485.1|51979244|XM_507485 OJ1126_B06.24 gene product {Oryza sativa (japonica cultivar-group)}		-0.01
2485	TC324946	UP|RL7A_ORYSA (P35685) 60S ribosomal protein L7a	-0.01	
2489	TC347178	UP|Q762A6_ORYSA (Q762A6) BRI1-KD interacting protein 108, 89%		-0.01
2491	TC333590	UP|Q3MST7_ORYSA (Q3MST7) Ribosomal L32		-0.01
2512	TC324399	UP|Q9FJA6_ARATH (Q9FJA6) 40S ribosomal protein S3 (AT5g35530/MOK9_14), 88%		-0.01
2544	TC318701	GB|CAA42105.1|16073|AMZEIN zein protein {Acetabularia mediterranea}	-0.01	
2588	TC339383	UP|Q5I7K2_WHEAT (Q5I7K2) Ribosomal protein S7	-0.01	
2598	TC341092	UP|Q7XY20_WHEAT (Q7XY20) Ribosomal protein L19, 43%		0.01
2814	TC336190	UP|Q7XC31_ORYSA (Q7XC31) 60S ribosomal protein L27		-0.01
2826	TC318708	UP|Q8H8S1_ORYSA (Q8H8S1) Ribosomal protein L15		-0.01
2875	TC342323	UP|Q8L4F2_ORYSA (Q8L4F2) 40S ribosomal protein S23		0.01
2882	TC333590	UP|Q3MST7_ORYSA (Q3MST7) Ribosomal L32	-0.01	
2885	TC369791	UP|Q2QNF3_ORYSA (Q2QNF3) 60s ribosomal protein l2		-0.01
2897	TC369026	UP|RS8_MAIZE (Q08069) 40S ribosomal protein S8	-0.01	-0.01
2906	TC345548	UP|Q8W1C9_MAIZE (Q8W1C9) Ribosomal protein L35A	-0.01	-0.01

2951	TC341880	UP|Q2R1J8_ORYSA (Q2R1J8) Ribosomal protein S4, 98%		-0.01
2965	TC321360	UP|Q9AV87_ORYSA (Q9AV87) 60S ribosomal protein L21		0.01
2975	TC341226	Zea mays clone cho1c.pk003.k1, mRNA sequence	-0.01	
3018	TC366592	UP|RL18A_ORYSA (Q943F3) 60S ribosomal protein L18a	-0.01	
3029	TC365889	UP|Q5VNV9_ORYSA (Q5VNV9) 40S subunit ribosomal protein, 80%	-0.01	
3201	TC346815	GB|BAA02155.1|303853|RICRPL3A ribosomal protein L3 {Oryza sativa (japonica cultivar-group)}	0.01	-0.01
3241	TC325889			-0.01
3298	TC334274	UP|RS4_MAIZE (O22424) 40S ribosomal protein S4		-0.02
3299	TC339525	UP|Q9FUL7_MAIZE (Q9FUL7) 40S ribosomal protein S24, 80%		-0.01
3384	TC324038	UP|Q7XIX6_ORYSA (Q7XIX6) RNaseP-associated protein-like, 80%	0.01	
3395	TC370212	UP|Q8H8S1_ORYSA (Q8H8S1) Ribosomal protein L15		0.01
3521	TC369811	UP|Q7XBH6_ORYSA (Q7XBH6) Ribosomal L9-like protein		-0.01
3605	TC324117	UP|Q8RZ90_ORYSA (Q8RZ90) Ribosomal protein L18a-like, 91%	-0.01	
3610	TC344993	GB|AAP85547.1|32493112|AY323130 ribosomal protein large subunit 13 {Oryza sativa (japonica cultivar-group)}		-0.01
3617	TC341839	UP|Q5U7K1_9POAL (Q5U7K1) S27 ribosomal protein	-0.01	-0.01
3623	TC347411	UP|Q9MB32_ORYSA (Q9MB32) Heat shock protein 90, 19%		-0.01
3657	TC316159	RF|XP_507392.1|51979030|XM_507392 B1056G08.113 gene product {Oryza sativa (japonica cultivar-group)}, 72%	0.01	
3685	TC325889		-0.01	
3824	TC322963	GB|BAA02155.1|303853|RICRPL3A ribosomal protein L3 {Oryza sativa (japonica cultivar-group)}		0.01
4077	TC334532	UP|Q7XC31_ORYSA (Q7XC31) 60S ribosomal protein L27		0.01

4082	TC335104	UP|RL7A_ORYSA (P35685) 60S ribosomal protein L7a, 80%		0.01
4091	TC325572	UP|RS141_MAIZE (P19950) 40S ribosomal protein S14 (Clone MCH1)		0.01
4105	TC342802	GB|AAX96401.1|62734292|AC133931 Ribosomal L38e protein family {Oryza sativa (japonica cultivar-group)}		0.01
4108	TC363721	UP|Q7XR19_ORYSA (Q7XR19) 60S ribosomal protein L6	0.01	
4182	TC334274	UP|RS4_MAIZE (O22424) 40S ribosomal protein S4	0.01	
4188	TC347178	UP|Q762A6_ORYSA (Q762A6) BRI1-KD interacting protein 108, 89%		0.01
4411	TC347350	RF|XP_507392.1|51979030|XM_507392 B1056G08.113 gene product {Oryza sativa (japonica cultivar-group)}, 72%	-0.02	0.01
4458	TC316271	UP|O22453_MAIZE (O22453) Ribsomal protein S4		0.01
4476	TC362382	UP|RS19_ORYSA (P40978) 40S ribosomal protein S19, 98%	0.01	
4484	TC316836	UP|Q6RJY1_CAPAN (Q6RJY1) 60S ribosomal protein L12		-0.01
4513	TC339626	UP|Q7GD83_ARATH (Q7GD83) 40S ribosomal protein S15A		-0.01
4577	TC340118	UP|RLA1_MAIZE (P52855) 60S acidic ribosomal protein P1 (L12)		0.01
4578	TC364589	Zea mays clone Contig325 mRNA sequence		0.01
4663	TC328181	UP|Q84XZ0_9LAMI (Q84XZ0) 60S ribosomal protein L34, 65%	0.02	
4777	TC341740	UP|Q5I7K3_WHEAT (Q5I7K3) Ribosomal protein S29		0.01
4850	TC325255	UP|Q2R1J8_ORYSA (Q2R1J8) Ribosomal protein S4, 98%		-0.01
4856	TC348584	UP|Q6LCT7_MAIZE (Q6LCT7) Ubiquitin fusion protein	-0.01	
4929	TC318484	UP|Q5I7K3_WHEAT (Q5I7K3) Ribosomal protein S29, 96%	0.01	-0.01
4957	TC318175	UP|Q7XY20_WHEAT (Q7XY20) Ribosomal protein L19, 89%		0.01
4983	TC350465	UP|Q8GTE2_CICAR (Q8GTE2) Ribosomal protein RL5		0.01

4986	TC325889		-0.01	
5029	TC337294	UP|RL17_MAIZE (O48557) 60S ribosomal protein L17	0.01	
5203	TC349603	UP|Q5I7K3_WHEAT (Q5I7K3) Ribosomal protein S29		-0.01
5228	TC346815	GB|BAA02155.1|303853|RICRPL3A ribosomal protein L3 {Oryza sativa (japonica cultivar-group)}		-0.01
5260	TC338324	UP|Q2R4A1_ORYSA (Q2R4A1) Ribosomal protein S7	0.01	
5305	TC325889		0.02	
5325	TC358642	UP|Q5I7K8_WHEAT (Q5I7K8) Ribosomal protein l34, 97%		0.01
5332	-	-	0.01	
5375	TC330576	UP|Q5I7L3_WHEAT (Q5I7L3) Ribosomal protein L10A	0.01	
5541	TC345934	UP|RL111_ARATH (P42795) 60S ribosomal protein L11-1 (L16A), 98%	0.01	
5546	TC330862	RF|XP_507356.1|51978958|XM_507356 OJ1014_E09.28 gene product {Oryza sativa (japonica cultivar-group)}, 97%		0.01
5557	TC327833	UP|RS142_MAIZE (P19951) 40S ribosomal protein S14 (Clone MCH2)		0.01
5735	TC342104	UP|Q6K955_ORYSA (Q6K955) 60S ribosomal protein-like, 19%		0.01
5759	TC366670	UP|Q7XEQ3_ORYSA (Q7XEQ3) Ribosomal S17		0.01
5787	TC342981	UP|RS26_ORYSA (P49216) 40S ribosomal protein S26 (S31), 91%	-0.01	
6119	TC369629	UP|Q5I7L1_WHEAT (Q5I7L1) Ribosomal protein L13a		0.01
6126	TC329095	UP|ZEB2_MAIZE (P08031) Zein-beta precursor (16 kDa)		0.01
6132	TC318701	GB|CAA42105.1|16073|AMZEIN zein protein {Acetabularia mediterranea}	0.01	
6172	TC342981	UP|RS26_ORYSA (P49216) 40S ribosomal protein S26 (S31), 91%		0.01
6177	TC364357	Zea mays clone Contig30.F mRNA sequence		0.02
6357	TC331934	UP|Q3MST7_ORYSA (Q3MST7) Ribosomal L32		0.01

6402	TC364389	UP|RS15_ORYSA (P31674) 40S ribosomal protein S15, 89%	-0.01	
6447	TC326854	RF|XP_507312.1|51965056|XM_507312 OJ1211_G06.30 gene product {Oryza sativa (japonica cultivar-group)}, 97%	0.01	
6452	TC340118	UP|RLA1_MAIZE (P52855) 60S acidic ribosomal protein P1 (L12)		0.01
6484	TC368507	UP|Q6L5M1_BROIN (Q6L5M1) Glycoprotein, 97%	0.01	
6500	TC332529	RF|XP_507485.1|51979244|XM_507485 OJ1126_B06.24 gene product {Oryza sativa (japonica cultivar-group)}		-0.01
6501	TC345208	GB|AAP85547.1|32493112|AY323130 ribosomal protein large subunit 13 {Oryza sativa (japonica cultivar-group)}	0.01	
6514	TC325789	Zea mays clone cho1c.pk002.j11, mRNA sequence		-0.01
6608	TC323112	UP|RL7A_ORYSA (P35685) 60S ribosomal protein L7a	0.01	
6747	TC368426	UP|Q8W1C9_MAIZE (Q8W1C9) Ribosomal protein L35A	0.01	
6835	TC359939	UP|Q5I7K6_WHEAT (Q5I7K6) Ribosomal protein L7, 79%	-0.01	
6849	-	-	-0.01	
6877	TC334532	UP|Q7XC31_ORYSA (Q7XC31) 60S ribosomal protein L27		0.01
6880	TC330340	UP|RS13_MAIZE (Q05761) 40S ribosomal protein S13		0.01
6906	TC349495	GB|AAX96401.1|62734292|AC133931 Ribosomal L38e protein family {Oryza sativa (japonica cultivar-group)}		0.01
6926	TC323112	UP|RL7A_ORYSA (P35685) 60S ribosomal protein L7a	0.01	
6964	TC369726	RF|XP_507485.1|51979244|XM_507485 OJ1126_B06.24 gene product {Oryza sativa (japonica cultivar-group)}		0.01
6973	TC349603	UP|Q5I7K3_WHEAT (Q5I7K3) Ribosomal protein S29		-0.01
6978	TC342119	UP|Q8GTE2_CICAR (Q8GTE2) Ribosomal protein RL5		0.01
6983	TC325255	UP|Q2R1J8_ORYSA (Q2R1J8) Ribosomal protein S4, 98%		-0.01

7058	TC369026	UP|RS8_MAIZE (Q08069) 40S ribosomal protein S8		-0.01
7146	TC338457	UP|Q9FUL7_MAIZE (Q9FUL7) 40S ribosomal protein S24, 94%	0.01	
7154	TC325255	UP|Q2R1J8_ORYSA (Q2R1J8) Ribosomal protein S4, 98%		-0.01
7210	TC341880	UP|Q2R1J8_ORYSA (Q2R1J8) Ribosomal protein S4, 98%		0.01
7257	TC318175	UP|Q7XY20_WHEAT (Q7XY20) Ribosomal protein L19, 89%		0.01
7260	TC328924	UP|O82579_MAIZE (O82579) Ribosomal protein L26, 98%		0.01
7273	TC338324	UP|Q2R4A1_ORYSA (Q2R4A1) Ribosomal protein S7	0.01	
7274	TC334532	UP|Q7XC31_ORYSA (Q7XC31) 60S ribosomal protein L27		0.01
7279	TC328966	RF|XP_506724.1|51963882|XM_506724 OJ9003_G05.34 gene product {Oryza sativa (japonica cultivar-group)}		0.01
7336	TC350051	RF|XP_506775.1|51963984|XM_506775 OJ1115_D03.49 gene product {Oryza sativa (japonica cultivar-group)}, 97%		0.01
7351	TC324946	UP|RL7A_ORYSA (P35685) 60S ribosomal protein L7a		0.01
7420	TC332529	RF|XP_507485.1|51979244|XM_507485 OJ1126_B06.24 gene product {Oryza sativa (japonica cultivar-group)}		0.01
7549	TC330438	UP|RS12_HORVU (Q9XHS0) 40S ribosomal protein S12, 93%	0.01	
7615	TC344993	GB|AAP85547.1|32493112|AY323130 ribosomal protein large subunit 13 {Oryza sativa (japonica cultivar-group)}		-0.01
7653	TC356842	UP|Q6XC06_MAIZE (Q6XC06) Glyoxalase I, 22%		-0.01
7663	TC366533	RF|XP_506775.1|51963984|XM_506775 OJ1115_D03.49 gene product {Oryza sativa (japonica cultivar-group)}, 85%	0.01	
7672	TC368426	UP|Q8W1C9_MAIZE (Q8W1C9) Ribosomal protein L35A		0.01
7688	TC336929	UP|Q6RI22_9ASPA (Q6RI22) 60S ribosomal protein L44	0.02	
7727	TC342775	UP|RL17_MAIZE (O48557) 60S ribosomal protein L17	0.01	

7733	-	-		0.01
7780	TC336499	UP|Q5I7K8_WHEAT (Q5I7K8) Ribosomal protein l34		-0.01
7879	TC325882	UP|RS15_ORYSA (P31674) 40S ribosomal protein S15, 98%		0.01
7956	TC351103	UP|Q7X9K4_WHEAT (Q7X9K4) S28 ribosomal protein, 78%	0.01	
8021	TC352496	Zea mays clone Contig349 mRNA sequence	0.01	
8106	TC360851	RF|XP_506775.1|51963984|XM_506775 OJ1115_D03.49 gene product {Oryza sativa (japonica cultivar-group)}	0.01	
8163	TC316822	UP|Q9FJA6_ARATH (Q9FJA6) 40S ribosomal protein S3 (AT5g35530/MOK9_14), 86%		-0.01
8172	TC350175	UP|Q20BM6_PANGI (Q20BM6) Ribosomal protein L31, 90%	0.01	
8310	TC331460	UP|Q6V959_WHEAT (Q6V959) Ribosomal protein L3, 56%	0.01	
8404	TC316271	UP|O22453_MAIZE (O22453) Ribsomal protein S4		-0.01
8409	TC323771	UP|RS10_ORYSA (Q9AYP4) 40S ribosomal protein S10, 95%		-0.01
8428	TC345208	GB|AAP85547.1|32493112|AY323130 ribosomal protein large subunit 13 {Oryza sativa (japonica cultivar-group)}	0.01	
8449	TC330576	UP|Q5I7L3_WHEAT (Q5I7L3) Ribosomal protein L10A	0.01	
8484	TC371087	UP|Q5GMM4_CAPCH (Q5GMM4) 60S ribosomal protein L37a		-0.01
8508	TC335247	UP|RL24_HORVU (P50888) 60S ribosomal protein L24	0.01	
8512	TC350465	UP|Q8GTE2_CICAR (Q8GTE2) Ribosomal protein RL5	-0.01	
8514	TC345548	UP|Q8W1C9_MAIZE (Q8W1C9) Ribosomal protein L35A	0.01	
8564	TC363721	UP|Q7XR19_ORYSA (Q7XR19) 60S ribosomal protein L6		-0.01
8724	TC346005	UP|Q9FUL7_MAIZE (Q9FUL7) 40S ribosomal protein S24	0.01	
8759	TC322963	GB|BAA02155.1|303853|RICRPL3A ribosomal protein L3 {Oryza sativa (japonica cultivar-group)}		0.01

8801	TC322766	UP|Q5WMY3_ORYSA (Q5WMY3) Cytoplasmic ribosomal protein L18		0.01
8830	TC324494	RF|XP_473060.1|50926169|XM_473060 {Oryza sativa (japonica cultivar-group)}		0.01
8876	TC339071	RF|XP_507356.1|51978958|XM_507356 OJ1014_E09.28 gene product {Oryza sativa (japonica cultivar-group)}		0.01
8877	TC369026	UP|RS8_MAIZE (Q08069) 40S ribosomal protein S8		-0.01
8897	TC350051	RF|XP_506775.1|51963984|XM_506775 OJ1115_D03.49 gene product {Oryza sativa (japonica cultivar-group)}, 97%		0.01
8963	TC361544	UP|Q5I7L5_WHEAT (Q5I7L5) Ribosomal protein L36, 93%	-0.01	
9215	TC329395	GB|AAP85547.1|32493112|AY323130 ribosomal protein large subunit 13 {Oryza sativa (japonica cultivar-group)}		0.01
9217	TC361544	UP|Q5I7L5_WHEAT (Q5I7L5) Ribosomal protein L36, 93%	-0.01	
9222	TC342775	UP|RL17_MAIZE (O48557) 60S ribosomal protein L17	0.01	
9245	TC322766	UP|Q5WMY3_ORYSA (Q5WMY3) Cytoplasmic ribosomal protein L18		0.01
9267	TC334274	UP|RS4_MAIZE (O22424) 40S ribosomal protein S4		0.01
9289	-	-		0.01
9297	TC328116	UP|Q2QYC7_ORYSA (Q2QYC7) Ribosomal protein S9, 97%	0.01	
9325	TC359103	Zea mays clone Contig890.F mRNA sequence	0.01	
9341	TC323771	UP|RS10_ORYSA (Q9AYP4) 40S ribosomal protein S10, 95%	0.01	
9384	TC330576	UP|Q5I7L3_WHEAT (Q5I7L3) Ribosomal protein L10A	0.01	
9470	TC362492	UP|Q2QNF3_ORYSA (Q2QNF3) 60s ribosomal protein l2, 36%	0.01	
9569	TC354655	UP|Q7G9L3_ARATH (Q7G9L3) ADP-ribosylation factor 1	-0.01	
9642	TC323771	UP|RS10_ORYSA (Q9AYP4) 40S ribosomal protein S10, 95%	0.01	
9709	TC337820	Zea mays clone EL01N0425H11.c mRNA sequence		0.01

9710	TC323192	UP|RS10_ORYSA (Q9AYP4) 40S ribosomal protein S10, 98%	0.01	
9958	TC344938	UP|RS4_MAIZE (O22424) 40S ribosomal protein S4, 52%	0.01	
10076	TC346347	UP|NOLA2_ARATH (Q9LEY9) H/ACA ribonucleoprotein complex subunit 2-like protein (Nhp2-like protein), 77%		-0.01
10113	TC321360	UP|Q9AV87_ORYSA (Q9AV87) 60S ribosomal protein L21		-0.01
10123	TC320924	UP|RL7A_ORYSA (P35685) 60S ribosomal protein L7a	0.01	
10138	TC346170	Zea mays clone Contig334 mRNA sequence	0.01	
10210	TC324946	UP|RL7A_ORYSA (P35685) 60S ribosomal protein L7a	0.01	
10311	TC370380	UP|RLA0_MAIZE (O24573) 60S acidic ribosomal protein P0	0.01	
10338	TC328593	UP|RS10_ORYSA (Q9AYP4) 40S ribosomal protein S10, 98%	0.01	
10412	TC337175	RF|XP_473060.1|50926169|XM_473060 {Oryza sativa (japonica cultivar-group)}		-0.01
10421	TC347178	UP|Q762A6_ORYSA (Q762A6) BRI1-KD interacting protein 108, 89%		-0.01
10514	TC316836	UP|Q6RJY1_CAPAN (Q6RJY1) 60S ribosomal protein L12	-0.01	
10539	TC347888	RF|NP_916542.1|34910390|NM_191653 ribosomal protein L28-like {Oryza sativa (japonica cultivar-group)}, 95%		0.01
10545	TC334634	Zea mays clone Contig717.F mRNA sequence		-0.01
10581	TC369026	UP|RS8_MAIZE (Q08069) 40S ribosomal protein S8	0.01	
10806	TC322766	UP|Q5WMY3_ORYSA (Q5WMY3) Cytoplasmic ribosomal protein L18		0.01
10810	TC335247	UP|RL24_HORVU (P50888) 60S ribosomal protein L24		-0.01
10860	TC324946	UP|RL7A_ORYSA (P35685) 60S ribosomal protein L7a		0.01
10863	TC337840	UP|Q7XC31_ORYSA (Q7XC31) 60S ribosomal protein L27		-0.01
10878	TC368300	UP|H2B3_MAIZE (Q43261) Histone H2B.3		-0.01

10880	TC333637	UP|Q8W1C9_MAIZE (Q8W1C9) Ribosomal protein L35A		-0.01
10911	TC316159	RF|XP_507392.1|51979030|XM_507392 B1056G08.113 gene product {Oryza sativa (japonica cultivar-group)}, 72%	0.01	-0.01
10960	TC323112	UP|RL7A_ORYSA (P35685) 60S ribosomal protein L7a	0.01	
10971	TC324387	UP|Q7XC31_ORYSA (Q7XC31) 60S ribosomal protein L27		-0.01
11203	TC330576	UP|Q5I7L3_WHEAT (Q5I7L3) Ribosomal protein L10A		-0.01
11226	TC352741	RF|NP_196094.1|15238305|NM_120556 amino acid binding {Arabidopsis thaliana}, 48%	0.01	-0.01
11252	TC350051	RF|XP_506775.1|51963984|XM_506775 OJ1115_D03.49 gene product {Oryza sativa (japonica cultivar-group)}, 97%	-0.01	0.02
11277	TC323112	UP|RL7A_ORYSA (P35685) 60S ribosomal protein L7a		-0.01
11299	TC316427	UP|Q5GWV4_XANOR (Q5GWV4) 50S ribosomal protein L15, 49%		-0.02
11387	TC326208	UP|Q7XBH6_ORYSA (Q7XBH6) Ribosomal L9-like protein		-0.01
11423	TC342429	UP|RS19_ORYSA (P40978) 40S ribosomal protein S19, 98%	0.01	
11458	TC362382	UP|RS19_ORYSA (P40978) 40S ribosomal protein S19, 98%	0.01	
11684	TC366533	RF|XP_506775.1|51963984|XM_506775 OJ1115_D03.49 gene product {Oryza sativa (japonica cultivar-group)}, 85%		-0.01
11695	TC324494	RF|XP_473060.1|50926169|XM_473060 {Oryza sativa (japonica cultivar-group)}	0.01	
11706	TC325572	UP|RS141_MAIZE (P19950) 40S ribosomal protein S14 (Clone MCH1)	0.01	
11708	TC325255	UP|Q2R1J8_ORYSA (Q2R1J8) Ribosomal protein S4, 98%		-0.01
11732	-	-		0.01
11733	TC322963	GB|BAA02155.1|303853|RICRPL3A ribosomal protein L3 {Oryza sativa (japonica cultivar-group)}	0.01	
11763	TC359495	RF|XP_506775.1|51963984|XM_506775 OJ1115_D03.49 gene product {Oryza sativa (japonica cultivar-group)}	0.01	

11774	TC323766	UP|Q9FUL7_MAIZE (Q9FUL7) 40S ribosomal protein S24	0.01	
11790	TC346005	UP|Q9FUL7_MAIZE (Q9FUL7) 40S ribosomal protein S24	0.01	
11948	TC325093	UP|Q8VZZ8_MAIZE (Q8VZZ8) Ribosomal protein L2		-0.01
12009	TC333658	UP|RS12_HORVU (Q9XHS0) 40S ribosomal protein S12, 91%	0.01	
12015	TC325572	UP|RS141_MAIZE (P19950) 40S ribosomal protein S14 (Clone MCH1)	0.01	
12020	TC331279	RF|XP_506724.1|51963882|XM_506724 OJ9003_G05.34 gene product {Oryza sativa (japonica cultivar-group)}	0.01	
12028	TC366592	UP|RL18A_ORYSA (Q943F3) 60S ribosomal protein L18a		0.01
12051	TC343031	RF|XP_507607.1|51979721|XM_507607 P0562A06.14 gene product {Oryza sativa (japonica cultivar-group)}		0.01
12108	TC363531	UP|Q9AV77_ORYSA (Q9AV77) 60S ribosomal protein L17		-0.01
12136	TC318771	UP|Q8GTE2_CICAR (Q8GTE2) Ribosomal protein RL5		0.01
12146	TC316271	UP|O22453_MAIZE (O22453) Ribsomal protein S4	0.01	
12322	TC328593	UP|RS10_ORYSA (Q9AYP4) 40S ribosomal protein S10, 98%		0.01
12343	TC341542	UP|RS141_MAIZE (P19950) 40S ribosomal protein S14 (Clone MCH1), 95%	0.01	
12344	TC359196	UP|Q8RZ90_ORYSA (Q8RZ90) Ribosomal protein L18a-like, 91%	0.01	
12416	TC348875	UP|Q6K853_ORYSA (Q6K853) 40S ribosomal protein S30-like		-0.01
12420	TC363559	UP|RL41_ARATH (P62120) 60S ribosomal protein L41	-0.01	
12434	TC325255	UP|Q2R1J8_ORYSA (Q2R1J8) Ribosomal protein S4, 98%		-0.01
12717	TC330576	UP|Q5I7L3_WHEAT (Q5I7L3) Ribosomal protein L10A		0.01
12751	TC336123	UP|RLA2A_MAIZE (P46252) 60S acidic ribosomal protein P2A (P2)	0.02	
12863	TC335199	UP|RL71_ARATH (P60040) 60S ribosomal protein L7-1, 92%	0.01	-0.02

	13075	TC324315	UP|RS19_ORYSA (P40978) 40S ribosomal protein S19, 98%		0.01
	13655	TC337840	UP|Q7XC31_ORYSA (Q7XC31) 60S ribosomal protein L27		0.01
	13698	TC338957	UP|RS15_ORYSA (P31674) 40S ribosomal protein S15	0.01	
	13815	TC321360	UP|Q9AV87_ORYSA (Q9AV87) 60S ribosomal protein L21	0.02	
	14351	TC361827	UP|RL10_MAIZE (P45633) 60S ribosomal protein L10	0.01	
	14413	TC328116	UP|Q2QYC7_ORYSA (Q2QYC7) Ribosomal protein S9, 97%	0.02	
	14457	TC362486	UP|Q8RZ90_ORYSA (Q8RZ90) Ribosomal protein L18a-like, 30%		-0.02
	14481	TC324117	UP|Q8RZ90_ORYSA (Q8RZ90) Ribosomal protein L18a-like, 91%	0.02	
	14801	TC330576	UP|Q5I7L3_WHEAT (Q5I7L3) Ribosomal protein L10A	0.01	
	15401	TC332427	UP|RS26_ORYSA (P49216) 40S ribosomal protein S26 (S31), 94%		0.01
	201	TC323903	UP|O82589_ARATH (O82589) F11O4.6, 30%	-0.01	
	4518	TC354770	UP|BXDC1_ORYSA (Q9AWM9) Brix domain-containing protein 1 homolog, 97%	-0.01	
	10055	TC354770	UP|BXDC1_ORYSA (Q9AWM9) Brix domain-containing protein 1 homolog, 97%		-0.02
	10144	TC354770	UP|BXDC1_ORYSA (Q9AWM9) Brix domain-containing protein 1 homolog, 97%		-0.02
	11686	TC354770	UP|BXDC1_ORYSA (Q9AWM9) Brix domain-containing protein 1 homolog, 97%		-0.01
Synthesis.mito/plastid ribosomal protein	10872	TC343295	UP|RM21_ARATH (Q8L9A0) 50S ribosomal protein L21, mitochondrial precursor, 51%		0.01
	5697	TC353606	UP|Q6H730_ORYSA (Q6H730) Ribosomal protein L12-like protein, 96%		0.01
	10317	TC349666	UP|RK28_ARATH (O22795) 50S ribosomal protein L28, chloroplast precursor, 57%	-0.01	
	10912	TC317556	UP|Q6H730_ORYSA (Q6H730) Ribosomal protein L12-like protein, 96%		-0.02
	13879	TC337398	RF|XP_506887.1|51964206|XM_506887 P0491E01.20 gene product {Oryza sativa (japonica cultivar-group)}, 52%	0.01	0.01
	15425	TC316935	UP|RR1_SPIOL (P29344) 30S ribosomal protein S1, chloroplast precursor (CS1), 83%	0.01	0.01

	4645	TC332850	GB|AAP31927.1|30387523|BT006583 At3g49080 {Arabidopsis thaliana}, 47%		0.01
	6134	-	-	-0.01	
	7648	TC348550	UP|RK29_MAIZE (Q9SWI6) 50S ribosomal protein L29, chloroplast precursor		0.01
	9821	TC343296	UP|Q944L5_ARATH (Q944L5) At1g26740/T24P13_11, 43%	0.01	
Synthesis.release	4230	TC336309	RF|XP_469434.1|50918075|XM_469434 eukaryotic peptide chain release factor subunit 1-3 (eRF1-3) {Oryza sativa (japonica cultivar-group)}		0.01
Targeting.chloroplast	3916	TC351158	UP|Q67UZ3_ORYSA (Q67UZ3) Chloroplast thylakoidal processing peptidase-like protein, 75%		0.01
	9579	TC340828	UP|SECA_PEA (Q41062) Preprotein translocase secA subunit, chloroplast precursor, 6%		0.01
Targeting.mitochondria	1328	TC330375	UP|Q7XI32_ORYSA (Q7XI32) Small zinc finger-like protein, 80%		-0.01
	4462	TC338637	UP|Q9AXQ2_CUCME (Q9AXQ2) Mitochondrial processing peptidase beta subunit, 63%	-0.01	
	5921	TC338875	UP|TIM13_ORYSA (Q9XGY5) Mitochondrial import inner membrane translocase subunit Tim13, 94%		0.02
	5952	TC350060	UP|Q9XGY3_MALDO (Q9XGY3) Small zinc finger-like protein, 87%		0.02
Targeting.nucleus	354	TC332416	UP|IMA1B_ORYSA (Q9SLX0) Importin alpha-1b subunit, 31%	-0.02	-0.01
	474	TC330100	UP|NTF2_ORYSA (Q9XJ54) Nuclear transport factor 2		-0.01
	3174	TC329566	Zea mays clone Contig824.F mRNA sequence		0.01
	4498	TC364802	Zea mays clone Contig677 mRNA sequence	-0.01	-0.01
	4552	TC323138	Zea mays clone Contig541.F mRNA sequence		-0.01
	5264	TC326154	UP|Q2HVW5_MEDTR (Q2HVW5) Nucleoporin interacting component Protein prenyltransferase, 23%		0.01
	5738	TC368286	UP|IMA1B_ORYSA (Q9SLX0) Importin alpha-1b subunit, 34%		0.01
	15135	TC340766	Zea mays clone EL01N0424D02.d mRNA sequence	0.01	
Targeting.secretory	788	TC328393	UP|Q9MAY9_MAIZE (Q9MAY9) Nonclathrin coat protein zeta1-COP	-0.01	

	pathway	13101	TC328668	UP|SC24A_ARATH (Q9SFU0) Protein transport protein Sec24-like At3g07100, 12%	0.01	
		12599	TC316097	Zea mays clone Contig412 mRNA sequence		-0.01
		31	TC326586	UP|Q6UCJ1_CUCSA (Q6UCJ1) Signal recognition particle receptor protein, 66%		-0.02
		1301	TC336895	GB|BAC79360.1|32879776|AB114831 signal recognition particle 54kDa subunit {Oryza sativa (japonica cultivar-group)}, 28%		-0.01
		5047	TC344514	UP|SC61G_ORYSA (P38385) Protein transport protein SEC61 gamma subunit		0.01
		5060	TC316181	Zea mays clone EL01N0424F12.d mRNA sequence	0.01	
		6749	TC320100	UP|Q6YZD2_ORYSA (Q6YZD2) Coated vesicle membrane protein-like, 96%		-0.01
		10664	TC353156	UP|SC61G_ORYSA (P38385) Protein transport protein SEC61 gamma subunit		-0.01
		13002	TC318103	Zea mays clone Contig122 mRNA sequence		0.01
		14578	TC349011	UP|SC61G_ORYSA (P38385) Protein transport protein SEC61 gamma subunit, 96%	0.01	
		6380	TC321275	Zea mays clone EL01N0316C05.c mRNA sequence		0.01
		10252	TC360480	UP|Q5JJV1_ORYSA (Q5JJV1) Vacuolar protein sorting 55 family-like	0.01	
Photosynthesis	Calvin cycle	1770	TC369233	UP|TPIC_SECCE (P46225) Triosephosphate isomerase, chloroplast precursor, 88%	-0.01	
		5658	TC369233	UP|TPIC_SECCE (P46225) Triosephosphate isomerase, chloroplast precursor, 88%		0.01
		2590	TC318980	UP|ALF_MAIZE (P08440) Fructose-bisphosphate aldolase, cytoplasmic isozyme	-0.01	
		14881	TC370414	UP|ALFC_ORYSA (Q40677) Fructose-bisphosphate aldolase, chloroplast precursor		0.01
		10901	TC343644	UP|PGKH_WHEAT (P12782) Phosphoglycerate kinase, chloroplast precursor, 33%		0.01
		10315	TC319860	UP|KPPR_WHEAT (P26302) Phosphoribulokinase, chloroplast precursor, 97%		0.01
	Lightreaction	5935	TC349893	UP|Q84PA4_ORYSA (Q84PA4) H+-transporting ATP synthase chain 9-like protein, 47%		0.01
		5796	TC334243	RF|XP_507256.1|51964944|XM_507256 P0493A04.32 gene product {Oryza sativa (japonica cultivar-group)}, 77%		0.01

		12731	TC344931	UP|O80429_MAIZE (O80429) Ferredoxin	-0.01	
		13876	TC335286	UP|FER1_MAIZE (P27787) Ferredoxin-1, chloroplast precursor	0.01	0.01
		710	TC370521	UP|PSAD_HORVU (P36213) Photosystem I reaction center subunit II, chloroplast precursor, 83%	0.02	0.01
		14874	TC340435	UP|Q2L3E3_BRASY (Q2L3E3) Photosystem 1 subunit 5, 61%	0.01	
		12894	TC326665	UP|Q41747_MAIZE (Q41747) Chlorophyll a/b-binding apoprotein CP26 precursor		0.02
		15284	TC358479	UP|CB21_MAIZE (P12329) Chlorophyll a-b binding protein 1, chloroplast precursor	0.01	0.01
		13095	TC320982	UP|PSBQ1_MAIZE (Q41048) Oxygen-evolving enhancer protein 3-1, chloroplast precursor	0.01	0.01
		15455	TC324016	UP|PSBO_WHEAT (P27665) Oxygen-evolving enhancer protein 1, chloroplast precursor , 98%		0.01
		12714	TC325580	RF|NP_192172.1|15235432|NM_116497 ATP binding {Arabidopsis thaliana}, 32%	0.01	0.01
	Photorespiration	8136	-	-		0.02
Redox	Ascorbate and glutathione	5766	TC327849	UP|Q6K680_ORYSA (Q6K680) Cytochrome b5 domain-containing protein-like, 98%		0.01
		1052	TC317139	UP|Q65XA0_ORYSA (Q65XA0) Dehydroascorbate reductase	-0.01	
		3724	TC336263	GB|BAA08264.1|1321661|D45423 ascorbate peroxidase {Oryza sativa}		-0.01
		12160	TC325195	UP|Q65XA0_ORYSA (Q65XA0) Dehydroascorbate reductase		0.01
		14022	TC336263	GB|BAA08264.1|1321661|D45423 ascorbate peroxidase {Oryza sativa}		-0.01
		15100	TC325671	UP|GME2_ORYSA (Q2R1V8) GDP-mannose 3, 5-epimerase 2		-0.01
		1397	TC317394	UP|GSHRC_ORYSA (P48642) Glutathione reductase, cytosolic (GR) (GRase) , 97%		0.01
		5624	TC317394	UP|GSHRC_ORYSA (P48642) Glutathione reductase, cytosolic (GR) (GRase) , 97%	-0.01	
		12621	TC318636	UP|Q5G1T9_WHEAT (Q5G1T9) Gamma-glutamylcysteine synthetase, 36%		0.01
	Dismutases and catalases	416	TC325282	UP|Q94L33_ARATH (Q94L33) Ania-6a type cyclin, 15%		-0.01
		417	TC337070	UP|SODC5_MAIZE (P23346) Superoxide dismutase [Cu-Zn] 4AP		-0.01

	722	TC329768	GB|AAA33511.1|168622|MZESOD2A SOD2 protein {Zea mays}		-0.01
	1371	TC321188	UP|SODC4_MAIZE (P23345) Superoxide dismutase [Cu-Zn] 4A		-0.02
	1687	TC337070	UP|SODC5_MAIZE (P23346) Superoxide dismutase [Cu-Zn] 4AP		-0.01
	3284	TC329768	GB|AAA33511.1|168622|MZESOD2A SOD2 protein {Zea mays}		-0.02
	4753	TC327155	UP|SODC2_MESCR (O49044) Superoxide dismutase [Cu-Zn] 2, 84%		0.01
	5666	-	-		0.01
	6186	TC329768	GB|AAA33511.1|168622|MZESOD2A SOD2 protein {Zea mays}		0.01
	9722	TC329768	GB|AAA33511.1|168622|MZESOD2A SOD2 protein {Zea mays}	0.01	
Glutaredoxins	894	TC338490	UP|Q84Z96_ORYSA (Q84Z96) Glutaredoxin protein family-like, 80%		-0.01
	4228	TC330958	Zea mays clone EL01N0558G04.c mRNA sequence		0.02
	8600	TC338490	UP|Q84Z96_ORYSA (Q84Z96) Glutaredoxin protein family-like, 80%	-0.01	
	10428	TC326201	UP|Q84Z96_ORYSA (Q84Z96) Glutaredoxin protein family-like, 72%	-0.01	-0.01
Misc	9900	TC345232	UP|NCB5R_ARATH (P83291) NADH-cytochrome b5 reductase-like protein (B5R) , 78%		0.01
Peroxiredoxin	12563	TC329950	GB|CAJ01693.1|67904930|AM039889 2-Cys peroxiredoxin {Oryza sativa (japonica cultivar-group)}, 81%		-0.01
Thioredoxin	1775	TC349862	UP|Q5EUE1_MAIZE (Q5EUE1) Protein disulfide isomerase	-0.01	
	2402	TC335905	UP|Q4W1F7_MAIZE (Q4W1F7) Thioredoxin h1 protein		-0.01
	3592	TC350147	UP|Q5EUC2_MAIZE (Q5EUC2) Adenosine 5'-phosphosulfate reductase 8, 31%	-0.01	
	5001	TC323131	UP|Q5EUD1_MAIZE (Q5EUD1) Protein disulfide isomerase		0.01
	6014	TC335905	UP|Q4W1F7_MAIZE (Q4W1F7) Thioredoxin h1 protein		-0.01
	7022	TC325410	UP|Q4W1F6_MAIZE (Q4W1F6) Thioredoxin h2 protein		0.01
	8103	TC322717	GB|AAL25614.1|16648849|AY058202 AT4g04950/T1J1_6 {Arabidopsis thaliana}, 84%	0.01	

		9000	TC325410	UP|Q4W1F6_MAIZE (Q4W1F6) Thioredoxin h2 protein	0.01	0.01
		12053	TC349862	UP|Q5EUE1_MAIZE (Q5EUE1) Protein disulfide isomerase	-0.01	0.01
		12424	TC335905	UP|Q4W1F7_MAIZE (Q4W1F7) Thioredoxin h1 protein	-0.01	
		15224	TC335905	UP|Q4W1F7_MAIZE (Q4W1F7) Thioredoxin h1 protein	0.01	
		15589	TC340829	Zea mays clone Contig166 mRNA sequence		0.01
RNA	Processing	489	TC353016	Zea mays clone EL01N0365E12.c mRNA sequence		-0.02
		941	TC365708	UP|Q9M6E6_TOBAC (Q9M6E6) Poly(A)-binding protein, 84%		-0.01
		1454	TC328932	UP|Q9SHY4_ARATH (Q9SHY4) F1E22.8, 14%		-0.01
		1498	TC338000	UP|RUXF_ARATH (Q9SUM2) Probable small nuclear ribonucleoprotein F, 97%		-0.01
		1651	TC340113	UP|Q8LAK5_ARATH (Q8LAK5) Small nuclear ribonucleoprotein homolog (At4g30330)	-0.01	
		2035	TC350023	UP|Q9FJN9_ARATH (Q9FJN9) Poly(A)-binding protein II-like, 71%	-0.01	
		3255	TC353173	GB|AAM70547.1|21700847|AY124838 AT3g07590/MLP3_4 {Arabidopsis thaliana}		0.01
		4338	TC318864	UP|Q9FJN9_ARATH (Q9FJN9) Poly(A)-binding protein II-like, 81%	0.01	
		4946	TC365708	UP|Q9M6E6_TOBAC (Q9M6E6) Poly(A)-binding protein, 84%	0.01	
		5021	TC328932	UP|Q9SHY4_ARATH (Q9SHY4) F1E22.8, 14%		0.01
		5969	TC325402	UP|Q6NQH4_ARATH (Q6NQH4) At1g02680 (TAF13) (Transcription factor TFIID), 72%		0.01
		6048	TC316492	RF|NP_567250.1|18412147|NM_116546 nucleic acid binding {Arabidopsis thaliana}, 34%	-0.02	0.02
		6919	TC352750	GB|AAM70547.1|21700847|AY124838 AT3g07590/MLP3_4 {Arabidopsis thaliana}	-0.01	
		6951	TC316815	UP|Q9LEB4_NICPL (Q9LEB4) RNA Binding Protein 45, 76%	0.02	
		8422	TC334832	UP|Q9FKB0_ARATH (Q9FKB0) Sm-like protein, 95%		-0.01
		9721	TC353016	Zea mays clone EL01N0365E12.c mRNA sequence		0.01

9772	TC317148	UP|Q8L5U6_ARATH (Q8L5U6) SnRNP core Sm protein Sm-X5-like protein		-0.01
9924	TC318918	UP|LSM4_ORYSA (Q9LGE6) Probable U6 snRNA-associated Sm-like protein LSm4		-0.01
10040	TC340391	RF|XP_507429.1|51979110|XM_507429 B1370C05.32 gene product {Oryza sativa (japonica cultivar-group)}		0.01
10087	TC353016	Zea mays clone EL01N0365E12.c mRNA sequence		-0.02
10196	TC317148	UP|Q8L5U6_ARATH (Q8L5U6) SnRNP core Sm protein Sm-X5-like protein		-0.01
10390	TC343274	UP|RUXG_ARATH (O82221) Probable small nuclear ribonucleoprotein G, 95%	-0.01	
10873	TC321989	UP|Q8L3W7_ARATH (Q8L3W7) Small nuclear ribonucleoprotein-like protein, 93%		0.01
12579	TC353016	Zea mays clone EL01N0365E12.c mRNA sequence		-0.01
15081	TC348359	GB|AAO23652.1|27765062|BT003087 At2g03870 {Arabidopsis thaliana}, 94%		0.01
249	TC336601	UP|Q6H874_ORYSA (Q6H874) DEAD/DEAH box helicase-like, 51%		-0.01
1824	TC347557	UP|Q9LU46_ARATH (Q9LU46) DEAD-box protein abstrakt, 14%		-0.01
11548	TC370333	Zea mays clone Contig595 mRNA sequence		-0.01
801	TC337634	UP|Q75KQ4_ORYSA (Q75KQ4) Expressed protein, 93%		-0.01
2011	TC320560	UP|Q6IVD0_MAIZE (Q6IVD0) Arginine/serine-rich splicing factor 2, 63%	-0.01	
3329	TC327822	RF|NP_178124.1|15220049|NM_106656 SUS2 (ABNORMAL SUSPENSOR 2) {Arabidopsis thaliana}, 24%		-0.01
4496	TC316321	Zea mays clone Contig631.F mRNA sequence		-0.01
4982	TC318699	UP|Q9FR79_ORYSA (Q9FR79) Pre-mRNA splicing factor, 65%		0.01
6030	TC320560	UP|Q6IVD0_MAIZE (Q6IVD0) Arginine/serine-rich splicing factor 2, 63%	-0.01	
6073	TC366189	UP|Q64HC3_MAIZE (Q64HC3) ASF/SF2-like pre-mRNA splicing factor SRP32		0.01
8749	TC363162	UP|Q64HB7_MAIZE (Q64HB7) ASF/SF2-like pre-mRNA splicing factor SRP31, 68%	0.01	

	10431	TC354282	UP|Q2QKC2_WHEAT (Q2QKC2) Pre-mRNA processing factor, 96%		-0.01
Regulation of transcription	3026	TC331449	GB|AAR25638.1|38638688|BT011002 At1g55300 {Arabidopsis thaliana}, 65%		0.01
	9666	TC327557	Zea mays clone EL01N0450G11.d mRNA sequence	-0.01	
	599	TC319612	UP|O49216_ORYSA (O49216) Nucleic acid binding protein, 79%		-0.01
	2230	TC319612	UP|O49216_ORYSA (O49216) Nucleic acid binding protein, 79%	-0.01	
	8203	TC319612	UP|O49216_ORYSA (O49216) Nucleic acid binding protein, 79%		-0.01
	8943	TC330804	RF|XP_506831.1|51964096|XM_506831 OJ1712_E04.22 gene product {Oryza sativa (japonica cultivar-group)}, 75%		-0.01
	8867	TC361801	UP|Q8S980_ORYSA (Q8S980) Auxin response factor 6b, 48%	-0.01	
	10948	TC327734	UP|Q8GST0_ORYSA (Q8GST0) Auxin response factor 1, 62%		0.01
	199	TC345741	UP|Q6K972_ORYSA (Q6K972) AGO1 homologous protein, 28%		-0.01
	2836	TC341177	UP|Q2LFC4_NICBE (Q2LFC4) AGO1-1, 43%		-0.02
	3732	TC345741	UP|Q6K972_ORYSA (Q6K972) AGO1 homologous protein, 28%	0.01	
	5911	TC347801	UP|Q2LFC3_NICBE (Q2LFC3) AGO1-2, 6%		0.01
	8068	TC316091	Zea mays clone Contig411.F mRNA sequence		-0.01
	2828	TC328570	UP|Q9FQ93_TOBAC (Q9FQ93) Anther ethylene-upregulated protein ER1, 50%	-0.01	
	7638	TC336347	UP|Q2WEL1_ORYSA (Q2WEL1) CaM-binding transcription factor, 31%		0.01
	3651	TC371167	UP|IAA3_ORYSA (Q5NB25) Auxin-responsive protein IAA3, 89%		-0.01
	8878	TC371167	UP|IAA3_ORYSA (Q5NB25) Auxin-responsive protein IAA3, 89%		0.01
	13537	TC371167	UP|IAA3_ORYSA (Q5NB25) Auxin-responsive protein IAA3, 89%		0.01
	3289	TC360833	UP|Q5MP56_MAIZE (Q5MP56) Barren stalk1		-0.02
	9745	-	-		-0.01

422	TC316584	UP|OCS1_MAIZE (P24068) Ocs element-binding factor 1 (OCSBF-1)		-0.01
1261	TC316584	UP|OCS1_MAIZE (P24068) Ocs element-binding factor 1 (OCSBF-1)		-0.01
4947	TC358956	UP|TGA4_ARATH (Q39162) Transcription factor TGA4, 55%		0.01
6317	TC323185	UP|Q41786_MAIZE (Q41786) Opaque2 heterodimerizing protein 2	0.01	0.01
7054	TC335475	UP|Q9SM11_MAIZE (Q9SM11) Em binding protein-1a, 50%	0.01	
11282	TC316584	UP|OCS1_MAIZE (P24068) Ocs element-binding factor 1 (OCSBF-1)		-0.01
11796	TC340015	UP|O82118_ORYSA (O82118) Zinc finger protein, 44%		0.01
12489	TC339782	UP|Q5JNB8_ORYSA (Q5JNB8) Zinc finger protein-like, 56%		-0.01
3056	-	-		-0.01
507	TC327319	RF|NP_174697.1|15218606|NM_103160 nucleic acid binding {Arabidopsis thaliana}, 42%		-0.01
896	TC359677	UP|Q5Z9H7_ORYSA (Q5Z9H7) Zinc finger protein-like, 89%	-0.01	-0.01
5379	TC363634	UP|Q94FY4_MAIZE (Q94FY4) C2H2 zinc-finger protein		0.01
5652	TC359677	UP|Q5Z9H7_ORYSA (Q5Z9H7) Zinc finger protein-like, 89%		0.01
90	TC353420	UP|Q9FU27_ORYSA (Q9FU27) CCCH-type zinc finger protein-like, 36%		-0.01
1016	TC328863	UP|Q69XQ3_ORYSA (Q69XQ3) KH domain-containing protein / zinc finger protein-like, 30%		-0.01
2522	TC317396	Zea mays clone EL01N0438E07.d mRNA sequence	-0.01	
3266	TC328649	UP|NFYB_MAIZE (P25209) Nuclear transcription factor Y subunit B(CAAT-box DNA-binding protein subunit B)	-0.02	
1829	TC341019	UP|Q7EYS4_ORYSA (Q7EYS4) Receptor like protein, 61%		-0.01
13372	TC346147	Zea mays clone EL01N0314H06.c mRNA sequence		0.01
1677	TC334623	UP|Q9AXT8_MAIZE (Q9AXT8) DNA cytosine methyltransferase MET2a	-0.01	
4649	TC315848	UP|Q8LPU6_MAIZE (Q8LPU6) DNA methyltransferase 101	-0.01	

5657	TC334623	UP|Q9AXT8_MAIZE (Q9AXT8) DNA cytosine methyltransferase MET2a		0.01
7180	TC356171	UP|Q84NK2_ORYSA (Q84NK2) Myb family transcription factor-like, 19%		0.01
11854	TC337991	UP|Q84NK2_ORYSA (Q84NK2) Myb family transcription factor-like, 21%		-0.01
11856	TC337991	UP|Q84NK2_ORYSA (Q84NK2) Myb family transcription factor-like, 21%		-0.01
812	TC322289	UP|Q94JE5_ORYSA (Q94JE5) Transcriptional coactivator-like, 84%		-0.01
12336	TC319449	GB|AAL11553.1|15983370|AF424559 At1g07470/F22G5_13 {Arabidopsis thaliana}, 43%		0.01
14286	TC364531	UP|Q69SU7_ORYSA (Q69SU7) Transcriptional coactivator p15 (PC4) family protein-like	0.01	
1694	TC324420	UP|Q6S3E0_9ROSI (Q6S3E0) Homeodomain protein HB2, 53%	-0.01	
3052	-	-	-0.02	
5460	-	-		0.01
6424	TC341077	UP|Q6RF30_MAIZE (Q6RF30) Rolled leaf1		0.01
8097	TC316855	UP|RSH1_MAIZE (Q41853) Homeobox protein rough sheath 1		-0.01
9367	TC331527	UP|Q7Y0W1_ORYSA (Q7Y0W1) GL2-type homeodomain protein, 15%		0.01
2413	TC326127	UP|Q94F81_MAIZE (Q94F81) HD2 type histone deacetylase HDA106	-0.01	
10650	TC316171	UP|HD2B_MAIZE (Q9M4U5) Histone deacetylase 2b	0.01	
14279	TC326127	UP|Q94F81_MAIZE (Q94F81) HD2 type histone deacetylase HDA106	0.01	
11338	TC354464	UP|Q5QLQ9_ORYSA (Q5QLQ9) HMG protein-like, 65%	0.01	
11647	TC327759	UP|Q84V72_MAIZE (Q84V72) M31 protein	0.01	
12174	TC331433	UP|MAD20_ORYSA (Q2QQA3) MADS-box trancription factor 20 (OsMADS20), 60%		-0.01
1282	TC358883	UP|Q8LK06_MAIZE (Q8LK06) Methyl binding domain protein MBD109, 83%		-0.01
4417	TC349735	GB|AAP21260.1|30102684|BT006452 At3g12560 {Arabidopsis thaliana}, 17%	-0.02	

12704	TC346568	UP|Q9AVV5_HORVU (Q9AVV5) MCB2 protein, 33%		0.01
81	TC364640	UP|MNB1B_MAIZE (P27347) DNA-binding protein MNB1B		-0.01
102	TC364640	UP|MNB1B_MAIZE (P27347) DNA-binding protein MNB1B		-0.01
220	TC364640	UP|MNB1B_MAIZE (P27347) DNA-binding protein MNB1B	-0.01	
483	TC364640	UP|MNB1B_MAIZE (P27347) DNA-binding protein MNB1B		-0.01
946	TC364640	UP|MNB1B_MAIZE (P27347) DNA-binding protein MNB1B		-0.01
988	TC364640	UP|MNB1B_MAIZE (P27347) DNA-binding protein MNB1B		-0.01
1251	TC364640	UP|MNB1B_MAIZE (P27347) DNA-binding protein MNB1B	-0.01	
2114	TC364640	UP|MNB1B_MAIZE (P27347) DNA-binding protein MNB1B	-0.01	
2618	TC364640	UP|MNB1B_MAIZE (P27347) DNA-binding protein MNB1B	-0.01	
2810	TC364640	UP|MNB1B_MAIZE (P27347) DNA-binding protein MNB1B		-0.02
3253	TC364640	UP|MNB1B_MAIZE (P27347) DNA-binding protein MNB1B		0.01
3281	TC364640	UP|MNB1B_MAIZE (P27347) DNA-binding protein MNB1B		-0.01
3333	TC364640	UP|MNB1B_MAIZE (P27347) DNA-binding protein MNB1B	0.01	
3376	TC364640	UP|MNB1B_MAIZE (P27347) DNA-binding protein MNB1B	0.01	
4111	TC364640	UP|MNB1B_MAIZE (P27347) DNA-binding protein MNB1B		0.01
4482	TC364640	UP|MNB1B_MAIZE (P27347) DNA-binding protein MNB1B		-0.01
5277	TC364640	UP|MNB1B_MAIZE (P27347) DNA-binding protein MNB1B		0.02
6157	TC364640	UP|MNB1B_MAIZE (P27347) DNA-binding protein MNB1B		-0.01
6173	TC364640	UP|MNB1B_MAIZE (P27347) DNA-binding protein MNB1B		0.01
6188	TC364640	UP|MNB1B_MAIZE (P27347) DNA-binding protein MNB1B		0.01

6522	TC364640	UP|MNB1B_MAIZE (P27347) DNA-binding protein MNB1B	0.01	
9338	TC364640	UP|MNB1B_MAIZE (P27347) DNA-binding protein MNB1B	0.01	
9344	TC364640	UP|MNB1B_MAIZE (P27347) DNA-binding protein MNB1B		-0.01
9352	TC364640	UP|MNB1B_MAIZE (P27347) DNA-binding protein MNB1B		0.01
9746	TC364640	UP|MNB1B_MAIZE (P27347) DNA-binding protein MNB1B	0.01	
9770	TC364640	UP|MNB1B_MAIZE (P27347) DNA-binding protein MNB1B	-0.01	
9783	TC364640	UP|MNB1B_MAIZE (P27347) DNA-binding protein MNB1B	0.01	
9808	TC364640	UP|MNB1B_MAIZE (P27347) DNA-binding protein MNB1B	0.01	
10121	TC364640	UP|MNB1B_MAIZE (P27347) DNA-binding protein MNB1B	0.01	
10458	TC364640	UP|MNB1B_MAIZE (P27347) DNA-binding protein MNB1B		0.01
10637	TC325826	UP|Q9LKY2_MAIZE (Q9LKY2) WD-repeat protein RBAP1, 88%		-0.01
10908	TC364640	UP|MNB1B_MAIZE (P27347) DNA-binding protein MNB1B	0.01	
11380	TC364640	UP|MNB1B_MAIZE (P27347) DNA-binding protein MNB1B	0.01	
12194	TC364640	UP|MNB1B_MAIZE (P27347) DNA-binding protein MNB1B	0.01	
12485	TC325479	UP|O80383_DAUCA (O80383) 98b, 56%	-0.02	
12551	TC364640	UP|MNB1B_MAIZE (P27347) DNA-binding protein MNB1B		0.01
895	TC317727	UP|Q8VZJ4_ARATH (Q8VZJ4) AT4g25730/F14M19_10, 30%		-0.01
2012	TC338706	UP|Q6DQ93_MUSAC (Q6DQ93) BTF3b-like transcription factor, 98%	-0.02	
2157	TC323456	GB|AAG40838.1|11878189|AF302492 NOP56-like protein {Arabidopsis thaliana}, 47%	-0.01	
2603	TC348770	RF|NP_181390.1|15224901|NM_129413 DNA binding {Arabidopsis thaliana}, 57%		0.01
2956	TC328113		-0.01	

3646	TC320314	UP|O22812_ARATH (O22812) AT-hook DNA-binding protein, 45%	0.02	
4011	TC325028	UP|PCNA_MAIZE (Q43266) Proliferating cell nuclear antigen (PCNA)	-0.01	
4658	TC324123	GB|AAR28023.1|39545920|AY463621 TAF6 {Arabidopsis thaliana}, 51%	-0.01	
4665	TC325507	UP|Q7XII4_ORYSA (Q7XII4) Remorin-like protein, 40%	-0.01	
6806	TC358642	UP|Q5I7K8_WHEAT (Q5I7K8) Ribosomal protein l34, 97%		0.01
6941	TC324617	UP|Q9LTV0_ARATH (Q9LTV0) Nucleolar protein, 86%		-0.01
7403	TC354762	UP|Q9M7F3_MAIZE (Q9M7F3) LIM transcription factor homolog, 52%		-0.02
7630	TC357075	UP|Q9M7F3_MAIZE (Q9M7F3) LIM transcription factor homolog		0.01
8996	TC357075	UP|Q9M7F3_MAIZE (Q9M7F3) LIM transcription factor homolog		0.01
9270	-	-		0.01
10025	TC324617	UP|Q9LTV0_ARATH (Q9LTV0) Nucleolar protein, 86%		-0.02
10976	TC323456	GB|AAG40838.1|11878189|AF302492 NOP56-like protein {Arabidopsis thaliana}, 47%	-0.01	-0.01
11767	TC331531	UP|Q8VYJ2_ARATH (Q8VYJ2) AT4g12080/F16J13_150, 29%	0.01	
12109	TC343260	UP|Q79FW8_MYCTU (Q79FW8) PE-PGRS FAMILY PROTEIN, 5%	-0.02	-0.01
12413	TC317249	Zea mays clone EL01N0327A01.c mRNA sequence	-0.01	
12472	TC341077	UP|Q6RF30_MAIZE (Q6RF30) Rolled leaf1		0.01
13779	TC317912	UP|Q9LQZ9_ARATH (Q9LQZ9) F10A5.22, 55%		0.01
14546	TC317249	Zea mays clone EL01N0327A01.c mRNA sequence	0.02	
5964	TC332381	UP|Q56YT3_ARATH (Q56YT3) Squamosa promoter binding protein-like 1, 25%		0.01
773	TC329103	UP|Q8L459_ORYSA (Q8L459) SET-domain transcriptional regulator family-like protein, 96%	-0.01	
3252	TC319117	UP|ATXR2_ARATH (Q5PP37) Histone-lysine N-methyltransferase ATXR2 , 29%		0.01

4399	TC337839	UP|Q8L821_MAIZE (Q8L821) SET domain-containing protein SET118, 17%		0.01
11304	TC319117	UP|ATXR2_ARATH (Q5PP37) Histone-lysine N-methyltransferase ATXR2 , 29%	0.01	
1909	TC341742	UP|Q94F75_MAIZE (Q94F75) Silencing group B protein	0.01	
380	TC334418	UP|PFD5_ARATH (P57742) Probable prefoldin subunit 5, 83%		0.01
454	TC331528	UP|Q6Z6E6_ORYSA (Q6Z6E6) Zinc finger transcription factor ZFP30, 92%		-0.02
583	TC326167	UP|Q3BCU2_MAIZE (Q3BCU2) Zinc finger protein		-0.01
2423	TC328589	UP|Q2IMJ3_ANADE (Q2IMJ3) LigA, 5%		0.01
2764	TC324694	Zea mays clone E04912704G01.c mRNA sequence	-0.01	
2795	TC331891	UP|Q69NK8_ORYSA (Q69NK8) CwfJ / zinc finger(CCCH-type)-like protein, 33%		0.01
2994	-	-		0.02
3002	TC322324	Zea mays clone EL01T0208A09.c mRNA sequence		-0.01
3370	TC343233	RF|XP_506746.1|51963926|XM_506746 OJ1225_F07.15 gene product {Oryza sativa (japonica cultivar-group)}		-0.01
4010	TC326167	UP|Q3BCU2_MAIZE (Q3BCU2) Zinc finger protein	-0.01	
4018	TC326167	UP|Q3BCU2_MAIZE (Q3BCU2) Zinc finger protein	-0.01	
4336	TC344147	UP|Q9SMT2_ARATH (Q9SMT2) Remorin-like protein, 39%		0.01
4467	TC331528	UP|Q6Z6E6_ORYSA (Q6Z6E6) Zinc finger transcription factor ZFP30, 92%	-0.01	
5739	-	-	-0.02	
5931	TC349234	RF|NP_196819.1|15240035|NM_121318 protein binding {Arabidopsis thaliana}, 21%		0.01
6162	TC337475	RF|NP_177101.2|42563074|NM_105609 metal ion binding {Arabidopsis thaliana}, 29%		0.01
6727	TC326167	UP|Q3BCU2_MAIZE (Q3BCU2) Zinc finger protein	0.02	
8330	-	-	0.02	

	8583	TC326167	UP|Q3BCU2_MAIZE (Q3BCU2) Zinc finger protein	0.01	
	9672	TC324152	UP|Q5NAR8_ORYSA (Q5NAR8) Purine rich element binding protein B-like, 75%		-0.01
	10511	TC318586	UP|Q9ZUM1_ARATH (Q9ZUM1) Expressed protein (At2g02170/F5O4.6), 23%		0.01
	10573	TC340108	UP|Q6Z6E6_ORYSA (Q6Z6E6) Zinc finger transcription factor ZFP30, 84%		0.01
	12039	TC320216	GB|AAP37853.1|30725662|BT008494 At1g11650 {Arabidopsis thaliana}, 45%	-0.01	
	12054	TC337761	UP|Q9ZUM1_ARATH (Q9ZUM1) Expressed protein (At2g02170/F5O4.6), 33%		0.01
	13438	TC326167	UP|Q3BCU2_MAIZE (Q3BCU2) Zinc finger protein	0.01	
	13609	TC354780	UP|Y1342_ARATH (Q9C810) PHD finger protein At1g33420, 13%	0.01	
	14306	TC331528	UP|Q6Z6E6_ORYSA (Q6Z6E6) Zinc finger transcription factor ZFP30, 92%	0.01	
	117	TC358046	UP|Q32SG4_MAIZE (Q32SG4) WRKY1		-0.02
	7269	TC358046	UP|Q32SG4_MAIZE (Q32SG4) WRKY1		-0.01
	8420	TC358046	UP|Q32SG4_MAIZE (Q32SG4) WRKY1	-0.01	-0.01
	9234	-	-		-0.02
	11037	TC360134	UP|Q949F7_ORYSA (Q949F7) WRKY-like DNA-binding protein, 44%		-0.01
	12486	TC358046	UP|Q32SG4_MAIZE (Q32SG4) WRKY1		-0.01
RNA binding	110	TC368465	Zea mays clone Contig980.F mRNA sequence		-0.02
	506	TC356293	UP|Q7XI13_ORYSA (Q7XI13) Glycine-rich RNA-binding protein-like, 47%		-0.02
	634	TC340045	UP|Q58T16_ARATH (Q58T16) FLK, 40%		-0.01
	947	TC318428	Zea mays clone EL01N0360D09.c mRNA sequence	-0.01	-0.01
	1693	TC316685	UP|Q68VB6_NICBE (Q68VB6) ALY protein, 44%	-0.01	
	2218	TC357630	UP|Q762A1_ORYSA (Q762A1) BRI1-KD interacting protein 113, 64%	-0.01	

2409	TC318428	Zea mays clone EL01N0360D09.c mRNA sequence	-0.01	
2457	TC320465	Zea mays clone EL01T0204C07.c mRNA sequence	-0.01	
2497	TC318428	Zea mays clone EL01N0360D09.c mRNA sequence	-0.01	-0.01
2502	TC343996	UP|Q651Z0_ORYSA (Q651Z0) RNA-binding protein-like, 42%	-0.02	
2515	TC368465	Zea mays clone Contig980.F mRNA sequence	-0.01	
2561	TC333114	Zea mays clone EL01N0526H03.c mRNA sequence	-0.01	
2826	TC316685	UP|Q68VB6_NICBE (Q68VB6) ALY protein, 44%		-0.01
2976	-	-	-0.02	
3242	TC340985	UP|Q42412_NICSY (Q42412) RNA-binding protein RZ-1, 69%		-0.01
3419	TC359617	GB|CAA80307.1|296548|MMFIBRLNA fibrillarin {Mus musculus}, 13%		0.01
4450	TC320562	Zea mays clone EL01N0424C12.d mRNA sequence	-0.01	
5019	TC359617	GB|CAA80307.1|296548|MMFIBRLNA fibrillarin {Mus musculus}, 13%		0.01
5711	TC368465	Zea mays clone Contig980.F mRNA sequence		0.01
6843	TC363572	UP|Q5ZDX8_ORYSA (Q5ZDX8) Heterogeneous nuclear ribonucleoprotein A2/B1-like, 83%		-0.01
7508	TC319296	Zea mays clone E04912708C10.c mRNA sequence		0.01
7764	TC368465	Zea mays clone Contig980.F mRNA sequence		-0.01
7850	TC330639	UP|Q9Z0H4_MOUSE (Q9Z0H4) Apoptosis-related RNA binding protein (Cugbp2 protein), 17%	0.01	
8498	TC318428	Zea mays clone EL01N0360D09.c mRNA sequence		-0.01
9236	TC368375	Zea mays clone EL01N0427G10.c mRNA sequence		0.01
9324	TC318428	Zea mays clone EL01N0360D09.c mRNA sequence	-0.01	
9964	TC341318	RF|NP_567249.1|18412143|NM_116545 RNA binding {Arabidopsis thaliana}, 22%		-0.02

		10120	TC368465	Zea mays clone Contig980.F mRNA sequence	-0.01	
		10125	TC342026	RF|NP_173208.1|15220810|NM_101627 RNA binding {Arabidopsis thaliana}, 52%		-0.01
		10771	TC320465	Zea mays clone EL01T0204C07.c mRNA sequence		-0.01
		10972	TC356293	UP|Q7XI13_ORYSA (Q7XI13) Glycine-rich RNA-binding protein-like, 47%		-0.01
		11036	TC368465	Zea mays clone Contig980.F mRNA sequence		-0.01
		11456	TC342544	UP|Q9VDI8_DROME (Q9VDI8) CG17838-PD, isoform D, 4%		-0.01
		12407	TC325449	UP|Q7XI13_ORYSA (Q7XI13) Glycine-rich RNA-binding protein-like, 68%	-0.01	-0.02
		12576	TC320465	Zea mays clone EL01T0204C07.c mRNA sequence		-0.01
		12579	TC353016	Zea mays clone EL01N0365E12.c mRNA sequence		-0.01
	Transcription	2470	TC321769	UP|Q9FJ98_ARATH (Q9FJ98) DNA-directed RNA polymerase II subunit-like protein, 74%	-0.01	
		4092	TC338505	UP|Q9ZTI2_MAIZE (Q9ZTI2) RNA polymerase sigma factor 2	-0.01	
		4902	TC337611	UP|Q6NLH0_ARATH (Q6NLH0) At3g16980, 39%		0.01
		7535	TC363009	GB|ABB47522.1|78708547|AE016959 expressed protein {Oryza sativa (japonica cultivar-group)}		0.01
		12510	TC339001	GB|AAR28030.1|39545934|AY463628 TAF10 {Arabidopsis thaliana}, 88%	-0.01	
		14778	TC319720	UP|Q4ABR3_BRARP (Q4ABR3) 80A08_4, 39%	0.01	
Secondary metabolism	Flavonoids	7472	-	-		0.02
	Isoprenoids	405	TC324140	UP|Q8S985_ORYSA (Q8S985) Arabidopsis ETTIN-like protein 1, 21%		-0.01
		12703	TC337919	UP|Q944F8_HEVBR (Q944F8) Hydroxymethylglutaryl coenzyme A synthase, 20%		0.02
		10043	TC361735	UP|Q944G1_HEVBR (Q944G1) Phosphomevalonate kinase, 16%	-0.01	
		259	TC341351	UP|Q6IWA6_9POAL (Q6IWA6) Cycloartenol synthase, 17%	-0.01	
	N misc	4652	TC322649	UP|Q8H5F0_ORYSA (Q8H5F0) Betaine aldehyde dehydrogenase-like, 96%	-0.01	

	Phenylpropanoids	5879	TC321542	UP|ZRP4_MAIZE (P47917) O-methyltransferase ZRP4, 69%		0.01
		10304	TC360718	UP|O49010_MAIZE (O49010) Herbicide safener binding protein		0.01
		10853	TC318677	RF|XP_507177.1|51964786|XM_507177 OSJNBb0070J06.25 gene product {Oryza sativa (japonica cultivar-group)}	-0.01	
		14037	TC317258	Zea mays clone EL01N0529H05.c mRNA sequence	0.02	
		7862	TC321460	UP|Q9M7S2_LOLPR (Q9M7S2) 4-coumarate--CoA ligase 4CL2, 61%		0.01
		11066	TC319703	UP|Q8S412_LOLPR (Q8S412) Cinnamyl alcohol dehydrogenase, 80%		0.01
		7089	TC328868	UP|Q7X5Y7_MAIZE (Q7X5Y7) Caffeoyl CoA 3-O-methyltransferase, 95%	-0.01	
		11151	TC327975	RF|XP_473058.1|50926145|XM_473058 {Oryza sativa (japonica cultivar-group)}, 31%		-0.02
		4315	TC315883	UP|Q8VXG7_MAIZE (Q8VXG7) Phenylalanine ammonia-lyase		0.01
	Simple phenols	10284	-	-	0.01	
	Wax	6104	TC328714	UP|Q2HW67_MEDTR (Q2HW67) Sterol desaturase, 54%		0.01
Signaling	14-3-3 proteins	2174	TC362113	UP|14331_MAIZE (P49106) 14-3-3-like protein GF14-6	-0.01	
		4888	TC322255	UP|Q6PLR9_MAIZE (Q6PLR9) 14-3-3-like protein, 89%	0.01	
		5698	TC318304	UP|O24223_ORYSA (O24223) GF14-d protein, 97%		0.01
		6379	TC316450	UP|14337_LYCES (P93212) 14-3-3 protein 7, 72%		0.01
	Calcium	13	TC324214	UP|Q7DLR7_MAIZE (Q7DLR7) Calmodulin		-0.01
		581	TC342822			-0.01
		880	TC364854	UP|Q43712_MAIZE (Q43712) Calcium-binding protein precursor (Calreticulin)	-0.01	-0.01
		1348	TC364854	UP|Q43712_MAIZE (Q43712) Calcium-binding protein precursor (Calreticulin)		-0.01
		2123	TC339753	UP|CALX_HELTU (Q39994) Calnexin homolog precursor, 39%		-0.01
		2166	TC324214	UP|Q7DLR7_MAIZE (Q7DLR7) Calmodulin	-0.01	

	4703	TC347162	UP|Q43699_MAIZE (Q43699) Calmodulin		0.01
	4968	TC364854	UP|Q43712_MAIZE (Q43712) Calcium-binding protein precursor (Calreticulin)	0.01	
	5175	TC325834	UP|Q42479_ARATH (Q42479) Calcium-dependent protein kinase, 42%		0.01
	6308	TC358185	UP|Q7XBA3_MAIZE (Q7XBA3) Group 3 pollen allergen		0.01
	8408	TC345850	UP|O49184_ORYSA (O49184) Calmodulin		-0.01
	9976	TC363006	UP|Q2V3M9_ARATH (Q2V3M9) Protein At3g59820, 61%		0.01
	10098	TC320057	Zea mays clone EK07D2312B08.c mRNA sequence	-0.01	
	10401	TC320057	Zea mays clone EK07D2312B08.c mRNA sequence	0.01	
	11459	TC336540	UP|Q41798_MAIZE (Q41798) Calnexin, 97%		-0.01
G-proteins	166	TC323818	UP|Q6AVS3_ORYSA (Q6AVS3) Expressed protein, 16%		-0.01
	169	TC332681	UP|RAB7_PENCL (Q40787) Ras-related protein Rab7		-0.01
	454	TC331528	UP|Q6Z6E6_ORYSA (Q6Z6E6) Zinc finger transcription factor ZFP30, 92%		-0.02
	778	TC333977	UP|Q41137_RICCO (Q41137) Eukaryotic release factor 3, 71%	-0.01	
	834	TC357523	UP|Q94K24_LYCES (Q94K24) Ran binding protein-1, 62%		-0.02
	953	TC334900	UP|O22470_ORYSA (O22470) GDP dissociation inhibitor protein OsGDI1		-0.02
	1292	TC334900	UP|O22470_ORYSA (O22470) GDP dissociation inhibitor protein OsGDI1		-0.02
	2636	TC365154	Zea mays clone EL01N0552F09.d mRNA sequence	-0.01	
	3295	TC357523	UP|Q94K24_LYCES (Q94K24) Ran binding protein-1, 62%	0.02	
	3625	TC332681	UP|RAB7_PENCL (Q40787) Ras-related protein Rab7	-0.01	
	3671	TC335389	UP|Q656P7_ORYSA (Q656P7) Root hair defective 3 GTP-binding protein-like, 40%		0.01
	4017	TC324616	UP|RB11D_TOBAC (Q40522) Ras-related protein Rab11D, 88%	-0.01	

4467	TC331528	UP|Q6Z6E6_ORYSA (Q6Z6E6) Zinc finger transcription factor ZFP30, 92%	-0.01	
4470	TC323232	Zea mays clone EL01N0314E02.c mRNA sequence	-0.01	
4506	TC357523	UP|Q94K24_LYCES (Q94K24) Ran binding protein-1, 62%	-0.01	
4961	TC319276	RF|NP_193883.2|30685465|NM_118272 nucleotide binding {Arabidopsis thaliana}, 66%		0.01
5723	TC318318	UP|Q9SVA6_ARATH (Q9SVA6) GTP-binding-like protein	0.01	0.01
6723	TC326054	UP|O81695_AVEFA (O81695) Ras-like small monomeric GTP-binding protein		-0.01
7094	TC336480	UP|Q5N703_ORYSA (Q5N703) RabGAP/TBC domain-containing protein-like, 47%	-0.01	
7803	TC316414	UP|O50019_MAIZE (O50019) Yptm3 protein		-0.01
8570	TC335465	GB|AAL16275.1|16226834|AF428345 AT3g54190/F24B22_150 {Arabidopsis thaliana}, 35%		0.01
9560	TC366622	GB|AAT28677.1|47499878|AY620417 GTP-binding protein {Oryza sativa (japonica cultivar-group)}	0.01	
9690	TC331147	UP|Q84ZE8_ORYSA (Q84ZE8) Auxin-regulated protein-like	0.01	
10212	TC323232	Zea mays clone EL01N0314E02.c mRNA sequence	-0.01	
10452	TC348968	UP|Q94K24_LYCES (Q94K24) Ran binding protein-1, 63%		0.01
10505	TC345387	UP|Q7GD79_ORYSA (Q7GD79) Small GTP-binding protein (Ran2)		0.01
10573	TC340108	UP|Q6Z6E6_ORYSA (Q6Z6E6) Zinc finger transcription factor ZFP30, 84%		0.01
11055	TC357523	UP|Q94K24_LYCES (Q94K24) Ran binding protein-1, 62%	-0.01	
11083	-	-		-0.01
11748	TC341678	UP|Q3S835_HORVU (Q3S835) RAB7, 26%		0.01
12368	TC319470	UP|Q9FPK1_ORYSA (Q9FPK1) Small GTP-binding protein RAB5B, 98%	0.01	
12553	TC332681	UP|RAB7_PENCL (Q40787) Ras-related protein Rab7		0.01
13289	TC347827	cytochrome P450 monooxygenase	0.02	

	14306	TC331528	UP|Q6Z6E6_ORYSA (Q6Z6E6) Zinc finger transcription factor ZFP30, 92%	0.01	
	14436	TC329697	RF|XP_507320.1|51965072|XM_507320 P0544G09.9 gene product {Oryza sativa (japonica cultivar-group)}, 83%	0.01	
	15000	TC357812	UP|RGP2_ORYSA (Q40723) Ras-related protein RGP2 (GTP-binding regulatory protein RGP2)		-0.02
Light	16	TC337635	GB|AAT85270.1|50881425|AC133335 expressed protein {Oryza sativa (japonica cultivar-group)}, 96%		-0.01
	3651	TC371167	UP|IAA3_ORYSA (Q5NB25) Auxin-responsive protein IAA3, 89%		-0.01
	4435	TC319190	GB|AAT85270.1|50881425|AC133335 expressed protein {Oryza sativa (japonica cultivar-group)}, 96%	-0.01	
	8878	TC371167	UP|IAA3_ORYSA (Q5NB25) Auxin-responsive protein IAA3, 89%		0.01
	14240	TC348377	UP|LIRP1_ORYSA (Q03200) Light-regulated protein precursor, 62%	-0.01	-0.01
	2118	TC361767	Zea mays clone EL01N0511G04.d mRNA sequence		-0.01
	9118	TC345577	UP|Q7XI09_ORYSA (Q7XI09) COP1-interacting protein 7 (CIP7)-like protein, 19%		0.01
	14781	TC328922	UP|CSN4_ARATH (Q8L5U0) COP9 signalosome complex subunit 4	0.01	
	15406	TC371218	UP|Q9M6R3_ORYSA (Q9M6R3) Constitutive photomorphogenic 11, 31%		0.01
MAP kinases	630	TC316755	UP|Q6Z437_ORYSA (Q6Z437) MAP kinase MAPK2		-0.01
	1579	TC342552	RF|NP_187455.1|15231910|NM_111677 MAPKKK6 ATP binding {Arabidopsis thaliana}, 4%	-0.01	
	4861	TC316110	UP|O49975_MAIZE (O49975) Protein kinase ZmMEK1		0.01
	6924	TC327899	RF|NP_191892.2|22331922|NM_116198 ATP binding {Arabidopsis thaliana}, 44%		0.01
	8842	TC316110	UP|O49975_MAIZE (O49975) Protein kinase ZmMEK1	-0.01	
Phosphinositides	4075	TC323772	RF|XP_506641.1|51963722|XM_506641 P0523B07.38-1 gene product {Oryza sativa (japonica cultivar-group)}		0.01
	12085	TC336141	UP|Q69SU0_ORYSA (Q69SU0) Inositol 5-phosphatase 3-like protein, 29%		-0.01

		656	TC366721	UP|Q6ZJN4_ORYSA (Q6ZJN4) Phosphatidylinositol 3, 5-kinase-like, 5%		-0.01
	Receptor kinases	12378	TC351791	UP|EXS_ARATH (Q9LYN8) Leucine-rich repeat receptor protein kinase EXS precursor, 5%		0.01
		9731	TC334736	UP|Q4ABW3_BRARP (Q4ABW3) 4D11_24, 38%	-0.01	
		8166	TC330342	UP|Q6Y2W9_MAIZE (Q6Y2W9) Atypical receptor-like kinase MARK		-0.01
		8534	TC324554	UP|Q67ZT9_ARATH (Q67ZT9) Receptor-kinase isolog, 37%		0.02
		3909	TC338207	UP|RIPK2_MOUSE (P58801) Receptor-interacting serine/threonine-protein kinase 2, 5%	-0.01	
		4	TC334747	UP|Q84XU5_ELAGV (Q84XU5) Receptor-like protein kinase, 47%	-0.01	
		2292	TC368374	GB|AAA18780.1|488466|MMU08339 GDF7 {Mus musculus}, 15%	-0.01	
		5130	TC371000	UP|Q5ZBB1_ORYSA (Q5ZBB1) Dual-specific kinase DSK1-like, 6%		0.01
		5438	TC335983	RF|NP_173940.2|79352581|NM_102380 ATP binding {Arabidopsis thaliana}, 42%		0.02
		15175	TC334394	UP|Q76BK8_TOBAC (Q76BK8) PERK1-like protein kinase, 45%	0.01	
	Sugar and nutrient physiology	769	TC336760	UP|Q7Y0S8_SOLTU (Q7Y0S8) Erg-1, 42%	-0.01	
		6907	TC343667	RF|XP_506983.1|51964396|XM_506983 P0627E03.37-2 gene product {Oryza sativa (japonica cultivar-group)}, 19%		-0.01
		9398	TC316748	UP|Q8LF96_ARATH (Q8LF96) PRL1 protein, 78%	0.01	
		8032	TC334570	UP|O82423_MAIZE (O82423) Pyruvate dehydrogenase kinase isoform 1		0.01
Stress	Abiotic	12064	TC318776	Zea mays clone EL01N0523E03.c mRNA sequence	0.01	
	Abiotic.cold	919	TC360345	Zea mays clone Contig55 mRNA sequence	-0.01	
		3422	TC347320			-0.01
	Abiotic.drought/salt	9509	TC358512	Zea mays clone EL01N0507F07.c mRNA sequence		0.01
		11641	TC332105	UP|Q5QMP3_ORYSA (Q5QMP3) Fiber protein Fb2-like, 81%	0.01	
	Abiotic.heat	96	TC334920	UP|Q9LHA8_ARATH (Q9LHA8) 70 kDa heat shock protein (AT3g12580/T2E22_110), 75%		-0.01

171	TC347913	RF|NP_680194.1|22326960|NM_147889 heat shock protein binding {Arabidopsis thaliana}, 47%		-0.01
863	TC347411	UP|Q9MB32_ORYSA (Q9MB32) Heat shock protein 90, 19%		-0.01
3361	TC316727	UP|Q53NM9_ORYSA (Q53NM9) DnaK-type molecular chaperone hsp70-rice	0.01	
3623	TC341609	UP|Q9MB32_ORYSA (Q9MB32) Heat shock protein 90, 19%		-0.01
4619	TC316727	UP|Q53NM9_ORYSA (Q53NM9) DnaK-type molecular chaperone hsp70-rice	-0.01	0.01
5378	TC320862	UP|HSP7M_PHAVU (Q01899) Heat shock 70 kDa protein, mitochondrial precursor, 93%		0.01
5642	TC349327	UP|HSP81_ORYSA (P33126) Heat shock protein 81-1		0.01
6043	TC321298	UP|HSP81_ORYSA (P33126) Heat shock protein 81-1, 64%		0.01
6943	TC316139	UP|O65160_MAIZE (O65160) DnaJ-related protein ZMDJ1		-0.01
7114	TC331556	RF|NP_849977.1|30680384|NM_179646 heat shock protein binding {Arabidopsis thaliana}, 21%		0.01
7376	TC331864	UP|Q43638_SECCE (Q43638) Heat-shock protein precursor, 75%		0.02
8858	TC349327	UP|HSP81_ORYSA (P33126) Heat shock protein 81-1		0.01
9842	TC321298	UP|HSP81_ORYSA (P33126) Heat shock protein 81-1, 64%	0.01	
10068	TC364947	UP|Q8LIK7_ORYSA (Q8LIK7) DnaJ protein family-like protein, 78%		-0.01
10537	TC340577	Zea mays clone Contig439 mRNA sequence		0.01
10568	TC340794	UP|Q40867_PENAM (Q40867) Heat shock protein 17.9	0.01	
10614	TC329368	RF|NP_680194.1|22326960|NM_147889 heat shock protein binding {Arabidopsis thaliana}, 97%		0.02
12916	TC337378	UP|Q5ZCV2_ORYSA (Q5ZCV2) DNAJ heat shock N-terminal domain-containing protein-like, 22%	0.01	0.01
13646	TC326224	UP|Q5VRZ9_ORYSA (Q5VRZ9) DnaJ protein-like, 18%	0.01	
13848	TC353080	Zea mays clone EK07D2312C08.c mRNA sequence	0.01	0.01
14006	TC340090	UP|Q8SB39_ORYSA (Q8SB39) Heat shock protein 90, 34%	0.01	

		14991	TC337494	UP|HSP81_ORYSA (P33126) Heat shock protein 81-1	0.01	
	Abiotic.touch/wounding	3514	TC331915	RF|XP_507201.1|51964834|XM_507201 P0426E02.15-2 gene product {Oryza sativa (japonica cultivar-group)}, 42%	-0.01	
	Abiotic.unspecified	6308	TC358185	UP|Q7XBA3_MAIZE (Q7XBA3) Group 3 pollen allergen		0.01
		6933	TC369189	GB|AAN31783.1|23396189|AC134516 Putataive pollen specific protein C13 precursor {Oryza sativa (japonica cultivar-group)}, 88%		0.01
		9355	TC332882	UP|Q9LWS6_ORYSA (Q9LWS6) Ozone-responsive stress-related protein-like	-0.01	
	Biotic	3886	TC342468	Zea mays clone Contig17 mRNA sequence		0.02
		10028	TC333594	UP|Q8L698_WHEAT (Q8L698) Defensin precursor, 82%	-0.01	
		14868	-	-		0.01
TCA / org	Transformation.other organic acid transformaitons	6199	TC338918	UP|MDHC_MAIZE (Q08062) Malate dehydrogenase, cytoplasmic	0.01	
		8857	TC338918	UP|MDHC_MAIZE (Q08062) Malate dehydrogenase, cytoplasmic	0.01	
		5803	TC320434			0.01
		1451	TC342810	Zea mays clone EL01N0311E09.c mRNA sequence		-0.02
		5478	TC346935	UP|Q9SIB9_ARATH (Q9SIB9) Cytoplasmic aconitate hydratase, 15%	-0.01	
		8919	TC341315	UP|ACOC_CUCMA (P49608) Aconitate hydratase, cytoplasmic, 65%		0.01
		10660	TC346935	UP|Q9SIB9_ARATH (Q9SIB9) Cytoplasmic aconitate hydratase, 15%		0.01
		13743	TC356756	UP|Q9SIB9_ARATH (Q9SIB9) Cytoplasmic aconitate hydratase, 25%	0.01	0.01
		10071	TC323156	UP|Q9XGU8_ORYSA (Q9XGU8) NADP-specific isocitrate dehydrogenase, 62%		-0.02
		4378	TC327187	UP|Q9SWR9_MAIZE (Q9SWR9) Dihydrolipoamide S-acetyltransferase		0.01
		15013	TC340359	RF|XP_507349.1|51978944|XM_507349 P0507H12.6 gene product {Oryza sativa (japonica cultivar-group)}, 20%		-0.02

		3134	TC325985	RF|XP_506871.1|51964174|XM_506871 OJ1234_B11.18 gene product {Oryza sativa (japonica cultivar-group)}		0.01
Tetrapyrrole synthesis	Chlorophyll b synthase	2896	TC325932	UP|Q9FYV0_ORYSA (Q9FYV0) LLS1 protein, 44%	-0.01	-0.01
	Ferrochelatase	5176	TC328133	GB|AAB71887.1|2460251|AF020791 ferrochelatase {Hordeum vulgare}, 34%		0.01
	GSA	10437	TC349802			-0.01
	Heme oxygenase	13935	TC344824	UP|Q94FW8_SORBI (Q94FW8) Heme oxygenase 2, 39%	0.01	0.01
	Magnesium chelatase	15651	TC360217	UP|Q94C01_HORVD (Q94C01) Mg-chelatase subunit XANTHA-F, 21%	0.01	
	Porphobilinogen deaminase	3118	TC316595	UP|Q8RYB1_WHEAT (Q8RYB1) Porphobilinogen deaminase	-0.01	
	Sirohydrochlorin ferrochelatase	7178	TC342730	UP|Q9XE75_SORBI (Q9XE75) Patatin-like protein, 22%		0.01
Transport	ABC transporters and multidrug resistance systems	932	TC365137	UP|Q8GU82_ORYSA (Q8GU82) PDR-like ABC transporter, 14%		-0.01
		14038	TC351515	UP|Q8GU82_ORYSA (Q8GU82) PDR-like ABC transporter, 17%	0.02	
	Amino acids	9441	TC358852	UP|Q6AST5_ORYSA (Q6AST5) Amino acid permease domain containing protein, 93%	0.01	
	Calcium	10204	TC317575	UP|CAX1A_ORYSA (Q769E5) Vacuolar cation/proton exchanger 1a (Ca(2+)/H(+) exchanger 1a) (OsCAX1a), 84%	-0.01	
	Major Intrinsic Proteins	6661	TC316215	UP|Q9ATM6_MAIZE (Q9ATM6) Plasma membrane integral protein ZmPIP2-4		0.02
		7301	TC340492	UP|O64964_MAIZE (O64964) Tonoplast intrinsic protein		0.01
		9688	TC340492	UP|O64964_MAIZE (O64964) Tonoplast intrinsic protein	0.01	
	Metabolite transporters at the envelope membrane	6896	TC317834	UP|ADT_ORYSA (P31691) ADP,ATP carrier protein, mitochondrial precursor, 82%		0.01
		9266	TC361986	UP|ADT1_MAIZE (P04709) ADP,ATP carrier protein 1, mitochondrial precursor	0.01	
		12066	TC361986	UP|ADT1_MAIZE (P04709) ADP,ATP carrier protein 1, mitochondrial precursor		-0.01

	12873	TC330885	UP|TPT_MAIZE (P49133) Triose phosphate/phosphate translocator, chloroplast precursor (cTPT), 33%	0.01	0.01
Metabolite transporters at the mitochondrial membrane	770	TC336665	UP|Q6YVE7_ORYSA (Q6YVE7) Mitochondrial aspartate-glutamate carrier protein-like, 62%	-0.01	
	4925	TC316120	Zea mays clone EL01N0561A03.c mRNA sequence	0.01	
	5127	TC325048	RF|NP_198104.1|15240964|NM_122634 binding {Arabidopsis thaliana}, 87%	0.01	
	6896	TC317834	UP|ADT_ORYSA (P31691) ADP,ATP carrier protein, mitochondrial precursor, 82%		0.01
	9266	TC361986	UP|ADT1_MAIZE (P04709) ADP,ATP carrier protein 1, mitochondrial precursor	0.01	
	11344	TC317834	UP|ADT_ORYSA (P31691) ADP,ATP carrier protein, mitochondrial precursor, 82%		-0.01
	12066	TC361986	UP|ADT1_MAIZE (P04709) ADP,ATP carrier protein 1, mitochondrial precursor		-0.01
	14310	TC318389	Zea mays clone EL01N0520C05.d mRNA sequence	0.01	
Metal	10204	TC317575	UP|CAX1A_ORYSA (Q769E5) Vacuolar cation/proton exchanger 1a (Ca(2+)/H(+) exchanger 1a) (OsCAX1a), 84%	-0.01	
	15428	TC358775	UP|Q257D6_HORVU (Q257D6) Zinc transporter protein ZIP7, 26%	0.02	
Misc	619	TC317022	UP|Q2TJ67_ORYSA (Q2TJ67) Plastid ATP/ADP transporter, 74%		-0.01
	2418	TC317406	UP|Q5N9C8_ORYSA (Q5N9C8) SPP30-like		0.01
	3152	TC317406	UP|Q5N9C8_ORYSA (Q5N9C8) SPP30-like		0.01
	4075	TC323772	RF|XP_506641.1|51963722|XM_506641 P0523B07.38-1 gene product {Oryza sativa (japonica cultivar-group)}		0.01
	4728	TC363898	UP|Q9LSH7_ARATH (Q9LSH7) Transporter-like protein, 25%		0.01
	5027	TC317406	UP|Q5N9C8_ORYSA (Q5N9C8) SPP30-like	0.01	
	5129	TC333034	UP|Q6ZCZ7_ORYSA (Q6ZCZ7) Phosphatidylinositol transfer-like, 57%		0.01
	5488	TC344207	UP|Q9VVI1_DROME (Q9VVI1) CG7564-PA (LD04387p), 5%		0.01
	7667	TC338556	RF|NP_177653.1|15222153|NM_106173 transporter {Arabidopsis thaliana}, 68%		-0.01

	9150	TC321414	RF|XP_506412.1|51963480|XM_506412 OJ1112_E08.118 gene product {Oryza sativa (japonica cultivar-group)}	0.01	
	10925	TC317022	UP|Q2TJ67_ORYSA (Q2TJ67) Plastid ATP/ADP transporter, 74%		-0.01
	11670	TC324295	UP|Q8GRK9_ORYSA (Q8GRK9) Transport protein particle component Bet3-like protein, 97%	0.01	
	14252	-	-		-0.02
P- and v-ATPases	15536	TC340232	UP|VATC_ARATH (Q9SDS7) Vacuolar ATP synthase subunit C, 47%		-0.02
	616	TC316468	UP|VATE_CITLI (Q9SWE7) Vacuolar ATP synthase subunit E		0.01
	1017	TC342725	UP|VATL_AVESA (P23957) Vacuolar ATP synthase 16 kDa proteolipid subunit, 55%		-0.02
	2701	TC329393	UP|VATF_ARATH (Q9ZQX4) Probable vacuolar ATP synthase subunit F , 98%		0.01
	6511	TC316468	UP|VATE_CITLI (Q9SWE7) Vacuolar ATP synthase subunit E		0.01
	8303	TC351589	UP|VATF_ARATH (Q9ZQX4) Probable vacuolar ATP synthase subunit F , 88%	0.01	
	9220	TC316383	UP|Q945E8_PENAM (Q945E8) Vacuolar H+-ATPase 16 kDa proteolipid subunit c	-0.01	
	12309	TC330115	UP|Q945E8_PENAM (Q945E8) Vacuolar H+-ATPase 16 kDa proteolipid subunit c		0.01
	14322	TC330827	UP|Q93W07_ORYSA (Q93W07) Vacuolar ATPase B subunit, 56%	0.01	-0.01
	15159	TC348484	UP|Q2L9B8_WHEAT (Q2L9B8) Vacuolar ATP synthase subunit E	0.01	
Phosphate	8849	TC336778	RF|NP_181341.2|42569720|NM_129362 carbohydrate transporter/ organic anion transporter/ sugar porter {Arabidopsis thaliana}, 35%		0.01
Porins	5838	TC321095	UP|Q7Y1C6_PENAM (Q7Y1C6) PgPOR29 (Voltage dependent anion channel protein)		0.01
Unspecified anions	13983	TC330189		0.01	
	14149	TC349106	UP|Q2L3B0_BRASY (Q2L3B0) Chloride channel-f protein, 19%		0.01
	2804	TC322146	RF|NP_849575.1|30678203|NM_179244 ATP binding {Arabidopsis thaliana}, 89%	-0.01	-0.01
	4201	TC327920	UP|Q2VT97_9POAL (Q2VT97) Na+/H+ antiporter		0.01

		6454	TC317834	UP|ADT_ORYSA (P31691) ADP,ATP carrier protein, mitochondrial precursor, 82%		0.01
		7457	TC349025	UP|Q67UQ7_ORYSA (Q67UQ7) Magnesium transporter CorA-like, 22%		0.01
		7903	TC343346	GB|AAR27948.1|39545690|AY463691 DUR3 {Oryza sativa (japonica cultivar-group)}, 14%		0.01
		11344	TC317834	UP|ADT_ORYSA (P31691) ADP,ATP carrier protein, mitochondrial precursor, 82%		-0.01
		12498	TC327266	GB|AAN73212.1|25360813|AY150287 MRS2-2 {Arabidopsis thaliana}, 80%	-0.01	-0.01
Transporter	Sugars	4734	TC324113	UP|Q9FRT7_ORYSA (Q9FRT7) Monosaccharide transporter 1, 67%		0.01
		7784	-	-		0.02
		10468	TC324798	UP|Q8GT52_HORVU (Q8GT52) Hexose transporter, 23%		0.01
		4494	TC348162	UP|Q6PST5_MAIZE (Q6PST5) Sucrose transporter 2, 43%	-0.01	
Not assigned	No ontology	190	TC320041	UP|Q5QMR4_ORYSA (Q5QMR4) Integral membrane protein-like, 97%		-0.01
		228	TC341710			-0.01
		438	TC316482			-0.02
		563	TC328713			-0.02
		609	TC316200	UP|Q9M582_MAIZE (Q9M582) Hypersensitive-induced response protein		-0.01
		632	TC336207	UP|Q84UC4_ARATH (Q84UC4) HASTY, 10%	-0.01	
		805	TC370071	UP|Q8H6A5_MAIZE (Q8H6A5) Translationally controlled tumor protein-like protein		-0.01
		814	TC370071	UP|Q8H6A5_MAIZE (Q8H6A5) Translationally controlled tumor protein-like protein		-0.02
		835	TC330220	RF|NP_567410.1|18414065|NM_117446 hydrolase/ pyrophosphatase {Arabidopsis thaliana}, 96%		-0.01
		872	TC362570	Zea mays clone EL01N0560B10.c mRNA sequence	-0.01	-0.01
		883	TC325586	UP|Q84PA9_ORYSA (Q84PA9) Sarcoplasmic reticulum protein (With alternative splicing), 78%		-0.01
		913	TC323432	GB|AAF50128.1|7294794|AE003547 CG7949-PA {Drosophila melanogaster}, 42%		-0.01

918	TC355837	UP|Q5VRJ8_ORYSA (Q5VRJ8) Peroxisomal biogenesis factor 11 protein-like, 98%		-0.01
1054	TC328713		-0.01	
1071	TC338143	UP|Q94DF0_ORYSA (Q94DF0) Pleckstrin homology (PH) domain-containing protein-like, 88%		0.01
1141	TC370630	Zea mays clone Contig384 mRNA sequence	-0.01	
1354	TC316993	UP|Q5VQP7_ORYSA (Q5VQP7) Leucine-rich repeat protein, 94%	-0.01	
1400	TC343036	RF|NP_193367.2|42566868|NM_117729 SPK1 (SPIKE1) {Arabidopsis thaliana}, 30%		-0.01
1569	TC354693	UP|Q9FMW9_ARATH (Q9FMW9) Farnesyl cysteine carboxyl methyltransferase-like protein, 22%	-0.01	
1643	TC319161		-0.01	
1781	TC357501			-0.02
1854	TC340710	UP|Q53AN3_ORYSA (Q53AN3) Gibberellin-stimulated protein, 75%		-0.01
1954	TC320811	UP|Q93Z42_ARATH (Q93Z42) AT5g19750/T29J13_170, 56%	-0.01	
2006	TC325611	UP|Q9SYL3_ARATH (Q9SYL3) F3F20.17 protein, 80%		-0.01
2126	TC357696	UP|Q84UA5_MAIZE (Q84UA5) Liguleless2-like protein	-0.01	
2348	TC331987	UP|Q2QKB8_WHEAT (Q2QKB8) Alternative splicing regulator, 62%	-0.01	
2371	TC323589	UP|Q564K3_ARATH (Q564K3) Chromosome associate protein subunit H, 18%		0.01
2408	TC319161			-0.01
2443	TC328798	UP|Q3MST6_ORYSA (Q3MST6) Peptidyl prolyl cis-trans isomerase	-0.01	
2683	TC337690	UP|Q9SUL3_ARATH (Q9SUL3) OBP33PEP like protein, 59%		0.01
2713	TC358978	UP|Q9M584_MAIZE (Q9M584) Hypersensitive-induced response protein, 21%		0.01
3030	TC326203	UP|Q5N8G9_ORYSA (Q5N8G9) QUAKING isoform 5-like		0.01
3033	TC338926	UP|Q337S2_ORYSA (Q337S2) HR-like lesion-inducing, 78%	-0.01	

3225	TC334113	GB|AAD56315.1|5923664|ATAC009326 Yippee-like protein {Arabidopsis thaliana}, 44%	-0.01	
3528	TC343708	UP|Q8RUF8_ARATH (Q8RUF8) AT5g12040/F14F18_210, 46%		0.01
3626	TC345274	UP|Q7XQH1_ORYSA (Q7XQH1) OJ000114_01.6 protein, 10%	-0.01	
3751	TC321807		-0.01	
3774	TC353250			0.01
3804	TC329354	RF|NP_567847.1|18417605|NM_119195 ATPase {Arabidopsis thaliana}, 66%	0.01	
3837	TC327577	RF|NP_195140.2|30689939|NM_119577 nucleic acid binding {Arabidopsis thaliana}, 37%		0.01
4237	TC370251	UP|G10A_ORYSA (Q94DE2) Protein G10 homolog 1		0.01
4243	TC324206	UP|Q67U13_ORYSA (Q67U13) Translocation protein-related-like, 90%		0.01
4269	TC331921	UP|Q940S3_ARATH (Q940S3) At1g31070/F17F8_1, 51%		0.01
4295	TC363760	UP|Q69SJ5_ORYSA (Q69SJ5) NatC N(Alpha)-terminal acetyltransferase, Mak10 subunit-like, 32%		0.01
4322	TC343175	RF|NP_191890.1|15229411|NM_116196 glutamate binding {Arabidopsis thaliana}, 60%		0.01
4345	TC351372	UP|EBP2_ARATH (Q9LUJ5) Probable rRNA-processing protein EBP2 homolog, 58%		0.02
4357	TC327629	RF|XP_507481.1|51979234|XM_507481 OJ1342_D02.8 gene product {Oryza sativa (japonica cultivar-group)}, 82%		0.01
4480	TC338289	UP|Q6ZFI5_ORYSA (Q6ZFI5) Parathymosin-like, 51%	-0.01	
4488	TC328820	UP|Q8H2M0_ORYSA (Q8H2M0) KH domain-like protein, 32%		-0.01
4612	TC316196	UP|Q9M588_MAIZE (Q9M588) Prohibitin	-0.01	
4640	TC337053	UP|Q287W4_OLIPU (Q287W4) Transducin/WD-40 repeat family protein, 34%	-0.01	
4642	-	-		0.01
4669	TC318294	UP|Q337S2_ORYSA (Q337S2) HR-like lesion-inducing	-0.01	
4733	TC331439	RF|NP_850560.1|30681809|NM_180229 catalytic {Arabidopsis thaliana}, 64%		0.01

4742	TC316827	UP|Q5UDB6_MAIZE (Q5UDB6) INDETERMINATE-related protein 9		0.01
4749	TC331877	RF|XP_507328.1|51965088|XM_507328 OJ1125_C01.28 gene product {Oryza sativa (japonica cultivar-group)}, 31%		0.01
4775	TC335870	RF|NP_195600.1|15234862|NM_120049 FAD binding {Arabidopsis thaliana}, 18%		0.01
4780	TC326843	RF|NP_849992.1|30680811|NM_179661 RNA binding {Arabidopsis thaliana}, 35%		0.01
4826	TC369551	Zea mays clone Contig690 mRNA sequence	0.01	
4843	TC324875	RF|NP_850027.2|42570301|NM_179696 oxidoreductase {Arabidopsis thaliana}, 62%	-0.01	
4924	TC341434			0.01
4994	TC335804	Zea mays clone Contig94.F mRNA sequence		0.01
5286	TC345192	RF|NP_564718.2|30696124|NM_104525 catalytic/ hydrolase {Arabidopsis thaliana}, 20%		0.01
5327	TC340710	UP|Q53AN3_ORYSA (Q53AN3) Gibberellin-stimulated protein, 75%		-0.01
5525	TC331860	UP|Q5YJL8_HYAOR (Q5YJL8) Hydrolase, 84%	0.01	
5531	TC316797	Zea mays clone Contig158 mRNA sequence		0.01
5575	TC330725			0.01
5665	TC338267	GB|AAP37787.1|30725530|BT008428 At4g30200 {Arabidopsis thaliana}, 13%		0.01
5707	TC328339	UP|O22875_ARATH (O22875) Expressed protein, 83%	0.01	0.01
5795	TC344758	UP|Q2R2T0_ORYSA (Q2R2T0) Maf-like protein, 42%		0.01
5934	TC342248	GB|AAO63368.1|28950889|BT005304 At2g48120 {Arabidopsis thaliana}, 73%		0.01
6011	TC366841	UP|Q6K448_ORYSA (Q6K448) SWIB complex BAF60b domain-containing protein-like, 87%	-0.01	
6020	TC328339	UP|O22875_ARATH (O22875) Expressed protein, 83%	-0.02	
6103	TC345447	UP|YIPL_SOLTU (P59234) Protein yippee-like		0.01
6189	TC344758	UP|Q2R2T0_ORYSA (Q2R2T0) Maf-like protein, 42%		0.01

6318	TC348095	UP|Q67YS7_ARATH (Q67YS7) MRNA, complete cds, clone: RAFL24-10-D10, 11%		0.01
6624	TC330434	UP|Q6H5H5_ORYSA (Q6H5H5) Emsy N terminus domain-containing protein-like, 87%		0.01
6761	TC331782			-0.01
6860	-	-		-0.01
6899	TC330220	RF|NP_567410.1|18414065|NM_117446 hydrolase/ pyrophosphatase {Arabidopsis thaliana}, 96%		-0.01
6908	TC335188	UP|Q7M4Q5_HUMAN (Q7M4Q5) Basic proline-rich peptide IB-8a, 36%		-0.01
7307	TC363410	UP|Q6NMK2_ARATH (Q6NMK2) At5g49400, 66%	-0.01	
7436	TC355836	UP|Q9ZWT4_IPOPU (Q9ZWT4) Transposase, 8%		0.01
7460	TC341608	RF|XP_507328.1|51965088|XM_507328 OJ1125_C01.28 gene product {Oryza sativa (japonica cultivar-group)}, 57%	-0.01	
7709	TC321340	UP|Q9SC83_NICPL (Q9SC83) VAP27, 80%		0.01
7838	TC364594	UP|Q2QTL0_ORYSA (Q2QTL0) Expressed protein, 98%		0.01
7858	TC326902			0.01
7949	TC343279	RF|NP_201147.2|42568733|NM_125737 EMB2746 catalytic {Arabidopsis thaliana}, 34%		0.01
8226	TC328257	UP|Q337C1_ORYSA (Q337C1) Expressed protein, 66%		-0.01
8369	TC325902	UP|Q7F241_ORYSA (Q7F241) Speckle-type POZ protein-like protein		-0.01
8433	TC316694	UP|Q306I3_HORVU (Q306I3) Reticulon, 97%		0.01
8473	TC336091	UP|Q9M578_ORYSA (Q9M578) XIG, 95%		0.01
8489	TC330528	UP|Q9LIL4_ARATH (Q9LIL4) Coated vesicle membrane protein-like (AT3g22845/MWI23_22), 87%		-0.01
8565	TC319484		0.01	
8574	TC318488	UP|Q7XI46_ORYSA (Q7XI46) Hydrolase-like protein, 72%	-0.01	

8741	TC316512	Zea mays clone EL01N0529A02.c mRNA sequence		-0.01
8812	TC360634	RF|XP_507481.1|51979234|XM_507481 OJ1342_D02.8 gene product {Oryza sativa (japonica cultivar-group)}, 94%	-0.01	
9080	TC331439	RF|NP_850560.1|30681809|NM_180229 catalytic {Arabidopsis thaliana}, 64%		0.01
9192	TC323978	UP|Q5UDB7_MAIZE (Q5UDB7) INDETERMINATE-related protein 7		0.01
9329	TC326817	UP|Q6V9I5_SOLCH (Q6V9I5) P18, 62%		0.01
9439	TC316482			-0.01
9522	TC326902		0.01	0.01
9525	TC352408	UP|Q9M145_ARATH (Q9M145) Predicted protein, 17%		0.01
9651	TC336764	UP|Q2QX79_ORYSA (Q2QX79) Nrap protein, 23%		-0.02
9691	TC341160	UP|Q6K478_ORYSA (Q6K478) Integral membrane protein-like		-0.01
9742	TC323434	UP|Q940M0_ARATH (Q940M0) At1g63980/F22C12_9, 45%	0.01	
9816	TC348425	GB|AAT77840.1|50540683|AC147426 expressed protein {Oryza sativa (japonica cultivar-group)}, 89%		-0.01
10308	TC368973	UP|Q7Y031_ARATH (Q7Y031) At1g10030, 42%	-0.01	
10419	TC332272	UP|MGN_ORYSA (P49030) Protein mago nashi homolog, 92%		-0.01
10478	TC324512	UP|Q6K1Q5_ORYSA (Q6K1Q5) Glycolipid transfer protein-like		0.01
10521	TC326649	GB|CAB38784.1|4490293|ATF17M5 WD-repeat protein-like protein {Arabidopsis thaliana}, 59%		0.01
10601	TC340570	UP|Q337C1_ORYSA (Q337C1) Expressed protein, 47%		-0.01
10658	TC329745	UP|Q2RAY1_ORYSA (Q2RAY1) Expressed protein, 97%		0.02
10814	TC337887	UP|Q6Z3Y5_ORYSA (Q6Z3Y5) PWWP domain protein-like, 43%		0.01
10935	TC337996	UP|S1FA2_ORYSA (Q7XLX6) DNA-binding protein S1FA2		0.01

11166	TC331544	UP|Q2QLN8_ORYSA (Q2QLN8) Expressed protein, 85%		0.01
11207	TC326846			-0.01
11233	TC320041	UP|Q5QMR4_ORYSA (Q5QMR4) Integral membrane protein-like, 97%		-0.01
11263	TC323610	UP|Q8LR49_ORYSA (Q8LR49) Nuclear LIM factor interactor-interacting protein hyphal form-like, 89%		-0.01
11279	TC335804	Zea mays clone Contig94.F mRNA sequence	-0.01	
11300	TC322293	UP|Q5N9K2_ORYSA (Q5N9K2) BAG domain containing protein-like, 90%	-0.01	-0.01
11384	TC325586	UP|Q84PA9_ORYSA (Q84PA9) Sarcoplasmic reticulum protein (With alternative splicing), 78%		-0.01
11646	TC333661	UP|Q6K3S8_ORYSA (Q6K3S8) Prefoldin-like, 89%		-0.01
11664	TC341160	UP|Q6K478_ORYSA (Q6K478) Integral membrane protein-like		-0.01
11751	TC335566	UP|Q6DBH4_ARATH (Q6DBH4) At2g23820, 70%	0.01	
11766	TC337996	UP|S1FA2_ORYSA (Q7XLX6) DNA-binding protein S1FA2	0.01	-0.01
11777	TC323899	RF|NP_174226.1|15218889|NM_102672 nucleotide binding {Arabidopsis thaliana}, 38%	0.01	-0.01
11779	TC325392	UP|Q3E7S6_ARATH (Q3E7S6) Protein At5g53050, 65%	0.01	
11825	TC332589	UP|Q93VM9_ARATH (Q93VM9) AT5g09860/MYH9_7, 52%		-0.01
11835	TC323753	RF|NP_188244.2|42564243|NM_112494 nucleic acid binding {Arabidopsis thaliana}, 32%		-0.01
11885	TC319363	UP|Q6NL07_ARATH (Q6NL07) At1g13820, 50%		0.01
12032	TC338652	UP|Q8W065_ORYSA (Q8W065) Nuclear movement protein-like, 89%		0.01
12062	TC358760	UP|Q8L6E9_PEA (Q8L6E9) GDA2 protein, 70%	0.01	
12185	TC337996	UP|S1FA2_ORYSA (Q7XLX6) DNA-binding protein S1FA2		0.01
12327	TC371750	UP|Q4F886_ORYSA (Q4F886) DT-related protein, 77%		0.01
12376	TC330994	RF|NP_187695.3|42563999|NM_111921 catalytic/ hydrolase {Arabidopsis thaliana}, 52%		0.01

12444	TC317050	Zea mays clone Contig43 mRNA sequence		-0.01
12468	TC328339	UP|O22875_ARATH (O22875) Expressed protein, 83%		-0.01
12557	TC317564	Zea mays clone EL01N0520H08.c mRNA sequence	0.01	
12720	TC360690	UP|Q8W065_ORYSA (Q8W065) Nuclear movement protein-like		0.01
12736	TC324408	GB|AAT72926.1|50293119|AY656247 17.7 kDa low temperature induced protein {Oryza sativa (japonica cultivar-group)}, 30%		0.01
12744	TC322693	UP|Q337S2_ORYSA (Q337S2) HR-like lesion-inducing	0.02	-0.02
12776	TC351863	GB|CAA66406.1|1495267|ATORF12 {Arabidopsis thaliana}, 8%		0.01
12807	TC358535	Zea mays clone EL01N0314F02.c mRNA sequence		-0.01
12850	TC364270	UP|Q761Y3_ORYSA (Q761Y3) BRI1-KD interacting protein 132, 13%	0.01	
12937	TC331260	UP|Q84WJ1_ARATH (Q84WJ1) At5g49570, 16%	0.02	
12981	TC353880	UP|Q5N9Q7_ORYSA (Q5N9Q7) Peptidyl-tRNA hydrolase-like, 60%	0.01	
12986	TC338036	UP|Q9XHE1_MAIZE (Q9XHE1) CENPCA protein, 17%	0.01	0.01
13165	TC335999	RF|NP_001031176.1|79319792|NM_001036099 catalytic/ hydrolase {Arabidopsis thaliana}, 79%	0.02	
13217	TC317154	Zea mays clone EL01N0372A05.c mRNA sequence	0.01	0.01
13436	TC323254	RF|NP_850385.1|30689259|NM_180054 calmodulin binding {Arabidopsis thaliana}, 72%		-0.02
14209	TC343018	UP|Q2VCI0_SOLTU (Q2VCI0) Translocon-associated protein beta family protein-like, 70%	0.01	
14385	TC341889	gb|K02202.1|MZERG17S Maize 17S ribosomal RNA gene and flanks, 32%	0.01	-0.02
14512	TC356728	UP|Q337R1_ORYSA (Q337R1) Expressed protein, 34%	0.01	
14649	TC345176	UP|Q2R2S2_ORYSA (Q2R2S2) Maf-like protein, 85%	0.01	
14852	TC327316	GB|AAR28019.1|39545912|AY463617 TAF14b {Arabidopsis thaliana}, 39%		0.01
14862	TC369948	UP|Q9FFQ2_ARATH (Q9FFQ2) Alcohol dehydrogenase-like protein, 19%		0.01

14976	TC354470	UP|Q6EPQ3_ORYSA (Q6EPQ3) SPX (SYG1/Pho81/XPR1) domain-containing protein-like, 48%		-0.02
15032	TC325830	UP|Q69NN6_ORYSA (Q69NN6) Mechanosensitive ion channel domain-containing protein-like, 84%	0.01	
15601	TC332008	GB|CAB38784.1|4490293|ATF17M5 WD-repeat protein-like protein {Arabidopsis thaliana}, 69%		-0.01
978	TC350910			-0.01
2655	TC342589	UP|Q9SR28_ARATH (Q9SR28) F3L24.22 protein, 9%	-0.01	
9239	TC343712	UP|Q2QAV0_ARATH (Q2QAV0) Fused, 19%	-0.01	0.01
4487	TC341610		-0.01	
9214	TC341610		-0.01	
10674	TC350378	GB|AAC04627.1|2920837|OSU95135 a novel fungal inducible gene {Oryza sativa}, 57%		0.01
7045	TC333055	UP|Q4V398_ARATH (Q4V398) At5g15740, 64%		0.01
14	TC365456	UP|Q9SDN6_TOBAC (Q9SDN6) FH protein NFH1, 12%		-0.01
5881	TC334427	UP|Q69MT2_ORYSA (Q69MT2) Diaphanous protein-like, 48%		0.01
1363	TC326117	RF|XP_507202.1|51964836|XM_507202 P0488B06.44 gene product {Oryza sativa (japonica cultivar-group)}, 76%		-0.02
3017	-	-		-0.01
7398	TC320122	RF|XP_507202.1|51964836|XM_507202 P0488B06.44 gene product {Oryza sativa (japonica cultivar-group)}, 91%		0.01
8576	TC323491	GB|AAT77876.1|50540720|AC103550 expressed protein {Oryza sativa (japonica cultivar-group)}, 96%		-0.01
857	TC340260	Zea mays clone Contig275 mRNA sequence		-0.01
1035	TC347109		-0.01	
1426	TC338962	UP|Q53Q87_ORYSA (Q53Q87) Expressed protein, 28%		-0.01

	2265	TC341680			-0.01
	9570	TC357549	UP|Q2QYR0_ORYSA (Q2QYR0) Expressed protein, 21%		-0.01
	11974	TC336786	UP|Q66GI4_ARATH (Q66GI4) At2g32230, 36%		0.01
	10924	TC355698	RF|NP_917278.1|34911862|NM_192389 pherophorin - like protein {Oryza sativa (japonica cultivar-group)}, 32%		0.01
	8833	TC338708	UP|Q8LLB9_HORVU (Q8LLB9) BPM, 51%		0.01
	7035	TC336515	UP|Q9LHM6_ARATH (Q9LHM6) Pre-rRNA processing protein RRP5, 13%		0.01
	4376	TC342816	UP|Q852K2_ORYSA (Q852K2) Expressed protein, 39%		0.01
Unknown	3	TC329095	UP|ZEB2_MAIZE (P08031) Zein-beta precursor	-0.01	
	8	TC321682	UP|Q8S2G4_ORYSA (Q8S2G4) Mucin-like protein, 86%	-0.01	
	28	TC334537			-0.01
	30	TC339858	UP|Q9LK32_ARATH (Q9LK32) Gb|AAF03438.1 (AT3g27210/K17E12_3), 16%		-0.01
	41	TC327546	UP|Q9SXG0_ORYSA (Q9SXG0) F1F0-ATPase inhibitor protein, 93%		-0.01
	240	TC339396	GB|1WVK_A|58177252|1WVK_A Chain A, Nmr Solution Structure Of The Partially Disordered Protein At2g23090 From {Arabidopsis thaliana}, 63%	-0.01	-0.01
	402	TC338766	Zea mays clone EL01N0302D08.c mRNA sequence		-0.01
	424	TC323761	RF|NP_496363.1|17533631|NM_063962 LEThal family member (let-858) {Caenorhabditis elegans}, 3%		-0.01
	442	TC350359	RF|NP_680117.2|30693041|NM_148864 EMB2423 {Arabidopsis thaliana}, 31%	-0.01	
	448	TC342886	UP|Q8RX56_ARATH (Q8RX56) AT5g06970/MOJ9_14, 10%		-0.02
	533	TC316503			0.01
	572	TC323761	RF|NP_496363.1|17533631|NM_063962 LEThal family member (let-858) {Caenorhabditis elegans}, 3%	-0.01	-0.01

767	TC319724			-0.02
774	TC328007	UP|Q7XCR5_ORYSA (Q7XCR5) Expressed protein, 93%	-0.01	
806	TC340730	GB|AAL16180.1|16226487|AF428412 At3g07760/F17A17.10 {Arabidopsis thaliana}, 94%		-0.01
810	TC324333	UP|Q84VS2_SOLTU (Q84VS2) Potyviral helper component protease-interacting protein 2, 33%		-0.01
897	TC326450			-0.01
928	TC332518			-0.01
1006	TC349780	UP|Q7XBW2_ORYSA (Q7XBW2) Expressed protein, 92%	-0.01	
1014	TC342660	UP|Q7M3Z4_ILLAR (Q7M3Z4) Sperm chromatin protein I2-1, 46%		-0.01
1015	TC334944	GB|AAP13387.1|30023708|BT006279 At3g52220 {Arabidopsis thaliana}, 59%		-0.01
1050	TC346553	Zea mays clone EL01N0501D07.c mRNA sequence	-0.01	-0.01
1056	TC349178		-0.01	-0.01
1059	TC336955	UP|Q9LIM4_ARATH (Q9LIM4) Arabidopsis thaliana genomic DNA, chromosome 3, BAC clone:F4B12, 39%	-0.01	-0.01
1143	TC346921	UP|Q6ATQ2_ORYSA (Q6ATQ2) Expressed protein, 67%		0.01
1171	TC341254		-0.01	
1172	TC343206		-0.01	
1289	TC359074			-0.01
1482	TC352561	UP|Q84T74_ORYSA (Q84T74) Expressed protein, 62%		-0.01
1541	TC317638	RF|XP_507493.1|51979262|XM_507493 OJ1058_F07.21 gene product {Oryza sativa (japonica cultivar-group)}, 82%		-0.01
1681	TC324604	Zea mays clone Contig497.F mRNA sequence	-0.01	
1719	TC354704	UP|Q75L86_ORYSA (Q75L86) BLE1 protein, 91%	-0.01	

1747	TC336840	UP|Q2QZT8_ORYSA (Q2QZT8) Expressed protein, 54%	-0.01	
1799	TC355112		-0.01	
1826	TC328201	UP|Q761Z6_ORYSA (Q761Z6) BRI1-KD interacting protein 118, 46%		-0.01
1896	TC341879	UP|Q75GV2_ORYSA (Q75GV2) Expressed protein, 88%	0.02	-0.01
1962	TC341505	UP|Q33A24_ORYSA (Q33A24) Expressed protein, 3%	-0.01	
1965	TC358004	UP|Q29SB6_MAIZE (Q29SB6) Pathogenesis-related protein 10	-0.01	
2039	TC328277			-0.01
2095	TC319490		-0.01	
2122	TC327546	UP|Q9SXG0_ORYSA (Q9SXG0) F1F0-ATPase inhibitor protein, 93%	-0.01	
2145	TC331928	GB|AAL16180.1|16226487|AF428412 At3g07760/F17A17.10 {Arabidopsis thaliana}, 94%		0.01
2246	TC318123	Zea mays clone EL01N0557A09.c mRNA sequence	-0.01	
2338	TC332875	UP|Q9LF51_ARATH (Q9LF51) Glutamine-rich protein, 49%		0.01
2419	TC329244	UP|Q2QW42_ORYSA (Q2QW42) Expressed protein, 92%	-0.01	
2468	TC330431	UP|Q4RD34_TETNG (Q4RD34) Chromosome undetermined SCAF17449, whole genome shotgun sequence, 19%	-0.01	
2504	TC340524	UP|Q75ID5_ORYSA (Q75ID5) Expressed protein, 85%	-0.01	
2506	TC332852		-0.01	
2562	TC320051	UP|Q9SXG0_ORYSA (Q9SXG0) F1F0-ATPase inhibitor protein, 93%	-0.01	
2593	TC354908	UP|Q6ST18_MAIZE (Q6ST18) Heat shock factor binding protein 2		0.01
2619	TC340506	UP|Q4ABY1_BRARP (Q4ABY1) 4D11_6, 47%		0.01
2688	TC345002			0.01
2786	TC338074	UP|Q6AVF2_ORYSA (Q6AVF2) Expressed protein, 35%	-0.01	

2883	TC347567	UP|Q5BS67_SCHJA (Q5BS67) SJCHGC05844 protein, 59%		-0.01
2892	TC338905			-0.01
2973	TC332518		-0.01	
3093	TC355900	UP|Q75GR6_ORYSA (Q75GR6) Ribosomal protein L17-like protein, 16%	-0.01	
3132	TC346226	UP|Q9SGX1_ARATH (Q9SGX1) F1N19.1, 25%		0.01
3135	TC329338	UP|Q6AVF2_ORYSA (Q6AVF2) Expressed protein, 35%	-0.02	
3178	TC316335	Zea mays clone EL01N0515E01.d mRNA sequence		0.01
3297	TC351715	UP|Q7XDT2_ORYSA (Q7XDT2) Ribosomal rna assembly protein mis3, 94%		-0.01
3327	TC317000	UP|Q5YJL6_HYAOR (Q5YJL6) Autophagy protein AGP6, 94%		-0.01
3458	TC341024		-0.01	
3568	TC361335	UP|P93630_MAIZE (P93630) HMGc1 protein	0.01	
3624	TC366481		0.02	-0.02
3680	TC345044	UP|Q5JME7_ORYSA (Q5JME7) Lysine ketoglutarate reductase trans-splicing related 1-like, 89%	-0.01	
3683	TC335333		-0.01	-0.01
3823	TC318403	Zea mays clone EL01T0201D03.c mRNA sequence	0.01	
3903	TC333376	UP|Q6YUB8_ORYSA (Q6YUB8) Major intrinsic protein-like, 50%		0.01
4124	TC327906	UP|Q5YLM3_MAIZE (Q5YLM3) Roothairless 1		0.01
4135	TC342294	UP|Q5GAS0_MAIZE (Q5GAS0) Tac7077, 89%		0.01
4156	TC320124	UP|Q8LQG0_ORYSA (Q8LQG0) Leaf senescence protein-like, 64%	-0.01	
4218	TC321357		-0.01	
4229	TC324604	Zea mays clone Contig497.F mRNA sequence		0.01

4262	TC342732	UP|Q2V2V7_ARATH (Q2V2V7) Protein At5g64400, 33%	0.01	
4522	TC343231	UP|Q945Q5_ARATH (Q945Q5) At2g30700/T11J7.9 (Expressed protein), 18%		0.01
4615	TC335637	UP|Q2V2V7_ARATH (Q2V2V7) Protein At5g64400, 48%	-0.01	
4617	TC339594	GB|AAC72966.1|3860049|AF096867 synapsin IIb {Mus musculus}, 4%	-0.01	
4684	TC326941	UP|Q6YZC6_ORYSA (Q6YZC6) Cation exchanger-like protein, 98%	0.01	
4774	TC343714			0.01
4903	TC321966	UP|Q6K819_ORYSA (Q6K819) MADS box interactor-like, 80%	0.01	
5110	TC324583	GB|AAC28224.1|3377842|T27D20 {Arabidopsis thaliana}, 70%	0.02	
5279	TC345686			0.01
5336	TC340890	UP|O22632_MAIZE (O22632) Nitrate-induced NOI protein		0.01
5644	TC323761	RF|NP_496363.1|17533631|NM_063962 LEThal family member (let-858) {Caenorhabditis elegans}, 3%	-0.01	
5664	TC326000			0.01
5665	TC329577	UP|Q2QPD5_ORYSA (Q2QPD5) Expressed protein, 6%		0.01
5685	TC320051	UP|Q9SXG0_ORYSA (Q9SXG0) F1F0-ATPase inhibitor protein, 93%		0.01
6012	TC350167	GB|BAD06873.1|40714351|AB111915 replication protein A 14kDa {Oryza sativa (japonica cultivar-group)}, 92%	-0.01	
6019	TC341103	UP|Q4T4D2_TETNG (Q4T4D2) Chromosome undetermined SCAF9708, whole genome shotgun sequence, 3%	-0.02	
6056	TC316538	Zea mays clone Contig600 mRNA sequence	-0.01	
6086	TC340355	UP|Q1XA87_WHEAT (Q1XA87) Salt tolerant protein		0.01
6145	TC329567	UP|Q8RUX8_ARATH (Q8RUX8) Expressed protein, 82%		0.01
6165	TC320904	UP|Q3G2T7_9DELT (Q3G2T7) Magnesium chelatase, ChlI subunit, 3%		0.01

6167	TC317000	UP|Q5YJL6_HYAOR (Q5YJL6) Autophagy protein AGP6, 94%		0.01
6297	TC330062	GB|AAQ89625.1|37202020|BT010603 At4g21720 {Arabidopsis thaliana}, 73%		0.01
6342	TC334850			0.01
6457	TC331670	GB|AAP21210.1|30102584|BT006402 At5g36710 {Arabidopsis thaliana}, 55%		0.01
6495	TC321853	Zea mays clone EL01N0553G07.c mRNA sequence	0.02	
6510	TC320447	UP|Q6NM29_ARATH (Q6NM29) At2g15730, 56%		0.01
6520	TC348434	small subunit ribosomal protein S28		0.01
6566	TC354908	UP|Q6ST18_MAIZE (Q6ST18) Heat shock factor binding protein 2		0.01
6625	TC343411	UP|Q304B1_ARATH (Q304B1) Protein At3g48380, 39%		0.01
6813	TC319714	Zea mays clone EL01N0407C06.c mRNA sequence	-0.01	
6962	-	-		-0.01
7121	TC351706	UP|Q9XGD8_MAIZE (Q9XGD8) BETL4 protein precursor	-0.01	
7256	TC334537			0.01
7313	TC328601	UP|Q1YJ75_9RHIZ (Q1YJ75) PAS sensor histidine kinase/response regulator, 3%	-0.01	
7316	TC318989	GB|AAL28159.1|16767882|AY060611 GH03581p {Drosophila melanogaster}, 11%		0.01
7534	TC334510		0.01	
7638	TC328306	UP|Q8L7Z1_ARATH (Q8L7Z1) AT5g16250/T21H19_170, 58%		0.01
7695	TC332602			-0.02
7753	-	-		0.01
7938	TC339873	RF|XP_474252.1|50929449|XM_474252 {Oryza sativa (japonica cultivar-group)}, 42%	0.01	
8028	TC343208	UP|Q6IDB8_ARATH (Q6IDB8) At5g26800, 38%		0.01

8044	TC351248	UP|UFM1_ORYSA (Q94DM8) Probable ubiquitin-fold modifier 1 precursor, 58%		-0.01
8068	TC316091	Zea mays clone Contig411.F mRNA sequence		-0.01
8123	TC321966	UP|Q6K819_ORYSA (Q6K819) MADS box interactor-like, 80%	0.01	
8223	TC327180	UP|Q64HC0_MAIZE (Q64HC0) ASF/SF2-like pre-mRNA splicing factor SRP30	0.01	
8241	TC352948	UP|Q2XX25_ZEAMP (Q2XX25) Phospholipid transfer protein 1	0.02	
8397	TC364375	UP|Q4RPN5_TETNG (Q4RPN5) Chromosome 12 SCAF15007, whole genome shotgun sequence., 18%	-0.01	
8453	TC334828	UP|O46132_LOCMI (O46132) Nicotinic acetylcholine receptor, alpha1 subunit, 3%	-0.01	
8463	TC321976			-0.01
8467	TC320051	UP|Q9SXG0_ORYSA (Q9SXG0) F1F0-ATPase inhibitor protein, 93%		0.01
8488	TC340524	UP|Q75ID5_ORYSA (Q75ID5) Expressed protein, 85%		-0.01
8490	TC326656	UP|Q9FZF0_ARATH (Q9FZF0) T2E6.19, 50%	-0.01	
8540	-	-		0.01
8566	TC320124	UP|Q8LQG0_ORYSA (Q8LQG0) Leaf senescence protein-like, 64%	0.01	
8705	TC339653	GB|BAA11822.1|1542809|D83144 Six3a {Mus musculus}, 6%		0.02
8776	TC324793			0.01
8861	TC350092			0.01
8932	TC364874	UP|Q2HTU7_MEDTR (Q2HTU7) Uncharacterized Cys-rich domain, 9%		0.01
8983	TC329841			-0.01
9194	TC351177	UP|Q6H7J8_ORYSA (Q6H7J8) Transcription factor-like, 10%		0.01
9257	TC345977	UP|Q6KA74_ORYSA (Q6KA74) Ankyrin repeat protein-like, 53%		0.01
9312	TC334537			0.01

9335	-	-	-0.01	0.01
9364	TC330044	GB|AAN73294.1|25141199|BT002297 At5g12010/F14F18_180 {Arabidopsis thaliana}, 34%		0.02
9501	TC331757	UP|Q53PH6_ORYSA (Q53PH6) Expressed protein, 28%	0.01	
9608	TC325833	Zea mays clone EL01T0403C05.c mRNA sequence		-0.01
9620	TC351878	UP|ELOF1_ORYSA (Q8LHP0) Transcription elongation factor 1 homolog		0.01
9668	TC345882		-0.01	
9686	TC318327	UP|Y3377_ARATH (Q6ID70) Protein At3g03773, 68%	0.02	
9718	TC316721	UP|Q3ED49_ARATH (Q3ED49) Protein At1g29980, 82%		-0.01
9779	TC335114			-0.01
9825	TC319292			-0.01
9916	TC353961	UP|O64572_ARATH (O64572) Expressed protein, 3%		-0.01
9931	TC322521	UP|Q2QQ99_ORYSA (Q2QQ99) Expressed protein	0.02	
9947	TC340418	GB|AAP37753.1|30725462|BT008394 At4g28200 {Arabidopsis thaliana}, 13%	0.01	
10032	TC334944	GB|AAP13387.1|30023708|BT006279 At3g52220 {Arabidopsis thaliana}, 59%	-0.01	
10041	TC345598	GB|AAL16180.1|16226487|AF428412 At3g07760/F17A17.10 {Arabidopsis thaliana}, 90%	-0.01	-0.01
10095	TC342440	UP|YB1E_SCHPO (P87179) Serine-rich protein C30B4.01c precursor, 7%	-0.01	
10096	TC344393	UP|Q8IQY7_DROME (Q8IQY7) CG32552-PA, 9%		0.01
10107	TC329862	UP|Q9LR90_ARATH (Q9LR90) T23E23.20, 55%		-0.02
10145	TC324583	GB|AAC28224.1|3377842|T27D20 {Arabidopsis thaliana}, 70%		-0.02
10167	TC319800	UP|Q6AST9_ORYSA (Q6AST9) Expressed protein, 78%	-0.01	
10324	TC331186	UP|Q2T6H4_BURTA (Q2T6H4) Mte8-like protein, 5%	-0.01	-0.01

10329	TC358439	UP|Q9LH72_ARATH (Q9LH72) Arabidopsis thaliana genomic DNA, chromosome 3, BAC clone: T21E2, 22%	0.01	0.01
10352	TC317710			-0.01
10371	TC338148	UP|Q6Z756_ORYSA (Q6Z756) Erwinia induced protein 2	-0.01	
10461	TC321561	UP|Q2V3X6_ARATH (Q2V3X6) Protein At3g07565, 64%		0.01
10481	TC316091	Zea mays clone Contig411.F mRNA sequence		0.01
10520	TC348548			0.01
10642	TC347242	UP|Q7VCK3_PROMA (Q7VCK3) Porin homolog, 4%		0.01
10659	TC355940			0.01
10751	TC316538	Zea mays clone Contig600 mRNA sequence		-0.01
10768	TC358226	UP|Q8HYH9_MACEU (Q8HYH9) Type I interferon, 33%	-0.01	
10822	TC326115	UP|Q94A30_ARATH (Q94A30) At1g55500/T5A14_10, 35%		0.01
10833	TC341103	UP|Q4T4D2_TETNG (Q4T4D2) Chromosome undetermined SCAF9708, whole genome shotgun sequence, 3%		-0.01
10849	TC325818	UP|Q6NM29_ARATH (Q6NM29) At2g15730, 67%		0.01
10870	TC354456			0.01
10940	TC325872	GB|AAP54551.1|31432987|AE016959 expressed protein {Oryza sativa (japonica cultivar-group)}, 58%	0.01	
10963	TC332852		-0.01	
11032	TC369763	GB|AAA74598.1|598099|CSSPRP protamine P1 {Caenolestes fuliginosus}, 49%		-0.01
11057	TC334869	UP|O74346_SCHPO (O74346) SPBC21D10.06c protein, 3%		0.01
11150	TC352754	UP|Q9XI88_ARATH (Q9XI88) F7A19.8 protein, 33%		-0.01
11177	TC354635			-0.01

11269	TC326383	UP|Q8S2Y8_MAIZE (Q8S2Y8) Glycine-rich RNA binding protein		-0.01
11501	TC342660	UP|Q7M3Z4_ILLAR (Q7M3Z4) Sperm chromatin protein I2-1, 46%	0.01	
11546	TC362889	Zea mays clone EL01N0312G06.c mRNA sequence		-0.01
11690	TC316721	UP|Q3ED49_ARATH (Q3ED49) Protein At1g29980, 82%	-0.01	
12001	TC327546	UP|Q9SXG0_ORYSA (Q9SXG0) F1F0-ATPase inhibitor protein, 93%	-0.01	
12058	TC341103	UP|Q4T4D2_TETNG (Q4T4D2) Chromosome undetermined SCAF9708, whole genome shotgun sequence, 3%	-0.02	
12095	TC334059		-0.01	
12179	TC328374		-0.01	
12260	TC336554	RF|XP_805306.1|71405362|XM_800213 neurobeachin/beige protein {Trypanosoma cruzi strain CL Brener}, 3%	-0.01	-0.01
12324	TC324782	Zea mays clone EL01N0440C07.c mRNA sequence		0.01
12349	TC355837	UP|Q5VRJ8_ORYSA (Q5VRJ8) Peroxisomal biogenesis factor 11 protein-like, 98%	0.01	0.02
12430	TC325252	UP|Q6VBQ7_SORBI (Q6VBQ7) Partner of Nob1	0.01	
12459	TC345686			0.02
12511	TC366355	UP|Q9FZF0_ARATH (Q9FZF0) T2E6.19, 30%		0.01
12644	TC317365	Zea mays clone EL01N0503G10.c mRNA sequence		0.01
12664	TC344478	UP|Q6AVK1_ORYSA (Q6AVK1) Striated muscle activator-like protein		0.01
12732	TC333668	UP|Q75GI8_ORYSA (Q75GI8) Expressed protein, 34%	0.02	
13008	TC352769	UP|Q6NLH7_ARATH (Q6NLH7) At5g19300, 38%	0.01	
13022	TC357346			0.01
13074	TC350009	UP|O82167_ARATH (O82167) Expressed protein (At2g35260/T4C15.7), 22%	0.02	

		13104	TC329826	UP|Q53L24_ORYSA (Q53L24) Expressed protein, 95%		0.01
		13110	TC338107	GB|AAP89016.1|32892213|AY327123 complex interacting protein 9 {Arabidopsis thaliana}, 67%		0.01
		13136	TC339323	UP|Q337L1_ORYSA (Q337L1) Expressed protein, 58%	0.01	
		13269	TC339764	UP|Q75K33_ORYSA (Q75K33) Expressed protein, 59%	0.01	0.02
		13474	TC337463	Zea mays clone EL01N0518E08.c mRNA sequence		0.01
		13630	TC317936	RF|XP_473409.1|50927577|XM_473409 {Oryza sativa (japonica cultivar-group)}, 77%	0.01	
		13849	TC321068	UP|Q33BI2_ORYSA (Q33BI2) Expressed protein, 40%		0.01
		14420	TC327321		0.02	
		14625	TC341110		0.01	
		14633	TC336475	UP|Q9LPN0_ARATH (Q9LPN0) F2J10.3 protein, 32%	0.02	
		14724	TC316335	Zea mays clone EL01N0515E01.d mRNA sequence		-0.01
		14989	TC317792	UP|Q6JN49_MAIZE (Q6JN49) Ethylene receptor	-0.01	
		15085	TC338398		0.01	
		15172	TC344292	UP|Q6CHT8_YARLI (Q6CHT8) Yarrowia lipolytica chromosome A of strain CLIB122 of Yarrowia lipolytica, 17%	0.02	
		15206	TC346709		0.01	
		15283	TC332432	UP|Q415M3_KINRA (Q415M3) N-acyl-D-amino-acid deacylase, 3%		0.01
		15296	TC334876	UP|Q41800_MAIZE (Q41800) Dof2		0.01
		15445	TC366290	UP|Q4ZN44_PSEU2 (Q4ZN44) Protein-disulfide reductase, 5%	0.01	
		15520	TC345051	UP|GOGA5_ARATH (Q8S8N9) Golgin-84, 11%		0.01
		15602	TC317500	UP|Q3S4H5_MAIZE (Q3S4H5) Dual-specificity protein-like phosphatase 3		-0.01
No homology in Arabidopsis		153	TC346145	UP|Q2NNE0_ARATH (Q2NNE0) Calmodulin binding protein IQD22, 25%		-0.01

158	TC320122	RF|XP_507202.1|51964836|XM_507202 P0488B06.44 gene product {Oryza sativa (japonica cultivar-group)}, 91%		-0.01
196	-	-		-0.01
238	TC317940	Zea mays clone EL01N0523H11.d mRNA sequence	-0.01	
256	TC332825	RF|XP_507494.1|51979265|XM_507494 OJ1476_F05.20 gene product {Oryza sativa (japonica cultivar-group)}, 68%		-0.01
260	TC368894			-0.01
268	TC322707	UP|Q84UY9_ORYSA (Q84UY9) BHLH transcription factor PTF1, 60%		0.01
286	TC352842	UP|Q3HTL0_VOLCA (Q3HTL0) Pherophorin-V1 protein precursor, 6%		-0.01
337	TC322278	RF|XP_470380.1|50920039|XM_470380 expressed protein {Oryza sativa (japonica cultivar-group)}, 6%		0.01
372	TC319355	Zea mays clone Contig526 mRNA sequence	-0.01	
379	TC338570	UP|Q9LMR2_ARATH (Q9LMR2) F7H2.6 protein (At1g15720) (MYB transcription factor), 7%		-0.02
413	TC335188	UP|Q7M4Q5_HUMAN (Q7M4Q5) Basic proline-rich peptide IB-8a, 36%		-0.01
429	TC348986			-0.02
551	TC332090	UP|Q6H548_ORYSA (Q6H548) Gamma-tubulin complex component 5-like, 53%		-0.02
557	TC357376			-0.02
580	TC326995	UP|Q6ZHH2_ORYSA (Q6ZHH2) Tyrosine specific protein phosphatase-like, 54%	-0.01	
670	-	-	-0.01	
675	TC332265	UP|Q69JJ6_ORYSA (Q69JJ6) TA1 protein-like, 68%		-0.02
679	TC358985	UP|Q852L4_ORYSA (Q852L4) Expressed protein, 11%	-0.01	
687	TC351138			-0.01
693	TC320507		-0.01	

750	TC334905	UP|Q9FRS7_ARATH (Q9FRS7) F22O13.6 (At1g08580), 54%	-0.01	
771	TC353572	UP|Q3LXA7_MAIZE (Q3LXA7) Retinoblastoma-related 3, 3%		0.01
777	TC320403	Zea mays clone EL01N0314F03.d mRNA sequence	-0.01	
779	TC332316	UP|Q9FNN8_ARATH (Q9FNN8) Cleft lip and palate associated transmembrane protein-like, 17%	-0.01	
802	TC325695	RF|XP_507274.1|51964980|XM_507274 P0481F05.17 gene product {Oryza sativa (japonica cultivar-group)}, 47%		-0.02
819	TC329289	Zea mays clone EL01T0206E06.c mRNA sequence		-0.01
838	TC338433			-0.01
870	TC332464	UP|Q2J869_FRASC (Q2J869) Tetratricopeptide TPR_2, 3%	-0.01	
898	TC326996	GB|AAN12188.1|23094280|AE003595 CG7383-PB, isoform B {Drosophila melanogaster}, 6%		-0.01
957	TC333604	UP|Q5ZB52_ORYSA (Q5ZB52) Zinc knuckle containing protein-like, 9%		-0.01
1031	TC352444	GB|AAB47766.1|1843628|ATU88061 SNF5 homolog BSH {Arabidopsis thaliana}, 11%	-0.01	
1064	TC350178	UP|Q2QM47_ORYSA (Q2QM47) Serine/threonine protein phosphatase, 48%		-0.02
1068	-	-	-0.01	
1081	TC349039	UP|Q6B6R4_ORYSA (Q6B6R4) Transcription factor WRKY07, 6%	-0.01	
1124	TC349293	UP|Q9SBM1_VOLCA (Q9SBM1) Hydroxyproline-rich glycoprotein DZ-HRGP precursor, 6%	-0.01	
1127	TC345204	emb|X80212.2|FHRRNA Funaria hygrometrica 18S rRNA, 5.8S rRNA and 25S rRNA genes and ITS1 and 2, 3%	-0.01	
1152	TC340474		-0.01	
1155	TC317343	Zea mays clone Contig82 mRNA sequence	-0.01	
1175	TC365024	UP|Q69K57_ORYSA (Q69K57) Smr domain-containing protein-like, 5%	-0.01	
1197	TC328630			0.01

1224	TC337299			0.01
1295	TC328186	UP|Q441S7_SOLUS (Q441S7) Pseudouridine synthase, Rsu, 7%	-0.01	-0.01
1313	TC335689		-0.01	
1320	TC335434	UP|Q40853_PICGL (Q40853) Late embryogenesis abundant protein, 44%		-0.01
1337	TC359615	RF|NP_174766.1|15219561|NM_103230 protein binding {Arabidopsis thaliana}, 10%		-0.01
1361	TC328349	UP|Q5N7Y5_ORYSA (Q5N7Y5) Target of myb1-like, 5%		-0.01
1365	TC337298	UP|Q8GST0_ORYSA (Q8GST0) Auxin response factor 1, 53%		-0.01
1443	TC345435			-0.02
1444	-	-	-0.01	
1447	TC350764	UP|SIS_LYMST (P42579) Sodium-influx-stimulating peptide precursor, 10%	-0.01	
1449	TC328576	UP|Q41383_SPIOL (Q41383) Protein kinase, 22%		-0.01
1450	TC330854	UP|Q9FRZ8_ORYSA (Q9FRZ8) E2F homolog, 30%		-0.02
1462	TC329903	UP|Q8GS08_ORYSA (Q8GS08) Disease resistance response protein-like, 56%		-0.01
1474	TC340891	RF|NP_187797.3|30681938|NM_112024 cysteine-type endopeptidase/ double-stranded DNA binding {Arabidopsis thaliana}, 3%		-0.01
1486	TC351830	UP|Q69JW8_ORYSA (Q69JW8) Loricrin-like protein, 7%		-0.01
1515	TC345017	UP|Q6ZJM8_ORYSA (Q6ZJM8) F-box protein family-like, 14%	0.01	
1525	TC340644			-0.01
1620	TC326053	UP|Q69WS1_ORYSA (Q69WS1) Synaptobrevin-like protein (Synaptobrevin 1)	-0.02	
1648	TC328336	UP|Q41787_MAIZE (Q41787) Light harvesting chlorophyll a /b binding protein precursor, 69%	-0.01	
1689	TC342763	UP|Q9PF60_XYLFA (Q9PF60) Endo-1, 4-beta-glucanase, 16%	-0.01	
1695	TC349324	Zea mays clone Contig71 mRNA sequence	-0.01	

1716	TC371250	UP|Q5RZZ4_WHEAT (Q5RZZ4) Meiosis 5, 67%	-0.01	
1720	TC344298	UP|Q6H7E0_ORYSA (Q6H7E0) Steroid membrane binding protein-like, 47%	-0.01	
1802	TC316017	UP|Q93X47_LYCES (Q93X47) Cyclin dependent kinase C, 84%		-0.01
1938	TC315890	UP|PSAB_MAIZE (P04967) Photosystem I P700 chlorophyll a apoprotein A2		0.01
1947	TC327958	Zea mays clone EL01N0561B04.c mRNA sequence		0.01
1950	TC360741	UP|Q6E664_ZEAPE (Q6E664) MPI, 85%	-0.01	
1993	TC356965	UP|Q6K3S8_ORYSA (Q6K3S8) Prefoldin-like, 89%		0.01
2038	TC316378	Zea mays clone Contig713.F mRNA sequence	-0.01	-0.01
2054	TC317810	UP|Q651P3_ORYSA (Q651P3) Peptidoglycan-binding LysM domain-containing protein-like, 88%	-0.02	
2102	TC344047	GB|AAM63860.1|21555444|AY086811 progesterone-binding protein-like {Arabidopsis thaliana}, 67%	-0.01	
2131	TC339500	UP|Q8LPJ1_ARATH (Q8LPJ1) Protein kinase ADK1-like protein (Casein kinase 1-like protein 6), 9%	-0.01	
2163	TC367463	UP|Q4A192_SACOF (Q4A192) Beclin 1 protein, 28%		-0.01
2173	TC353057	UP|Q8VD22_MOUSE (Q8VD22) Ring finger protein 44, 6%	-0.01	
2175	TC327711	UP|Q94AY2_ARATH (Q94AY2) At1g20880/F9H16_14, 40%	-0.01	
2227	TC334647	UP|ROGF1_ARATH (Q93ZY2) Rop guanine nucleotide exchange factor 1, 8%		0.01
2289	TC320965	RF|XP_506750.1|51963934|XM_506750 P0419A09.8 gene product {Oryza sativa (japonica cultivar-group)}, 47%	-0.01	
2309	TC323404	UP|Q9FW70_ORYSA (Q9FW70) Kinesin-like protein, 9%	0.01	0.02
2345	TC342590		-0.01	
2361	TC359020	RF|NP_001031966.1|79329010|NM_001036889 ATP binding {Arabidopsis thaliana}, 38%	-0.01	

2379	TC355308	UP|Q9SD81_ARATH (Q9SD81) Glycerophosphodiester phosphodiesterase-like protein (At5g08030), 8%		0.01
2416	TC330026	UP|Q40452_TOBAC (Q40452) A membrane-associated salt-inducible protein, 58%		-0.02
2426	TC326653	UP|Q67UH7_ORYSA (Q67UH7) Mitochondrial transcription termination factor-like, 42%	-0.01	
2483	TC342696	RF|XP_506722.1|51963878|XM_506722 OJ9003_G05.19 gene product {Oryza sativa (japonica cultivar-group)}, 18%	-0.01	
2505	TC318172	UP|Q414E5_KINRA (Q414E5) Carboxymethylenebutenolidase, 7%		0.01
2545	TC367145	UP|Q6VBJ3_CANGA (Q6VBJ3) Epa4p, 9%	-0.01	
2566	TC331229	RF|XP_506696.1|51963826|XM_506696 P0575F10.6-2 gene product {Oryza sativa (japonica cultivar-group)}, 85%	-0.01	
2574	TC329695	UP|Q7XIW7_ORYSA (Q7XIW7) Myosin heavy chain-like, 52%	-0.01	
2638	TC351034	Zea mays clone EL01N0519H11.c mRNA sequence		0.01
2664	TC353202	UP|Q75GQ2_ORYSA (Q75GQ2) Expressed protein, 21%	0.01	
2702	TC347641	UP|Q67VB8_ORYSA (Q67VB8) CREG2-protein-like, 70%		0.01
2729	TC358871	UP|O23864_9ORYZ (O23864) Polyprotein, 6%		0.01
2735	TC333816			0.02
2739	TC354714	UP|Q8SA97_MAIZE (Q8SA97) Ornithine carbamoyltransferase, 4%		0.01
2773	TC349959			0.01
2777	TC322758	UP|Q3E9H4_ARATH (Q3E9H4) Protein At5g15550, 28%	-0.01	
2787	TC344397	UP|Q6PKC9_HUMAN (Q6PKC9) SFRS11 protein, 6%		0.01
2811	TC349802		-0.01	-0.01
2880	TC316727	UP|Q53NM9_ORYSA (Q53NM9) DnaK-type molecular chaperone hsp70-rice		0.01

2890	TC328408	RF|XP_506138.1|51963242|XM_506138 P0585H11.111 gene product {Oryza sativa (japonica cultivar-group)}, 35%	0.01	
2891	TC326562	UP|Q9VN28_DROME (Q9VN28) CG14650-PA, 3%	-0.01	
2914	TC321900	GB|AAA19069.1|500730|U10402 C. elegans neuro d homolog protein 1 {Caenorhabditis elegans}, 8%	-0.01	
2915	TC324922	UP|Q5MFV3_ORYSA (Q5MFV3) BTH-induced ERF transcriptional factor 1, 5%		-0.01
3035	TC335481	UP|Q30KI3_9POAL (Q30KI3) RLK1, 13%		0.01
3042	TC347535	UP|Q3CML1_ALTAT (Q3CML1) Sugar transporter precursor, 4%	-0.01	
3063	TC333281	GB|AAT85270.1|50881425|AC133335 expressed protein {Oryza sativa (japonica cultivar-group)}, 96%	0.01	-0.01
3073	TC332066	UP|Q69XV5_ORYSA (Q69XV5) DNA-binding protein-like, 38%	0.02	
3092	TC318654	UP|Q9LL87_MAIZE (Q9LL87) Beta-glucosidase aggregating factor, 13%	-0.01	
3096	TC348210	UP|Q9MAN1_ARATH (Q9MAN1) T25K16.3, 29%	-0.01	
3101	TC327563	emb|Z00028.1|CHZMRRNA Zea mays chloroplast rRNA-operon, 12%		0.01
3124	TC346624	UP|MIOX_ORYSA (Q5Z8T3) Probable inositol oxygenase, 12%	-0.01	
3129	TC343004	RF|XP_507561.1|51979539|XM_507561 P0470F10.18 gene product {Oryza sativa (japonica cultivar-group)}		0.01
3131	TC352799	UP|Q2IMJ3_ANADE (Q2IMJ3) LigA, 4%		0.01
3146	TC333587	Zea mays clone EL01N0413D09.c mRNA sequence	-0.01	
3148	TC341695	UP|Q69TF7_ORYSA (Q69TF7) Subtilase-like, 6%	-0.01	0.01
3159	TC322856	Zea mays clone EL01N0551H02.c mRNA sequence	-0.02	
3171	TC335136	UP|PFD2_ARATH (Q9LJ98) Probable prefoldin subunit 2, 49%		0.01
3175	TC336325	UP|Q5N7K2_ORYSA (Q5N7K2) Zinc finger protein-like, 9%		0.01

3191	TC345491	UP|Q8GHY4_PSERE (Q8GHY4) Partitioning protein, 7%		0.01
3192	TC339282	RF|NP_178116.2|30699476|NM_106648 protein transporter {Arabidopsis thaliana}, 3%	-0.01	
3195	TC330406	UP|Q6F3A5_ORYSA (Q6F3A5) Expressed protein, 22%	-0.01	0.01
3199	TC358379	RF|XP_506705.1|51963844|XM_506705 P0576F08.15 gene product {Oryza sativa (japonica cultivar-group)}, 5%	-0.01	0.01
3290	TC337009	UP|Q6ZL26_ORYSA (Q6ZL26) Mitogen activated protein kinase kinase, 24%	-0.01	
3296	TC333060	UP|Q2R2Z3_ORYSA (Q2R2Z3) Expressed protein, 32%		-0.01
3300	TC349665	UP|Q3H3A5_9ACTO (Q3H3A5) Uncharacterised conserved protein UCP005026, 3%	-0.01	
3404	TC344447	RF|NP_496366.1|17533635|NM_063965 C.Elegans Y-box family member (cey-1) {Caenorhabditis elegans}, 16%	-0.01	
3437	TC370236	GB|AAK60291.1|14326491|AF385698 At2g43780/F18O19.11 {Arabidopsis thaliana}, 70%		0.01
3445	TC347011			0.01
3475	TC357151	UP|Q8L685_VOLCA (Q8L685) Pherophorin-dz1 protein precursor, 6%	-0.01	
3476	TC336471		-0.01	
3481	TC343186	UP|Q9FQX3_APIGR (Q9FQX3) Mannitol transporter, 30%		-0.02
3542	TC335300	GB|AAP68253.1|31711794|BT008814 At3g07950 {Arabidopsis thaliana}, 19%		-0.01
3561	TC317661	UP|Q94BW4_CINCA (Q94BW4) Type 2 ribosome-inactivating protein cinnamomin II precursor, 19%		0.01
3563	TC348586	GB|AAA83618.1|1125842|U43375 Sulfatase domain protein protein 1 {Caenorhabditis elegans} , 3%		0.01
3566	TC368853			0.01
3597	TC357768	UP|MDCG_PSESM (Q87V59) Phosphoribosyl-dephospho-CoA transferase (Holo-ACP synthase) , 10%		0.01
3638	TC320314	UP|O22812_ARATH (O22812) AT-hook DNA-binding protein, 45%	-0.01	

3735	TC317111	Zea mays clone EL01N0519D03.d mRNA sequence		0.01
3790	TC330854	UP|Q9FRZ8_ORYSA (Q9FRZ8) E2F homolog, 30%		0.01
3885	TC350995		0.02	
3907	TC323005	RF|NP_563787.2|42561780|NM_100609 RNA binding {Arabidopsis thaliana}, 40%	0.01	
3969	TC363771		-0.01	
3970	TC340803		-0.01	
3971	TC359383	UP|Q8L5P7_PHAVU (Q8L5P7) LHY protein, 7%		0.02
3976	TC352574			0.01
4012	TC354601	Zea mays clone EL01N0421G10.c mRNA sequence	-0.01	
4027	TC344202	Z.mays (MU141D) mRNA for U14 small nucleolar RNA	-0.01	
4207	TC340985	UP|Q42412_NICSY (Q42412) RNA-binding protein RZ-1, 69%		-0.01
4221	TC350504	RF|NP_198440.1|15239221|NM_122982 3'-5' exonuclease/ nucleic acid binding {Arabidopsis thaliana}, 8%	0.01	
4225	TC329760	UP|Q94L33_ARATH (Q94L33) Ania-6a type cyclin, 9%		0.01
4244	TC344774	UP|Q40W17_KINRA (Q40W17) Prolyl-tRNA synthetase, bacterial, 3%		0.01
4271	TC329852	GB|AAQ23057.1|33591100|AY344485 heat shock factor RHSF3 {Oryza sativa (japonica cultivar-group)}, 84%		0.01
4273	TC336183	UP|Q9MAL9_ARATH (Q9MAL9) T25K16.15 (At1g01160/F6F3_1) (GRF1-interacting factor 2), 37%	0.02	
4282	TC368407	UP|Q85F99_9NEOP (Q85F99) Cytochrome oxidase subunit I, 6%	-0.01	
4285	TC346017	UP|Q86C60_LOLOP (Q86C60) Protamine, 32%	-0.01	0.01
4313	TC319654	UP|Q53M44_ORYSA (Q53M44) At2g43970/F6E13.10, 66%	-0.01	
4314	TC354745		-0.01	

4325	TC353450			0.01
4358	TC342192			0.01
4360	TC359374	GB|BAD82705.1|56785066|AP004331 OsNAC4 protein {Oryza sativa (japonica cultivar-group)}, 57%		0.01
4365	TC328704	UP|Q9AV77_ORYSA (Q9AV77) 60S ribosomal protein L17, 62%		0.01
4385	TC363091			0.02
4419	TC322815	UP|MAD32_ORYSA (Q8S151) MADS-box transcription factor 32 (OsMADS32), 88%	-0.02	
4436	TC321328	UP|Q2F7H7_MAIZE (Q2F7H7) Coproporphyrinogen III oxidase, 61%	-0.01	
4439	TC347469	UP|Q9ATM9_MAIZE (Q9ATM9) Plasma membrane integral protein ZmPIP2-1, 32%	-0.01	
4473	TC348425	GB|AAT77840.1|50540683|AC147426 expressed protein {Oryza sativa (japonica cultivar-group)}, 89%	-0.01	0.01
4486	TC319627	UP|EXG2_SCHPO (Q10444) Glucan 1, 3-beta-glucosidase 2 precursor, 3%		0.01
4495	TC359777	UP|Q40YQ9_KINRA (Q40YQ9) GTP cyclohydrolase I precursor, 4%	-0.02	-0.01
4570	TC329437	Zea mays clone Contig96 mRNA sequence		0.01
4576	TC322614	UP|Q3LVM2_TAROF (Q3LVM2) TO49-1rc, 29%	-0.01	
4646	TC368305	UP|U2AF1_DROME (Q94535) Splicing factor U2af 38 kDa subunit, 7%	-0.01	
4657	TC368620		-0.01	
4661	TC320442	UP|Q3GSZ0_9ACTO (Q3GSZ0) Response regulator receiver:ATP-binding region, ATPase-like:Histidine kinase A, N-terminal, 3%	0.01	
4670	TC325950	UP|Q7X9C0_LOTJA (Q7X9C0) NIN-like protein 2, 32%	-0.01	
4679	TC359029	RF|NP_910326.1|34897960|NM_185437 serine/threonine-protein kinase Mak (male germ cell-associated kinase)-like {Oryza sativa (japonica cultivar-group)}, 36%		0.01
4694	TC329329	RF|NP_914823.1|34906952|NM_189934 DNA-binding protein-like protein {Oryza sativa (japonica cultivar-group)}, 14%		0.01

4701	TC346518	UP|Q651C2_ORYSA (Q651C2) Aspartic proteinase nepenthesin II-like, 9%		0.01
4724	TC362689	UP|Q5TUQ4_ANOGA (Q5TUQ4) ENSANGP00000028695, 12%		0.01
4738	TC342494	UP|Q84XF7_MALXI (Q84XF7) Integral membrane protein Nramp1, 12%		0.01
4771	TC334657	UP|Q6ENK8_ORYSA (Q6ENK8) Splicing factor 4-like protein, 48%		0.01
4776	TC340131	UP|Q6K9T9_ORYSA (Q6K9T9) Metallo-beta-lactamase-like, 66%		0.01
4779	TC359998			0.01
4807	TC366047	UP|Q5U7K6_9POAL (Q5U7K6) Metallothionein-like protein, 93%	-0.01	
4833	TC349655		0.01	
4842	TC358772	UP|SEM11_ARATH (Q9XIR8) Probable 26 proteasome complex subunit sem1-1, 92%	-0.01	
4849	TC316136	Zea mays clone EL01N0372H11.d mRNA sequence		0.01
4859	-	-		0.01
4860	TC347826	UP|Q9M8Z1_ARATH (Q9M8Z1) T6K12.3 protein (AT3g04350/T6K12_3), 28%	-0.01	
4869	TC316178	Zea mays clone Contig461 mRNA sequence		-0.01
4873	TC349665	UP|Q3H3A5_9ACTO (Q3H3A5) Uncharacterised conserved protein UCP005026, 3%		0.01
4920	TC331730	UP|GIANT_DROME (P39572) Protein giant, 4%		0.01
4984	TC340040	Zea mays clone Contig84 mRNA sequence		0.01
5039	TC316466	Zea mays clone EL01N0372C04.c mRNA sequence		0.01
5042	-	-	0.01	0.01
5121	TC330354	UP|Q4STU6_TETNG (Q4STU6) Chromosome undetermined SCAF14113, whole genome shotgun sequence, 10%		0.01
5126	TC352487	UP|Q6ZLD8_ORYSA (Q6ZLD8) Fiber protein-like, 40%		0.02
5168	TC337016	UP|Q3GT62_9ACTO (Q3GT62) Phosphoglycerate/bisphosphoglycerate mutase:RNase H, 4%	-0.01	

5172	TC361527			0.01
5189	TC358283	UP|Q293G4_DROPS (Q293G4) GA14932-PA, 11%		0.01
5200	TC332886			0.01
5221	TC340985	UP|Q42412_NICSY (Q42412) RNA-binding protein RZ-1, 69%		-0.01
5295	TC352854	UP|H1_MAIZE (P23444) Histone H1, 89%	-0.01	
5364	TC335058			0.01
5429	TC348388	UP|Q294I1_DROPS (Q294I1) GA11316-PA, 3%	0.01	
5448	TC357323	UP|Q9GZF7_CAEEL (Q9GZF7) Collagen protein 102, 4%		-0.01
5472	TC330232	UP|Q6NKV4_ARATH (Q6NKV4) At5g59410, 81%		0.01
5481	TC344608	UP|Q84YJ6_ORYSA (Q84YJ6) DNA-binding protein family-like, 13%		0.01
5527	TC345643	UP|Q2IMJ3_ANADE (Q2IMJ3) LigA, 5%		0.01
5585	TC340240	UP|Q7XBA5_ORYSA (Q7XBA5) Drought-induced protein DI1, 36%		0.01
5613	TC318719	RF|NP_172201.1|15222340|NM_100595 structural constituent of ribosome {Arabidopsis thaliana}, 27%	0.01	
5648	-	-		0.01
5693	TC360799	UP|MAD32_ORYSA (Q8S151) MADS-box transcription factor 32 (OsMADS32), 42%	-0.01	-0.01
5717	TC325899	GB|AAB23484.1|256638|S45168 15 kda organ-specific salt-induced protein Method: conceptual translation with partial peptide sequencing {Oryza sativa}, 10%		0.01
5840	TC350331	GB|AAH13549.1|15488836|BC013549 trinucleotide repeat containing 5 {Mus musculus}, 5%		0.01
5844	TC351115			0.01
5855	TC325355	RF|NP_198865.1|15242719|NM_123413 RNA binding {Arabidopsis thaliana}, 47%		0.01
5866	-	-	-0.01	

5870	-	-		0.01
5877	TC334223	UP|Q2J8A0_FRASC (Q2J8A0) Dehydrogenase subunit, 6%		0.01
5883	TC327706	UP|Q9FIR6_ARATH (Q9FIR6) Gb|AAD25674.1, 8%		0.01
5904	TC368330	UP|Q6EPQ3_ORYSA (Q6EPQ3) SPX (SYG1/Pho81/XPR1) domain-containing protein-like, 25%		0.01
5910	TC352336	UP|Q2RGK2_MOOTA (Q2RGK2) Diguanylate cyclase (GGDEF domain) with PAS/PAC sensor, 5%		0.01
5929	TC357545			0.01
5930	TC360536			0.02
5933	TC363275			0.01
5939	TC347373			0.01
5970	TC365235			0.01
5973	TC355990	UP|Q8LPK4_ARATH (Q8LPK4) Alpha-adaptin C-like protein, 10%		0.01
5980	TC345640			0.01
6024	TC337851	UP|Q8IP68_DROME (Q8IP68) CG31813-PA, 8%	-0.01	
6050	TC347648	UP|Q9XFG7_WHEAT (Q9XFG7) Isoamylase 1, 5%	-0.01	0.01
6057	TC316846	UP|Q4SP86_TETNG (Q4SP86) Chromosome 15 SCAF14542, whole genome shotgun sequence, 5%		0.01
6068	TC325310	UP|IMA1A_ORYSA (Q71VM4) Importin alpha-1a subunit, 44%		0.01
6097	TC332416	UP|IMA1B_ORYSA (Q9SLX0) Importin alpha-1b subunit, 31%		0.02
6110	TC357861	UP|Q6DDA7_XENTR (Q6DDA7) MGC89913 protein, 6%	-0.01	
6130	TC345654			0.01
6133	-	-		0.01
6135	TC329074	UP|Q96232_ASPOF (Q96232) Proline-rich-like protein, 54%		0.01

6149	TC348376	UP|Q655X0_ORYSA (Q655X0) Thioredoxin-like, 61%		0.01
6153	TC354043	UP|Q4SV43_TETNG (Q4SV43) Chromosome undetermined SCAF13807, whole genome shotgun sequence., 7%		0.02
6158	TC332699	UP|Q7Q1D5_ANOGA (Q7Q1D5) ENSANGP00000015795, 15%		-0.01
6163	TC340040	Zea mays clone Contig84 mRNA sequence		0.01
6164	TC341226	Zea mays clone cho1c.pk003.k1, mRNA sequence		0.01
6203	TC367414			0.01
6226	TC358253			0.01
6232	TC366919			0.01
6245	TC350323	RF|NP_199086.2|22327528|NM_123636 hydrolase, hydrolyzing O-glycosyl compounds {Arabidopsis thaliana}, 23%		0.02
6278	TC358078			0.01
6293	TC339102			0.01
6294	TC338081			0.01
6312	TC340854			0.01
6314	TC331624	UP|Q9M9F8_ARATH (Q9M9F8) F3F9.7, 8%		0.01
6330	TC345001	UP|Q4KXD9_LOPEL (Q4KXD9) Early salt stress and cold acclimation-induced protein 2-1, 39%		0.01
6335	TC357421			0.01
6336	TC333248	UP|Q75GI7_ORYSA (Q75GI7) Expressed protein, 72%		-0.02
6358	TC316726	Zea mays clone Contig130 mRNA sequence	-0.01	
6359	TC323761	RF|NP_496363.1|17533631|NM_063962 LEThal family member (let-858) {Caenorhabditis elegans}, 3%	-0.01	
6369	TC362576			0.01

6374	TC350639			0.01
6376	TC324720			0.02
6378	TC347823			0.01
6385	TC338887		-0.02	
6430	TC321193	Zea mays clone EL01N0450E05.d mRNA sequence		0.01
6456	TC345066	UP|Q653F2_ORYSA (Q653F2) RNA polymerase I specific transcription initiation factor RRN3-like, 87%		0.01
6634	TC333502	UP|Q2RBF5_ORYSA (Q2RBF5) Expressed protein, 15%		-0.01
6644	TC368618	RF|NP_179899.1|15227788|NM_127882 CYP96A1 heme binding {Arabidopsis thaliana}, 5%	0.01	
6670	TC325879	UP|CSN7_ARATH (Q94JU3) COP9 signalosome complex subunit 7, 79%		-0.01
6672	TC345894			0.01
6679	TC365330	UP|Q9LMH4_ARATH (Q9LMH4) F16A14.4, 12%	0.01	
6688	TC352800	UP|Q4S436_TETNG (Q4S436) Chromosome 20 SCAF14744, whole genome shotgun sequence, 5%		-0.01
6725	TC366040	RF|XP_507334.1|51965100|XM_507334 P0562A06.11 gene product {Oryza sativa (japonica cultivar-group)}, 73%	0.02	
6732	TC356515	UP|Q5ZBG2_ORYSA (Q5ZBG2) Polypyrimidine tract-binding protein 1-like, 47%	0.01	0.01
6776	TC323929	RF|NP_194194.1|15233900|NM_118596 metal ion binding {Arabidopsis thaliana}, 62%		0.02
6830	TC318428	Zea mays clone EL01N0360D09.c mRNA sequence	-0.01	-0.01
6895	TC347294		-0.01	-0.01
6923	TC318428	Zea mays clone EL01N0360D09.c mRNA sequence		-0.02
7002	TC331697			0.01
7004	TC359185	UP|HD2B_MAIZE (Q9M4U5) Histone deacetylase 2b, 5%		0.01

7012	TC340012	UP|Q45W71_ARAHY (Q45W71) Auxin-repressed protein, 79%		-0.01
7028	TC334811	Zea mays clone EL01N0361C03.c mRNA sequence		0.01
7046	TC327016	RF|NP_188147.1|15232545|NM_112392 3-hydroxybutyryl-CoA dehydrogenase/ oxidoreductase {Arabidopsis thaliana}, 90%		0.01
7047	TC328551	UP|O80644_ARATH (O80644) Expressed protein, 67%	-0.01	
7070	TC349644	UP|Q80LM0_NPVAH (Q80LM0) Basic DNA-binding protein P6.9, 25%		0.01
7122	TC336222			0.02
7166	TC341185	UP|Q2R0W8_ORYSA (Q2R0W8) Expressed protein, 63%		0.01
7176	TC316550	Zea mays clone EL01N0365C01.c mRNA sequence	0.01	
7177	TC328541	UP|Q5BM98_9ROSI (Q5BM98) Secondary cell wall-related glycosyltransferase family 47, 23%		0.01
7286	TC336094	UP|Q2JFS0_FRASC (Q2JFS0) Porphobilinogen deaminase, 5%	-0.01	
7295	TC358218	UP|Q9M640_MAIZE (Q9M640) Delta-COP, 24%		0.01
7371	TC354827	UP|Q9FNN8_ARATH (Q9FNN8) Cleft lip and palate associated transmembrane protein-like, 38%	-0.01	
7385	TC357882	UP|Q5U7K6_9POAL (Q5U7K6) Metallothionein-like protein, 98%		0.01
7414	TC320322	UP|TKTC_MAIZE (Q7SIC9) Transketolase, chloroplast (TK) , 60%		0.01
7425	TC363889	UP|Q9SSS7_ARATH (Q9SSS7) F6D8.2 protein (At1g52730), 95%		0.01
7426	TC330445	RF|XP_506148.1|51963248|XM_506148 P0474G09.102 gene product {Oryza sativa (japonica cultivar-group)}, 16%		0.01
7441	TC344041	UP|Q6YWQ2_ORYSA (Q6YWQ2) Acidic 82 kDa protein-like, 10%		0.02
7445	-	-		0.01
7461	TC354922	UP|Q3IQZ7_NATPD (Q3IQZ7) Transport ATPase, component for probable LAO/AO transport systems, 6%		0.01
7470	-	-		0.01

7473	TC317766	UP|CEBPB_MOUSE (P28033) CCAAT/enhancer-binding protein beta , 5%		0.01
7482	-	-		0.01
7495	TC328911	GB|AAA28381.1|156976|DROBARH1A2 {Drosophila ananassae}, 3%		0.01
7510	TC340710	UP|Q53AN3_ORYSA (Q53AN3) Gibberellin-stimulated protein, 75%	0.01	
7522	TC330192	Zea mays clone Contig459 mRNA sequence		0.01
7528	TC346984		0.01	
7544	TC353135		-0.01	
7567	TC330120	UP|Q4C3T1_CROWT (Q4C3T1) ABC-1, 6%	-0.01	
7617	TC329306		-0.01	
7618	TC329085	RF|XP_506513.1|51963608|XM_506513 P0503D09.102 gene product {Oryza sativa (japonica cultivar-group)}, 12%	-0.01	
7636	TC323281	UP|Q942G1_ORYSA (Q942G1) Alliin lyase-like, 49%	-0.01	
7868	TC368305	UP|U2AF1_DROME (Q94535) Splicing factor U2af 38 kDa subunit, 7%	0.01	
7881	-	-		0.01
7883	TC329581	RF|NP_566630.1|18402188|NM_112817 kinase {Arabidopsis thaliana}, 31%	0.01	
7889	TC360282	UP|DRTS_MAIZE (O81395) Bifunctional dihydrofolate reductase-thymidylate synthase [Includes: Dihydrofolate reductase Thymidylate synthase] , 7%	0.01	
7933	TC360719	UP|Q6A4T5_DROME (Q6A4T5) SGG, 8%		0.01
7957	TC349163	UP|Q6YS82_ORYSA (Q6YS82) RRM-containing RNA-binding protein-like, 23%		0.01
7973	TC365119		-0.01	0.01
7977	TC360547	UP|Q5NKH8_ORYSA (Q5NKH8) MYB20 protein, 8%		0.01
8017	TC327342		-0.01	

8023	-	-	0.02	
8030	TC369901		0.01	
8095	TC317715	Zea mays clone cho1c.pk003.i13, mRNA sequence	0.01	
8113	TC341545	UP|Q6Z5K5_ORYSA (Q6Z5K5) Sucrase-like protein, 32%	0.01	
8119	TC348873	UP|Q9ZUX1_ARATH (Q9ZUX1) Cytochrome P450 CYP94C1 (At2g27690/F15K20.21), 71%		0.01
8138	-	-		0.01
8150	-	-		0.01
8244	TC355604	RF|NP_568713.1|18423070|NM_124360 EMB1879 {Arabidopsis thaliana}, 74%		0.02
8253	TC345662	UP|Q5SN38_ORYSA (Q5SN38) TA9 protein-like, 25%	-0.01	
8258	TC317114	UP|Q941Y7_ORYSA (Q941Y7) RING finger-like protein, 75%		-0.01
8277	TC350880	UP|CIRB_CHAPA (P56879) Circulin-B (CIRB), 90%	0.01	
8301	TC331967	UP|OXA1L_ARATH (Q9SKD3) Inner membrane protein OXA1-like, mitochondrial precursor, 8%	0.01	0.01
8327	TC349593	UP|Q9SKW6_ARATH (Q9SKW6) F5J5.4, 5%	0.01	
8329	TC327880	UP|DCAM_MAIZE (O24575) S-adenosylmethionine decarboxylase proenzyme , 38%		0.01
8374	TC362839			0.01
8378	TC323783	UP|Q653N3_ORYSA (Q653N3) Myosin II heavy chain-like, 18%		0.01
8380	TC319665	UP|Q40YD6_KINRA (Q40YD6) UspA, 9%	-0.01	
8407	TC367145	UP|Q6VBJ3_CANGA (Q6VBJ3) Epa4p, 9%		-0.01
8411	TC316958	Zea mays clone Contig969.F mRNA sequence	-0.01	-0.02
8413	TC318654	UP|Q9LL87_MAIZE (Q9LL87) Beta-glucosidase aggregating factor, 13%		-0.01
8440	TC338534	UP|Q5QNL2_ORYSA (Q5QNL2) DNA polymerase delta p66 subunit-like, 26%	-0.01	

8461	TC341015		0.01	
8472	-	-		0.01
8496	TC367799	Zea mays clone Contig87 mRNA sequence	0.01	
8509	TC367985	UP|Q2QMW0_ORYSA (Q2QMW0) Expressed protein, 93%		0.01
8539	TC333114	Zea mays clone EL01N0526H03.c mRNA sequence	-0.01	
8621	TC334948	UP|Q7F1H3_ORYSA (Q7F1H3) Blue copper-binding protein-like, 75%		-0.01
8622	TC331925	GB|AAP68268.1|31711824|BT008829 At5g47680 {Arabidopsis thaliana}, 14%		0.01
8625	TC336112	RF|NP_566035.1|18406715|NM_130078 phosphatidate cytidylyltransferase {Arabidopsis thaliana}, 9%		0.01
8640	TC334809	RF|XP_481420.1|50943785|XM_481420 chloroplast RNA processing protein-like {Oryza sativa (japonica cultivar-group)}, 7%		0.01
8656	-	-	-0.01	
8677	TC327971	UP|Q8RUF8_ARATH (Q8RUF8) AT5g12040/F14F18_210, 41%		-0.01
8728	TC353100	UP|Q2UIQ9_ASPOR (Q2UIQ9) Predicted protein, 11%	0.01	
8752	TC365366		-0.01	
8762	TC334154	UP|IF4A3_NICPL (P41380) Eukaryotic initiation factor 4A-3 , 47%	-0.01	
8772	TC362985	UP|MNB1A_MAIZE (P38564) Dof zinc finger protein MNB1A, 39%		0.01
8774	TC358552			0.01
8788	TC355604	RF|NP_568713.1|18423070|NM_124360 EMB1879 {Arabidopsis thaliana}, 74%	0.01	
8874	TC326740	UP|Q4ZH87_ORYSA (Q4ZH87) DsRNA binding protein RBP, 52%		0.02
8887	TC365017	UP|Q651Q0_ORYSA (Q651Q0) Bzip-related transcription factor-like, 4%	-0.01	
8976	TC316393	UP|Q6H759_ORYSA (Q6H759) Copper chaperone homolog CCH, 78%	0.01	

8986	TC364361			-0.01
9027	TC344200	UP|Q9LMV5_ARATH (Q9LMV5) F5M15.20, 8%		-0.02
9075	TC322306	Zea mays clone EL01N0324A06.c mRNA sequence		0.01
9076	-	-	0.01	0.01
9077	-	-		0.01
9083	TC333143	UP|Q6SSD6_WHEAT (Q6SSD6) Pollen-specific protein, 36%		0.01
9136	TC318470	UP|Q6YZ10_ORYSA (Q6YZ10) 27k vesicle-associated membrane protein-associated protein-like, 86%		0.01
9156	-	-		0.01
9173	TC353924	UP|Q69IU6_ORYSA (Q69IU6) Ankyrin-like protein, 18%		0.01
9261	TC352034			0.01
9268	TC340040	Zea mays clone Contig84 mRNA sequence		0.01
9271	TC326772	UP|Q2KP14_ARAHY (Q2KP14) Soluble diacylglycerol acyltransferase, 15%	-0.01	0.01
9332	TC329244	UP|Q2QW42_ORYSA (Q2QW42) Expressed protein, 92%		0.01
9340	TC325282	UP|Q94L33_ARATH (Q94L33) Ania-6a type cyclin, 15%		-0.01
9348	TC320180	UP|Q6NMZ4_ARATH (Q6NMZ4) At4g34412, 76%		0.01
9360	TC358764			0.01
9361	TC336376	UP|Q3E9B0_ARATH (Q3E9B0) Protein At5g19950, 25%	0.02	
9365	TC316321	Zea mays clone Contig631.F mRNA sequence		0.01
9389	TC347844	UP|Q7XAM4_ORYSA (Q7XAM4) Chromodomain-helicase-DNA-binding protein-like protein, 89%		0.01
9456	-	-		0.01
9482	TC347013	UP|Q6Z750_ORYSA (Q6Z750) Ankyrin-like protein, 18%		0.01

9491	TC356820	RF|NP_173279.2|30685853|NM_101701 aminoacyl-tRNA hydrolase {Arabidopsis thaliana}, 5%	0.01	0.01
9573	TC333300	UP|SOX_ARATH (Q9SJA7) Probable sarcosine oxidase, 13%		-0.01
9576	TC331255	UP|RF2B_ORYSA (Q6S4P4) Transcription factor RF2b, 66%	-0.01	
9616	TC371086	RF|NP_187361.1|15231373|NM_111585 GTP binding {Arabidopsis thaliana}, 33%		0.01
9659	TC340991	UP|Q9LM45_ARATH (Q9LM45) F2E2.21, 45%	-0.01	
9663	TC362853		-0.01	
9695	TC339225		0.01	
9698	TC318293	GB|AAA33166.1|167666|DDIANVIIA annexin VII {Dictyostelium discoideum}, 10%		0.01
9739	TC367362	UP|Q74Z85_ASHGO (Q74Z85) AGR318Cp, 5%	-0.02	
9754	TC335188	UP|Q7M4Q5_HUMAN (Q7M4Q5) Basic proline-rich peptide IB-8a, 36%		-0.01
9826	TC355955	UP|PR4_PHAVU (Q09020) Wound-induced basic protein, 40%		-0.01
9827	TC358883	UP|Q8LK06_MAIZE (Q8LK06) Methyl binding domain protein MBD109, 83%		0.01
9841	TC353940	UP|Q5N7Q9_ORYSA (Q5N7Q9) Phosphatidic acid phosphatase beta-like, 82%	0.01	
9847	TC349766		0.01	
9863	TC364761	UP|Q2IMJ3_ANADE (Q2IMJ3) LigA, 6%	0.01	0.01
9865	TC359810	UP|Q338G6_ORYSA (Q338G6) BT004686 At1g26948, 73%	-0.01	
9885	TC331270			-0.01
9888	TC347513	UP|Q4S810_TETNG (Q4S810) Chromosome 9 SCAF14710, whole genome shotgun sequence., 5%	0.01	0.01
9911	TC352340	UP|O24343_SORBI (O24343) Serine/threonine kinase, 37%		-0.02
9963	TC366187			-0.01
9971	TC346806	UP|Q9B0N5_9GOBI (Q9B0N5) NADH dehydrogenase subunit 2, 15%	0.01	

9972	TC338383	UP|PSCBP_RAT (Q5I0L6) Pleckstrin homology Sec7 and coiled-coil domain-binding protein, 5%		-0.02
10018	TC335804	Zea mays clone Contig94.F mRNA sequence	-0.01	
10027	TC333060	UP|Q2R2Z3_ORYSA (Q2R2Z3) Expressed protein, 32%	-0.01	-0.01
10049	TC349896		-0.01	-0.01
10119	TC328349	UP|Q5N7Y5_ORYSA (Q5N7Y5) Target of myb1-like, 5%	-0.01	
10151	TC340673	UP|Q29AF1_DROPS (Q29AF1) GA11058-PA, 6%	-0.02	-0.01
10153	TC315966	UP|CF23_DROME (Q01522) Chorion transcription factor Cf2, isoform III, 4%	-0.01	
10161	TC356462		-0.01	
10162	TC360680	UP|Q8X3T8_ECO57 (Q8X3T8) No significant matches, 9%	-0.01	
10195	TC319694	UP|Q6ID77_ARATH (Q6ID77) At3g01435, 43%	0.01	
10223	TC318194	Zea mays clone EL01N0424C02.c mRNA sequence		-0.01
10238	TC360965	RF|XP_507164.1|51964760|XM_507164 OJ1734_E04.12 gene product {Oryza sativa (japonica cultivar-group)}, 82%		0.01
10258	TC330626	UP|Q5U7K6_9POAL (Q5U7K6) Metallothionein-like protein, 94%		-0.01
10279	TC328183	Zea mays clone EL01N0526G04.d mRNA sequence	0.01	
10280	TC353921	UP|CS029_HUMAN (Q8WUQ7) Protein C19orf29 (NY-REN-24 antigen), 4%		0.01
10282	TC339405		0.02	
10296	TC347256	UP|Q40553_TOBAC (Q40553) N.tabacum mRNA pNLA-28, 53%		0.01
10337	TC332885	UP|Q57182_BORBU (Q57182) Flagellar export apparatus (Flagellar export protein), 9%	0.01	
10377	TC358464			0.02
10378	TC360498			0.01
10400	TC323447	GB|AAF79509.1|8778501|AC002328 F20N2.5 {Arabidopsis thaliana}, 5%	0.01	0.01

10417	TC327958	Zea mays clone EL01N0561B04.c mRNA sequence	-0.01	-0.01
10451	TC358709	UP|NU3C_MAIZE (P19044) NAD(P)H-quinone oxidoreductase chain 3, chloroplast		0.02
10467	TC343594	RF|XP_507607.1|51979721|XM_507607 P0562A06.14 gene product {Oryza sativa (japonica cultivar-group)}		0.01
10518	TC348284	UP|Q9XHE5_ARATH (Q9XHE5) Microtubule-associated protein, 5%		0.01
10605	TC320381	UP|Q66PX4_SACOF (Q66PX4) Mitochondrial uncoupling protein 4, 61%		0.01
10672	TC350638	UP|Q564C9_WHEAT (Q564C9) Jasmonate-induced protein, 10%	0.01	
10678	-	-		0.01
10691	TC340018	UP|Q6IR48_BRARE (Q6IR48) Zgc:63914, 6%		0.01
10699	TC358268		-0.01	
10732	TC341247	UP|Q9ASQ1_ARATH (Q9ASQ1) At1g04290/F19P19_27, 57%		0.01
10761	TC347222	UP|Q75HY5_ORYSA (Q75HY5) Unknow protein, 19%		0.01
10767	TC349562	UP|Q40543_TOBAC (Q40543) Protein-serine/threonine kinase, 49%	-0.02	
10778	TC356846	PRF|1211356A|225315|1211356A zein gamma. {Zea mays} , 28%		0.01
10785	TC329176	UP|RS3_AMBME (P79891) 40S ribosomal protein S3, 8%		0.01
10811	TC324455	UP|Q7XSU6_ORYSA (Q7XSU6) Peroxidase, 44%	-0.01	
10812	TC340673	UP|Q29AF1_DROPS (Q29AF1) GA11058-PA, 6%		-0.01
10829	TC324490			-0.01
10832	-	-		-0.01
10915	TC321559	UP|Q6VBJ3_CANGA (Q6VBJ3) Epa4p, 4%		-0.02
10937	TC338881			0.01
10982	TC334528			-0.02

11071	-	-		0.01
11086	TC335575			-0.01
11118	TC359171	Zea mays clone Contig535 mRNA sequence		0.01
11129	TC333733	UP|Q3AZU3_SYNS9 (Q3AZU3) ATPase, 3%		0.01
11132	TC368847			0.01
11139	TC341271	UP|Q7XTF6_ORYSA (Q7XTF6) OJ991214_12.11 protein, 80%		0.01
11155	TC360713	UP|Q761Y0_ORYSA (Q761Y0) BRI1-KD interacting protein 135, 8%		-0.01
11162	TC340095	UP|PALI_MAGGR (Q51MB1) pH-response regulator protein palI/RIM9, 5%		0.01
11174	TC316398	Zea mays clone EL01N0323A09.c mRNA sequence	0.02	0.01
11178	TC363069			0.01
11179	TC359235			0.01
11220	TC327557	Zea mays clone EL01N0450G11.d mRNA sequence	-0.01	
11267	TC361198	GB|AAX96058.1|62733949|AC145322 At5g63940 {Oryza sativa (japonica cultivar-group)}, 6%	0.01	
11331	TC319227	RF|XP_507033.1|51964496|XM_507033 OJ1111_C07.25 gene product {Oryza sativa (japonica cultivar-group)}		-0.02
11350	TC335866	UP|Q6ZPH0_MOUSE (Q6ZPH0) MKIAA1853 protein, 7%		-0.01
11393	TC346416	UP|Q3JI01_BURP1 (Q3JI01) Limonene-1, 2-epoxide hydrolase catalytic domain family, 3%		-0.01
11435	TC319393	UP|Q9BIU2_9ARAC (Q9BIU2) Fibroin 1, 13%		0.01
11442	TC352776	UP|Q3JV00_BURP1 (Q3JV00) Phosphoglycerate mutase family protein, 3%	0.01	
11475	TC356322	UP|LSPA_LEPIC (Q72PS8) Lipoprotein signal peptidase, 5%	0.01	0.01
11479	TC327835	GB|AAT72926.1|50293119|AY656247 17.7 kDa low temperature induced protein {Oryza sativa (japonica cultivar-group)}, 59%	0.01	0.01

11480	TC362001		0.01	
11560	TC341760	Zea mays clone Contig727 mRNA sequence	0.01	-0.01
11568	TC316706	UP|Q33BI2_ORYSA (Q33BI2) Expressed protein, 3%	-0.01	
11569	TC352278	UP|Q40474_TOBAC (Q40474) Axi 1 protein, 11%		0.01
11578	TC359018	UP|Q9MAL6_ARATH (Q9MAL6) T25K16.18, 26%		0.01
11580	TC347272	RF|NP_174226.1|15218889|NM_102672 nucleotide binding {Arabidopsis thaliana}, 33%		0.01
11616	TC349896		-0.01	
11658	TC349665	UP|Q3H3A5_9ACTO (Q3H3A5) Uncharacterised conserved protein UCP005026, 3%	-0.01	0.01
11662	TC339225			-0.01
11668	TC352322	UP|Q6H884_ORYSA (Q6H884) Aspartic acid-rich protein aspolin1-like protein, 51%	-0.01	
11672	TC316448	Zea mays clone Contig591 mRNA sequence	0.01	
11698	TC328001	UP|Q2IMJ3_ANADE (Q2IMJ3) LigA, 6%	-0.01	
11735	TC356344		0.01	
11762	TC346167		0.01	
11769	TC338364	UP|Q6DEE3_XENLA (Q6DEE3) Ctnnal1-prov protein, 3%	0.01	
11829	TC363410	UP|Q6NMK2_ARATH (Q6NMK2) At5g49400, 66%		-0.02
11841	TC351292	GB|AAT68023.1|49618877|AY644637 caffeoyl-CoA O-methyltransferase {Oryza sativa (japonica cultivar-group)}, 91%	0.01	
11862	TC326305	UP|Q6MYW6_ASPFU (Q6MYW6) Basic proline-rich protein, 6%	0.01	
11868	TC332726	UP|Q8T0U6_DROME (Q8T0U6) GH05039p (CG2096-PA, isoform A), 6%		-0.02
11872	TC363760	UP|Q69SJ5_ORYSA (Q69SJ5) NatC N(Alpha)-terminal acetyltransferase, Mak10 subunit-like, 32%	0.01	
11875	TC352238		0.01	

11893	TC334484	UP|Q2IUG2_RHOP2 (Q2IUG2) LipA a lipoprotein, 15%	-0.01	-0.01
11902	TC354271		0.01	
11921	TC341812	UP|Q9T6M0_GLOPA (Q9T6M0) NADH-ubiquinone oxidoreductase subunit 1, 7%		-0.02
11952	TC342579	UP|IAA4_SORBI (P81367) Alpha-amylase inhibitor 4 (SI alpha-4), 73%	-0.01	
11957	TC362025		-0.01	
11989	TC364395		-0.01	
12019	TC326117	RF|XP_507202.1|51964836|XM_507202 P0488B06.44 gene product {Oryza sativa (japonica cultivar-group)}, 76%	0.02	
12034	TC357288	UP|Q8LLB9_HORVU (Q8LLB9) BPM, 36%	-0.02	
12056	TC340673	UP|Q29AF1_DROPS (Q29AF1) GA11058-PA, 6%	-0.01	
12072	TC330905	UP|Q52UU1_9ROSI (Q52UU1) Squamosa promoter binding-like protein, 18%	-0.01	
12076	TC330209	Zea mays clone Contig1032.F mRNA sequence		0.01
12082	TC323380	UP|Q41719_ZEADI (Q41719) Hydroxyproline-rich glycoprotein precursor, 98%	-0.01	
12090	TC325310	UP|IMA1A_ORYSA (Q71VM4) Importin alpha-1a subunit, 44%	-0.01	
12094	TC329792		-0.01	
12107	TC323648	UP|Q9ARE4_FLABI (Q9ARE4) ZF-HD homeobox protein, 49%	-0.01	
12129	-	-	-0.01	
12245	-	-		0.01
12264	TC330733	UP|Q7XTF6_ORYSA (Q7XTF6) OJ991214_12.11 protein, 90%		0.01
12277	TC330862	RF|XP_507356.1|51978958|XM_507356 OJ1014_E09.28 gene product {Oryza sativa (japonica cultivar-group)}, 97%		0.01
12278	TC320067	UP|Q94II1_ARATH (Q94II1) ERD7 protein, 30%		0.01

12280	TC330121	RF|XP_473062.1|50926175|XM_473062 {Oryza sativa (japonica cultivar-group)}, 90%		0.01
12306	TC368330	UP|Q6EPQ3_ORYSA (Q6EPQ3) SPX (SYG1/Pho81/XPR1) domain-containing protein-like, 25%		0.01
12308	TC349932	UP|Q69XQ3_ORYSA (Q69XQ3) KH domain-containing protein / zinc finger protein-like, 30%		0.01
12331	TC351382	RF|YP_009207.1|46852414|NC_005869 V {Porcine adenovirus A} , 4%		0.01
12372	TC366191	GB|CAA54234.1|472869|ATARPMR ARP protein {Arabidopsis thaliana}, 26%		0.01
12373	TC363625			-0.01
12380	TC334528		0.01	0.01
12389	TC355914	UP|Q697H7_9HEMI (Q697H7) ATP synthase F0 subunit 8, 21%	-0.01	
12438	TC347648	UP|Q9XFG7_WHEAT (Q9XFG7) Isoamylase 1, 5%	-0.01	
12441	TC349324	Zea mays clone Contig71 mRNA sequence		0.01
12446	TC357440	UP|Q9LL87_MAIZE (Q9LL87) Beta-glucosidase aggregating factor, 13%		0.01
12453	TC333515	UP|YB1E_SCHPO (P87179) Serine-rich protein C30B4.01c precursor, 21%		0.01
12462	TC346415	UP|Q67W96_ORYSA (Q67W96) Kelch repeat containing F-box protein-like, 7%		0.01
12490	TC331265	UP|Q6AWY1_ORYSA (Q6AWY1) Growth-regulating factor 8, 47%	-0.01	-0.01
12500	TC360307	UP|Q9SXG8_ORYSA (Q9SXG8) Dof zinc finger protein, 38%		0.01
12501	TC341524	UP|Q8W529_MAIZE (Q8W529) Methionine synthase, 25%	-0.01	
12594	TC332552	UP|Q7WED7_BORBR (Q7WED7) Branched-chain amino acid ABC transporter, permease protein, 5%		0.01
12602	TC333402	UP|Q53N84_ORYSA (Q53N84) Expressed protein, 98%		0.01
12627	TC319603	Zea mays clone Contig63 mRNA sequence		0.01
12673	TC338711	UP|Q9ZRB5_SOLTU (Q9ZRB5) Ci21B protein, 90%		0.01
12694	TC357114	UP|Q88TX4_LACPL (Q88TX4) Phosphoglycerate mutase, 6%		0.01

12721	TC358826	UP|Q6IMV8_ORYSA (Q6IMV8) Transposase, 10%		0.02
12733	TC347087	UP|Q9FRF5_ORYSA (Q9FRF5) Expressed protein, 39%	-0.02	
12735	TC317134	Zea mays clone Contig604 mRNA sequence		0.01
12740	TC328224	UP|BAB1_DROME (Q9W0K7) Protein bric-a-brac 1, 3%		0.01
12757	TC353870	UP|Q2W275_MAGMM (Q2W275) Predicted hydrolase or acyltransferase, 6%		0.01
12773	TC355122	UP|IAA30_ORYSA (P0C132) Auxin-responsive protein IAA30, 75%	-0.01	
12774	TC340081	UP|Q84Z28_ORYSA (Q84Z28) Abnormal spindle-like protein, 14%		0.01
12827	TC349668		0.01	0.01
12846	TC324229	GB|AAF79875.1|8778876|AC000348 T7N9.15 {Arabidopsis thaliana}, 9%	0.01	0.01
12848	TC348410		0.01	
12853	TC367668			0.01
12858	TC320188	RF|XP_507223.1|51964878|XM_507223 OJ1198_B10.8 gene product {Oryza sativa (japonica cultivar-group)}, 65%	0.01	0.01
12983	TC363925	RF|NP_567636.1|18415765|NM_118299 TOM1 (TOBAMOVIRUS MULTIPLICATION 1) {Arabidopsis thaliana}, 7%		-0.01
13017	TC329711	UP|Q9LN12_ARATH (Q9LN12) T6D22.4, 70%	0.02	
13031	TC343760			-0.01
13037	TC338449	UP|Q9FM40_ARATH (Q9FM40) Emb|CAB87783.1, 60%	0.01	0.01
13083	TC370083	GB|BAD82812.1|56790017|AB182389 CLV1-like LRR receptor kinase {Oryza sativa (japonica cultivar-group)}, 3%	0.01	
13086	TC333266	UP|Q26XN8_XANP2 (Q26XN8) Preprotein translocase SecG subunit, 8%		0.01
13112	TC364911	UP|Q28EK6_XENTR (Q28EK6) CHK1 checkpoint homolog (S. pombe), 14%	0.01	
13115	TC335745	UP|Q7F191_ORYSA (Q7F191) Regulator of nonsense transcripts 1 homolog-like protein, 73%		0.01

13169	TC342801			-0.01
13182	TC362079	UP|Q8LCJ7_ARATH (Q8LCJ7) Flavonol synthase-like protein, 89%		0.01
13190	TC347790		-0.02	
13340	TC372162	UP|Q5ZBZ5_ORYSA (Q5ZBZ5) Potential U2 snRNA pseudouridine synthase-like, 5%		0.01
13386	TC364553	UP|Q7NZE3_CHRVO (Q7NZE3) Protein-N p-phosphohistidine-sugar phosphotransferase, 3%		0.01
13391	TC351748	UP|Q278P5_MYCFV (Q278P5) Helix-turn-helix, Fis-type, 5%		-0.01
13429	TC343148			-0.01
13440	TC367289	UP|Q917L2_9PARA (Q917L2) Nucleocapsid protein, 4%		0.01
13445	TC341091	UP|Q8W1D1_MAIZE (Q8W1D1) Gag-pol, 13%		0.01
13480	TC319635	UP|Q5JMG8_ORYSA (Q5JMG8) Copper chaperone (CCH)-related protein-like, 45%		0.02
13533	TC368953			0.01
13577	TC318978	GB|AAQ65156.1|34365689|BT010533 At1g03360 {Arabidopsis thaliana}, 68%	0.01	
13602	TC354574		0.01	
13611	TC350087	UP|O22453_MAIZE (O22453) Ribsomal protein S4, 20%	0.01	
13633	TC357094		0.01	
13649	TC330224		0.02	
13653	TC366047	UP|Q5U7K6_9POAL (Q5U7K6) Metallothionein-like protein, 93%		-0.02
13705	TC362387		0.01	
13710	TC367078			0.01
13721	TC351034	Zea mays clone EL01N0519H11.c mRNA sequence		0.01
13792	TC350517	UP|Q940V0_ARATH (Q940V0) T23O15.3/T23O15.3, 74%	0.01	

13830	TC367680		0.01	0.01
13844	-	-	0.01	
13851	TC335272	RF|NP_193367.2|42566868|NM_117729 SPK1 (SPIKE1) {Arabidopsis thaliana}, 9%	0.01	
13852	TC318233	Zea mays clone EL01N0525C10.d mRNA sequence		0.01
13853	TC346779	UP|Q9FIV1_ARATH (Q9FIV1) Gb|AAF02153.1, 44%		-0.01
13888	TC365811	UP|PDXJ_BACFN (Q5L912) Pyridoxal phosphate biosynthetic protein pdxJ (PNP synthase), 5%	0.01	
13901	TC355702	UP|Q60EC2_ORYSA (Q60EC2) Unknow protein, 83%		0.01
13943	TC368750		0.01	
13976	TC350475	UP|Q3Y6P4_ORYSA (Q3Y6P4) EIN3-like protein 1, 28%		-0.01
13977	TC319345	UP|Q459S3_9BURK (Q459S3) FAD linked oxidase, C-terminal:FAD linked oxidase, N-terminal, 4%		0.01
13978	-	-		0.01
14005	TC316565	UP|Q9LVJ8_ARATH (Q9LVJ8) Arabidopsis thaliana genomic DNA, chromosome 3, P1 clone: MDC16, 15%	0.01	
14021	TC337041	UP|KSG7_ARATH (Q39011) Shaggy-related protein kinase eta, 94%	0.01	
14063	TC324526		0.01	
14095	TC352807	UP|Q9LPI5_ARATH (Q9LPI5) F6N18.17, 64%	0.02	
14096	TC353719	UP|Q9AVR1_9GENT (Q9AVR1) S-adenosyl-L-methionine:salicylic acid carboxyl methyltransferase, 7%	0.01	
14150	TC337798	UP|Q40X28_KINRA (Q40X28) Pantoate-beta-alanine ligase, 4%		0.01
14200	TC331899	RF|NP_001021570.1|71989038|NM_001026399 M01B12.5a {Caenorhabditis elegans}, 5%		0.01
14205	TC338310			0.01
14208	TC318395	UP|Q7XC69_ORYSA (Q7XC69) Expressed protein, 91%	0.01	

14214	TC316467	Zea mays clone Contig88 mRNA sequence	0.01	
14244	TC340894	UP|Q7XBH1_ORYSA (Q7XBH1) Stress-responsive protein, 89%	0.01	-0.02
14288	TC329199	UP|Q304W8_ORYSA (Q304W8) Serine/threonine kinase, 36%	0.01	
14309	TC330845	UP|Q7XAL7_ORYSA (Q7XAL7) Rac GTPase activating protein 3-like protein, 17%	0.01	
14390	TC366561		0.01	
14396	TC367607	Zea mays clone EL01T0203B04.c mRNA sequence	0.01	
14415	TC347002	GB|BAB47035.1|13928206|AB042240 {Triticum aestivum}, 31%		0.01
14421	TC342839	UP|Q9FQ08_ARATH (Q9FQ08) Ku70-like protein, 8%	0.01	
14430	TC343286	UP|Q91YW8_MOUSE (Q91YW8) Pprc1 protein, 5%	0.01	
14467	TC317468	UP|RF2A_ORYSA (Q69IL4) Transcription factor RF2a, 32%	0.01	0.01
14483	TC343822		0.01	
14487	TC322299	UP|FABG_CUPLA (P28643) 3-oxoacyl-[acyl-carrier-protein] reductase, chloroplast precursor, 77%		0.01
14488	TC363429		-0.01	-0.01
14489	TC354046		0.01	
14491	TC330965	UP|Q7XII4_ORYSA (Q7XII4) Remorin-like protein, 67%		0.01
14515	TC361944	UP|PYRE_STRMU (Q8DTV2) Orotate phosphoribosyltransferase, 6%	0.01	
14517	TC321151	UP|Q93X44_LYCES (Q93X44) Protein tyrosine phosphatase, 69%	0.01	
14539	TC329765	RF|XP_469022.1|50917251|XM_469022 expressed protein {Oryza sativa (japonica cultivar-group)}, 16%	0.01	
14655	TC342421		0.01	
14674	TC357032	UP|Q7XTF4_ORYSA (Q7XTF4) OJ991214_12.13 protein, 57%		0.01
14744	TC362561	UP|Q9AV77_ORYSA (Q9AV77) 60S ribosomal protein L17, 96%	0.01	

14753	TC354596	UP|Q40347_MAGSL (Q40347) Globulin precursor, 5%	0.01	
14829	-	-		0.01
14842	TC341957	UP|O80708_ARATH (O80708) F8K4.23 protein, 37%	0.01	-0.01
14847	TC366333	RF|NP_504265.1|17566624|NM_071864 ZC266.2 {Caenorhabditis elegans}, 8%		0.01
14902	-	-		-0.01
14915	TC327356	Zea mays clone EL01T0403G12.d mRNA sequence		0.01
14957	TC319911	UP|Q2QU44_ORYSA (Q2QU44) Expressed protein, 44%		-0.02
14973	TC358632		-0.01	
15021	TC351256	UP|Q84VA0_ORYSA (Q84VA0) E2F dimerization factor, 82%		-0.01
15045	TC334027	UP|O81652_PIMBR (O81652) Phyb1, 64%	-0.02	
15094	TC351515	UP|Q8GU82_ORYSA (Q8GU82) PDR-like ABC transporter, 17%		0.01
15208	TC344955	UP|Q9FIR6_ARATH (Q9FIR6) Gb|AAD25674.1, 71%		0.01
15274	TC356635	UP|RK16_MAIZE (P08528) Chloroplast 50S ribosomal protein L16		0.01
15285	TC329289	Zea mays clone EL01T0206E06.c mRNA sequence		0.01
15288	TC326179	UP|Q9SKD8_ARATH (Q9SKD8) Expressed protein (At2g46420/F11C10.11), 75%		0.01
15315	TC366051			0.01
15382	TC352715	UP|Q2R3H0_ORYSA (Q2R3H0) Expressed protein, 12%		-0.01
15385	TC336567	GB|AAM74492.1|21703099|AY123978 At1g76120/T23E18_5 {Arabidopsis thaliana}, 34%		-0.01
15446	TC331624	UP|Q9M9F8_ARATH (Q9M9F8) F3F9.7, 8%	0.02	
15448	TC341923			0.01
15453	TC354039			0.01

	15476	-	-	-0.01	

Appendix S9. Transcripts with significant abundance change in *Andropogon gerardii* and *Sorghastrum nutans* in response to drought recovery. Differences are based on the two July dates (q-value<0.05). Light grey shaded transcripts exhibited significant abundance level changes in response to both short-term drought-recovery and variation in LWP across the five sampling dates.

**Gene Ontology**		**SPOTID**	**Gene Index**	**Gene Annotation**	**Estimate of Difference**	
					A. gerardii	***S. nutans***
Amino acid metabolism	Degradation	9107	TC336139	RF|XP_506747.1|51963928|XM_506747 OJ1524_D08.17 gene product {Oryza sativa (japonica cultivar-group)}, 53%		-0.39
		138	TC337595	UP|LGUL_CICAR (O49818) Lactoylglutathione lyase, 90%		1.15
		305	TC318646	GB|AAB17995.1|1644427|ATU74610 glyoxalase II {Arabidopsis thaliana}, 93%		-0.81
		6479	TC316141	UP|Q6XC06_MAIZE (Q6XC06) Glyoxalase I		-0.61
		8931	TC337595	UP|LGUL_CICAR (O49818) Lactoylglutathione lyase, 90%		1.14
		8344	TC338549	Zea mays clone EL01N0447H09.c mRNA sequence		-0.85
		11474	TC325321	UP|MCCB_ARATH (Q9LDD8) Methylcrotonoyl-CoA carboxylase beta chain, mitochondrial precursor, 44%		-1.31
		4744	TC317495	UP|ARGI1_ARATH (P46637) Arginase, 93%		-0.67
		5022	TC348477	UP|ARGI1_ARATH (P46637) Arginase, 54%		-0.35
		4844	TC355203	UP|Q94F76_MAIZE (Q94F76) Silencing group B protein		0.97
		1782	TC343761	RF|NP_566838.1|18405808|NM_113768 oxidoreductase, acting on paired donors, with incorporation or reduction of mo		1.26
		2521	TC343761	RF|NP_566838.1|18405808|NM_113768 oxidoreductase, acting on paired donors, with incorporation or reduction of mo		-0.47
		6349	TC325870	UP|Q9ZPK0_DATGL (Q9ZPK0) Thiosulfate sulfurtransferase, 79%		-0.75
		3112	TC316310	UP|Q6V9T1_ORYSA (Q6V9T1) Glycine dehydrogenase P protein, 56%		-0.43
	Synthesis	4461	TC367699	UP|O24566_MAIZE (O24566) 3-phosphoshikimate 1-carboxyvinyltransferase		0.48

3326	TC316746	UP|Q5NTH4_ORYSA (Q5NTH4) Shikimate kinase 1, 95%		1.08
1969	TC317448	UP|Q9LXU2_ARATH (Q9LXU2) Anthranilate phosphoribosyltransferase-like protein, 43%		-0.66
4904	TC337288	GB|BAD54377.1|53793170|AP005382 asparagine synthetase {Oryza sativa (japonica cultivar-group)}, 34%		0.66
525	TC365565	UP|METK_ORYSA (P46611) S-adenosylmethionine synthetase 1		0.85
6272	TC332230	UP|METK_ORYSA (P46611) S-adenosylmethionine synthetase 1, 82%		-0.51
3673	TC322696	GB|AAM65160.1|21593211|AY087619 branched-chain-amino-acid transaminase-like protein {Arabidopsis thaliana}, 48%		0.70
11365	TC356062	UP|Q41768_MAIZE (Q41768) Acetohydroxyacid synthase		0.76
4256	TC328550	UP|AGT23_ARATH (Q9SR86) Alanine--glyoxylate aminotransferase 2 homolog 3, mitochondrial precursor, 65%		-0.93
760	TC344923	UP|Q43305_PANMI (Q43305) Aspartate aminotransferase precursor		0.52
4917	TC318365	UP|Q5F4K8_PINPS (Q5F4K8) Aspartate aminotransferase, 77%		0.74
5210	TC318365	UP|Q5F4K8_PINPS (Q5F4K8) Aspartate aminotransferase, 77%		1.07
6074	TC318365	UP|Q5F4K8_PINPS (Q5F4K8) Aspartate aminotransferase, 77%		-0.41
9553	TC327241	UP|AATC_ORYSA (P37833) Aspartate aminotransferase, cytoplasmic		-0.41
5343	TC366738	UP|Q2QVC1_ORYSA (Q2QVC1) Argininosuccinate synthase (Citrulline--aspartate ligase) , 40%		0.67
658	TC333854	UP|O24447_ARATH (O24447) Carbamoyl phosphate synthetase small subunit, 41%		0.59
1285	TC329858	UP|Q8LJP7_9POAL (Q8LJP7) Phosphoribosyl pyrophosphate synthetase		0.53
5532	TC316719	UP|Q5UJF9_ORYSA (Q5UJF9) Beta-cyanoalanine synthase, 86%		-0.42
5678	TC364009	UP|CYSK_MAIZE (P80608) Cysteine synthase		-0.65
11540	TC323544	UP|Q9XEA9_ORYSA (Q9XEA9) Cysteine synthase, 94%		-0.30
1402	TC364188	RF|NP_195146.1|15235282|NM_119583 amino acid binding {Arabidopsis thaliana}, 48%		0.61

		3104	TC351802	Zea mays clone EL01N0552F04.c mRNA sequence		-0.33
		11888	TC350146	UP|IAA6_ORYSA (Q8LQ74) Auxin-responsive protein IAA6 (Indoleacetic acid-induced protein 6), 46%		0.53
		11889	TC330409	UP|SERB_ARATH (O82796) Phosphoserine phosphatase, chloroplast precursorase, 13%		-0.86
C1-metabolism	Methylenetetrahydrofolate dehydrogenase & Methenyltetrahydrofolate	11370	TC336767	UP|Q9M090_ARATH (Q9M090) Kinase binding protein-like, 29%		-0.65
		4966	TC330600	Zea mays clone EK07D2304H10.c mRNA sequence		0.66
	S-(hydroxymethyl)glutathione dehydrogenase & S-(hydroxymethyl)glut	1703	TC330451	UP|ADHX_MAIZE (P93629) Alcohol dehydrogenase class 3		-0.72
Cell wall	Cell wall proteins	6827	TC344002	RF|XP_507353.1|51978952|XM_507353 P0496D04.23-2 gene product {Oryza sativa (japonica cultivar-group)}, 77%		0.77
	Cellulose synthesis	5529	TC369104	UP|Q9LLI1_MAIZE (Q9LLI1) Cellulose synthase-9		-0.32
	Modification	3293	TC322932	UP|Q94KT6_MAIZE (Q94KT6) Alpha-expansin 2		0.43
		12495	TC320186	UP|P93671_HORVU (P93671) Xyloglucan endotransglycosylase (XET), 92%		-0.39
Cell	Cycle	6600	TC316094	UP|Q41734_MAIZE (Q41734) Cyclin IaZm		0.71
		1738	TC329729	GB|AAC39444.1|3080738|ATU77365 pasticcino 1-A {Arabidopsis thaliana}, 36%		-0.77
		4185	TC342752	UP|PPIL3_CRYNE (Q5KHA8) Peptidyl-prolyl cis-trans isomerase-like 3, 95%		0.36
		6023	TC371041	UP|CYPH_MAIZE (P21569) Peptidyl-prolyl cis-trans isomerase		-0.46
		6154	TC371041	UP|CYPH_MAIZE (P21569) Peptidyl-prolyl cis-trans isomerase		-0.61
		7477	TC347403	RF|NP_914824.1|34906954|NM_189935 rapamycin-binding protein-like {Oryza sativa (japonica cultivar-group)}, 86%		-0.40

		10950	TC371041	UP|CYPH_MAIZE (P21569) Peptidyl-prolyl cis-trans isomerase		-0.64
		11756	TC316397	Zea mays clone EL01N0530F05.c mRNA sequence		-0.13
		12521	TC371041	UP|CYPH_MAIZE (P21569) Peptidyl-prolyl cis-trans isomerase		-0.55
	Division	851	TC369924	UP|Q84YE5_SORBI (Q84YE5) Cyclin-dependent kinase-like protein, 45%		1.34
		934	TC323523	UP|RRFC_SPIOL (P82231) Ribosome recycling factor, chloroplast precursor, 71%		0.81
		3770	TC316332	UP|Q6Z8N6_ORYSA (Q6Z8N6) Protein cdc2 kinase		1.16
		4255	TC330193	UP|Q8L6H8_ORYSA (Q8L6H8) SMC4 protein, 40%		-0.89
		6055	TC359645	UP|Q7XE16_ORYSA (Q7XE16) AAA family ATPase, CDC48 subfamily, 98%		1.01
		6304	TC328183	Zea mays clone EL01N0526G04.d mRNA sequence		-0.54
		9362	TC343425	UP|Q7XE16_ORYSA (Q7XE16) AAA family ATPase, CDC48 subfamily, 23%		-0.52
		10500	TC363584	RF|NP_173417.2|30686578|NM_101843 Ran GTPase binding {Arabidopsis thaliana}, 70%	1.08	
	Organisation	876	TC364898	UP|TBB1_MAIZE (P18025) Tubulin beta-1 chain		0.91
		929	TC356501	UP|TBA3_MAIZE (P22275) Tubulin alpha-3 chain		1.29
		962	TC367492	UP|TBA1_MAIZE (P14640) Tubulin alpha-1 chain		1.00
		1005	TC316054	UP|Q5XPX5_SACOF (Q5XPX5) Actin		1.10
		1222	TC347779	GB|BAC78565.1|32352144|AB110173 ankyrin {Oryza sativa (japonica cultivar-group)}, 80%		0.88
		1734	TC356501	UP|TBA3_MAIZE (P22275) Tubulin alpha-3 chain		1.86
		1822	TC329080	UP|Q941F3_ARATH (Q941F3) AT4g01710/T15B16_22, 94%		-1.05
		2446	TC360078	UP|TBB5_MAIZE (Q43697) Tubulin beta-5 chain		-0.57
		2887	TC357183	UP|PROF4_MAIZE (O22655) Profilin-4 (ZmPRO4)		-0.57
		2996	TC339557	UP|TBB7_MAIZE (Q41784) Tubulin beta-7 chain		0.79

		3150	TC335890	UP|ACT7_ORYSA (P17300) Actin-7, 18%		-0.40
		3455	TC344584	UP|Q5XPX5_SACOF (Q5XPX5) Actin		0.87
		3537	TC329623	RF|NP_568836.2|30696705|NM_125000 ATARP8 (ACTIN-RELATED PROTEIN 8) structural constituent of cytoskeleton {Arab		-0.60
		3643	TC356501	UP|TBA3_MAIZE (P22275) Tubulin alpha-3 chain	0.73	0.85
		4113	TC348153	UP|O22470_ORYSA (O22470) GDP dissociation inhibitor protein OsGDI1, 68%		-0.92
		4128	TC316520	UP|Q84P56_TOBAC (Q84P56) TGB12K interacting protein 2, 81%		0.80
		4200	TC333132	UP|Q43863_MAIZE (Q43863) Annexin p33		0.91
		4481	TC367661	UP|Q2QPX3_ORYSA (Q2QPX3) Potyvirus VPg interacting protein, 87%		0.45
		4845	TC364898	UP|TBB1_MAIZE (P18025) Tubulin beta-1 chain		1.14
		4858	TC347779	GB|BAC78565.1|32352144|AB110173 ankyrin {Oryza sativa (japonica cultivar-group)}, 80%		1.34
		5118	TC322283	Zea mays clone Contig305 mRNA sequence		-0.23
		5227	TC330905	UP|Q52UU1_9ROSI (Q52UU1) Squamosa promoter binding-like protein, 18%		-1.06
		5684	TC327439	UP|PROF5_MAIZE (Q9FR39) Profilin-5 (ZmPRO5)		-0.77
		5856	TC319498	UP|Q2RBQ8_ORYSA (Q2RBQ8) Expressed protein, 89%		-0.76
		6903	TC356501	UP|TBA3_MAIZE (P22275) Tubulin alpha-3 chain	1.04	
		7243	TC356501	UP|TBA3_MAIZE (P22275) Tubulin alpha-3 chain		1.00
		7950	TC366285	UP|ACT1_MAIZE (P02582) Actin-1		-0.76
		8888	TC342814	UP|ACT1_ORYSA (P13362) Actin-1		-0.53
		9322	TC344584	UP|Q5XPX5_SACOF (Q5XPX5) Actin		-0.43
		9428	TC327439	UP|PROF5_MAIZE (Q9FR39) Profilin-5 (ZmPRO5)		-0.77
		10174	TC339557	UP|TBB7_MAIZE (Q41784) Tubulin beta-7 chain		-0.41

		10227	TC359833	UP|Q2QLT8_ORYSA (Q2QLT8) Cofilin/tropomyosin-type actin-binding protein		-0.65
		10463	TC339557	UP|TBB7_MAIZE (Q41784) Tubulin beta-7 chain		-0.30
		10474	-	-		-0.49
		11311	TC339557	UP|TBB7_MAIZE (Q41784) Tubulin beta-7 chain		0.45
		11649	TC316008	UP|ACT3_ORYSA (P17299) Actin-3		-0.51
		12573	TC354205	UP|ADF3_MAIZE (Q41764) Actin-depolymerizing factor 3		-0.54
	Vesicle transport	302	TC366946	UP|Q9SV20_ARATH (Q9SV20) Beta-COP-like protein, 21%		-0.85
		1208	TC323514	UP|Q69WS1_ORYSA (Q69WS1) Synaptobrevin-like protein (Synaptobrevin 1)		0.61
		6828	TC333972	RF|XP_506688.1|51963810|XM_506688 OJ1217_F02.19 gene product {Oryza sativa (japonica cultivar-group)}		1.24
		7140	TC316498	UP|Q8S0N4_ORYSA (Q8S0N4) Vesicle transport v-SNARE (Vesicle soluble NSF attachment protein receptor) protein-lik		-0.54
		8364	TC324979	UP|Q2QXJ7_ORYSA (Q2QXJ7) Syntaxin 81, 66%		0.41
Development	Storage proteins	324	TC325204	UP|Q9SYT3_MAIZE (Q9SYT3) Azs22-12 (22kD alpha zein 5)		-0.67
		6746	TC318434	UP|Q946V8_MAIZE (Q946V8) 19kD alpha zein D1		-0.29
		11553	TC364534	UP|Q946V6_MAIZE (Q946V6) 19kD alpha zein B1		-0.50
	Unspecified	132	TC360988	UP|Q654W0_ORYSA (Q654W0) G-box binding protein-like, 94%		0.81
		1022	TC332106	Zea mays clone EL01N0526B05.d mRNA sequence		0.50
		4156	TC327894	UP|Q8LQG0_ORYSA (Q8LQG0) Leaf senescence protein-like, 36%		0.89
		5265	TC331035	Zea mays clone EL01N0562H06.d mRNA sequence		0.75
		5266	TC358593			-0.45
		8170	TC318199	UP|Q5ZCB3_ORYSA (Q5ZCB3) Seven in absentia protein-like, 17%		-0.51

		9276	TC333326	UP|Q84V71_MAIZE (Q84V71) M4 protein		0.66
		9975	TC329769	UP|Q4QWQ6_SACOF (Q4QWQ6) NAC23, 98%	0.94	
DNA	Repair	1726	TC316705	UP|Q9STA6_LYCES (Q9STA6) RAD23 protein, 80%		0.94
		2148	TC336128	UP|Q761Y5_ORYSA (Q761Y5) BRI1-KD interacting protein 129, 96%		-0.69
		4656	TC326759	UP|Q5VQ75_ORYSA (Q5VQ75) DNA repair protein XRCC1-like, 85%		-0.47
		4806	TC319104	Zea mays clone EL01N0511E01.c mRNA sequence		1.53
		6773	TC325299	GB|AAF14582.1|6503086|AF188623 nucleotide excision repair protein XP-D homolog {Arabidopsis thaliana}, 15%		-0.56
	Synthesis/chromatin structure	254	TC341938	UP|Q84TG1_ARATH (Q84TG1) At3g09720, 75%		-0.67
		479	TC352342	UP|Q93VJ8_ARATH (Q93VJ8) AT5g11200/F2I11_90 (AT5g11170/F2I11_60), 57%		0.90
		3402	TC355062	UP|Q93VJ8_ARATH (Q93VJ8) AT5g11200/F2I11_90 (AT5g11170/F2I11_60), 98%		-0.99
		3430	TC332041	UP|Q5S1P5_EPICO (Q5S1P5) DNA polymerase delta p12 subunit, 41%		-0.40
		6093	TC320791	UP|Q5ZPI9_ORYSA (Q5ZPI9) Topoisomerase 6 subunit A, 98%		-0.36
		6631	TC350388	GB|AAO24587.1|27808614|BT003155 At1g67320 {Arabidopsis thaliana}, 82%		-0.94
		7053	TC342004	UP|Q9SX04_MAIZE (Q9SX04) Replication origin activator 2, 23%		-1.10
		11429	TC327736	UP|O65573_ARATH (O65573) PRL1-associated protein-like protein, 25%		-1.01
		485	TC357259	UP|H2A_MAIZE (P40280) Histone H2A		1.22
		534	TC341422	UP|Q811M0_MOUSE (Q811M0) Hist1h4h protein, 97%		0.99
		657	TC325723	UP|H2AV3_ORYSA (Q84MP7) Probable histone H2A variant 3		0.63
		915	TC359346	UP|Q4ABW1_BRARP (Q4ABW1) 4D11_26, 37%		0.41
		933	TC336265	UP|H2B1_WHEAT (P27807) Histone H2B, 98%		0.87
		1202	TC335637	UP|Q2V2V7_ARATH (Q2V2V7) Protein At5g64400, 48%		0.62

1242	TC329978	UP|H2B2_MAIZE (P30756) Histone H2B.2		0.47
1662	TC327070	UP|Q6LB28_LYCES (Q6LB28) Histone H3 variant H3.3		-0.67
1717	TC359088	UP|Q76MV0_TOBAC (Q76MV0) H3 histone		0.58
1841	TC341191	UP|Q6LB28_LYCES (Q6LB28) Histone H3 variant H3.3		-0.88
2133	TC327808	UP|Q76MV0_TOBAC (Q76MV0) H3 histone		0.90
2337	TC352556	UP|H2A3_ORYSA (Q6ZL42) Probable histone H2A.3		-0.46
2552	TC345967	UP|Q76N07_SOLME (Q76N07) Histone H4-like protein		0.50
2970	TC316760	UP|H1_MAIZE (P23444) Histone H1		-0.19
3265	TC364001	UP|H2A_MAIZE (P40280) Histone H2A, 57%		0.89
3337	TC359088	UP|Q76MV0_TOBAC (Q76MV0) H3 histone		0.83
3358	TC335527	UP|H2B5_MAIZE (P54348) Histone H2B, 98%		0.61
3397	TC365788	UP|H2A_MAIZE (P40280) Histone H2A		0.85
3652	TC327808	UP|Q76MV0_TOBAC (Q76MV0) H3 histone		0.92
3684	TC345524	UP|H2B2_MAIZE (P30756) Histone H2B.2		0.86
3690	TC330266	UP|Q76MV0_TOBAC (Q76MV0) H3 histone		0.94
3775	TC346327	UP|Q76N07_SOLME (Q76N07) Histone H4-like protein		0.66
4162	TC329978	UP|H2B2_MAIZE (P30756) Histone H2B.2		0.87
4173	TC345256	UP|Q4SKJ3_TETNG (Q4SKJ3) Chromosome undetermined SCAF14565, whole genome shotgun sequence, 47%		0.89
4591	TC347498	UP|Q76MV0_TOBAC (Q76MV0) H3 histone		-0.58
4823	TC365788	UP|H2A_MAIZE (P40280) Histone H2A		1.24
4852	TC336265	UP|H2B1_WHEAT (P27807) Histone H2B, 98%		0.58

		4898	TC327808	UP|Q76MV0_TOBAC (Q76MV0) H3 histone		0.61
		4941	TC365788	UP|H2A_MAIZE (P40280) Histone H2A		-0.42
		4945	TC336265	UP|H2B1_WHEAT (P27807) Histone H2B, 98%		0.91
		4985	TC365788	UP|H2A_MAIZE (P40280) Histone H2A		0.75
		4987	TC327808	UP|Q76MV0_TOBAC (Q76MV0) H3 histone		0.78
		5322	TC331917	UP|Q2QPG9_ORYSA (Q2QPG9) Histone h2a. [norway spruce, picea excelsa, 93%		0.50
		5337	TC321087	UP|H2A_MAIZE (P40280) Histone H2A, 81%		-0.52
		5344	TC336265	UP|H2B1_WHEAT (P27807) Histone H2B, 98%		0.65
		6032	TC342331	UP|H2B1_MAIZE (P30755) Histone H2B.1		-0.51
		6034	TC329978	UP|H2B2_MAIZE (P30756) Histone H2B.2		-0.43
		6155	TC365788	UP|H2A_MAIZE (P40280) Histone H2A		0.80
		6166	TC355183	UP|H2B2_MAIZE (P30756) Histone H2B.2		-0.56
		6490	TC316760	UP|H1_MAIZE (P23444) Histone H1		0.84
		6555	TC318413	UP|H2B1_MAIZE (P30755) Histone H2B.1		0.92
		6841	TC365788	UP|H2A_MAIZE (P40280) Histone H2A		0.81
		6864	TC345967	UP|Q76N07_SOLME (Q76N07) Histone H4-like protein		0.77
		6886	TC336965	UP|Q8CGN9_MOUSE (Q8CGN9) Histone protein Hist2h3c1 (H3 histone, family 2), 78%		0.64
		6970	-	-		-0.37
		7160	TC330119	UP|H2B1_WHEAT (P27807) Histone H2B, 97%		-0.39
		7276	TC365788	UP|H2A_MAIZE (P40280) Histone H2A		-0.59
		7635	TC365788	UP|H2A_MAIZE (P40280) Histone H2A		-1.14

		8006	TC355183	UP|H2B2_MAIZE (P30756) Histone H2B.2	1.37	
		8046	TC336265	UP|H2B1_WHEAT (P27807) Histone H2B, 98%		-0.56
		8060	TC327070	UP|Q6LB28_LYCES (Q6LB28) Histone H3 variant H3.3		-0.49
		8109	TC342976	UP|H2AV3_ORYSA (Q84MP7) Probable histone H2A variant 3		0.67
		8359	TC328939	UP|H2B1_WHEAT (P27807) Histone H2B, 98%		-0.37
		8464	TC341533	UP|Q76N07_SOLME (Q76N07) Histone H4-like protein		-0.39
		8670	TC345256	UP|Q4SKJ3_TETNG (Q4SKJ3) Chromosome undetermined SCAF14565, whole genome shotgun sequence, 47%		-0.78
		8972	TC357259	UP|H2A_MAIZE (P40280) Histone H2A		-0.65
		9224	TC338271	UP|Q76N07_SOLME (Q76N07) Histone H4-like protein		0.73
		9258	TC355183	UP|H2B2_MAIZE (P30756) Histone H2B.2		0.69
		9287	TC345967	UP|Q76N07_SOLME (Q76N07) Histone H4-like protein		0.60
		9393	TC359346	UP|Q4ABW1_BRARP (Q4ABW1) 4D11_26, 37%		-0.30
		9603	TC341533	UP|Q76N07_SOLME (Q76N07) Histone H4-like protein		0.50
		9715	TC327808	UP|Q76MV0_TOBAC (Q76MV0) H3 histone		-0.57
		9748	TC357259	UP|H2A_MAIZE (P40280) Histone H2A		1.46
		10022	TC329978	UP|H2B2_MAIZE (P30756) Histone H2B.2		-0.48
		10048	TC327070	UP|Q6LB28_LYCES (Q6LB28) Histone H3 variant H3.3		-0.40
		10124	TC327808	UP|Q76MV0_TOBAC (Q76MV0) H3 histone		-0.46
		10140	TC359148	UP|H2B1_WHEAT (P27807) Histone H2B, 98%		0.94
		10541	TC327070	UP|Q6LB28_LYCES (Q6LB28) Histone H3 variant H3.3		-0.41
		10891	TC327808	UP|Q76MV0_TOBAC (Q76MV0) H3 histone		-0.79

		10977	TC336265	UP|H2B1_WHEAT (P27807) Histone H2B, 98%		-0.61
		11256	TC345967	UP|Q76N07_SOLME (Q76N07) Histone H4-like protein		0.96
		11327	TC363966	UP|Q76N07_SOLME (Q76N07) Histone H4-like protein		-0.35
		11339	TC355183	UP|H2B2_MAIZE (P30756) Histone H2B.2		0.51
		11660	TC329978	UP|H2B2_MAIZE (P30756) Histone H2B.2		-0.55
		11712	TC329978	UP|H2B2_MAIZE (P30756) Histone H2B.2		-0.78
		12157	TC335527	UP|H2B5_MAIZE (P54348) Histone H2B, 98%		-0.44
		12482	TC327070	UP|Q6LB28_LYCES (Q6LB28) Histone H3 variant H3.3		-0.78
		12488	TC327070	UP|Q6LB28_LYCES (Q6LB28) Histone H3 variant H3.3		-0.56
		12532	TC349142	UP|H2B5_MAIZE (P54348) Histone H2B		-0.52
		12565	TC332934	UP|Q2QPG9_ORYSA (Q2QPG9) Histone h2a. [norway spruce, picea excelsa, 96%		-0.55
		11570	TC341955	UP|Q3LAG0_WHEAT (Q3LAG0) Transposase-related protein w-gary2, 10%		-0.75
	Unspecified	981	TC365295			0.94
		2313	TC315881	UP|Q42419_MAIZE (Q42419) MudrA protein		-0.79
		4075	TC323772	RF|XP_506641.1|51963722|XM_506641 P0523B07.38-1 gene product {Oryza sativa (japonica cultivar-group)}		-0.70
		5859	TC330704	UP|Q3EAL4_ARATH (Q3EAL4) Protein At3g52050, 44%		-0.99
		9419	TC350213	UP|Q9M2L7_ARATH (Q9M2L7) Helicase-like protein, 4%		-0.70
		11292	TC341756			-0.79
Fermentation	PDC	4821	TC326112	UP|Q9LF46_ARATH (Q9LF46) 2-hydroxyphytanoyl-CoA lyase-like protein, 14%		0.66
Gluconeogenese/ glyoxylate cycle	Pyruvate dikinase	14748	TC335589	GB|1VBG_A|62738111|1VBG_A Chain A, Pyruvate Phosphate Dikinase From Maize. {Zea mays}, 24%		-0.78

Glycolysis	Enolase	80	TC335086	UP|ENO2_MAIZE (P42895) Enolase 2		0.52
		5687	TC369719	UP|ENO2_MAIZE (P42895) Enolase 2		-0.70
		5708	TC369719	UP|ENO2_MAIZE (P42895) Enolase 2		-1.08
		9682	TC369719	UP|ENO2_MAIZE (P42895) Enolase 2		-0.59
	Glyceraldehyde 3-phosphate dehydrogenase	511	TC334343	UP|Q37265_PINSY (Q37265) Glyceraldehyde-3-phosphate dehydrogenase precursor, 35%		0.85
		4301	TC367854	UP|G3PD_MAIZE (Q09054) Glyceraldehyde-3-phosphate dehydrogenase, cytosolic 2		-0.58
	PEPCase	494	TC339812	UP|Q9SAZ6_MAIZE (Q9SAZ6) Phosphoenolpyruvate carboxylase		0.82
		2065	TC339812	UP|Q9SAZ6_MAIZE (Q9SAZ6) Phosphoenolpyruvate carboxylase		1.02
		10422	TC339812	UP|Q9SAZ6_MAIZE (Q9SAZ6) Phosphoenolpyruvate carboxylase		-0.72
		11127	TC361054	UP|Q43267_MAIZE (Q43267) PEP carboxylase		-0.47
	PEPCK	10155	TC325108	UP|Q5EC59_MAIZE (Q5EC59) Phosphoenolpyruvate carboxylase kinase 1		-0.81
	PGM	9115	TC352484	UP|PGM_SCHPO (O74374) Probable phosphoglucomutase (Glucose phosphomutase) (PGM) , 14%		-0.66
	PK	7747	TC365959	UP|Q2RAK2_ORYSA (Q2RAK2) Pyruvate kinase		0.77
	Pyrophosphate-fructose-6-P phosphotransferase	4897	TC330834	RF|NP_192313.2|30679628|NM_116642 6-phosphofructokinase {Arabidopsis thaliana}, 27%		0.74
		5048	TC323414	Zea mays clone Contig720.F mRNA sequence		0.42
	TPI	11337	TC367984	GB|AAB81110.1|168647|MZETPI2 triosephosphate isomerase 1 {Zea mays}		-0.53
	UGPase	1722	TC316102	UP|Q6Y643_BAMOL (Q6Y643) UDP-glucose pyrophosphorylase		0.99
Hormone metabolism	Auxin	3447	TC318991	UP|Q52QX9_MANES (Q52QX9) Aldo/keto reductase AKR, 74%		-0.34
		6642	TC336619	UP|IAA19_ORYSA (Q6AT33) Auxin-responsive protein IAA19, 43%		-0.48
		7476	-	-		-0.61
		2466	TC327424	UP|Q6U8C6_WHEAT (Q6U8C6) ETTIN-like auxin response factor, 36%		-0.54

		2475	TC328632	UP|Q6YZX7_ORYSA (Q6YZX7) Auxin efflux carrier protein-like, 76%		-0.45
		10993	TC338370	UP|PIN1B_ORYSA (P0C0X5) Probable auxin efflux carrier component 1b (OsPIN1b), 45%		-0.50
	Brassinosteroid	7249	TC346307	UP|Q942F3_ORYSA (Q942F3) Extra sporogenous cells-like, 45%		-0.69
		875	TC337393	UP|Q69JI7_ORYSA (Q69JI7) BHLH protein family-like, 47%		0.91
		3128	TC341309	UP|C90D2_ORYSA (Q94IW5) Cytochrome P450 90D2 (C6-oxidase) , 10%		-0.61
		3955	TC354772	UP|C90D2_ORYSA (Q94IW5) Cytochrome P450 90D2 (C6-oxidase) , 24%		-0.51
		9421	TC321776	UP|Q84YE6_SORBI (Q84YE6) Cytochrome P450-like protein, 93%		0.90
		2370	TC337966	Zea mays clone Contig708.F mRNA sequence		-0.57
	Ethylene	1911	TC333057	Zea mays clone Contig194 mRNA sequence		-0.72
		2350	TC345632	UP|Q9LXT3_ARATH (Q9LXT3) Transcriptional coactivator-like protein (AT3g58680)		0.93
Lipid metabolism	FA synthesis and FA elongation.ACP protein	8510	TC328449	UP|Q41765_MAIZE (Q41765) Acyl carrier protein, 96%		-0.61
		6138	-	-		-0.87
		9160	TC340273	UP|Q8RVT5_PANGI (Q8RVT5) Acyl-CoA-binding protein		-0.48
		11955	TC330097	UP|Q9ZRC2_ARATH (Q9ZRC2) Acyl-CoA binding protein, 75%		-0.74
		11258	-	-		-0.34
		7069	TC331051	RF|XP_506198.1|51963302|XM_506198 OJ1014_E09.29 gene product {Oryza sativa (japonica cultivar-group)}		1.06
	Lipid degradation	3256	-	-		-0.36
		5625	TC362864	UP|Q5U7K9_9POAL (Q5U7K9) Stem-specific protein, 61%		-0.35
	Lipid transfer proteins etc	9716	TC321032	UP|Q2PCD1_WHEAT (Q2PCD1) Type 1 non specific lipid transfer protein precursor, 95%		1.07
	Phospholipid	7576	TC345201	UP|Q6QA26_ORYSA (Q6QA26) Phosphoethanolamine N-methyltransferase, 19%		0.91

	synthesis	12680	TC324729	UP|Q5N7U2_ORYSA (Q5N7U2) Phospholipid/glycerol acyltransferase-like protein, 39%		-0.90
		2403	TC337701	GB|AAD29709.2|71164865|AF140496 cholinephosphate cytidylyltransferase {Oryza sativa (japonica cultivar-group)},		1.04
Major CHO metabolism	Degradation	964	TC369323	UP|Q5F305_SOYBN (Q5F305) Beta-amylase, 19%		0.84
		12873	TC330885	UP|TPT_MAIZE (P49133) Triose phosphate/phosphate translocator, chloroplast precursor (cTPT), 33%		-0.76
		5640	TC361097	UP|SCRK2_MAIZE (Q6XZ78) Fructokinase-2 (ZmFRK2)		-0.42
		6439	TC319752	UP|NOL10_BRARE (Q802W4) Nucleolar protein 10, 3%		0.55
		2748	TC338178	UP|SUS1_MAIZE (P04712) Sucrose synthase 1, 39%		-0.50
		2832	TC315871	UP|SUS2_MAIZE (P49036) Sucrose synthase 2		0.62
		3401	TC315871	UP|SUS2_MAIZE (P49036) Sucrose synthase 2		-0.66
		8815	TC315871	UP|SUS2_MAIZE (P49036) Sucrose synthase 2		0.65
	Synthesis.starch	12100	TC315909	UP|GLGL2_MAIZE (P55234) Glucose-1-phosphate adenylyltransferase large subunit 2, chloroplast precursor, 98%		-0.95
		12742	TC370441	UP|O81387_MAIZE (O81387) Starch branching enzyme IIb		1.13
		3415	TC361986	UP|ADT1_MAIZE (P04709) ADP,ATP carrier protein 1, mitochondrial precursor		0.92
		11368	TC361986	UP|ADT1_MAIZE (P04709) ADP,ATP carrier protein 1, mitochondrial precursor		-0.62
Metal handling	Binding, chelation and storage	8300	TC316359	UP|Q43661_WHEAT (Q43661) Wali7 protein, 82%		-0.67
		776	TC340070	UP|Q6H759_ORYSA (Q6H759) Copper chaperone homolog CCH, 78%		0.78
		3682	TC357882	UP|Q5U7K6_9POAL (Q5U7K6) Metallothionein-like protein, 98%		1.06
		4533	TC357882	UP|Q5U7K6_9POAL (Q5U7K6) Metallothionein-like protein, 98%		0.90
		6557	TC357882	UP|Q5U7K6_9POAL (Q5U7K6) Metallothionein-like protein, 98%		1.02
		10927	TC358604	UP|Q5U7K6_9POAL (Q5U7K6) Metallothionein-like protein, 98%		0.89

		12075	TC342362	UP|Q6J338_9ROSI (Q6J338) Copper chaperone, 84%		-0.50
Minor CHO metabolism	Others	6082	TC330920	UP|Q657Z1_ORYSA (Q657Z1) Carbohydrate kinase-like, 92%		-0.61
		8884	TC320523	UP|Q5SN59_ORYSA (Q5SN59) Ribokinase-like, 88%		-0.30
	Sugar alcohols	5837	TC330138	UP|Q67Y17_ARATH (Q67Y17) MRNA, complete cds, clone: RAFL25-18-P16, 28%		-0.39
	Trehalose	8859	TC341261	UP|Q2TSD4_GOSHI (Q2TSD4) Trehalose 6-phosphate synthase, 19%		-0.59
		899	TC344391	UP|Q1W5S5_PENAM (Q1W5S5) Ramosa 3, 98%		0.52
		5162	TC343278			1.05
Misc	Acid and other phosphatases	4370	TC318380	UP|Q6J5M7_SOLTU (Q6J5M7) Purple acid phosphatase 1, 84%		-0.78
		8279	TC319626	UP|Q5N7Q9_ORYSA (Q5N7Q9) Phosphatidic acid phosphatase beta-like, 77%		-0.42
		11908	TC365709			-0.69
	Beta 1,3 glucan hydrolases	7941	TC346721	RF|NP_683538.1|22330905|NM_148696 hydrolase, hydrolyzing O-glycosyl compounds {Arabidopsis thaliana}, 15%		-0.82
	Cytochrome P450	885	TC320192	UP|C78A1_MAIZE (P48420) Cytochrome P450 78A1		1.13
		4329	TC322752	UP|C89A2_ARATH (Q42602) Cytochrome P450 89A2 (CYPLXXXIX) (ATH 6-1) , 26%		-0.61
	GCN5-related N-acetyltransferase	4877	TC328698	Zea mays clone EK07D2304B01.c mRNA sequence		0.94
	GDSL-motif lipase	3906	TC319891	UP|Q5Z983_ORYSA (Q5Z983) GDSL-lipase-like, 48%		0.55
		4294	TC347899	UP|Q8RZ61_ORYSA (Q8RZ61) Lipase-like, 50%		-0.66
		5909	TC321327	UP|Q69Y47_ORYSA (Q69Y47) EREBP-like protein, 62%		-0.34
	Gluco-, galacto- and mannosidases	2775	TC329285	RF|XP_469436.1|50918079|XM_469436 beta-glucosidase (with alternative splicing) {Oryza sativa (japonica cultivar-		-0.39
		2635	TC336687	PRF|1303351A|225458|1303351A transferase,glutathione S. {Zea mays}		-0.78
		3772	TC316394	UP|Q9FQA9_MAIZE (Q9FQA9) Glutathione S-transferase GST 30		1.17

	5338	TC336687	PRF|1303351A|225458|1303351A transferase,glutathione S. {Zea mays}		-0.73
	9040	TC327151	UP|Q9FQC1_MAIZE (Q9FQC1) Glutathione S-transferase GST 18, 93%		-0.32
	9071	TC327409	UP|Q9FQC0_MAIZE (Q9FQC0) Glutathione S-transferase GST 19		-0.54
Invertase/pectin methylesterase inhibitor family protein	3103	TC325396	UP|Q2IIH7_ANADE (Q2IIH7) PE-PGRS family protein, 4%		-0.47
Misc2	2369	TC327905	UP|Q84YD8_SORBI (Q84YD8) ATPase-like protein		-0.51
	6845	TC327407	UP|Q6YVJ1_ORYSA (Q6YVJ1) Nodulin-like protein, 53%		1.16
Myrosinases-lectin-jacalin	1879	-	-		-0.73
	2805	TC318654	UP|Q9LL87_MAIZE (Q9LL87) Beta-glucosidase aggregating factor, 13%		0.91
	5713	TC318654	UP|Q9LL87_MAIZE (Q9LL87) Beta-glucosidase aggregating factor, 13%		-0.64
	9096	TC344912	UP|Q2QWD7_ORYSA (Q2QWD7) Jacalin homolog, 17%		-1.62
Nitrilases, *nitrile lyases, berberine bridge enzymes, reticuline oxidases,	3787	TC349301	UP|Q6ZI62_ORYSA (Q6ZI62) Glycosyl transferase-like protein, 94%		1.14
	4134	TC317535	UP|Q3E9P0_ARATH (Q3E9P0) Protein At4g38220, 62%		0.59
	4500	TC349301	UP|Q6ZI62_ORYSA (Q6ZI62) Glycosyl transferase-like protein, 94%		-0.74
O- methyl transferases	2064	TC324790	UP|Q9SU94_ARATH (Q9SU94) Arginine methyltransferase, 47%		0.90
	9654	TC317094	UP|Q9SU94_ARATH (Q9SU94) Arginine methyltransferase, 83%		1.13
Oxidases - copper, flavone etc.	4028	TC321693	RF|NP_176759.1|15218830|NM_105256 amine oxidase/ oxidoreductase {Arabidopsis thaliana}, 84%		0.85
	4816	TC334940	RF|NP_191464.1|15231622|NM_115767 oxidoreductase {Arabidopsis thaliana}, 39%		0.53
Protease inhibitor/seed storage/lipid transfer protein (LTP) family protein	10242	TC341209	UP|O24556_MAIZE (O24556) Physical impedance induced protein, 98%		-0.43
	15226	TC331947	UP|Q40721_ORYSA (Q40721) RCc3 protein, 84%	-2.36	

	Short chain dehydrogenase/reductase (SDR)	2183	TC321060	UP|Q6RVV4_PEA (Q6RVV4) Short-chain dehydrogenase Tic32, 85%		1.14
		2263	TC328591	UP|Q6H7C9_ORYSA (Q6H7C9) Short-chain dehydrogenase/reductase protein-like, 48%		-0.54
		5826	TC326786	UP|Q7QJE4_ANOGA (Q7QJE4) ENSANGP00000019038, 64%		-0.60
	UDP glucosyl and glucoronyl transferases	6883	TC341436	UP|Q69XJ6_ORYSA (Q69XJ6) Glycosylation enzyme-like protein, 89%	1.60	0.61
Mitochondrial electron transport / ATP synthesis	Cytochrome c	7870	TC335038	UP|CYC_MAIZE (P00056) Cytochrome c		0.73
		11232	TC340040	Zea mays clone Contig84 mRNA sequence		-0.54
		9993	TC349725	GB|CAA92107.1|1070356|HVCOXVCMR cytochrome c oxidase, Vc subunit {Hordeum vulgare subsp. vulgare}		0.80
		11439	TC349725	GB|CAA92107.1|1070356|HVCOXVCMR cytochrome c oxidase, Vc subunit {Hordeum vulgare subsp. vulgare}		-0.41
		12775	TC320634	UP|Q9SXV0_ORYSA (Q9SXV0) Cytochrome c oxidase subunit 6b-1 (Cytochrome c oxidase subunit 6b), 77%		0.52
	F1-ATPase	42	TC332628	UP|Q6IY71_WHEAT (Q6IY71) Mitochondrial ATP synthase		-1.01
		1008	TC336130	Zea mays clone EL01T0403D12.c mRNA sequence		0.69
		2539	TC332628	UP|Q6IY71_WHEAT (Q6IY71) Mitochondrial ATP synthase		-0.42
		4997	TC316326	Zea mays clone Contig504 mRNA sequence		0.72
		5528	TC319963	UP|ATP4_IPOBA (Q40089) ATP synthase delta' chain, mitochondrial precursor, 88%		0.90
		6407	TC318927	Zea mays clone EL01N0316B09.c mRNA sequence		0.99
		6947	TC317812	UP|ATP4_IPOBA (Q40089) ATP synthase delta' chain, mitochondrial precursor, 88%		0.79
	NADH-DH	730	TC323979	UP|NUIM_SOLTU (P80269) NADH-ubiquinone oxidoreductase 23 kDa subunit, mitochondrial precursor, 78%		-0.48
		3620	TC335018	UP|Q7XBW1_ORYSA (Q7XBW1) Expressed protein, 98%		0.90

		3698	TC363891	UP|Q8LGE7_ARATH (Q8LGE7) NADH:ubiquinone oxidoreductase-like protein (At5g18800), 94%		-0.47
		5072	TC315950	RF|XP_506513.1|51963608|XM_506513 P0503D09.102 gene product {Oryza sativa (japonica cultivar-group)}		0.48
		6101	TC315950	RF|XP_506513.1|51963608|XM_506513 P0503D09.102 gene product {Oryza sativa (japonica cultivar-group)}		-0.52
		8348	TC337162	UP|Q9FIJ2_ARATH (Q9FIJ2) NADH dehydrogenase 10.5K chain-like protein, 96%		-0.53
		8748	TC370724	UP|Q4TZT1_GOSHI (Q4TZT1) Fb14, 89%		-0.53
N-metabolism.ammonia metabolism	Glutamate synthase	12643	TC361721	UP|GLTB_MAIZE (P23225) Ferredoxin-dependent glutamate synthase, chloroplast precursor		-1.09
Nucleotide metabolism	Degradation	127	TC321788	Zea mays clone Contig509 mRNA sequence		0.96
		832	TC368240	Zea mays clone EL01N0505C10.c mRNA sequence		0.63
		4517	TC324155	RF|NP_563745.1|18390550|NM_100442 hydrolase {Arabidopsis thaliana}, 98%		-0.26
	Deoxynucleotide metabolism.pseudouridine synthase	4069	TC335546	UP|Q9LY83_ARATH (Q9LY83) TRNA synthase-like protein, 26%		-0.65
		4118	TC355887	Zea mays clone EL01N0449F09.c mRNA sequence		-0.35
		10569	TC332262	Zea mays clone EL01N0422F11.c mRNA sequence		-0.25
	Phosphotransfer and pyrophosphatases	4167	TC357469	UP|Q2P9V0_9MAGN (Q2P9V0) Soluble inorganic pyrophosphatase, 83%		-0.81
		5088	TC316241	UP|IPYR_MAIZE (O48556) Soluble inorganic pyrophosphatase (Pyrophosphate phospho-hydrolase) (PPase) , 93%		1.14
		2471	TC360950	PDB|1PKU_A|61679782|1PKU_A Chain A, Crystal Structure Of Nucleoside Diphosphate Kinase From Rice. {Oryza sativa}		-0.52
		7725	TC332430	UP|NDK1_SACOF (P93554) Nucleoside diphosphate kinase 1		-0.69
	Salvage	14657	TC320666	UP|Q501D4_ARATH (Q501D4) At1g72040, 59%		-0.80
		2754	TC316151	UP|Q9XGC6_MAIZE (Q9XGC6) Adenosine kinase		-0.29

		4099	TC316151	UP|Q9XGC6_MAIZE (Q9XGC6) Adenosine kinase		-0.40
	Synthesis	4839	TC362876	UP|Q8VZW9_VIGUN (Q8VZW9) Succinoaminoimidazolecarboximide ribonucleotide synthetase, 71%		1.16
OPP	Oxidative PP	2121	TC315976	UP|Q7FRX8_ORYSA (Q7FRX8) Cytosolic 6-phosphogluconate dehydrogenase, 98%		-0.50
		3477	TC316529	UP|Q7X7I6_ORYSA (Q7X7I6) Glucose-6-phosphate 1-dehydrogenase (G6PD)		-0.56
		9196	TC339413	UP|Q7X7I6_ORYSA (Q7X7I6) Glucose-6-phosphate 1-dehydrogenase (G6PD) , 34%		0.62
Polyamine metabolism	Degradation	8219	TC317890	UP|PAO_MAIZE (O64411) Polyamine oxidase precursor		0.80
	Synthesis	7055	TC327880	UP|DCAM_MAIZE (O24575) S-adenosylmethionine decarboxylase proenzyme , 38%		-0.73
		8529	TC368082	UP|DCAM_MAIZE (O24575) S-adenosylmethionine decarboxylase proenzyme		0.72
Protein	Assembly and cofactor ligation	1386	TC324362	RF|NP_187678.1|15228351|NM_111903 ATNAP7 {Arabidopsis thaliana}, 65%		0.66
		2963	TC321813	UP|Q8L984_ARATH (Q8L984) NifU-like protein, 73%		1.04
	Amino acid activation	9781	TC369342	UP|Q41754_MAIZE (Q41754) Ubiquitin		-0.78
		3022	TC332341	GB|AAX95729.1|62733612|AC084023 valyl-tRNA synthetase {Oryza sativa (japonica cultivar-group)}, 37%		0.56
		7041	TC315942	UP|O82108_MAIZE (O82108) Seryl-tRNA synthetase		-0.64
		9687	TC320954	UP|O82108_MAIZE (O82108) Seryl-tRNA synthetase, 24%		0.78
		1049	TC319527	UP|Q8LPC9_ORYSA (Q8LPC9) Threonyl-tRNA synthetase		0.72
	Degradation	3555	TC342769	UP|Q43034_PETCR (Q43034) Leucine aminopeptidase (Cytosol aminopeptidase), 41%		-0.47
		6589	TC320398	UP|Q8GVF6_ORYSA (Q8GVF6) Pyrrolidone carboxyl peptidase-like protein, 97%		0.81
		6599	TC320398	UP|Q8GVF6_ORYSA (Q8GVF6) Pyrrolidone carboxyl peptidase-like protein, 97%		1.30
		8524	TC347585	UP|PSA2_ORYSA (Q9LSU2) Proteasome subunit alpha type 2		-0.52
		3087	TC319606	UP|Q5ZDA1_ORYSA (Q5ZDA1) BCS1 protein-like, 88%		0.88

9105	TC322484	UP|ASPRX_ORYSA (P42211) Aspartic proteinase precursor, 44%		-0.27
860	TC339358	UP|Q8SBA4_ORYSA (Q8SBA4) Autophagocytosis protein AUT1-like, 80%		0.75
2514	TC321972	UP|Q655R7_ORYSA (Q655R7) OTU-like cysteine protease-like, 94%		0.39
6588	TC316118	UP|CYSP1_MAIZE (Q10716) Cysteine proteinase 1 precursor		0.85
11915	TC316118	UP|CYSP1_MAIZE (Q10716) Cysteine proteinase 1 precursor		-0.88
12564	TC330002	UP|ORYB_ORYSA (P25777) Oryzain beta chain precursor, 38%		-0.55
7904	TC331803	Zea mays clone E04912707E08.c mRNA sequence		-0.54
9313	TC348818	GB|AAG33975.1|11320956|AF250961 methionine aminopeptidase-like protein {Arabidopsis thaliana}, 67%		0.75
994	TC318915	GB|AAP49525.1|31376397|BT008763 At1g28110 {Arabidopsis thaliana}, 47%		0.78
2042	TC332407	UP|Q8LKH2_ORYSA (Q8LKH2) ATP-dependent Clp protease, 93%		1.05
2143	TC342859	UP|CLPAB_LYCES (P31542) ATP-dependent Clp protease ATP-binding subunit clpA homolog CD4B, chloroplast precursor,		0.75
5350	TC318915	GB|AAP49525.1|31376397|BT008763 At1g28110 {Arabidopsis thaliana}, 47%		0.56
8456	TC329123	Zea mays clone EL01N0372E04.c mRNA sequence		-0.89
576	TC331627	UP|Q41753_MAIZE (Q41753) Ubiquitin fusion protein		0.58
2024	TC342043	GB|BAD33626.1|50726105|AP005579 polyubiquitin 2 {Oryza sativa (japonica cultivar-group)}		-0.73
3611	TC355466	UP|Q6LCT7_MAIZE (Q6LCT7) Ubiquitin fusion protein		0.62
4343	TC329112	UP|Q6LCT7_MAIZE (Q6LCT7) Ubiquitin fusion protein		-0.52
4520	TC316125	UP|O82143_ORYSA (O82143) OsS5a (26S proteasome regulatory particle non-ATPase subunit10), 97%		-0.77
6029	TC321380	UP|Q6K653_ORYSA (Q6K653) Ubiquitin-associated (UBA)/TS-N domain-containing protein-like, 97%		-0.59

		7115	TC345593	UP|Q6K881_ORYSA (Q6K881) Phosphatidylinositol 3-and 4-kinase-like, 24%		-0.73
		11266	TC329112	UP|Q6LCT7_MAIZE (Q6LCT7) Ubiquitin fusion protein		-0.28
		12032	TC355466	UP|Q6LCT7_MAIZE (Q6LCT7) Ubiquitin fusion protein		-0.73
		1308	TC338357	GB|CAA48378.1|22658|ATUBCJEA ubiquitin-conjugating enzyme {Arabidopsis thaliana}		0.86
		2119	TC324643	UP|UBCY_ARATH (P42743) Ubiquitin-conjugating enzyme E2-18 kDa		-0.57
		3400	TC322656	UP|O48555_MAIZE (O48555) Ubiquitin conjugating enzyme		0.84
		4638	TC318336	UP|O48555_MAIZE (O48555) Ubiquitin conjugating enzyme		-0.59
		4746	TC328561	UP|Q94F47_ARATH (Q94F47) At1g64230/F22C12_17 (Ubiquitin conjugating enzyme UBC9A) (Ubiquitinating enzyme)		-0.74
		5050	TC327700	GB|AAL16250.1|16226747|AF428320 AT5g05080/MUG13_6 {Arabidopsis thaliana}, 77%		0.58
		5818	TC335249	RF|NP_565834.1|18404032|NM_129165 ubiquitin conjugating enzyme/ ubiquitin-like activating enzyme {Arabidopsis th		-0.69
		5959	TC362594	UP|Q5XUV4_WHEAT (Q5XUV4) Ubiquitin-conjugating enzyme, 91%		-0.52
		6867	TC335249	RF|NP_565834.1|18404032|NM_129165 ubiquitin conjugating enzyme/ ubiquitin-like activating enzyme {Arabidopsis th		-0.51
		7714	TC322656	UP|O48555_MAIZE (O48555) Ubiquitin conjugating enzyme		-0.92
		8237	TC364608	UP|Q5XUV4_WHEAT (Q5XUV4) Ubiquitin-conjugating enzyme		-0.35
		8458	TC326342	UP|Q70I24_NICBE (Q70I24) SUMO E2 conjugating enzyme SCE1		-0.88
		9751	TC364608	UP|Q5XUV4_WHEAT (Q5XUV4) Ubiquitin-conjugating enzyme		-0.56
		9806	TC364608	UP|Q5XUV4_WHEAT (Q5XUV4) Ubiquitin-conjugating enzyme		-0.64
		10469	TC328274	UP|Q3HVN0_SOLTU (Q3HVN0) Ubiquitin-conjugating enzyme family protein-like, 96%		-0.87
		11320	TC322656	UP|O48555_MAIZE (O48555) Ubiquitin conjugating enzyme		-0.68
		11760	TC343341	UP|O48555_MAIZE (O48555) Ubiquitin conjugating enzyme		-0.57

		12947	TC350852	UP|Q9M551_9ROSI (Q9M551) Polyubiquitin		-0.60
		332	TC324967	UP|Q9FMB2_ARATH (Q9FMB2) Arabidopsis thaliana genomic DNA, chromosome 5, TAC clone:K15E6, 29%		-1.34
		1149	TC320110	UP|Q7XI08_ORYSA (Q7XI08) Auxin-regulated protein-like protein, 94%	0.70	
		3390	TC327047	RF|NP_171642.1|15223384|NM_100017 nucleic acid binding {Arabidopsis thaliana}, 33%		1.20
		4029	TC326909	RF|NP_176574.2|30696917|NM_105064 zinc ion binding {Arabidopsis thaliana}, 49%		0.87
		4340	TC341640	UP|Q6AUV7_ORYSA (Q6AUV7) JmjC domain containing protein, 25%		-0.31
		5932	TC355375	UP|Q9M2V1_ARATH (Q9M2V1) RING finger-like protein (At3g54360/T12E18_50), 67%		-0.67
		6283	TC341780	UP|Q5NAS8_ORYSA (Q5NAS8) C3H2C3 RING-finger protein-like, 23%		-1.02
		12726	TC341311	UP|Q2HT04_MEDTR (Q2HT04) Zinc finger, RING-type, 16%		-0.37
		567	-	-		0.71
		865	TC328917	RF|XP_506618.1|51963694|XM_506618 P0015C07.29 gene product {Oryza sativa (japonica cultivar-group)}, 38%		0.77
		3304	TC358196	UP|Q940D2_LYCES (Q940D2) SKIP5-like protein, 65%		-0.98
		8130	TC358196	UP|Q940D2_LYCES (Q940D2) SKIP5-like protein, 65%		-0.40
		8446	TC361063	RF|XP_506618.1|51963694|XM_506618 P0015C07.29 gene product {Oryza sativa (japonica cultivar-group)}, 37%		-0.66
		11728	TC335181	UP|Q6K235_ORYSA (Q6K235) F-box-like protein, 48%		-0.63
		3244	TC331345	UP|RBX1A_ARATH (Q940X7) RING-box protein 1a, 91%		-0.61
		333	TC352000			-0.39
		4822	TC369780	UP|Q6PL11_ORYSA (Q6PL11) Skp1 protein, 98%		0.68
		12912	TC324371	UP|Q2BBE2_9BACI (Q2BBE2) Drug/metabolite transporter (DMT) superfamily protein, 6%		0.74
		654	TC358997	UP|PRS7_ORYSA (Q9FXT9) 26S protease regulatory subunit 7, 61%		1.37

		991	TC358772	UP|SEM11_ARATH (Q9XIR8) Probable 26 proteasome complex subunit sem1-1, 92%		1.02
		3269	TC318099	UP|PSD6_ORYSA (Q8W425) 26S proteasome non-ATPase regulatory subunit 6		-0.78
		3410	TC322372	UP|Q6Z8F7_ORYSA (Q6Z8F7) 26S proteosome regulatory subunit-like, 38%		0.92
		6876	TC317822	UP|Q9FER4_MAIZE (Q9FER4) 20S proteasome alpha subunit		-0.46
		8181	TC333080	UP|Q9LST4_ORYSA (Q9LST4) Beta 7 subunit of 20S proteasome, 68%		0.58
		9828	TC333442	UP|PSA3_ORYSA (Q9LSU0) Proteasome subunit alpha type 3		-0.59
		10131	TC346913	UP|PSA3_ORYSA (Q9LSU0) Proteasome subunit alpha type 3		-0.81
		1943	TC370401	UP|Q2RAM6_ORYSA (Q2RAM6) Ubiquitin family		-0.59
		4422	TC354405	Zea mays clone EL01N0407D06.c mRNA sequence		-0.47
		4568	TC325536	UP|Q2A9F5_BRAOL (Q2A9F5) Ubiquitin family protein, 23%		0.76
		7240	TC317247	Zea mays clone Contig385.F mRNA sequence		0.66
		9946	TC339404	UP|SMT3_ORYSA (P55857) Ubiquitin-like protein SMT3, 93%		-0.61
		12539	TC348403	UP|Q39257_PEA (Q39257) Ubiquitin, 56%		-0.70
		622	TC340806	UP|Q9FPS3_ARATH (Q9FPS3) Ubiquitin-specific protease 24, 53%		-0.45
	Folding	5715	TC333531	UP|TBCA_ARATH (O04350) Tubulin-specific chaperone A , 75%		-0.54
		7806	TC340344	UP|Q6B4V4_VITVI (Q6B4V4) Chloroplast chaperonin 21, 82%		0.85
	Postranslational modification	982	TC359878	PRF|2206327A|1587206|2206327A T complex protein. {Cucumis sativus}		0.80
		1234	TC353892	UP|Q8GV30_ORYSA (Q8GV30) Serine/threonine protein kinase, 95%		0.85
		1607	TC322951	RF|NP_849370.1|30682312|NM_179039 MHK ATP binding {Arabidopsis thaliana}, 20%		0.76
		2306	TC336670	UP|Q5SN38_ORYSA (Q5SN38) TA9 protein-like, 25%		-0.37
		2542	TC316748	UP|Q8LF96_ARATH (Q8LF96) PRL1 protein, 78%		0.72

		2717	TC350528	RF|NP_190753.2|22331739|NM_115044 CPK13 ATP binding {Arabidopsis thaliana}, 93%		-0.36
		2931	TC320533	UP|SAPK5_ORYSA (Q7XKA8) Serine/threonine-protein kinase SAPK5 (Osmotic stress/abscisic acid-activated protein k		0.92
		3258	TC318355	UP|Q1WD14_SHEEP (Q1WD14) Polymorphic epithelial mucin, 13%		-0.85
		3456	TC340620	UP|ZB14_MAIZE (P42856) 14 kDa zinc-binding protein (Protein kinase C inhibitor) (PKCI), 98%		-0.62
		3675	TC326770	UP|Q6PS57_ORYSA (Q6PS57) Cyclin-dependent kinase subunit, 91%		-0.70
		4922	TC341559			-0.31
		6916	TC336577	UP|Q4R1K7_ORYSA (Q4R1K7) Aurora kinase, 66%		0.65
		8001	-	-		1.07
		8806	TC339077	UP|Q5Z7K2_ORYSA (Q5Z7K2) Serine/threonine protein phosphatase PP2A-1 catalytic subunit		0.69
		8875	TC338344	UP|Q7F270_ORYSA (Q7F270) ADP-ribosylation factor 1, 95%		-0.57
		10122	TC316229	UP|Q9FQF7_MAIZE (Q9FQF7) Protein kinase CK2 regulatory subunit CK2B2		-0.66
		10932	TC354655	UP|Q7G9L3_ARATH (Q7G9L3) ADP-ribosylation factor 1		0.74
		11555	TC326787	UP|O24186_ORYSA (O24186) 10 kDa chaperonin	1.05	
		11687	TC333099	RF|NP_179594.1|15225287|NM_127562 ATP binding {Arabidopsis thaliana}, 24%		-0.76
		11937	TC325980	UP|O23334_ARATH (O23334) Kinase like protein, 73%		-0.52
		2052	TC324529	RF|NP_192172.1|15235432|NM_116497 ATP binding {Arabidopsis thaliana}, 69%		1.13
	Synthesis.elongation	308	TC328020	GB|CAA50573.1|402753|GMFUSA translation elongation factor EF-G {Glycine max}, 81%		-0.68
		425	TC367625	Zea mays clone EL01N0413C10.c mRNA sequence		0.83
		540	TC367625	Zea mays clone EL01N0413C10.c mRNA sequence		0.90
		910	TC346618	Zea mays clone Contig308 mRNA sequence		0.91
		1370	TC369772	Zea mays clone Contig257 mRNA sequence		0.58

		1407	TC365231	Zea mays clone Contig598 mRNA sequence		0.74
		2049	TC336397	UP|EF1A_MAIZE (Q41803) Elongation factor 1-alpha, 45%		0.91
		2117	TC324535	Zea mays clone Contig365 mRNA sequence		0.66
		2153	TC338816	RF|NP_001031452.1|79323586|NM_001036375 GTP binding {Arabidopsis thaliana}, 28%		-0.35
		2194	TC316769	UP|Q9FYV3_SACOF (Q9FYV3) Elongation factor		0.68
		2420	TC336397	UP|EF1A_MAIZE (Q41803) Elongation factor 1-alpha, 45%		0.52
		2439	TC369304	UP|Q9SGT4_ARATH (Q9SGT4) Elongation factor EF-2, 79%		1.00
		4015	TC340493	UP|Q9M7E6_MAIZE (Q9M7E6) Elongation factor 1 alpha		0.78
		4709	TC349287	GB|AAL10483.1|15983773|AY056792 AT4g29060/F19B15_90 {Arabidopsis thaliana}, 4%		-0.54
		6169	TC337809	UP|O50018_MAIZE (O50018) Elongation factor 1-alpha		-0.58
		6838	TC332928	GB|AAB72097.1|2465428|AF021257 32 kDa protein {Hordeum vulgare subsp. vulgare}, 34%		0.73
		7391	TC367625	Zea mays clone EL01N0413C10.c mRNA sequence		0.66
		10199	TC346618	Zea mays clone Contig308 mRNA sequence		0.70
		10859	TC346618	Zea mays clone Contig308 mRNA sequence	0.85	
		12452	TC336969	UP|O50018_MAIZE (O50018) Elongation factor 1-alpha, 57%		-0.49
	Synthesis.initiation	295	TC316851	UP|IF4E2_MAIZE (O81482) Eukaryotic translation initiation factor 4E-2		-0.62
		530	TC346232	Zea mays clone Contig468 mRNA sequence		0.71
		831	TC333592	UP|SUI1_ORYSA (P33278) Protein translation factor SUI1 homolog		0.82
		833	TC320480	UP|Q2QME5_ORYSA (Q2QME5) Translation initiation factor eIF-2 gamma chain F20D22.6		0.92
		901	TC366376	UP|O24449_MAIZE (O24449) Translational initiation factor eIF-4A		1.28
		2543	TC331710	UP|IF1A_WHEAT (P47815) Eukaryotic translation initiation factor 1A		-0.53

			3240	TC343477	UP|IF33_ARATH (Q9C5Z2) Eukaryotic translation initiation factor 3 subunit 3, 96%		0.72
			3369	TC346232	Zea mays clone Contig468 mRNA sequence		0.80
			3618	TC333592	UP|SUI1_ORYSA (P33278) Protein translation factor SUI1 homolog		1.20
			3648	TC369746	RF|XP_506649.1|51963738|XM_506649 P0706E03.4-1 gene product {Oryza sativa (japonica cultivar-group)}, 13%		-0.55
			3755	TC366376	UP|O24449_MAIZE (O24449) Translational initiation factor eIF-4A		1.09
			3783	TC366625	UP|IF5_MAIZE (P55876) Eukaryotic translation initiation factor 5 (eIF-5), 98%		0.99
			4143	TC321218	UP|IF5A_MAIZE (P80639) Eukaryotic translation initiation factor 5A		-0.74
			5745	TC363663	UP|IF5A_MAIZE (P80639) Eukaryotic translation initiation factor 5A		-0.53
			6092	TC362103	UP|SUI1_MAIZE (P56330) Protein translation factor SUI1 homolog		-0.60
			6938	TC333592	UP|SUI1_ORYSA (P33278) Protein translation factor SUI1 homolog		0.64
			8065	TC320007	GB|AAM52229.1|21464549|AY120686 At1g11480/T23J18_15 {Arabidopsis thaliana}, 9%		0.75
			8731	TC354930	UP|IF5A_MAIZE (P80639) Eukaryotic translation initiation factor 5A (eIF-5A) (eIF-4D), 53%		-0.54
			9218	TC333592	UP|SUI1_ORYSA (P33278) Protein translation factor SUI1 homolog		1.18
			11347	TC321218	UP|IF5A_MAIZE (P80639) Eukaryotic translation initiation factor 5A		-0.40
			11765	TC356229	UP|O24558_MAIZE (O24558) Translation initiation factor		-0.64
			12449	TC321218	UP|IF5A_MAIZE (P80639) Eukaryotic translation initiation factor 5A		-0.53
		Synthesis.misc	10858	-	-		-0.37
			74	TC368507	UP|Q6L5M1_BROIN (Q6L5M1) Glycoprotein, 97%		-0.60
			323	TC331601	RF|XP_506724.1|51963882|XM_506724 OJ9003_G05.34 gene product {Oryza sativa (japonica cultivar-group)}		0.62
			374	TC347034	UP|RL31_PERFR (Q9M573) 60S ribosomal protein L31, 96%		0.64

426	TC337820	Zea mays clone EL01N0425H11.c mRNA sequence		0.85
436	TC326383	UP|Q8S2Y8_MAIZE (Q8S2Y8) Glycine-rich RNA binding protein		0.96
445	TC350465	UP|Q8GTE2_CICAR (Q8GTE2) Ribosomal protein RL5		0.99
473	TC330969	UP|RS11_MAIZE (P25460) 40S ribosomal protein S11		0.84
490	TC366592	UP|RL18A_ORYSA (Q943F3) 60S ribosomal protein L18a		-0.92
495	TC332929	UP|RS15D_ARATH (Q9FY64) 40S ribosomal protein S15-4, 98%		1.08
505	TC368426	UP|Q8W1C9_MAIZE (Q8W1C9) Ribosomal protein L35A		0.76
538	TC321558	UP|Q2R4A1_ORYSA (Q2R4A1) Ribosomal protein S7		1.03
554	TC326068	UP|Q9SM26_MAIZE (Q9SM26) Acidic ribosomal protein P2a-2		0.63
588	TC332529	RF|XP_507485.1|51979244|XM_507485 OJ1126_B06.24 gene product {Oryza sativa (japonica cultivar-group)}		0.44
605	TC363531	UP|Q9AV77_ORYSA (Q9AV77) 60S ribosomal protein L17		0.53
606	TC362382	UP|RS19_ORYSA (P40978) 40S ribosomal protein S19, 98%		0.85
612	TC321558	UP|Q2R4A1_ORYSA (Q2R4A1) Ribosomal protein S7		0.98
615	TC365889	UP|Q5VNV9_ORYSA (Q5VNV9) 40S subunit ribosomal protein, 80%		0.83
811	TC316836	UP|Q6RJY1_CAPAN (Q6RJY1) 60S ribosomal protein L12		0.82
827	TC341226	Zea mays clone cho1c.pk003.k1, mRNA sequence		0.98
836	TC317715	Zea mays clone cho1c.pk003.i13, mRNA sequence		0.62
841	TC347350	RF|XP_507392.1|51979030|XM_507392 B1056G08.113 gene product {Oryza sativa (japonica cultivar-group)}, 72%		1.05
843	TC321886	UP|Q9FSF6_TOBAC (Q9FSF6) Ribosomal protein L11-like, 97%		1.32
845	TC332529	RF|XP_507485.1|51979244|XM_507485 OJ1126_B06.24 gene product {Oryza sativa (japonica cultivar-group)}		0.48

		846	TC355817	UP|Q5I7K2_WHEAT (Q5I7K2) Ribosomal protein S7		0.78
		849	TC345147	UP|Q5WMY3_ORYSA (Q5WMY3) Cytoplasmic ribosomal protein L18		0.67
		856	TC324341	GB|BAA08264.1|1321661|D45423 ascorbate peroxidase {Oryza sativa}		0.49
		867	TC323766	UP|Q9FUL7_MAIZE (Q9FUL7) 40S ribosomal protein S24		0.56
		911	TC316432	RF|XP_507411.1|51979068|XM_507411 P0470D12.138 gene product {Oryza sativa (japonica cultivar-group)}		0.81
		936	TC343254			1.12
		944	TC342981	UP|RS26_ORYSA (P49216) 40S ribosomal protein S26 (S31), 91%		0.92
		989	TC339208	UP|Q5WMY3_ORYSA (Q5WMY3) Cytoplasmic ribosomal protein L18		0.56
		999	TC325882	UP|RS15_ORYSA (P31674) 40S ribosomal protein S15, 98%		0.52
		1180	TC338421	RF|XP_507607.1|51979721|XM_507607 P0562A06.14 gene product {Oryza sativa (japonica cultivar-group)}		0.87
		1281	TC343254			0.51
		1323	TC364858	UP|RS8_MAIZE (Q08069) 40S ribosomal protein S8		0.71
		1385	TC329253			0.80
		1544	TC339626	UP|Q7GD83_ARATH (Q7GD83) 40S ribosomal protein S15A		0.27
		1653	TC323771	UP|RS10_ORYSA (Q9AYP4) 40S ribosomal protein S10, 95%		-0.82
		1666	TC331279	RF|XP_506724.1|51963882|XM_506724 OJ9003_G05.34 gene product {Oryza sativa (japonica cultivar-group)}		-0.77
		1690	TC322864	UP|Q5WMY3_ORYSA (Q5WMY3) Cytoplasmic ribosomal protein L18, 98%		-0.76
		1702	TC337872	Zea mays clone Contig53 mRNA sequence		0.57
		1718	TC341804	UP|RS12_HORVU (Q9XHS0) 40S ribosomal protein S12, 91%		-0.56
		1750	TC345548	UP|Q8W1C9_MAIZE (Q8W1C9) Ribosomal protein L35A		0.89

		1754	TC347350	RF|XP_507392.1|51979030|XM_507392 B1056G08.113 gene product {Oryza sativa (japonica cultivar-group)}, 72%		0.96
		1924	TC369726	RF|XP_507485.1|51979244|XM_507485 OJ1126_B06.24 gene product {Oryza sativa (japonica cultivar-group)}		-0.58
		1945	TC343247	UP|Q6SPR2_SOYBN (Q6SPR2) Ribosomal protein L37, 98%		-0.62
		2033	TC328966	RF|XP_506724.1|51963882|XM_506724 OJ9003_G05.34 gene product {Oryza sativa (japonica cultivar-group)}		-0.62
		2058	TC338324	UP|Q2R4A1_ORYSA (Q2R4A1) Ribosomal protein S7		0.99
		2063	TC357084	UP|Q5I7K3_WHEAT (Q5I7K3) Ribosomal protein S29		0.70
		2089	TC368300	UP|H2B3_MAIZE (Q43261) Histone H2B.3		0.76
		2170	TC330209	Zea mays clone Contig1032.F mRNA sequence		-1.02
		2172	TC335017	UP|Q9AV87_ORYSA (Q9AV87) 60S ribosomal protein L21		0.99
		2304	TC342218	RF|XP_506724.1|51963882|XM_506724 OJ9003_G05.34 gene product {Oryza sativa (japonica cultivar-group)}		-0.50
		2410	TC368426	UP|Q8W1C9_MAIZE (Q8W1C9) Ribosomal protein L35A		0.81
		2435	TC328966	RF|XP_506724.1|51963882|XM_506724 OJ9003_G05.34 gene product {Oryza sativa (japonica cultivar-group)}		0.79
		2440	TC337840	UP|Q7XC31_ORYSA (Q7XC31) 60S ribosomal protein L27		0.71
		2496	TC323796	UP|RS13_MAIZE (Q05761) 40S ribosomal protein S13		0.71
		2510	TC321558	UP|Q2R4A1_ORYSA (Q2R4A1) Ribosomal protein S7		0.54
		2530	TC356993	UP|Q8L4F2_ORYSA (Q8L4F2) 40S ribosomal protein S23		-0.38
		2541	TC316306	UP|Q9FYS0_MAIZE (Q9FYS0) Ribosomal protein s6 RPS6-2		0.95
		2578	TC342916	UP|GUNA_PSEFL (P10476) Endoglucanase A precursor, 5%		0.94
		2584	TC317715	Zea mays clone cho1c.pk003.i13, mRNA sequence		0.94

2588	TC339383	UP|Q5I7K2_WHEAT (Q5I7K2) Ribosomal protein S7		1.05
2595	TC363319	UP|Q2QYC7_ORYSA (Q2QYC7) Ribosomal protein S9, 97%		0.43
2759	TC317715	Zea mays clone cho1c.pk003.i13, mRNA sequence		-0.33
2814	TC336190	UP|Q7XC31_ORYSA (Q7XC31) 60S ribosomal protein L27		0.57
2842	TC363076	UP|Q5WMY3_ORYSA (Q5WMY3) Cytoplasmic ribosomal protein L18		0.39
2845	TC350465	UP|Q8GTE2_CICAR (Q8GTE2) Ribosomal protein RL5		0.32
2853	TC325889			0.20
2875	TC342323	UP|Q8L4F2_ORYSA (Q8L4F2) 40S ribosomal protein S23		-0.31
2885	TC369791	UP|Q2QNF3_ORYSA (Q2QNF3) 60s ribosomal protein l2		0.70
2946	TC344993	GB|AAP85547.1|32493112|AY323130 ribosomal protein large subunit 13 {Oryza sativa (japonica cultivar-group)}		0.58
2965	TC321360	UP|Q9AV87_ORYSA (Q9AV87) 60S ribosomal protein L21		-0.35
3141	TC347034	UP|RL31_PERFR (Q9M573) 60S ribosomal protein L31, 96%		0.83
3143	TC338087	UP|RS141_MAIZE (P19950) 40S ribosomal protein S14 (Clone MCH1)		-0.27
3214	TC317715	Zea mays clone cho1c.pk003.i13, mRNA sequence		0.54
3222	-	-		-0.72
3236	TC355941	UP|Q5I7L3_WHEAT (Q5I7L3) Ribosomal protein L10A		0.97
3241	TC325889			0.51
3249	TC330862	RF|XP_507356.1|51978958|XM_507356 OJ1014_E09.28 gene product {Oryza sativa (japonica cultivar-group)}, 97%		0.85
3286	TC363076	UP|Q5WMY3_ORYSA (Q5WMY3) Cytoplasmic ribosomal protein L18		-0.75
3298	TC334274	UP|RS4_MAIZE (O22424) 40S ribosomal protein S4		0.41

3299	TC339525	UP|Q9FUL7_MAIZE (Q9FUL7) 40S ribosomal protein S24, 80%		0.65
3320	TC334274	UP|RS4_MAIZE (O22424) 40S ribosomal protein S4		-0.53
3351	TC329386	UP|Q8H8S1_ORYSA (Q8H8S1) Ribosomal protein L15		-0.67
3362	TC324499	UP|Q8RZ90_ORYSA (Q8RZ90) Ribosomal protein L18a-like, 91%		-0.57
3363	TC332107	UP|Q9AV77_ORYSA (Q9AV77) 60S ribosomal protein L17		0.61
3387	TC363531	UP|Q9AV77_ORYSA (Q9AV77) 60S ribosomal protein L17		0.84
3417	TC364589	Zea mays clone Contig325 mRNA sequence		0.89
3521	TC369811	UP|Q7XBH6_ORYSA (Q7XBH6) Ribosomal L9-like protein		0.91
3524	TC356815	UP|RS21_MAIZE (Q41852) 40S ribosomal protein S21		-0.51
3658	TC324117	UP|Q8RZ90_ORYSA (Q8RZ90) Ribosomal protein L18a-like, 91%		0.88
3660	TC318175	UP|Q7XY20_WHEAT (Q7XY20) Ribosomal protein L19, 89%		0.93
3692	TC337820	Zea mays clone EL01N0425H11.c mRNA sequence		0.36
3709	TC316403	RF|XP_506804.1|51964042|XM_506804 P0483C08.42 gene product {Oryza sativa (japonica cultivar-group)}		-0.60
3711	TC346347	UP|NOLA2_ARATH (Q9LEY9) H/ACA ribonucleoprotein complex subunit 2-like protein (Nhp2-like protein), 77%		0.45
3785	TC351143	UP|Q9MAW5_PANGI (Q9MAW5) Ribosomal protein L29, 98%		0.70
3901	TC369629	UP|Q5I7L1_WHEAT (Q5I7L1) Ribosomal protein L13a		-0.83
4023	TC325978	UP|Q9AV87_ORYSA (Q9AV87) 60S ribosomal protein L21		0.77
4033	-	-		0.93
4060	TC326208	UP|Q7XBH6_ORYSA (Q7XBH6) Ribosomal L9-like protein		0.73
4063	TC316271	UP|O22453_MAIZE (O22453) Ribsomal protein S4		-0.36

		4071	TC345548	UP|Q8W1C9_MAIZE (Q8W1C9) Ribosomal protein L35A		0.56
		4077	TC334532	UP|Q7XC31_ORYSA (Q7XC31) 60S ribosomal protein L27		-0.48
		4105	TC342802	GB|AAX96401.1|62734292|AC133931 Ribosomal L38e protein family {Oryza sativa (japonica cultivar-group)}		-0.47
		4123	TC330969	UP|RS11_MAIZE (P25460) 40S ribosomal protein S11		0.82
		4130	TC369629	UP|Q5I7L1_WHEAT (Q5I7L1) Ribosomal protein L13a		0.83
		4182	TC334274	UP|RS4_MAIZE (O22424) 40S ribosomal protein S4		1.06
		4456	TC369629	UP|Q5I7L1_WHEAT (Q5I7L1) Ribosomal protein L13a		0.88
		4484	TC316836	UP|Q6RJY1_CAPAN (Q6RJY1) 60S ribosomal protein L12		0.74
		4513	TC339626	UP|Q7GD83_ARATH (Q7GD83) 40S ribosomal protein S15A		0.45
		4532	TC323766	UP|Q9FUL7_MAIZE (Q9FUL7) 40S ribosomal protein S24		0.85
		4580	TC318175	UP|Q7XY20_WHEAT (Q7XY20) Ribosomal protein L19, 89%		0.66
		4663	TC328181	UP|Q84XZ0_9LAMI (Q84XZ0) 60S ribosomal protein L34, 65%		-0.56
		4758	TC338330	UP|Q5GMM4_CAPCH (Q5GMM4) 60S ribosomal protein L37a		-0.62
		4802	TC322963	GB|BAA02155.1|303853|RICRPL3A ribosomal protein L3 {Oryza sativa (japonica cultivar-group)}		0.85
		4808	TC346815	GB|BAA02155.1|303853|RICRPL3A ribosomal protein L3 {Oryza sativa (japonica cultivar-group)}		1.43
		4850	TC325255	UP|Q2R1J8_ORYSA (Q2R1J8) Ribosomal protein S4, 98%		0.86
		4854	TC330969	UP|RS11_MAIZE (P25460) 40S ribosomal protein S11		0.83
		4856	TC348584	UP|Q6LCT7_MAIZE (Q6LCT7) Ubiquitin fusion protein		1.37
		4870	TC368300	UP|H2B3_MAIZE (Q43261) Histone H2B.3		0.66
		4893	TC324315	UP|RS19_ORYSA (P40978) 40S ribosomal protein S19, 98%		0.77
		4906	TC366722	Zea mays clone Contig205 mRNA sequence		0.43

4932	TC369245	UP|Q6UEJ2_PEA (Q6UEJ2) Mini-chromosome maintenance 7, 23%		0.84
4948	TC360835	UP|RL41_ARATH (P62120) 60S ribosomal protein L41		0.83
4953	TC345444	RF|XP_507485.1|51979244|XM_507485 OJ1126_B06.24 gene product {Oryza sativa (japonica cultivar-group)}		0.98
4969	TC325978	UP|Q9AV87_ORYSA (Q9AV87) 60S ribosomal protein L21		0.71
4986	TC325889			1.79
4992	TC369722	UP|RL41_ARATH (P62120) 60S ribosomal protein L41		1.04
5153	TC325255	UP|Q2R1J8_ORYSA (Q2R1J8) Ribosomal protein S4, 98%		1.55
5345	TC346815	GB|BAA02155.1|303853|RICRPL3A ribosomal protein L3 {Oryza sativa (japonica cultivar-group)}		0.82
5542	TC333590	UP|Q3MST7_ORYSA (Q3MST7) Ribosomal L32		1.04
5557	TC327833	UP|RS142_MAIZE (P19951) 40S ribosomal protein S14 (Clone MCH2)		-0.43
5654	TC334031	UP|RS12_HORVU (Q9XHS0) 40S ribosomal protein S12, 91%		1.07
5735	TC342104	UP|Q6K955_ORYSA (Q6K955) 60S ribosomal protein-like, 19%		-0.78
5754	TC366722	Zea mays clone Contig205 mRNA sequence		-0.55
6172	TC342981	UP|RS26_ORYSA (P49216) 40S ribosomal protein S26 (S31), 91%		-0.58
6357	TC331934	UP|Q3MST7_ORYSA (Q3MST7) Ribosomal L32		-0.58
6409	TC349051	UP|RLA2A_MAIZE (P46252) 60S acidic ribosomal protein P2A (P2)		0.57
6411	TC316822	UP|Q9FJA6_ARATH (Q9FJA6) 40S ribosomal protein S3 (AT5g35530/MOK9_14), 86%		0.90
6425	TC328966	RF|XP_506724.1|51963882|XM_506724 OJ9003_G05.34 gene product {Oryza sativa (japonica cultivar-group)}		0.97
6463	TC325255	UP|Q2R1J8_ORYSA (Q2R1J8) Ribosomal protein S4, 98%		0.88
6500	TC332529	RF|XP_507485.1|51979244|XM_507485 OJ1126_B06.24 gene product {Oryza sativa (japonica cultivar-group)}		1.10

6528	TC330969	UP|RS11_MAIZE (P25460) 40S ribosomal protein S11		1.26
6545	TC350175	UP|Q20BM6_PANGI (Q20BM6) Ribosomal protein L31, 90%		0.81
6575	TC326729	UP|RL111_ARATH (P42795) 60S ribosomal protein L11-1 (L16A), 98%		0.61
6598	TC338085	UP|Q7XR19_ORYSA (Q7XR19) 60S ribosomal protein L6		0.61
6614	TC361827	UP|RL10_MAIZE (P45633) 60S ribosomal protein L10		0.77
6743	TC348142	RF|XP_507485.1|51979244|XM_507485 OJ1126_B06.24 gene product {Oryza sativa (japonica cultivar-group)}		-0.71
6779	TC366670	UP|Q7XEQ3_ORYSA (Q7XEQ3) Ribosomal S17		-0.32
6901	TC321558	UP|Q2R4A1_ORYSA (Q2R4A1) Ribosomal protein S7		0.78
6904	TC324399	UP|Q9FJA6_ARATH (Q9FJA6) 40S ribosomal protein S3 (AT5g35530/MOK9_14), 88%		1.01
7017	-	-		0.64
7097	TC359038	UP|Q6RH11_CAPAN (Q6RH11) 40S ribosomal protein S5, 41%		-0.66
7234	TC343267	UP|Q20BM6_PANGI (Q20BM6) Ribosomal protein L31, 90%		0.50
7267	TC334031	UP|RS12_HORVU (Q9XHS0) 40S ribosomal protein S12, 91%		0.85
7319	TC341839	UP|Q5U7K1_9POAL (Q5U7K1) S27 ribosomal protein		-0.31
7334	TC338957	UP|RS15_ORYSA (P31674) 40S ribosomal protein S15		1.01
7351	TC324946	UP|RL7A_ORYSA (P35685) 60S ribosomal protein L7a		-0.84
7611	TC369726	RF|XP_507485.1|51979244|XM_507485 OJ1126_B06.24 gene product {Oryza sativa (japonica cultivar-group)}		0.98
7658	TC363531	UP|Q9AV77_ORYSA (Q9AV77) 60S ribosomal protein L17		0.61
7733	-	-		-0.33
7744	TC355817	UP|Q5I7K2_WHEAT (Q5I7K2) Ribosomal protein S7	0.66	

7813	TC369424	UP|Q2PYZ0_SOLTU (Q2PYZ0) 40S ribosomal protein-like protein, 46%		-0.44
8011	TC342119	UP|Q8GTE2_CICAR (Q8GTE2) Ribosomal protein RL5		0.69
8020	TC348584	UP|Q6LCT7_MAIZE (Q6LCT7) Ubiquitin fusion protein		1.03
8182	TC350586	UP|Q7X9K4_WHEAT (Q7X9K4) S28 ribosomal protein, 78%		0.87
8404	TC316271	UP|O22453_MAIZE (O22453) Ribsomal protein S4		0.82
8512	TC350465	UP|Q8GTE2_CICAR (Q8GTE2) Ribosomal protein RL5		-0.15
8518	TC329395	GB|AAP85547.1|32493112|AY323130 ribosomal protein large subunit 13 {Oryza sativa (japonica cultivar-group)}		-0.65
8528	TC328924	UP|O82579_MAIZE (O82579) Ribosomal protein L26, 98%		1.34
8876	TC339071	RF|XP_507356.1|51978958|XM_507356 OJ1014_E09.28 gene product {Oryza sativa (japonica cultivar-group)}		-0.32
8886	TC333590	UP|Q3MST7_ORYSA (Q3MST7) Ribosomal L32		1.19
9201	TC362382	UP|RS19_ORYSA (P40978) 40S ribosomal protein S19, 98%		0.70
9288	TC348304	UP|RS141_MAIZE (P19950) 40S ribosomal protein S14 (Clone MCH1), 60%		0.59
9606	TC331803	Zea mays clone E04912707E08.c mRNA sequence		0.73
9628	TC325889			0.65
9652	TC325889			1.07
9657	-	-		0.55
9683	TC326476	UP|Q94AF6_ARATH (Q94AF6) AT5g20160/F5O24_50 (Ribosomal protein L7Ae-like), 97%		-0.51
9760	-	-		1.26
9805	TC355817	UP|Q5I7K2_WHEAT (Q5I7K2) Ribosomal protein S7	1.01	
9942	TC371087	UP|Q5GMM4_CAPCH (Q5GMM4) 60S ribosomal protein L37a		-0.44

		10054	TC319111	RF|XP_507356.1|51978958|XM_507356 OJ1014_E09.28 gene product {Oryza sativa (japonica cultivar-group)}, 98%		-0.32
		10065	-	-		-0.53
		10088	TC323112	UP|RL7A_ORYSA (P35685) 60S ribosomal protein L7a		1.10
		10129	TC363614	RF|XP_507392.1|51979030|XM_507392 B1056G08.113 gene product {Oryza sativa (japonica cultivar-group)}, 21%		-0.56
		10516	TC330969	UP|RS11_MAIZE (P25460) 40S ribosomal protein S11		-0.52
		10539	TC347888	RF|NP_916542.1|34910390|NM_191653 ribosomal protein L28-like {Oryza sativa (japonica cultivar-group)}, 95%		0.71
		10806	TC322766	UP|Q5WMY3_ORYSA (Q5WMY3) Cytoplasmic ribosomal protein L18		-0.49
		10863	TC337840	UP|Q7XC31_ORYSA (Q7XC31) 60S ribosomal protein L27		0.49
		11203	TC330576	UP|Q5I7L3_WHEAT (Q5I7L3) Ribosomal protein L10A		0.60
		11299	TC316427	UP|Q5GWV4_XANOR (Q5GWV4) 50S ribosomal protein L15, 49%	1.28	1.11
		11348	TC357012	UP|P93626_MAIZE (P93626) 40S ribosomal subunit protein S21		-0.45
		11539	TC359412	UP|Q2QYC7_ORYSA (Q2QYC7) Ribosomal protein S9, 97%		-0.45
		11557	TC363721	UP|Q7XR19_ORYSA (Q7XR19) 60S ribosomal protein L6		-0.48
		11688	TC336615	UP|Q9FJA6_ARATH (Q9FJA6) 40S ribosomal protein S3 (AT5g35530/MOK9_14), 86%		-0.77
		11732	-	-		-0.34
		11818	TC363076	UP|Q5WMY3_ORYSA (Q5WMY3) Cytoplasmic ribosomal protein L18		-0.41
		11973	TC371087	UP|Q5GMM4_CAPCH (Q5GMM4) 60S ribosomal protein L37a		-0.60
		13075	TC324315	UP|RS19_ORYSA (P40978) 40S ribosomal protein S19, 98%		0.93
		4421	TC354770	UP|BXDC1_ORYSA (Q9AWM9) Brix domain-containing protein 1 homolog, 97%		-0.29
		4634	TC335422	UP|O82589_ARATH (O82589) F11O4.6, 8%		-0.66

	Synthesis.mito/plastid ribosomal protein.mitochondrial	1973	TC324442	UP|Q9XGC7_MAIZE (Q9XGC7) Iron sulfur subunit of succinate dehydrogenase (Truncated) and ribosomal protein S14 pr		0.61
		691	TC316935	UP|RR1_SPIOL (P29344) 30S ribosomal protein S1, chloroplast precursor (CS1), 83%		0.57
		8161	TC358817	RF|NP_172011.1|15220443|NM_100397 EMB2394 structural constituent of ribosome {Arabidopsis thaliana}, 78%		0.89
		11293	TC330042	UP|RRP3_HORVU (O48609) Plastid-specific 30S ribosomal protein 3, chloroplast precursor (PSRP-3), 77%		-0.52
		243	TC333514	Zea mays clone EL01N0444C11.c mRNA sequence		-0.43
		3040	TC333514	Zea mays clone EL01N0444C11.c mRNA sequence		-0.47
		4645	TC332850	GB|AAP31927.1|30387523|BT006583 At3g49080 {Arabidopsis thaliana}, 47%		-0.49
		5920	TC346050	UP|RK3_TOBAC (O80360) 50S ribosomal protein L3, chloroplast precursor, 84%		-0.82
	Synthesis.release	4230	TC336309	RF|XP_469434.1|50918075|XM_469434 eukaryotic peptide chain release factor subunit 1-3 (eRF1-3) {Oryza sativa (ja		-0.76
	Targeting	9472	TC319178	UP|Q84PB7_ORYSA (Q84PB7) Inositol phosphatase-like protein		-1.01
		1328	TC330375	UP|Q7XI32_ORYSA (Q7XI32) Small zinc finger-like protein, 80%		0.85
		5952	TC350060	UP|Q9XGY3_MALDO (Q9XGY3) Small zinc finger-like protein, 87%		-0.90
		11400	TC331116	UP|Q6NKU9_ARATH (Q6NKU9) At5g55510, 37%		0.78
		5264	TC326154	UP|Q2HVW5_MEDTR (Q2HVW5) Nucleoporin interacting component Protein prenyltransferase, 23%		-0.84
		10257	TC344686	UP|Q32Y71_ORYSA (Q32Y71) Peroxisomal targeting signal 1 receptor long form, 48%		-0.65
		788	TC328393	UP|Q9MAY9_MAIZE (Q9MAY9) Nonclathrin coat protein zeta1-COP		0.89
		2001	TC326586	UP|Q6UCJ1_CUCSA (Q6UCJ1) Signal recognition particle receptor protein, 66%		0.61
		3589	TC344732	UP|Q5FYS3_DAUCA (Q5FYS3) ADP-ribosylation factor, 96%		0.77
		10252	TC360480	UP|Q5JJV1_ORYSA (Q5JJV1) Vacuolar protein sorting 55 family-like	1.10	

Photosynthesis	Calvin cyle	2590	TC318980	UP|ALF_MAIZE (P08440) Fructose-bisphosphate aldolase, cytoplasmic isozyme		-0.85
		11904	TC349449	UP|Q9FUI0_MAIZE (Q9FUI0) Ribulose 1, 5-bisphosphate carboxylase/oxygenase activase, 47%		-0.31
	Lightreaction	5796	TC334243	RF|XP_507256.1|51964944|XM_507256 P0493A04.32 gene product {Oryza sativa (japonica cultivar-group)}, 77%		-0.56
		15095	TC324016	UP|PSBO_WHEAT (P27665) Oxygen-evolving enhancer protein 1, chloroplast precursor , 98%		-0.36
		15455	TC324016	UP|PSBO_WHEAT (P27665) Oxygen-evolving enhancer protein 1, chloroplast precursor , 98%		-0.43
		3712	TC324529	RF|NP_192172.1|15235432|NM_116497 ATP binding {Arabidopsis thaliana}, 69%		1.04
	Photorespiration	3433	TC338593	UP|Q69J93_ORYSA (Q69J93) Phosphoglycerate dehydrogenase-like protein, 32%		0.54
Redox	Ascorbate and glutathione	2424	-	-		-0.57
		3724	TC336263	GB|BAA08264.1|1321661|D45423 ascorbate peroxidase {Oryza sativa}		-0.16
		3883	TC324341	GB|BAA08264.1|1321661|D45423 ascorbate peroxidase {Oryza sativa}		-0.69
		5570	TC356200	GB|BAA08264.1|1321661|D45423 ascorbate peroxidase {Oryza sativa}, 97%		0.79
		6423	TC324341	GB|BAA08264.1|1321661|D45423 ascorbate peroxidase {Oryza sativa}		-0.49
		8527	TC324341	GB|BAA08264.1|1321661|D45423 ascorbate peroxidase {Oryza sativa}		1.01
		12160	TC325195	UP|Q65XA0_ORYSA (Q65XA0) Dehydroascorbate reductase		-0.30
	Dismutases and catalases	416	TC325282	UP|Q94L33_ARATH (Q94L33) Ania-6a type cyclin, 15%		0.80
		1821	TC331770	GB|AAA33511.1|168622|MZESOD2A SOD2 protein {Zea mays}		-1.00
		4753	TC327155	UP|SODC2_MESCR (O49044) Superoxide dismutase [Cu-Zn] 2, 84%		-0.72
		8096	TC316288	UP|SODM1_MAIZE (P09233) Superoxide dismutase [Mn] 3.1, mitochondrial precursor	0.92	
	Glutaredoxins	10428	TC326201	UP|Q84Z96_ORYSA (Q84Z96) Glutaredoxin protein family-like, 72%		0.75
	Misc	9900	TC345232	UP|NCB5R_ARATH (P83291) NADH-cytochrome b5 reductase-like protein (B5R) , 78%		-0.34
	Thioredoxin	1775	TC349862	UP|Q5EUE1_MAIZE (Q5EUE1) Protein disulfide isomerase		1.33

		3592	TC350147	UP|Q5EUC2_MAIZE (Q5EUC2) Adenosine 5'-phosphosulfate reductase 8, 31%		1.19
		5001	TC323131	UP|Q5EUD1_MAIZE (Q5EUD1) Protein disulfide isomerase		-0.59
		6530	TC325410	UP|Q4W1F6_MAIZE (Q4W1F6) Thioredoxin h2 protein		0.75
		7022	TC325410	UP|Q4W1F6_MAIZE (Q4W1F6) Thioredoxin h2 protein	0.85	
		7892	TC347484	UP|Q30BI3_MAIZE (Q30BI3) NADPH-dependent reductase		-0.80
		8324	TC325364	UP|Q4ACU9_9ASTR (Q4ACU9) Thioredoxin h, 80%		-0.54
		10355	TC316531	UP|Q5EUD0_MAIZE (Q5EUD0) Protein disulfide isomerase		-0.44
		11477	TC354505	UP|Q6N0X2_RHOPA (Q6N0X2) Methyl-accepting chemotaxis receptor/sensory transducer precursor, 3%		-0.51
		12448	TC335905	UP|Q4W1F7_MAIZE (Q4W1F7) Thioredoxin h1 protein		-0.63
RNA	Processing	941	TC365708	UP|Q9M6E6_TOBAC (Q9M6E6) Poly(A)-binding protein, 84%		1.04
		1637	TC329637	UP|Q9FJN9_ARATH (Q9FJN9) Poly(A)-binding protein II-like, 80%		0.70
		2580	TC353016	Zea mays clone EL01N0365E12.c mRNA sequence	0.81	0.33
		2923	TC327650	UP|Q3LHL4_SOLTU (Q3LHL4) Pre-mRNA splicing factor, 57%		0.40
		4637	TC326099	Zea mays clone EK07D2303C05.c mRNA sequence		-0.34
		4915	TC317148	UP|Q8L5U6_ARATH (Q8L5U6) SnRNP core Sm protein Sm-X5-like protein		1.14
		4934	TC318918	UP|LSM4_ORYSA (Q9LGE6) Probable U6 snRNA-associated Sm-like protein LSm4		0.78
		5021	TC328932	UP|Q9SHY4_ARATH (Q9SHY4) F1E22.8, 14%		0.64
		5521	TC343274	UP|RUXG_ARATH (O82221) Probable small nuclear ribonucleoprotein G, 95%		0.51
		6161	TC359282	UP|Q6ENK8_ORYSA (Q6ENK8) Splicing factor 4-like protein, 66%		-0.62
		6445	TC329637	UP|Q9FJN9_ARATH (Q9FJN9) Poly(A)-binding protein II-like, 80%		0.94
		7327	TC320074	UP|Q9FJN9_ARATH (Q9FJN9) Poly(A)-binding protein II-like, 71%		0.53

		7704	TC316492	RF|NP_567250.1|18412147|NM_116546 nucleic acid binding {Arabidopsis thaliana}, 34%		-0.68
		8422	TC334832	UP|Q9FKB0_ARATH (Q9FKB0) Sm-like protein, 95%		0.52
		8852	TC353016	Zea mays clone EL01N0365E12.c mRNA sequence		0.43
		9721	TC353016	Zea mays clone EL01N0365E12.c mRNA sequence		-0.68
		10040	TC340391	RF|XP_507429.1|51979110|XM_507429 B1370C05.32 gene product {Oryza sativa (japonica cultivar-group)}		-0.38
		10873	TC321989	UP|Q8L3W7_ARATH (Q8L3W7) Small nuclear ribonucleoprotein-like protein, 93%		-0.43
		12074	TC353695	RF|NP_172528.2|30681779|NM_100933 nucleotide binding {Arabidopsis thaliana}, 23%		-0.42
		249	TC336601	UP|Q6H874_ORYSA (Q6H874) DEAD/DEAH box helicase-like, 51%		0.65
		279	TC340789	UP|Q6Z8Q6_ORYSA (Q6Z8Q6) Splicing factor 3B subunit 5-like protein		-0.60
		462	TC316321	Zea mays clone Contig631.F mRNA sequence		-0.43
		801	TC337634	UP|Q75KQ4_ORYSA (Q75KQ4) Expressed protein, 93%		1.14
		1004	TC317909	UP|Q2QKB4_WHEAT (Q2QKB4) U2AF large subunit, 95%		0.82
		1671	TC319273	UP|Q6AUG0_ORYSA (Q6AUG0) 'U2 snRNP auxiliary factor, small subunit', 88%		-0.84
		3202	TC339403	UP|Q69K06_ORYSA (Q69K06) Pre-mRNA splicing factor PRP38 protein-like, 97%		0.93
		3329	TC327822	RF|NP_178124.1|15220049|NM_106656 SUS2 (ABNORMAL SUSPENSOR 2) {Arabidopsis thaliana}, 24%	1.46	1.55
		6073	TC366189	UP|Q64HC3_MAIZE (Q64HC3) ASF/SF2-like pre-mRNA splicing factor SRP32		-0.70
		6826	TC319273	UP|Q6AUG0_ORYSA (Q6AUG0) 'U2 snRNP auxiliary factor, small subunit', 88%		0.50
		9613	TC339403	UP|Q69K06_ORYSA (Q69K06) Pre-mRNA splicing factor PRP38 protein-like, 97%		0.92
		12104	TC318737	Zea mays clone EL01N0563B09.c mRNA sequence		-0.97
		12785	TC325146	UP|Q2QKC2_WHEAT (Q2QKC2) Pre-mRNA processing factor, 97%		-1.13

	Regulation of transcription	4812	TC334991	UP|Q5N9F3_ORYSA (Q5N9F3) BolA-like protein, 88%		0.40
		599	TC319612	UP|O49216_ORYSA (O49216) Nucleic acid binding protein, 79%		1.13
		2230	TC319612	UP|O49216_ORYSA (O49216) Nucleic acid binding protein, 79%		1.10
		9299	TC325726	Zea mays clone EL01N0414F09.d mRNA sequence		-0.38
		375	TC338349	UP|Q9SWV4_LYCES (Q9SWV4) ER66 protein, 27%		-0.62
		3289	TC360833	UP|Q5MP56_MAIZE (Q5MP56) Barren stalk1		0.58
		1261	TC316584	UP|OCS1_MAIZE (P24068) Ocs element-binding factor 1 (OCSBF-1)		0.52
		9755	TC339782	UP|Q5JNB8_ORYSA (Q5JNB8) Zinc finger protein-like, 56%		0.90
		8314	TC325829	UP|Q2QM17_ORYSA (Q2QM17) YABBY protein, 59%		1.05
		1731	TC340158	GB|AAO73339.1|30103103|AY186610 transcription factor IIIA {Arabidopsis thaliana}, 27%		1.05
		2225	TC360277	UP|Q653D5_ORYSA (Q653D5) Zinc finger protein-like, 37%		0.49
		5652	TC359677	UP|Q5Z9H7_ORYSA (Q5Z9H7) Zinc finger protein-like, 89%		0.69
		10501	TC333848	UP|Q8LEE4_ARATH (Q8LEE4) Zinc finger protein, 37%		-0.47
		1016	TC328863	UP|Q69XQ3_ORYSA (Q69XQ3) KH domain-containing protein / zinc finger protein-like, 30%		0.74
		2935	TC328863	UP|Q69XQ3_ORYSA (Q69XQ3) KH domain-containing protein / zinc finger protein-like, 30%		0.59
		12816	TC363395	Zea mays clone EL01T0201H08.c mRNA sequence		-0.71
		4944	TC331266	UP|Q8W0W6_MAIZE (Q8W0W6) Repressor protein, 78%		0.42
		5657	TC334623	UP|Q9AXT8_MAIZE (Q9AXT8) DNA cytosine methyltransferase MET2a		0.82
		1625	TC316584	UP|OCS1_MAIZE (P24068) Ocs element-binding factor 1 (OCSBF-1)		-0.68
		11856	TC337991	UP|Q84NK2_ORYSA (Q84NK2) Myb family transcription factor-like, 21%		0.95
		3237	-	-		0.80

		4153	TC355856	UP|Q6AWY1_ORYSA (Q6AWY1) Growth-regulating factor 8, 46%		-0.32
		1631	TC327398	Zea mays clone EL01N0409A08.d mRNA sequence		-0.88
		5460	-	-		-0.69
		8602	TC340276	UP|Q1WD28_PSEMZ (Q1WD28) Class III homeodomain-leucine zipper, 14%		-0.34
		12660	TC315884	UP|Q4R0U0_MAIZE (Q4R0U0) Homeobox protein OCL1	-1.10	
		14861	TC325312	Zea mays clone EL01N0421G04.c mRNA sequence		0.53
		12174	TC331433	UP|MAD20_ORYSA (Q2QQA3) MADS-box trancription factor 20 (OsMADS20), 60%		0.91
		10448	TC358883	UP|Q8LK06_MAIZE (Q8LK06) Methyl binding domain protein MBD109, 83%	1.63	1.03
		10576	TC358883	UP|Q8LK06_MAIZE (Q8LK06) Methyl binding domain protein MBD109, 83%		1.22
		12704	TC346568	UP|Q9AVV5_HORVU (Q9AVV5) MCB2 protein, 33%		-0.55
		72	TC343112	UP|Q8W512_MAIZE (Q8W512) HMG-like nucleosome/chromatin assembly factor D		-0.53
		3243	TC364640	UP|MNB1B_MAIZE (P27347) DNA-binding protein MNB1B		-0.68
		3253	TC364640	UP|MNB1B_MAIZE (P27347) DNA-binding protein MNB1B		-0.43
		3742	TC364640	UP|MNB1B_MAIZE (P27347) DNA-binding protein MNB1B		-0.48
		4042	TC364640	UP|MNB1B_MAIZE (P27347) DNA-binding protein MNB1B		0.70
		4186	TC364640	UP|MNB1B_MAIZE (P27347) DNA-binding protein MNB1B		0.45
		4972	TC364640	UP|MNB1B_MAIZE (P27347) DNA-binding protein MNB1B		1.19
		5792	TC365371	UP|Q8W510_MAIZE (Q8W510) HMG type nucleosome/chromatin assembly factor D		-0.58
		6060	TC364640	UP|MNB1B_MAIZE (P27347) DNA-binding protein MNB1B		-0.57
		6180	TC364640	UP|MNB1B_MAIZE (P27347) DNA-binding protein MNB1B		-0.63
		6442	TC364640	UP|MNB1B_MAIZE (P27347) DNA-binding protein MNB1B		0.58

		7291	TC364640	UP|MNB1B_MAIZE (P27347) DNA-binding protein MNB1B		1.11
		7384	TC364640	UP|MNB1B_MAIZE (P27347) DNA-binding protein MNB1B		0.69
		8929	TC364640	UP|MNB1B_MAIZE (P27347) DNA-binding protein MNB1B		0.61
		9746	TC364640	UP|MNB1B_MAIZE (P27347) DNA-binding protein MNB1B		-0.60
		9774	TC364640	UP|MNB1B_MAIZE (P27347) DNA-binding protein MNB1B		-0.67
		10458	TC364640	UP|MNB1B_MAIZE (P27347) DNA-binding protein MNB1B		0.66
		11017	TC364640	UP|MNB1B_MAIZE (P27347) DNA-binding protein MNB1B		0.68
		11306	TC365371	UP|Q8W510_MAIZE (Q8W510) HMG type nucleosome/chromatin assembly factor D		-0.48
		11380	TC364640	UP|MNB1B_MAIZE (P27347) DNA-binding protein MNB1B		-0.69
		11691	TC364640	UP|MNB1B_MAIZE (P27347) DNA-binding protein MNB1B		-0.35
		11747	TC364640	UP|MNB1B_MAIZE (P27347) DNA-binding protein MNB1B		-0.57
		12524	TC364640	UP|MNB1B_MAIZE (P27347) DNA-binding protein MNB1B		-0.84
		4950	TC316182	UP|Q84QG8_MAIZE (Q84QG8) Floricaula/leafy-like 1		0.60
		2531	TC372391	UP|Q66GR6_ARATH (Q66GR6) At2g02740, 58%		0.38
		895	TC317727	UP|Q8VZJ4_ARATH (Q8VZJ4) AT4g25730/F14M19_10, 30%		0.59
		1158	TC338706	UP|Q6DQ93_MUSAC (Q6DQ93) BTF3b-like transcription factor, 98%		0.46
		1979	TC352142	UP|Q9SAQ3_MAIZE (Q9SAQ3) Proliferating cell nuclear antigen		-0.44
		2647	TC342812	UP|Q7CVD9_AGRT5 (Q7CVD9) AGR_L_543p, 4%		-0.38
		2956	TC328113			0.99
		6095	TC332546	Zea mays clone Contig290 mRNA sequence		-0.61
		6606	-	-		0.98

6853	TC336541	Zea mays clone EL01N0447D03.c mRNA sequence		0.97
7403	TC354762	UP|Q9M7F3_MAIZE (Q9M7F3) LIM transcription factor homolog, 52%	1.17	
7689	TC345401			-0.81
8220	TC345401			0.74
8996	TC357075	UP|Q9M7F3_MAIZE (Q9M7F3) LIM transcription factor homolog		-0.29
11353	TC316738	UP|Q9SAQ3_MAIZE (Q9SAQ3) Proliferating cell nuclear antigen		-0.76
12472	TC341077	UP|Q6RF30_MAIZE (Q6RF30) Rolled leaf1		-0.42
3503	TC331478	GB|AAF27058.1|6730637|AC008262 F4N2.13 {Arabidopsis thaliana}, 20%		-0.48
4003	-	-		0.63
6517	TC334689	UP|Q9LS09_ARATH (Q9LS09) Anti-silencing protein-like, 72%		0.67
12450	TC319914	UP|Q9LS09_ARATH (Q9LS09) Anti-silencing protein-like, 72%		-0.76
3689	TC316736	Zea mays clone Contig572.F mRNA sequence		1.00
4959	TC326255	RF|XP_506643.1|51963726|XM_506643 B1008E06.13 gene product {Oryza sativa (japonica cultivar-group)}		0.44
8135	TC372266	UP|Q84LS4_MAIZE (Q84LS4) Superal1		-0.38
380	TC334418	UP|PFD5_ARATH (P57742) Probable prefoldin subunit 5, 83%		-0.38
2423	TC328589	UP|Q2IMJ3_ANADE (Q2IMJ3) LigA, 5%		-0.69
2795	TC331891	UP|Q69NK8_ORYSA (Q69NK8) CwfJ / zinc finger(CCCH-type)-like protein, 33%		-0.28
3370	TC343233	RF|XP_506746.1|51963926|XM_506746 OJ1225_F07.15 gene product {Oryza sativa (japonica cultivar-group)}		0.91
3527	TC332569	RF|NP_196819.1|15240035|NM_121318 protein binding {Arabidopsis thaliana}, 27%		-0.50
3644	TC316995	Zea mays clone EL01N0320H11.d mRNA sequence		-0.55

		4140	TC326167	UP|Q3BCU2_MAIZE (Q3BCU2) Zinc finger protein		1.01
		5033	TC365511	Zea mays clone Contig483.F mRNA sequence		0.36
		5612	TC350631	RF|XP_506746.1|51963926|XM_506746 OJ1225_F07.15 gene product {Oryza sativa (japonica cultivar-group)}, 94%		-0.51
		5931	TC349234	RF|NP_196819.1|15240035|NM_121318 protein binding {Arabidopsis thaliana}, 21%		-0.51
		6202	TC329076	UP|Q402F0_LYCES (Q402F0) Tobamovirus multiplication 1 homolog 3, 57%		-0.62
		7691	TC331702	GB|BAB02292.1|11994333|AB017071 WD-40 repeat protein-like {Arabidopsis thaliana}, 36%		-0.89
		7772	TC357222	UP|Q6Z4J7_ORYSA (Q6Z4J7) LAs17 Binding protein-like, 14%		-0.76
		8373	TC331622	UP|Q5Z411_ORYSA (Q5Z411) PHD zinc finger protein-like, 25%		-0.47
		9672	TC324152	UP|Q5NAR8_ORYSA (Q5NAR8) Purine rich element binding protein B-like, 75%		0.58
		10322	TC329794	UP|Q8L8V0_ARATH (Q8L8V0) Transcription co-activator-like protein, 92%		-0.57
		12039	TC320216	GB|AAP37853.1|30725662|BT008494 At1g11650 {Arabidopsis thaliana}, 45%		1.05
		117	TC358046	UP|Q32SG4_MAIZE (Q32SG4) WRKY1		0.99
		2061	TC329118	UP|Q7XAA4_ORYSA (Q7XAA4) WRKY12, 26%		0.52
		7269	TC358046	UP|Q32SG4_MAIZE (Q32SG4) WRKY1		1.35
		8420	TC358046	UP|Q32SG4_MAIZE (Q32SG4) WRKY1		1.23
	RNA binding	110	TC368465	Zea mays clone Contig980.F mRNA sequence		0.70
		506	TC356293	UP|Q7XI13_ORYSA (Q7XI13) Glycine-rich RNA-binding protein-like, 47%		1.00
		548	TC368465	Zea mays clone Contig980.F mRNA sequence		0.83
		1705	TC366125	Zea mays clone Contig114 mRNA sequence		-0.87
		2409	TC318428	Zea mays clone EL01N0360D09.c mRNA sequence		0.95
		2447	TC348473	UP|PM14_ARATH (Q9FMP4) Pre-mRNA branch site p14-like protein, 91%		-0.55

		2502	TC343996	UP|Q651Z0_ORYSA (Q651Z0) RNA-binding protein-like, 42%		0.89
		3242	TC340985	UP|Q42412_NICSY (Q42412) RNA-binding protein RZ-1, 69%	1.14	
		3419	TC359617	GB|CAA80307.1|296548|MMFIBRLNA fibrillarin {Mus musculus}, 13%		1.30
		3655	TC321193	Zea mays clone EL01N0450E05.d mRNA sequence		0.51
		4528	TC347148	UP|Q6K950_ORYSA (Q6K950) Nucleolar RNA-binding Nop10p-like protein, 26%		0.87
		5958	TC340705	RF|NP_564915.1|18408904|NM_105465 nucleic acid binding {Arabidopsis thaliana}, 19%		-0.56
		6772	TC320562	Zea mays clone EL01N0424C12.d mRNA sequence		-0.46
		6843	TC363572	UP|Q5ZDX8_ORYSA (Q5ZDX8) Heterogeneous nuclear ribonucleoprotein A2/B1-like, 83%	1.22	0.82
		7352	TC318428	Zea mays clone EL01N0360D09.c mRNA sequence		0.85
		10120	TC368465	Zea mays clone Contig980.F mRNA sequence		0.94
	Transcription	1427	TC324721	UP|Q53LH8_ORYSA (Q53LH8) Dna-directed rna polymerase ii 8.2 kDa polypeptide (Ec 2.7.7.6)(Rpb10) (Rp10) (Abc10),		0.72
		1800	TC338904	UP|Q8L8I4_ORYSA (Q8L8I4) RNase L inhibitor-like protein, 29%		0.87
		2130	TC334693	UP|Q29PX1_ARATH (Q29PX1) At5g08565, 84%		-0.48
		4881	TC325948	UP|RPO2J_ARATH (Q38859) DNA-directed RNA polymerase II subunit J, 93%		0.92
		5780	TC319244	RF|XP_507586.1|51979679|XM_507586 P0524F03.33 gene product {Oryza sativa (japonica cultivar-group)}, 73%		-0.60
		6464	TC327098	RF|NP_174629.2|42562495|NM_103088 nucleic acid binding {Arabidopsis thaliana}, 4%		0.40
		7569	TC328209	UP|RPB2_LYCES (Q42877) DNA-directed RNA polymerase II 135 kDa polypeptide (RNA polymerase II subunit 2) , 9%		-0.93
		11262	TC338859	GB|AAR28026.1|39545926|AY463624 TAF9 {Arabidopsis thaliana}, 56%		-0.54
		11270	TC337611	UP|Q6NLH0_ARATH (Q6NLH0) At3g16980, 39%		-0.80
Secondary	Isoprenoids	2709	TC360874	UP|Q71RX2_MAIZE (Q71RX2) Isopentenyl pyrophosphate isomerase, 68%		-0.35

metabolism		3797	TC349841	UP|Q944G1_HEVBR (Q944G1) Phosphomevalonate kinase, 21%		0.72
	N misc	4652	TC322649	UP|Q8H5F0_ORYSA (Q8H5F0) Betaine aldehyde dehydrogenase-like, 96%		-0.80
	Phenylpropanoids	5879	TC321542	UP|ZRP4_MAIZE (P47917) O-methyltransferase ZRP4, 69%		-0.73
		1732	-	-		0.97
	Wax	6104	TC328714	UP|Q2HW67_MEDTR (Q2HW67) Sterol desaturase, 54%		-0.69
Signaling	14-3-3 proteins	2889	TC321724	UP|Q6PLR9_MAIZE (Q6PLR9) 14-3-3-like protein		-0.74
		2903	TC367395	UP|14331_MAIZE (P49106) 14-3-3-like protein GF14-6		-1.14
		4888	TC322255	UP|Q6PLR9_MAIZE (Q6PLR9) 14-3-3-like protein, 89%		-0.94
		6488	TC316225	UP|Q6XNL1_9POAL (Q6XNL1) 14-3-3-like protein		1.06
		6739	TC324017	UP|Q6PLR9_MAIZE (Q6PLR9) 14-3-3-like protein		-0.51
		8554	TC321724	UP|Q6PLR9_MAIZE (Q6PLR9) 14-3-3-like protein		-0.81
		9661	TC367395	UP|14331_MAIZE (P49106) 14-3-3-like protein GF14-6		-1.24
		12119	TC321724	UP|Q6PLR9_MAIZE (Q6PLR9) 14-3-3-like protein		-0.82
		12153	TC316225	UP|Q6XNL1_9POAL (Q6XNL1) 14-3-3-like protein		-0.79
		12522	TC360784	UP|14331_MAIZE (P49106) 14-3-3-like protein GF14-6		-1.13
	Calcium	1174	TC328342	UP|Q5NBP9_ORYSA (Q5NBP9) Protein kinase C substrate 80K-H isoform 2-like, 86%		0.95
		1940	TC352796	UP|Q7DLR7_MAIZE (Q7DLR7) Calmodulin		-0.53
		3273	TC336540	UP|Q41798_MAIZE (Q41798) Calnexin, 97%	0.90	
		3771	TC326659	UP|O49184_ORYSA (O49184) Calmodulin		0.67
		4703	TC347162	UP|Q43699_MAIZE (Q43699) Calmodulin		-0.92
		5151	TC319318	UP|Q7DLR7_MAIZE (Q7DLR7) Calmodulin		0.93

		6090	TC328011	UP|Q7DLR7_MAIZE (Q7DLR7) Calmodulin		-0.63
		7217	TC323374	UP|O49184_ORYSA (O49184) Calmodulin		0.98
		8081	TC364854	UP|Q43712_MAIZE (Q43712) Calcium-binding protein precursor (Calreticulin)	1.02	
		9081	TC350199	UP|Q330Q4_ORYSA (Q330Q4) IPK, 88%		-0.48
		9743	TC345850	UP|O49184_ORYSA (O49184) Calmodulin		-0.45
	G-proteins	166	TC323818	UP|Q6AVS3_ORYSA (Q6AVS3) Expressed protein, 16%	-1.22	
		242	TC316303	UP|Q9XF08_MAIZE (Q9XF08) Rop4 small GTP binding protein		-0.84
		778	TC333977	UP|Q41137_RICCO (Q41137) Eukaryotic release factor 3, 71%		0.81
		953	TC334900	UP|O22470_ORYSA (O22470) GDP dissociation inhibitor protein OsGDI1		0.76
		1912	TC327526	UP|Q9FEV1_ORYSA (Q9FEV1) RAB5A protein		-0.93
		1960	TC320901	UP|RAC6_ORYSA (Q6ZHA3) Rac-like GTP-binding protein 6 (OsRac6) (GTPase protein RacB)		-0.63
		2731	TC342487	UP|Q7Y0Q8_ORYSA (Q7Y0Q8) Rac GDP-dissociation inhibitor 1, 67%		-0.33
		3665	TC350493	RF|NP_199419.1|15237372|NM_123975 GTP binding {Arabidopsis thaliana}, 16%		-0.82
		3671	TC335389	UP|Q656P7_ORYSA (Q656P7) Root hair defective 3 GTP-binding protein-like, 40%		-0.43
		4280	TC343626	UP|Q08152_PEA (Q08152) GTP-binding protein, 88%		-0.57
		4470	TC323232	Zea mays clone EL01N0314E02.c mRNA sequence		0.54
		6323	TC346614	UP|YPTM2_MAIZE (Q05737) GTP-binding protein YPTM2		-0.39
		6750	TC350606	UP|YPTM2_MAIZE (Q05737) GTP-binding protein YPTM2		-0.60
		7310	TC341709	UP|O22470_ORYSA (O22470) GDP dissociation inhibitor protein OsGDI1		-0.69
		8146	-	-		1.10
		8570	TC335465	GB|AAL16275.1|16226834|AF428345 AT3g54190/F24B22_150 {Arabidopsis thaliana}, 35%		-0.55

		8580	TC330700	UP|Q4KDR7_PSEF5 (Q4KDR7) Efflux protein, LysE family, 7%		-0.67
		10505	TC345387	UP|Q7GD79_ORYSA (Q7GD79) Small GTP-binding protein (Ran2)		-0.39
		12553	TC332681	UP|RAB7_PENCL (Q40787) Ras-related protein Rab7		-0.44
	In sugar and nutrient physiology	769	TC336760	UP|Q7Y0S8_SOLTU (Q7Y0S8) Erg-1, 42%		0.59
		6907	TC343667	RF|XP_506983.1|51964396|XM_506983 P0627E03.37-2 gene product {Oryza sativa (japonica cultivar-group)}, 19%		1.01
	MAP kinases	6924	TC327899	RF|NP_191892.2|22331922|NM_116198 ATP binding {Arabidopsis thaliana}, 44%		-0.32
	Misc	1019	TC334435			0.76
	Receptor kinases	3444	TC343058	UP|Q84SG9_ORYSA (Q84SG9) Serine/threonine kinase receptor-like protein, 28%		-0.65
		5674	TC315927	UP|Q94IJ5_MAIZE (Q94IJ5) SERK2 protein precursor		-0.95
		9731	TC334736	UP|Q4ABW3_BRARP (Q4ABW3) 4D11_24, 38%		0.75
		8166	TC330342	UP|Q6Y2W9_MAIZE (Q6Y2W9) Atypical receptor-like kinase MARK		0.73
		14968	TC323107	UP|O81105_MAIZE (O81105) Leucine-rich repeat transmembrane protein kinase 1		-0.25
		2068	TC344861	RF|NP_195341.2|30690596|NM_119785 ATP binding {Arabidopsis thaliana}, 8%		1.03
		5130	TC371000	UP|Q5ZBB1_ORYSA (Q5ZBB1) Dual-specific kinase DSK1-like, 6%		-0.28
		10738	TC340970	UP|O49974_MAIZE (O49974) KI domain interacting kinase 1, 20%		-0.38
Stress	Abiotic.cold	3422	TC347320			-0.70
		6756	TC355196	UP|O82787_ORYSA (O82787) Early nodulin, 98%		-0.35
		7603	TC349797	Zea mays clone Contig456 mRNA sequence		-0.86
	Abiotic.drought/salt	5076	TC327696	RF|NP_174675.2|30693010|NM_103136 STT3B (STAUROSPORIN AND TEMPERATURE SENSITIVE 3-LIKE B) oligosaccharyl transf		0.64
		10180	TC336873	GB|ABB47874.1|78708899|AE016959 expressed protein {Oryza sativa (japonica cultivar-group)}, 14%		-0.77

	Abiotic.heat	863	TC347411	UP|Q9MB32_ORYSA (Q9MB32) Heat shock protein 90, 19%		0.89
		2165	TC346261	UP|Q67VC3_ORYSA (Q67VC3) ARG1-like protein, 55%		-0.52
		2464	TC366587	Zea mays clone EL01N0372B09.c mRNA sequence		-0.67
		2524	TC316727	UP|Q53NM9_ORYSA (Q53NM9) DnaK-type molecular chaperone hsp70-rice		-0.46
		2722	TC340577	Zea mays clone Contig439 mRNA sequence		-0.40
		4727	TC337494	UP|HSP81_ORYSA (P33126) Heat shock protein 81-1		-0.49
		9842	TC321298	UP|HSP81_ORYSA (P33126) Heat shock protein 81-1, 64%		-0.52
		10568	TC340794	UP|Q40867_PENAM (Q40867) Heat shock protein 17.9	1.16	
	Abiotic.light	2728	TC317372	RF|XP_506822.1|51964078|XM_506822 P0470G10.15 gene product {Oryza sativa (japonica cultivar-group)}, 91%		-0.38
		5276	TC317372	RF|XP_506822.1|51964078|XM_506822 P0470G10.15 gene product {Oryza sativa (japonica cultivar-group)}, 91%		-0.54
	Abiotic.touch/wounding	2437	TC355955	UP|PR4_PHAVU (Q09020) Wound-induced basic protein, 40%		0.70
		3514	TC331915	RF|XP_507201.1|51964834|XM_507201 P0426E02.15-2 gene product {Oryza sativa (japonica cultivar-group)}, 42%		1.26
	Abiotic.unspecified	4163	TC332882	UP|Q9LWS6_ORYSA (Q9LWS6) Ozone-responsive stress-related protein-like		0.58
		6933	TC369189	GB|AAN31783.1|23396189|AC134516 Putataive pollen specific protein C13 precursor {Oryza sativa (japonica cultivar		-0.47
		13697	TC342426	UP|Q6TM44_MAIZE (Q6TM44) Germin-like protein, 66%		-0.81
	Biotic	317	TC369550	UP|Q9SWZ5_WHEAT (Q9SWZ5) Secretory protein, 89%		0.69
		1176	TC328675	UP|Q40066_HORVU (Q40066) Protein zx, 71%		0.51
		3886	TC342468	Zea mays clone Contig17 mRNA sequence		-0.33
		11986	TC321039	UP|Q60ED8_ORYSA (Q60ED8) Von Willebrand factor type A domain containing protein, 65%		-0.41

TCA / org	Transformation	2235	TC369443	Zea mays clone Contig506.F mRNA sequence		-0.60
		3737	TC320434			-0.54
		5803	TC320434			-0.71
		11843	TC353455	UP|MAOC_MAIZE (P16243) NADP-dependent malic enzyme, chloroplast precursor		-0.63
		9264	TC358393	Zea mays clone cen3n.pk0128.b3, mRNA sequence		-1.17
		10660	TC346935	UP|Q9SIB9_ARATH (Q9SIB9) Cytoplasmic aconitate hydratase, 15%		-0.55
		4055	TC316108	UP|Q9XGU7_ORYSA (Q9XGU7) NADP-specific isocitrate dehydrogenase	0.90	
		4378	TC327187	UP|Q9SWR9_MAIZE (Q9SWR9) Dihydrolipoamide S-acetyltransferase		-0.71
		11450	TC334565	RF|XP_507349.1|51978944|XM_507349 P0507H12.6 gene product {Oryza sativa (japonica cultivar-group)}, 7%		-0.36
		3134	TC325985	RF|XP_506871.1|51964174|XM_506871 OJ1234_B11.18 gene product {Oryza sativa (japonica cultivar-group)}		-0.35
Tetrapyrrole synthesis	GSA	10437	TC349802			1.01
	Heme oxygenase	103	TC338748	UP|Q94FW9_SORBI (Q94FW9) Heme oxygenase 1, 98%		-0.77
	Porphobilinogen deaminase	3118	TC316595	UP|Q8RYB1_WHEAT (Q8RYB1) Porphobilinogen deaminase		-0.90
	Sirohydrochlorin ferrochelatase	7178	TC342730	UP|Q9XE75_SORBI (Q9XE75) Patatin-like protein, 22%		-0.47
Transport	ABC transporters and multidrug resistance systems	5041	-	-		-0.59
		12636	TC326679	UP|Q6Y3H9_MAIZE (Q6Y3H9) Multidrug resistance associated protein 2, 32%		-0.95
	Amino acids	4158	-	-		0.37
		9845	TC336284	UP|Q9LUH7_ORYSA (Q9LUH7) Amino acid permease, 56%		-0.53
	H+ transporting pyrophosphatase	5124	TC353090	UP|Q5K3Q7_MAIZE (Q5K3Q7) Vacuolar H+-translocating inorganic pyrophosphatase		0.62

	Major Intrinsic Proteins.PIP	325	TC325757	UP|Q9AQU5_MAIZE (Q9AQU5) Plasma membrane integral protein ZmPIP1-4		-0.60
		12470	TC370281	UP|PTH2_DROME (O97067) Probable peptidyl-tRNA hydrolase 2 (PTH 2) , 22%		-0.43
	Metabolite transporters at the envelope membrane	2302	TC333906	UP|Q59IV5_MESCR (Q59IV5) Plastidic phosphate translocator-like protein2, 98%		-0.71
		1227	TC316120	Zea mays clone EL01N0561A03.c mRNA sequence		0.45
		3311	TC317834	UP|ADT_ORYSA (P31691) ADP,ATP carrier protein, mitochondrial precursor, 82%		-0.79
		11543	TC356083	GB|AAK96620.1|15450697|AY052716 AT5g01340/T10O8_50 {Arabidopsis thaliana}, 27%		-1.31
	Metal	2334	TC346872	UP|Q2R041_ORYSA (Q2R041) Magnesium/proton exchanger AtMHX, 41%		-0.77
	Misc	4728	TC363898	UP|Q9LSH7_ARATH (Q9LSH7) Transporter-like protein, 25%		-0.48
		7716	TC358328			-0.96
		8151	TC317022	UP|Q2TJ67_ORYSA (Q2TJ67) Plastid ATP/ADP transporter, 74%		0.94
		10925	TC317022	UP|Q2TJ67_ORYSA (Q2TJ67) Plastid ATP/ADP transporter, 74%		0.80
	P- and v-ATPases.H+-transporting two-sector ATPase	2701	TC329393	UP|VATF_ARATH (Q9ZQX4) Probable vacuolar ATP synthase subunit F , 98%		-0.32
		3213	TC342725	UP|VATL_AVESA (P23957) Vacuolar ATP synthase 16 kDa proteolipid subunit, 55%		0.62
	Porins	5838	TC321095	UP|Q7Y1C6_PENAM (Q7Y1C6) PgPOR29 (Voltage dependent anion channel protein)		-0.43
	Potassium	644	TC332550	UP|Q9AYN7_PHRAU (Q9AYN7) High-affinity potassium transporter, 28%		-0.80
		2952	TC344894	UP|HAK2_ORYSA (Q942X8) Probable potassium transporter 2 (OsHAK2), 24%		-0.83
	Unspecified anions	4801	-	-		-0.48
		4201	TC327920	UP|Q2VT97_9POAL (Q2VT97) Na+/H+ antiporter		-0.67
Transporter	Sugars	5086	TC325057	RF|NP_567083.1|18411150|NM_115798 nucleotide-sugar transporter/ sugar porter {Arabidopsis thaliana}, 81%		0.37
		10468	TC324798	UP|Q8GT52_HORVU (Q8GT52) Hexose transporter, 23%		-0.62

Not assigned	No ontology	241	TC341143	GB|AAP54521.1|31432950|AE016959 expressed protein {Oryza sativa (japonica cultivar-group)}, 62%		-0.74
		508	TC322451	RF|NP_177656.2|22330642|NM_106176 FMN binding {Arabidopsis thaliana}, 19%		0.48
		582	TC321141	RF|XP_507481.1|51979234|XM_507481 OJ1342_D02.8 gene product {Oryza sativa (japonica cultivar-group)}, 84%		0.61
		609	TC316200	UP|Q9M582_MAIZE (Q9M582) Hypersensitive-induced response protein		0.80
		805	TC370071	UP|Q8H6A5_MAIZE (Q8H6A5) Translationally controlled tumor protein-like protein		0.90
		814	TC370071	UP|Q8H6A5_MAIZE (Q8H6A5) Translationally controlled tumor protein-like protein		0.65
		835	TC330220	RF|NP_567410.1|18414065|NM_117446 hydrolase/ pyrophosphatase {Arabidopsis thaliana}, 96%		0.97
		872	TC362570	Zea mays clone EL01N0560B10.c mRNA sequence		0.69
		883	TC325586	UP|Q84PA9_ORYSA (Q84PA9) Sarcoplasmic reticulum protein (With alternative splicing), 78%		1.16
		913	TC323432	GB|AAF50128.1|7294794|AE003547 CG7949-PA {Drosophila melanogaster}, 42%		0.98
		918	TC355837	UP|Q5VRJ8_ORYSA (Q5VRJ8) Peroxisomal biogenesis factor 11 protein-like, 98%		0.86
		1002	TC331019	UP|Q8L9L3_ARATH (Q8L9L3) Contains similarity to pyridoxamine 5-phosphate oxidase, 43%		0.58
		1055	TC330294	Zea mays clone EL01N0440F12.c mRNA sequence		0.50
		1116	TC321594	UP|Q9ATV7_HORVU (Q9ATV7) Arabinoxylan arabinofuranohydrolase isoenzyme AXAH-II, 83%		1.09
		1236	TC370071	UP|Q8H6A5_MAIZE (Q8H6A5) Translationally controlled tumor protein-like protein		0.57
		1400	TC343036	RF|NP_193367.2|42566868|NM_117729 SPK1 (SPIKE1) {Arabidopsis thaliana}, 30%		0.78
		1659	TC321171	UP|Q9LK52_ARATH (Q9LK52) Dbj|BAA90629.1		-0.99
		1688	TC324578	RF|NP_200011.1|15242242|NM_124577 nucleic acid binding {Arabidopsis thaliana}, 70%		-1.02
		1835	TC348384	UP|Q9SFU7_ARATH (Q9SFU7) T1B9.17 protein (AT3g07170/T1B9_17), 42%		-0.90
		1854	TC340710	UP|Q53AN3_ORYSA (Q53AN3) Gibberellin-stimulated protein, 75%		0.84

1907	TC328506			-0.54
2285	TC331398			0.56
2358	TC323187	Zea mays clone EL01N0444B10.c mRNA sequence		0.55
2371	TC323589	UP|Q564K3_ARATH (Q564K3) Chromosome associate protein subunit H, 18%		-0.36
2380	TC327194			-0.42
2408	TC319161			1.51
2462	TC319517			1.22
2518	TC317520	UP|Q1WUN6_9LACO (Q1WUN6) Dephospho-CoA kinase, 72%		0.43
2683	TC337690	UP|Q9SUL3_ARATH (Q9SUL3) OBP33PEP like protein, 59%		-0.98
2825	TC316327	Zea mays clone Contig41 mRNA sequence		-0.52
2860	TC322865	GB|AAO44081.1|28466945|BT004815 At3g57000 {Arabidopsis thaliana}, 56%		0.82
2920	TC331687			0.92
2961	TC344657	UP|Q2RAM9_ORYSA (Q2RAM9) DTW domain, 44%		0.61
3107	TC332325	UP|Q2R177_ORYSA (Q2R177) Expressed protein, 60%		-0.56
3138	TC340029	RF|XP_749417.1|70989135|XM_744324 yippee zinc-binding protein {Aspergillus fumigatus Af293}, 71%		-0.40
3272	TC335804	Zea mays clone Contig94.F mRNA sequence		-0.50
3368	TC320481	UP|Q8H1Q2_ARATH (Q8H1Q2) Nucleotide-binding protein, 88%		0.62
3436	TC336158	UP|Q5NA53_ORYSA (Q5NA53) Glycogenin-like protein, 19%		0.95
3522	TC336353	UP|Q8RYN2_ORYSA (Q8RYN2) Auxilin-like protein, 6%		0.73
3628	TC321458	UP|Q9LN36_ARATH (Q9LN36) F18O14.34, 52%		0.49
3762	TC322758	UP|Q3E9H4_ARATH (Q3E9H4) Protein At5g15550, 28%		1.34

3774	TC353250			0.80
3837	TC327577	RF|NP_195140.2|30689939|NM_119577 nucleic acid binding {Arabidopsis thaliana}, 37%		-0.34
3878	TC349744	UP|NUD23_ARATH (P93740) Nudix hydrolase 23, chloroplast precursor (AtNUDT23) , 69%		-0.51
3924	TC347884	UP|Q75ID2_ORYSA (Q75ID2) Expressed protein, 49%		0.42
4009	-	-		0.45
4049	TC333587	Zea mays clone EL01N0413D09.c mRNA sequence		0.30
4183	-	-		-0.47
4234	TC336299	RF|XP_506823.1|51964080|XM_506823 P0470G10.26 gene product {Oryza sativa (japonica cultivar-group)}, 71%		-0.61
4237	TC370251	UP|G10A_ORYSA (Q94DE2) Protein G10 homolog 1		-0.57
4289	TC345185	UP|Q5JN36_ORYSA (Q5JN36) Magmas-like protein, 98%		-0.92
4295	TC363760	UP|Q69SJ5_ORYSA (Q69SJ5) NatC N(Alpha)-terminal acetyltransferase, Mak10 subunit-like, 32%		-0.50
4345	TC351372	UP|EBP2_ARATH (Q9LUJ5) Probable rRNA-processing protein EBP2 homolog, 58%		-0.67
4357	TC327629	RF|XP_507481.1|51979234|XM_507481 OJ1342_D02.8 gene product {Oryza sativa (japonica cultivar-group)}, 82%		-0.76
4483	TC320197	UP|Q5JM91_ORYSA (Q5JM91) Ripening-related protein-like, 86%		0.44
4628	TC333123	UP|Q5JL23_ORYSA (Q5JL23) ABC-type transport system-like, 83%		-0.33
4647	TC328348	UP|Q6Z7E3_ORYSA (Q6Z7E3) Microsomal signal peptidase 25 kDa subunit(SPC25)-like protein, 93%		-0.86
4749	TC331877	RF|XP_507328.1|51965088|XM_507328 OJ1125_C01.28 gene product {Oryza sativa (japonica cultivar-group)}, 31%		-0.51
4751	TC326894	UP|Q9AR47_MAIZE (Q9AR47) VIP3 protein		-0.41
4775	TC335870	RF|NP_195600.1|15234862|NM_120049 FAD binding {Arabidopsis thaliana}, 18%		-0.35

4843	TC324875	RF|NP_850027.2|42570301|NM_179696 oxidoreductase {Arabidopsis thaliana}, 62%		1.12
4924	TC341434			-0.37
5196	TC324578	RF|NP_200011.1|15242242|NM_124577 nucleic acid binding {Arabidopsis thaliana}, 70%		0.41
5327	TC340710	UP|Q53AN3_ORYSA (Q53AN3) Gibberellin-stimulated protein, 75%		0.87
5464	TC327635	UP|Q7Y1Z2_WHEAT (Q7Y1Z2) 27K protein, 82%		-0.18
5671	TC319799	GB|1604369A|226743|1604369A sulfated surface glycoprotein SSG185. {Volvox carteri}, 9%		0.73
5677	TC356965	UP|Q6K3S8_ORYSA (Q6K3S8) Prefoldin-like, 89%		0.94
5737	TC321171	UP|Q9LK52_ARATH (Q9LK52) Dbj|BAA90629.1		-0.75
6017	TC370071	UP|Q8H6A5_MAIZE (Q8H6A5) Translationally controlled tumor protein-like protein	0.93	
6020	TC328339	UP|O22875_ARATH (O22875) Expressed protein, 83%		0.98
6103	TC345447	UP|YIPL_SOLTU (P59234) Protein yippee-like		-0.61
6532	TC319799	GB|1604369A|226743|1604369A sulfated surface glycoprotein SSG185. {Volvox carteri}, 9%		1.14
6563	TC332756			-0.42
6624	TC330434	UP|Q6H5H5_ORYSA (Q6H5H5) Emsy N terminus domain-containing protein-like, 87%		-0.53
6862	TC333448	Zea mays clone Contig485.F mRNA sequence		1.29
6908	TC335188	UP|Q7M4Q5_HUMAN (Q7M4Q5) Basic proline-rich peptide IB-8a, 36%	1.18	
7646	TC337887	UP|Q6Z3Y5_ORYSA (Q6Z3Y5) PWWP domain protein-like, 43%		-0.79
8022	TC323729	UP|Q2V3U6_ARATH (Q2V3U6) Protein At3g18850, 63%		-0.64
8391	TC361049	UP|Q9ZVF6_ARATH (Q9ZVF6) Expressed protein (At2g01490/F2I9.11), 66%		-0.45
8473	TC336091	UP|Q9M578_ORYSA (Q9M578) XIG, 95%		-0.60
8845	TC322293	UP|Q5N9K2_ORYSA (Q5N9K2) BAG domain containing protein-like, 90%		0.78

8862	TC354302	UP|Q5JN62_ORYSA (Q5JN62) Disease resistance protein-like, 42%		-0.39
9054	TC353155	UP|WDR50_ARATH (Q9FMU5) WD-repeat protein At5g14050, 40%		-0.54
9621	TC316993	UP|Q5VQP7_ORYSA (Q5VQP7) Leucine-rich repeat protein, 94%		-0.65
9623	TC354566	UP|Q6ZJG4_ORYSA (Q6ZJG4) HAD superfamily protein involved in N-acetyl-glucosamine catabolism-like		-0.61
9782	TC345704	UP|Q2QTY5_ORYSA (Q2QTY5) Lysosomal Cystine Transporter		-0.88
9878	TC343185	UP|Q8LFK0_ARATH (Q8LFK0) Beta-N-acetylhexosaminidase-like protein, 79%		-0.82
9899	TC332488	UP|Q67X40_ORYSA (Q67X40) Integral membrane family protein-like, 98%		-0.70
10229	TC315963	Zea mays clone Contig654 mRNA sequence		-0.78
10239	TC317205	Zea mays clone EL01N0426H04.c mRNA sequence		-0.71
10306	TC329533	GB|AAK91338.1|15215586|AY050321 AT4g23630/F9D16_100 {Arabidopsis thaliana}, 78%		-0.27
10836	TC329817	UP|Q8LQG2_ORYSA (Q8LQG2) Selenium-binding protein-like, 43%	0.67	
11207	TC326846			0.72
11677	TC329320	UP|Q2QYS8_ORYSA (Q2QYS8) Light-inducible protein ATLS1, 61%		-0.87
11751	TC335566	UP|Q6DBH4_ARATH (Q6DBH4) At2g23820, 70%		-0.69
11779	TC325392	UP|Q3E7S6_ARATH (Q3E7S6) Protein At5g53050, 65%		-1.36
12169	TC317597	RF|XP_507542.1|51979391|XM_507542 OJ1249_F12.26 gene product {Oryza sativa (japonica cultivar-group)}		-0.49
12444	TC317050	Zea mays clone Contig43 mRNA sequence		0.80
12648	-	-		-1.27
12776	TC351863	GB|CAA66406.1|1495267|ATORF12 {Arabidopsis thaliana}, 8%		-0.49
12865	TC338722	GB|AAD31570.1|4883601|AC006922 expressed protein {Arabidopsis thaliana}, 50%		0.68

		1363	TC326117	RF|XP_507202.1|51964836|XM_507202 P0488B06.44 gene product {Oryza sativa (japonica cultivar-group)}, 76%		0.80
		10342	TC320968			-0.44
		10528	TC323380	UP|Q41719_ZEADI (Q41719) Hydroxyproline-rich glycoprotein precursor, 98%		1.19
		111	TC316064	Zea mays clone EL01N0302C07.d mRNA sequence		-0.81
		1426	TC338962	UP|Q53Q87_ORYSA (Q53Q87) Expressed protein, 28%		0.89
		1567	TC338770			1.83
		11974	TC336786	UP|Q66GI4_ARATH (Q66GI4) At2g32230, 36%		-0.77
		3446	TC337817	GB|AAP54807.1|31433269|AE016959 expressed protein {Oryza sativa (japonica cultivar-group)}, 89%		0.78
	Unknown	331	TC339325			-1.38
		402	TC338766	Zea mays clone EL01N0302D08.c mRNA sequence		0.80
		533	TC316503			0.71
		560	TC328330	GB|AAO24579.1|27808598|BT003147 At1g36050 {Arabidopsis thaliana}, 30%		-0.56
		565	-	-		0.81
		572	TC323761	RF|NP_496363.1|17533631|NM_063962 LEThal family member (let-858) {Caenorhabditis elegans}, 3%		1.08
		873	-	-		0.59
		961	TC328078	UP|Q52ZI4_PEA (Q52ZI4) Expressed protein, 36%		0.63
		1154	TC328188	Zea mays clone EL01N0447F03.d mRNA sequence		0.73
		1226	TC318349			0.59
		1239	TC363968	RF|NP_849575.1|30678203|NM_179244 ATP binding {Arabidopsis thaliana}, 62%		-0.46
		1465	TC348647	UP|Q2QNJ0_ORYSA (Q2QNJ0) Expressed protein, 33%		0.84

		1529	TC329567	UP|Q8RUX8_ARATH (Q8RUX8) Expressed protein, 82%		-0.35
		1543	TC324966	UP|Q7XDQ5_ORYSA (Q7XDQ5) Expressed protein, 8%		0.56
		1629	TC321092	UP|Q53LP8_ORYSA (Q53LP8) Seed protein B32E, 62%		-1.04
		1674	TC332810	UP|Q27A62_MYCFV (Q27A62) Peptidase M24, 4%		-0.73
		1686	TC362044	GB|AAK63864.1|14488088|AF389292 AT4g01150/F2N1_18 {Arabidopsis thaliana}, 26%		-0.84
		1739	-	-		1.04
		1831	TC368976			-0.63
		1833	TC355940			-0.91
		1850	TC333934			1.26
		1917	TC341606	UP|Y2766_ARATH (O80934) Protein At2g37660, chloroplast precursor, 79%		0.76
		1939	TC350148	UP|Q2R1Z4_ORYSA (Q2R1Z4) Expressed protein, 65%		-0.76
		1957	TC324188	UP|Q94AD3_ARATH (Q94AD3) AT4g33690/T16L1_180, 28%		-0.46
		1965	TC358004	UP|Q29SB6_MAIZE (Q29SB6) Pathogenesis-related protein 10		-0.98
		2095	TC319490			1.18
		2246	TC318123	Zea mays clone EL01N0557A09.c mRNA sequence		-0.52
		2411	TC326557		0.35	
		2619	TC340506	UP|Q4ABY1_BRARP (Q4ABY1) 4D11_6, 47%		-0.83
		2688	TC345002			0.78
		2873	TC340730	GB|AAL16180.1|16226487|AF428412 At3g07760/F17A17.10 {Arabidopsis thaliana}, 94%		-0.42
		2892	TC338905			0.66
		2925	TC327961			0.44

2926	TC319292			0.89
2958	TC347955	PRF|2117429U|1582125|2117429U protamine P1. {Phascolarctos cinereus} , 40%		0.79
2964	TC320301	UP|Q6NLR2_ARATH (Q6NLR2) At1g64650, 57%		-0.48
3039	TC363450	UP|UTP11_ORYSA (Q8S1Z1) Probable U3 small nucleolar RNA-associated protein 11 (U3 snoRNA-associated protein 11),		-0.35
3069	-	-		-0.77
3080	TC337332	UP|Q501F5_ARATH (Q501F5) At4g26980, 18%		-0.46
3086	TC338043	UP|INSM1_HUMAN (Q01101) Insulinoma-associated protein 1 (Zinc finger protein IA-1), 6%		-0.46
3093	TC355900	UP|Q75GR6_ORYSA (Q75GR6) Ribosomal protein L17-like protein, 16%		0.95
3209	TC320051	UP|Q9SXG0_ORYSA (Q9SXG0) F1F0-ATPase inhibitor protein, 93%		0.70
3276	TC345400	UP|Q6YYC3_ORYSA (Q6YYC3) RNA polymerase II complex component SRB7 protein-like, 61%	1.19	
3324	-	-		-0.71
3334	TC324583	GB|AAC28224.1|3377842|T27D20 {Arabidopsis thaliana}, 70%		1.09
3392	TC342886	UP|Q8RX56_ARATH (Q8RX56) AT5g06970/MOJ9_14, 10%		1.36
3408	TC329437	Zea mays clone Contig96 mRNA sequence		-0.54
3458	TC341024			0.71
3661	TC331984	UP|Q53L24_ORYSA (Q53L24) Expressed protein, 95%		-0.73
3903	TC333376	UP|Q6YUB8_ORYSA (Q6YUB8) Major intrinsic protein-like, 50%		-0.36
4073	TC316370	Zea mays clone EL01N0519E08.c mRNA sequence		-0.48
4125	TC320099	UP|Q6YUX0_ORYSA (Q6YUX0) DNA binding protein-like		1.24
4524	TC365273	RF|XP_507376.1|51978998|XM_507376 OJ1699_E05.18 gene product {Oryza sativa (japonica cultivar-group)}, 87%		-0.34

4540	TC317567	UP|Q9SFU9_ARATH (Q9SFU9) T1B9.14 protein (At3g07190), 77%		1.51
4609	TC329705	UP|Q9FL94_ARATH (Q9FL94) Gb|AAC61821.1, 67%		-0.71
4684	TC326941	UP|Q6YZC6_ORYSA (Q6YZC6) Cation exchanger-like protein, 98%		-1.27
4705	TC339831			-0.81
4824	TC322414	UP|Q2IG67_ANADE (Q2IG67) Tetratricopeptide repeat protein, 3%		1.04
4835	TC362091	Zea mays clone Contig314 mRNA sequence		0.56
4956	TC339430			1.09
4995	TC316970	RF|NP_001006353.1|57530671|NM_001006353 coiled-coil domain containing 12 {Gallus gallus}, 27%		0.69
5053	TC332551	Zea mays clone EL01N0553A05.c mRNA sequence		0.91
5070	TC347622	UP|Q9MAC4_ARATH (Q9MAC4) T4P13.18 protein, 64%		0.87
5080	TC336410	UP|Q9LNA6_ARATH (Q9LNA6) F5O11.12, 61%		0.97
5146	TC327522			0.74
5676	TC323761	RF|NP_496363.1|17533631|NM_063962 LEThal family member (let-858) {Caenorhabditis elegans}, 3%		-0.51
5685	TC320051	UP|Q9SXG0_ORYSA (Q9SXG0) F1F0-ATPase inhibitor protein, 93%		-0.60
5853	TC351067			-0.82
5857	TC355569			-0.42
5913	TC350127			0.77
5915	TC356699	UP|Q8IP68_DROME (Q8IP68) CG31813-PA, 13%		-0.80
6117	TC345681			-0.67
6145	TC329567	UP|Q8RUX8_ARATH (Q8RUX8) Expressed protein, 82%		-0.54

		6179	TC364634	Zea mays clone Contig864.F mRNA sequence		-0.44
		6342	TC334850			-0.81
		6410	TC339717	UP|U139_ARATH (Q9SD88) UPF0139 protein At5g07960, 85%		1.01
		6460	TC320051	UP|Q9SXG0_ORYSA (Q9SXG0) F1F0-ATPase inhibitor protein, 93%		0.49
		6495	TC321853	Zea mays clone EL01N0553G07.c mRNA sequence		0.51
		6496	TC321853	Zea mays clone EL01N0553G07.c mRNA sequence		1.13
		6510	TC320447	UP|Q6NM29_ARATH (Q6NM29) At2g15730, 56%		-0.37
		6520	TC348434	small subunit ribosomal protein S28		-0.41
		6529	TC368543			0.80
		6566	TC354908	UP|Q6ST18_MAIZE (Q6ST18) Heat shock factor binding protein 2		-0.46
		6768	TC347794	UP|Q6NQD2_ARATH (Q6NQD2) At5g12240 (MRNA, complete cds, clone: RAFL22-05-F16), 18%		-0.63
		6925	TC357583	RF|XP_507110.1|51964652|XM_507110 OJ1163_G08.28 gene product {Oryza sativa (japonica cultivar-group)}, 84%		0.77
		6962	-	-		0.79
		7353	TC324524	RF|XP_473409.1|50927577|XM_473409 {Oryza sativa (japonica cultivar-group)}, 86%		-0.79
		7526	TC344580			-0.47
		7534	TC334510			-0.56
		7539	TC366999			-0.49
		7548	TC325645			-0.31
		7753	-	-		-0.44
		7979	TC336473	UP|Q6NPD1_ARATH (Q6NPD1) At5g62960, 27%		0.90
		8112	TC356037			1.50

8223	TC327180	UP|Q64HC0_MAIZE (Q64HC0) ASF/SF2-like pre-mRNA splicing factor SRP30		-0.58
8241	TC352948	UP|Q2XX25_ZEAMP (Q2XX25) Phospholipid transfer protein 1		-0.84
8283	TC330541	UP|Q2QNG6_ORYSA (Q2QNG6) Expressed protein		-0.67
8305	TC324890	UP|Q41810_MAIZE (Q41810) Glycine-rich protein		-0.29
8463	TC321976			0.58
8483	TC335240		1.48	
8549	TC322706			0.89
8861	TC350092			-0.48
8891	-	-		0.76
8983	TC329841			0.97
9153	TC331545	GB|BAC16499.1|23237926|AP005198 elongation factor 1 beta {Oryza sativa (japonica cultivar-group)}		-0.57
9257	TC345977	UP|Q6KA74_ORYSA (Q6KA74) Ankyrin repeat protein-like, 53%		-0.42
9314	TC333033	UP|Q651K5_ORYSA (Q651K5) AlphaSNBP(B)-like, 11%		-0.67
9551	TC351090	GB|1WVK_A|58177252|1WVK_A Chain A, Nmr Solution Structure Of The Partially Disordered Protein At2g23090 From {Ar		-0.40
9608	TC325833	Zea mays clone EL01T0403C05.c mRNA sequence		1.21
9684	TC340759	UP|Q762B4_ORYSA (Q762B4) BRI1-KD interacting protein 103, 28%		0.38
9705	TC335637	UP|Q2V2V7_ARATH (Q2V2V7) Protein At5g64400, 48%		-0.35
9718	TC316721	UP|Q3ED49_ARATH (Q3ED49) Protein At1g29980, 82%		0.81
9740	TC342082	RF|NP_850429.1|30689895|NM_180098 structural constituent of nuclear pore {Arabidopsis thaliana}, 29%		0.79
9776	TC323503	GB|AAF76881.1|8571429|AF251279 thioredoxin {Schizosaccharomyces pombe}, 54%		-0.64

9779	TC335114			0.78
9881	TC324002	UP|Q9LV43_ARATH (Q9LV43) Arabidopsis thaliana genomic DNA, chromosome 3, P1 clone: MOB24, 7%		0.58
9916	TC353961	UP|O64572_ARATH (O64572) Expressed protein, 3%		0.70
9931	TC322521	UP|Q2QQ99_ORYSA (Q2QQ99) Expressed protein		-0.98
9947	TC340418	GB|AAP37753.1|30725462|BT008394 At4g28200 {Arabidopsis thaliana}, 13%		-0.57
9948	TC350024	UP|Q337L1_ORYSA (Q337L1) Expressed protein, 16%		-0.30
10031	TC326221	Zea mays clone EK07D2304D03.c mRNA sequence		-0.33
10096	TC344393	UP|Q8IQY7_DROME (Q8IQY7) CG32552-PA, 9%		-0.64
10115	TC340753			-0.51
10520	TC348548		0.94	
10642	TC347242	UP|Q7VCK3_PROMA (Q7VCK3) Porin homolog, 4%		-0.67
10718	TC337445	UP|Q6H512_ORYSA (Q6H512) SAM-dependent methyltransferase-like, 75%		0.79
11101	TC348647	UP|Q2QNJ0_ORYSA (Q2QNJ0) Expressed protein, 33%		-0.34
11374	TC327546	UP|Q9SXG0_ORYSA (Q9SXG0) F1F0-ATPase inhibitor protein, 93%		0.67
11501	TC342660	UP|Q7M3Z4_ILLAR (Q7M3Z4) Sperm chromatin protein I2-1, 46%		-0.71
11547	TC338201	UP|U139_ARATH (Q9SD88) UPF0139 protein At5g07960, 85%		-0.94
11634	TC341103	UP|Q4T4D2_TETNG (Q4T4D2) Chromosome undetermined SCAF9708, whole genome shotgun sequence, 3%		-0.41
11655	TC344839	UP|Q03462_MAIZE (Q03462) Opaque2 heterodimerizing protein 1		-0.43
12260	TC336554	RF|XP_805306.1|71405362|XM_800213 neurobeachin/beige protein {Trypanosoma cruzi strain CL Brener}, 3%		-0.46
12483	TC329841			-0.56

		12511	TC366355	UP|Q9FZF0_ARATH (Q9FZF0) T2E6.19, 30%		-0.52
		15248	TC343370	UP|Q9FM34_ARATH (Q9FM34) Dbj|BAA95714.1, 39%	-1.85	
		15421	TC352787	GB|AAP42751.1|30984576|BT008738 At1g64385 {Arabidopsis thaliana}, 5%		-0.54
No homology in Arabidopsis		160	TC330905	UP|Q52UU1_9ROSI (Q52UU1) Squamosa promoter binding-like protein, 18%		0.72
		267	TC358736			-0.91
		273	-	-		-0.55
		301	TC347784	UP|Q7F169_ORYSA (Q7F169) S-receptor kinase PK3-like protein, 8%		-0.76
		327	TC355418			-0.80
		341	TC338864	RF|XP_506696.1|51963826|XM_506696 P0575F10.6-2 gene product {Oryza sativa (japonica cultivar-group)}, 19%		-0.70
		345	TC339580	RF|NP_187724.2|30681617|NM_111950 structural molecule {Arabidopsis thaliana}, 10%		-0.74
		361	TC332046	UP|Q6KA74_ORYSA (Q6KA74) Ankyrin repeat protein-like, 19%		-0.65
		368	TC355122	UP|IAA30_ORYSA (P0C132) Auxin-responsive protein IAA30, 75%		-0.46
		391	TC367874			1.30
		450	-	-		0.83
		492	TC334647	UP|ROGF1_ARATH (Q93ZY2) Rop guanine nucleotide exchange factor 1, 8%		-0.47
		557	TC357376			1.27
		604	TC353373	UP|NO12B_PEA (Q00665) Early nodulin 12B precursor (N-12B), 14%		-0.48
		638	TC334809	RF|XP_481420.1|50943785|XM_481420 chloroplast RNA processing protein-like {Oryza sativa (japonica cultivar-group		0.42
		648	TC346731			0.70
		653	TC326117	RF|XP_507202.1|51964836|XM_507202 P0488B06.44 gene product {Oryza sativa (japonica cultivar-group)}, 76%		-0.45

671	TC337739	UP|Q8H5F1_ORYSA (Q8H5F1) BHLH protein-like, 59%		-0.56
687	TC351138			-0.45
713	TC343460	UP|Q6ZLD8_ORYSA (Q6ZLD8) Fiber protein-like, 21%		-0.55
787	TC370055	UP|Q9VXU4_DROME (Q9VXU4) CG6340-PA, isoform A (SD07644p), 6%		0.65
819	TC329289	Zea mays clone EL01T0206E06.c mRNA sequence		0.79
839	TC369391	Zea mays clone Contig663 mRNA sequence		0.34
923	TC315966	UP|CF23_DROME (Q01522) Chorion transcription factor Cf2, isoform III, 4%		1.13
957	TC333604	UP|Q5ZB52_ORYSA (Q5ZB52) Zinc knuckle containing protein-like, 9%		0.98
1030	TC326949			1.18
1081	TC349039	UP|Q6B6R4_ORYSA (Q6B6R4) Transcription factor WRKY07, 6%		0.83
1095	TC351197			1.31
1175	TC365024	UP|Q69K57_ORYSA (Q69K57) Smr domain-containing protein-like, 5%		0.82
1337	TC359615	RF|NP_174766.1|15219561|NM_103230 protein binding {Arabidopsis thaliana}, 10%		-0.51
1365	TC337298	UP|Q8GST0_ORYSA (Q8GST0) Auxin response factor 1, 53%		0.79
1384	TC316634	Zea mays clone EL01N0360B07.d mRNA sequence		0.93
1421	TC351045	UP|Q3E9E6_ARATH (Q3E9E6) Protein At5g18620, 56%	0.65	1.03
1443	TC345435			0.87
1444	-	-		0.75
1576	TC356660	GB|AAO64933.1|29029108|BT005998 At3g20240 {Arabidopsis thaliana}, 35%		0.92
1624	TC319752	UP|NOL10_BRARE (Q802W4) Nucleolar protein 10, 3%		-0.58
1652	TC331265	UP|Q6AWY1_ORYSA (Q6AWY1) Growth-regulating factor 8, 47%		-0.58

1682	TC328186	UP|Q441S7_SOLUS (Q441S7) Pseudouridine synthase, Rsu, 7%		-0.67
1839	TC348917	UP|Q38HS8_SOLTU (Q38HS8) Ribosomal protein L23 family protein, 52%		-0.58
1844	TC334590	UP|Q53EA9_9NEOB (Q53EA9) NADH dehydrogenase subunit II, 5%		-0.94
1857	TC348916	UP|Q7XHZ1_ORYSA (Q7XHZ1) Heat shock transcription factor-like protein, 42%		-0.88
1874	TC332760	UP|Q69IN7_ORYSA (Q69IN7) Lecithin cholesterol acyltransferase-like, 76%		0.77
1919	TC365317			-0.58
1947	TC327958	Zea mays clone EL01N0561B04.c mRNA sequence		-0.55
1953	TC342639			-0.43
1958	TC338726	UP|Q7XBD4_MAIZE (Q7XBD4) Cinful1 polyprotein, 11%		-0.76
1985	TC358672	UP|Q655C7_ORYSA (Q655C7) Ring-H2 zinc finger protein-like, 6%		-0.78
2020	TC316958	Zea mays clone Contig969.F mRNA sequence		0.77
2103	TC321559	UP|Q6VBJ3_CANGA (Q6VBJ3) Epa4p, 4%		0.79
2163	TC367463	UP|Q4A192_SACOF (Q4A192) Beclin 1 protein, 28%		0.85
2227	TC334647	UP|ROGF1_ARATH (Q93ZY2) Rop guanine nucleotide exchange factor 1, 8%		-0.56
2240	TC335787	UP|Q39050_ARATH (Q39050) Casein kinase I (AT4g14340/dl3210c) (Casein kinase 1-like protein 11), 46%		0.46
2308	TC326537	RF|XP_506802.1|51964038|XM_506802 P0403C01.30 gene product {Oryza sativa (japonica cultivar-group)}, 61%		-0.87
2312	TC331058	Zea mays clone Contig161 mRNA sequence		-0.46
2314	TC323334	UP|Q3EAC4_ARATH (Q3EAC4) Protein At4g02920, 14%		-0.55
2315	TC353616	UP|O82688_HORVU (O82688) Amino acid selective channel protein, 46%		-0.75
2396	TC332870	UP|Q339L3_ORYSA (Q339L3) Expressed protein, 57%		0.89

2429	TC341325			1.17
2469	TC363228			-0.47
2535	-	-		0.31
2548	TC337513	UP|Q2QNR7_ORYSA (Q2QNR7) CTP synthase, 4%		-0.46
2574	TC329695	UP|Q7XIW7_ORYSA (Q7XIW7) Myosin heavy chain-like, 52%		0.73
2638	TC351034	Zea mays clone EL01N0519H11.c mRNA sequence		-0.38
2645	TC316120	Zea mays clone EL01N0561A03.c mRNA sequence		-0.94
2723	TC343045			-0.44
2742	TC352793	UP|Q2VAP6_9HIV1 (Q2VAP6) Nef protein, 6%		0.50
2751	TC355712	UP|Q1YMD7_9RHIZ (Q1YMD7) Ferredoxin, 3%		0.37
2803	TC326562	UP|Q9VN28_DROME (Q9VN28) CG14650-PA, 3%		0.53
2833	TC325375	UP|Q9SAP9_MAIZE (Q9SAP9) Transposable element Ac, 9%		-0.34
2924	TC363572	UP|Q5ZDX8_ORYSA (Q5ZDX8) Heterogeneous nuclear ribonucleoprotein A2/B1-like, 83%		1.06
2981	TC357019	UP|Q9VFQ3_DROME (Q9VFQ3) CG9930-PA, 4%		0.92
3042	TC347535	UP|Q3CML1_ALTAT (Q3CML1) Sugar transporter precursor, 4%		-0.71
3066	TC322392	UP|Q9FTG9_ORYSA (Q9FTG9) Taxadienol acetyl transferase-like, 86%		-0.43
3079	-	-		-0.63
3084	-	-		-0.53
3092	TC318654	UP|Q9LL87_MAIZE (Q9LL87) Beta-glucosidase aggregating factor, 13%		0.72
3094	TC326274			0.70
3120	TC330893	UP|Q5Z8P6_ORYSA (Q5Z8P6) Transcription factor-like, 66%		-0.43

3126	TC359967	UP|Q5QJ60_CYNDA (Q5QJ60) FAD-linked oxidoreductase BG60, 20%		-0.82
3140	TC355717			-0.54
3145	TC352422	UP|Q9FKE6_ARATH (Q9FKE6) Similarity to cyclin, 4%		-0.35
3148	TC341695	UP|Q69TF7_ORYSA (Q69TF7) Subtilase-like, 6%		-0.28
3156	TC351275	UP|Q5MGQ8_PENAM (Q5MGQ8) Transcription factor DREB2A, 78%		-0.42
3180	TC338114	UP|SAPK7_ORYSA (Q7XQP4) Serine/threonine-protein kinase SAPK7 (Osmotic stress/abscisic acid-activated protein k		-0.49
3200	TC325987	UP|Q9SAK4_ARATH (Q9SAK4) T8K14.14 protein, 5%		-0.66
3210	TC345702	UP|Q2JGX9_FRASC (Q2JGX9) Peptidase S1 and S6, chymotrypsin/Hap, 3%		0.64
3233	TC316958	Zea mays clone Contig969.F mRNA sequence		0.79
3341	TC326117	RF|XP_507202.1|51964836|XM_507202 P0488B06.44 gene product {Oryza sativa (japonica cultivar-group)}, 76%		-0.76
3364	TC330874	UP|Q75J20_ORYSA (Q75J20) Expressed protein, 59%		-0.96
3373	TC332928	GB|AAB72097.1|2465428|AF021257 32 kDa protein {Hordeum vulgare subsp. vulgare}, 34%		1.00
3438	TC345634	UP|Q3HRP1_ORYSA (Q3HRP1) Calcineurin B-like protein 6, 73%		0.59
3445	TC347011			-0.61
3472	-	-		0.96
3475	TC357151	UP|Q8L685_VOLCA (Q8L685) Pherophorin-dz1 protein precursor, 6%		1.07
3525	TC349063	UP|Q41521_WHEAT (Q41521) Triticum aestivum sulfur-rich/thionin-like protein, 17%		-0.51
3526	TC342721	UP|Q40W39_KINRA (Q40W39) UBA/THIF-type NAD/FAD binding fold:MoeZ/MoeB, 6%		-0.51
3552	TC342283	UP|Q9VHC0_DROME (Q9VHC0) CG16788-PA (LD23870p) (RNA-binding protein S1), 14%		0.51
3554	TC357441	UP|Q6DJL9_XENLA (Q6DJL9) MGC82047 protein, 8%		0.30

3561	TC317661	UP|Q94BW4_CINCA (Q94BW4) Type 2 ribosome-inactivating protein cinnamomin II precursor, 19%		-0.48
3563	TC348586	GB|AAA83618.1|1125842|U43375 Sulfatase domain protein protein 1 {Caenorhabditis elegans} , 3%		-0.48
3569	TC317193	Zea mays clone EL01N0519D06.c mRNA sequence		1.57
3578	TC335689			0.66
3638	TC320314	UP|O22812_ARATH (O22812) AT-hook DNA-binding protein, 45%		0.92
3656	TC359943	UP|Q9SQ55_SPIOL (Q9SQ55) Nuclear RNA binding protein A, 7%		1.02
3666	TC327042	UP|PYRG_SCHPO (O42644) CTP synthase , 20%		0.65
3718	TC328186	UP|Q441S7_SOLUS (Q441S7) Pseudouridine synthase, Rsu, 7%		-0.52
3749	-	-		0.81
3790	TC330854	UP|Q9FRZ8_ORYSA (Q9FRZ8) E2F homolog, 30%		-0.29
3828	TC337102	UP|Q5BZG2_SCHJA (Q5BZG2) SJCHGC06992 protein, 11%		-0.56
3841	TC333944	UP|Q9LU46_ARATH (Q9LU46) DEAD-box protein abstrakt, 90%		-0.34
3842	TC330270	UP|Q2HVE6_MEDTR (Q2HVE6) Zinc finger, C2H2-type, 40%		0.65
3897	-	-		-0.66
3898	TC354117	GB|AAA70046.1|902058|OSU29176 lipid transfer protein precursor {Oryza sativa}, 89%		-0.51
3905	TC353219			-0.43
3928	TC341598	GB|AAL16287.1|16226874|AF428357 At1g74450/F1M20_13 {Arabidopsis thaliana}, 21%		-0.43
3945	TC323965	UP|Q2QNZ9_ORYSA (Q2QNZ9) Expressed protein, 41%		-0.60
4059	TC318175	UP|Q7XY20_WHEAT (Q7XY20) Ribosomal protein L19, 89%		0.29
4139	TC350972			0.59
4202	TC328123	UP|Q8JHJ3_BRARE (Q8JHJ3) U2 small nuclear RNA auxiliary factor small subunit, 12%		0.39

4225	TC329760	UP|Q94L33_ARATH (Q94L33) Ania-6a type cyclin, 9%		-0.57
4231	TC337366			-0.80
4244	TC344774	UP|Q40W17_KINRA (Q40W17) Prolyl-tRNA synthetase, bacterial, 3%		-0.60
4248	TC317732	UP|Q9SDZ5_ORYSA (Q9SDZ5) Growth-regulating factor 1, 33%		-0.60
4312	TC323186	UP|Q5U1M6_ORYSA (Q5U1M6) Class III peroxidase 67 precursor, 70%		-0.44
4313	TC319654	UP|Q53M44_ORYSA (Q53M44) At2g43970/F6E13.10, 66%		0.74
4325	TC353450			-0.52
4354	TC339242	UP|Q8S566_ORYSA (Q8S566) Guanine nucleotide-exchange protein GEP1, 5%		-0.53
4358	TC342192			-0.51
4398	TC359004	RF|XP_804905.1|71404390|XM_799812 mucin-associated surface protein (MASP) {Trypanosoma cruzi strain CL Brener},		-0.46
4441	TC357882	UP|Q5U7K6_9POAL (Q5U7K6) Metallothionein-like protein, 98%		0.84
4446	TC343700	UP|NUKM_BRAOL (P42027) NADH-ubiquinone oxidoreductase 20 kDa subunit, mitochondrial precursor, 76%		0.79
4486	TC319627	UP|EXG2_SCHPO (Q10444) Glucan 1, 3-beta-glucosidase 2 precursor, 3%		-0.88
4570	TC329437	Zea mays clone Contig96 mRNA sequence		-0.48
4646	TC368305	UP|U2AF1_DROME (Q94535) Splicing factor U2af 38 kDa subunit, 7%		-0.89
4682	TC317310	UP|O81091_ORYSA (O81091) Aie2 protein, 54%		-0.78
4724	TC362689	UP|Q5TUQ4_ANOGA (Q5TUQ4) ENSANGP00000028695, 12%		-0.42
4738	TC342494	UP|Q84XF7_MALXI (Q84XF7) Integral membrane protein Nramp1, 12%		-0.89
4745	TC354165	UP|Q3I0N3_DACGL (Q3I0N3) P23, 65%		-0.42
4748	TC338723			-0.69

4754	TC354845	UP|Q8GSC4_TOBAC (Q8GSC4) DNA topoisomerase II, 9%		-0.73
4779	TC359998			0.57
4832	TC347576			0.94
4836	TC349665	UP|Q3H3A5_9ACTO (Q3H3A5) Uncharacterised conserved protein UCP005026, 3%		1.46
4837	TC332928	GB|AAB72097.1|2465428|AF021257 32 kDa protein {Hordeum vulgare subsp. vulgare}, 34%		1.29
4840	TC349422			0.71
4849	TC316136	Zea mays clone EL01N0372H11.d mRNA sequence		0.47
4857	TC363338			1.12
4862	TC359279			0.69
4909	TC366047	UP|Q5U7K6_9POAL (Q5U7K6) Metallothionein-like protein, 93%		0.95
4949	TC341432	UP|Q2IIH7_ANADE (Q2IIH7) PE-PGRS family protein, 6%		1.17
4954	TC342763	UP|Q9PF60_XYLFA (Q9PF60) Endo-1, 4-beta-glucanase, 16%		1.40
4989	TC350847	GB|BAD87975.1|57900486|AP003933 Abl tyrosine kinase-interacting-like protein {Oryza sativa (japonica cultivar-gr		1.19
4991	TC321419	RF|NP_188467.2|22331151|NM_112723 DNA binding {Arabidopsis thaliana}, 62%		0.52
5002	TC337178	UP|Q9SBM1_VOLCA (Q9SBM1) Hydroxyproline-rich glycoprotein DZ-HRGP precursor, 10%		0.68
5025	TC350333	UP|Q6TW83_9POXV (Q6TW83) ORF017 DNA-binding phosphoprotein (DNA binding phosophoprotein), 15%		0.66
5031	TC357780			1.36
5052	TC316466	Zea mays clone EL01N0372C04.c mRNA sequence		0.66
5168	TC337016	UP|Q3GT62_9ACTO (Q3GT62) Phosphoglycerate/bisphosphoglycerate mutase:RNase H, 4%		1.17
5173	TC325021	UP|Q9SBM1_VOLCA (Q9SBM1) Hydroxyproline-rich glycoprotein DZ-HRGP precursor, 6%		0.52

5221	TC340985	UP|Q42412_NICSY (Q42412) RNA-binding protein RZ-1, 69%		0.86
5429	TC348388	UP|Q294I1_DROPS (Q294I1) GA11316-PA, 3%		-0.83
5443	TC318748	UP|O24223_ORYSA (O24223) GF14-d protein, 97%		-0.67
5472	TC330232	UP|Q6NKV4_ARATH (Q6NKV4) At5g59410, 81%		-0.69
5502	TC333312			1.55
5539	TC348593			-0.56
5569	TC365583			0.58
5585	TC340240	UP|Q7XBA5_ORYSA (Q7XBA5) Drought-induced protein DI1, 36%		1.14
5646	TC324229	GB|AAF79875.1|8778876|AC000348 T7N9.15 {Arabidopsis thaliana}, 9%		0.95
5693	TC360799	UP|MAD32_ORYSA (Q8S151) MADS-box transcription factor 32 (OsMADS32), 42%		1.05
5836	TC332270	UP|Q5N8F0_ORYSA (Q5N8F0) MAPK activating protein-like, 4%		-0.53
5840	TC350331	GB|AAH13549.1|15488836|BC013549 trinucleotide repeat containing 5 {Mus musculus}, 5%		-0.38
5855	TC325355	RF|NP_198865.1|15242719|NM_123413 RNA binding {Arabidopsis thaliana}, 47%		-0.81
5867	TC339544	UP|Q4RPE5_TETNG (Q4RPE5) Chromosome 1 SCAF15008, whole genome shotgun sequence, 6%		-0.61
5870	-	-		-0.92
5871	TC346056			-0.90
5883	TC327706	UP|Q9FIR6_ARATH (Q9FIR6) Gb|AAD25674.1, 8%		-1.03
5929	TC357545			-0.71
5933	TC363275			-0.54
5939	TC347373			-0.79
5953	TC350386	UP|Q377E3_RHOPA (Q377E3) FoF1 ATP synthase, subunit I, 13%		-0.45

5956	TC357546	UP|Q2UJW3_ASPOR (Q2UJW3) Predicted protein, 3%		-0.65
5970	TC365235			-0.44
5989	TC338287			-0.52
5992	TC350101			-0.70
6050	TC347648	UP|Q9XFG7_WHEAT (Q9XFG7) Isoamylase 1, 5%		-0.71
6072	TC351723			-0.53
6110	TC357861	UP|Q6DDA7_XENTR (Q6DDA7) MGC89913 protein, 6%		-0.62
6181	TC329100	UP|Q7EYE2_ORYSA (Q7EYE2) WD-40 repeat protein-like, 5%	-0.53	-0.60
6203	TC367414			-0.38
6227	TC337735	UP|Q206M2_9ARAC (Q206M2) Major ampullate spidroin 1, 3%		-0.53
6235	TC369570	UP|Q69JJ2_ORYSA (Q69JJ2) Phosphatidylinositol 3-and 4-kinase family-like, 76%		-0.53
6241	-	-		-0.94
6242	TC339155	UP|O22705_ARATH (O22705) F8A5.19 protein, 23%		-0.98
6244	TC345140			-0.86
6291	TC331455	Zea mays clone EL01N0419B11.d mRNA sequence		-0.77
6309	TC364458	UP|Q9LR84_ARATH (Q9LR84) F21B7.1, 14%		-0.59
6313	TC352180	UP|Q5RL92_TRYCR (Q5RL92) Type IB topoisomerase small subunit, 7%		-0.54
6314	TC331624	UP|Q9M9F8_ARATH (Q9M9F8) F3F9.7, 8%		-0.60
6348	TC356096	GB|AAK95275.1|15294196|AF410289 At1g74880/F9E10_27 {Arabidopsis thaliana}, 8%		-0.72
6350	TC342007			-0.55
6353	TC351188	UP|Q6T5M1_MAIZE (Q6T5M1) Mutant 19 kDa S15P alpha-zein, 77%		-0.91

6358	TC316726	Zea mays clone Contig130 mRNA sequence		-0.81
6359	TC323761	RF|NP_496363.1|17533631|NM_063962 LEThal family member (let-858) {Caenorhabditis elegans}, 3%		-0.76
6377	TC356299	UP|Q41734_MAIZE (Q41734) Cyclin IaZm, 8%		-0.38
6378	TC347823			-0.76
6421	TC335985	UP|Q9DUC7_9VIRU (Q9DUC7) ORF1, 3%		0.74
6434	TC326600	RF|NP_179349.2|42569106|NM_127312 RNA binding {Arabidopsis thaliana}, 17%		0.53
6466	TC321900	GB|AAA19069.1|500730|U10402 C. elegans neuro d homolog protein 1 {Caenorhabditis elegans}, 8%		0.70
6478	TC357771	UP|Q93UB6_CARRU (Q93UB6) ATP synthase gamma subunit, 5%		0.93
6493	TC341857			0.59
6498	TC352127	RF|XP_507274.1|51964980|XM_507274 P0481F05.17 gene product {Oryza sativa (japonica cultivar-group)}, 75%		0.68
6515	TC358925	UP|Q3IVG1_RHOS4 (Q3IVG1) Poly (3-hydroxybutyrate) depolymerase, 5%		1.15
6542	TC339377	GB|AAC17422.1|3153821|AF062655 plenty-of-prolines-101 {Mus musculus}, 4%		0.68
6634	TC333502	UP|Q2RBF5_ORYSA (Q2RBF5) Expressed protein, 15%		0.69
6644	TC368618	RF|NP_179899.1|15227788|NM_127882 CYP96A1 heme binding {Arabidopsis thaliana}, 5%		-0.53
6645	TC316008	UP|ACT3_ORYSA (P17299) Actin-3		-0.55
6667	-	-		-0.94
6668	-	-		-0.94
6713	TC353696			0.87
6718	TC316711	Zea mays clone Contig266 mRNA sequence		0.71

6725	TC366040	RF|XP_507334.1|51965100|XM_507334 P0562A06.11 gene product {Oryza sativa (japonica cultivar-group)}, 73%		-0.77
6733	TC349890			-0.67
6762	TC355591	RF|NP_567410.1|18414065|NM_117446 hydrolase/ pyrophosphatase {Arabidopsis thaliana}, 46%		-0.54
6763	TC354797	RF|NP_180524.1|15227584|NM_128517 catalytic {Arabidopsis thaliana}, 33%		-0.86
6765	TC332940			-0.63
6797	TC335669			-0.32
6803	TC365265			0.99
7004	TC359185	UP|HD2B_MAIZE (Q9M4U5) Histone deacetylase 2b, 5%		-0.50
7012	TC340012	UP|Q45W71_ARAHY (Q45W71) Auxin-repressed protein, 79%		0.50
7030	TC333381	RF|NP_195678.1|15235982|NM_120129 DNA binding {Arabidopsis thaliana}, 7%	1.53	
7046	TC327016	RF|NP_188147.1|15232545|NM_112392 3-hydroxybutyryl-CoA dehydrogenase/ oxidoreductase {Arabidopsis thaliana}, 90%		-0.47
7176	TC316550	Zea mays clone EL01N0365C01.c mRNA sequence		-0.55
7259	TC338490	UP|Q84Z96_ORYSA (Q84Z96) Glutaredoxin protein family-like, 80%		1.12
7315	TC328186	UP|Q441S7_SOLUS (Q441S7) Pseudouridine synthase, Rsu, 7%		-0.45
7330	TC341760	Zea mays clone Contig727 mRNA sequence		0.47
7341	TC318428	Zea mays clone EL01N0360D09.c mRNA sequence		1.08
7385	TC357882	UP|Q5U7K6_9POAL (Q5U7K6) Metallothionein-like protein, 98%		0.26
7404	TC331697			-0.68
7430	TC342292			-0.64
7431	TC334459	GB|AAP37745.1|30725446|BT008386 At5g61156 {Arabidopsis thaliana}, 14%		-0.54

7441	TC344041	UP|Q6YWQ2_ORYSA (Q6YWQ2) Acidic 82 kDa protein-like, 10%		-0.45
7470	-	-		-0.67
7579	TC346806	UP|Q9B0N5_9GOBI (Q9B0N5) NADH dehydrogenase subunit 2, 15%		-0.33
7617	TC329306			-0.74
7618	TC329085	RF|XP_506513.1|51963608|XM_506513 P0503D09.102 gene product {Oryza sativa (japonica cultivar-group)}, 12%		-0.80
7682	TC341321	UP|Q6Y2W8_MAIZE (Q6Y2W8) GCK-like kinase MIK, 4%		-0.18
7804	TC353373	UP|NO12B_PEA (Q00665) Early nodulin 12B precursor (N-12B), 14%		-0.99
7824	TC342657	UP|Q2LXY5_SYNAS (Q2LXY5) Glutathione-regulated potassium-efflux system protein, 6%		-0.65
7841	TC352075	UP|Q9SCJ4_ARATH (Q9SCJ4) Kinesin-like protein, 7%		-1.00
7866	-	-		-1.10
7883	TC329581	RF|NP_566630.1|18402188|NM_112817 kinase {Arabidopsis thaliana}, 31%		-1.37
7889	TC360282	UP|DRTS_MAIZE (O81395) Bifunctional dihydrofolate reductase-thymidylate synthase [Includes: Dihydrofolate reduct		-1.14
7895	TC362211	UP|Q3GT93_9ACTO (Q3GT93) 2-oxoglutarate decarboxylase precursor, 3%		0.57
7901	TC361783			-0.77
7907	TC352169	UP|O22632_MAIZE (O22632) Nitrate-induced NOI protein, 18%		-0.40
7915	TC355870			-0.49
7947	TC339062	Zea mays clone Contig1004.F mRNA sequence		0.57
7969	TC331925	GB|AAP68268.1|31711824|BT008829 At5g47680 {Arabidopsis thaliana}, 14%		1.03
7970	TC338352	RF|XP_506552.1|51963632|XM_506552 P0625E02.103 gene product {Oryza sativa (japonica cultivar-group)}, 70%		-0.48
7993	TC320998	UP|Q7XBX3_ORYSA (Q7XBX3) Expressed protein, 78%		-0.76

8016	TC364590	UP|Q2QPC2_ORYSA (Q2QPC2) Expressed protein, 37%		0.75
8017	TC327342			0.35
8084	TC328621	UP|Q6VB66_HHV1R (Q6VB66) ORF_06L, 5%		0.67
8113	TC341545	UP|Q6Z5K5_ORYSA (Q6Z5K5) Sucrase-like protein, 32%		-0.36
8133	TC326117	RF|XP_507202.1|51964836|XM_507202 P0488B06.44 gene product {Oryza sativa (japonica cultivar-group)}, 76%		-0.46
8227	TC370481	UP|Q9LL87_MAIZE (Q9LL87) Beta-glucosidase aggregating factor, 13%		-0.45
8247	TC336584	UP|ZRP4_MAIZE (P47917) O-methyltransferase ZRP4, 67%		-0.70
8326	TC328258	RF|NP_194348.1|15236140|NM_118751 binding {Arabidopsis thaliana}, 59%		-0.63
8342	TC339295	GB|AAA39391.1|387397|MUSKTEPI2 epidermal keratin subunit I {Mus musculus}, 5%		0.35
8370	TC366053			-0.49
8375	TC355779	UP|Q94KD0_ARATH (Q94KD0) AT5g58470/mqj2_60, 5%		-0.51
8407	TC367145	UP|Q6VBJ3_CANGA (Q6VBJ3) Epa4p, 9%		0.68
8413	TC318654	UP|Q9LL87_MAIZE (Q9LL87) Beta-glucosidase aggregating factor, 13%		0.98
8509	TC367985	UP|Q2QMW0_ORYSA (Q2QMW0) Expressed protein, 93%		-0.59
8539	TC333114	Zea mays clone EL01N0526H03.c mRNA sequence		1.01
8558	TC325434			0.74
8625	TC336112	RF|NP_566035.1|18406715|NM_130078 phosphatidate cytidylyltransferase {Arabidopsis thaliana}, 9%		-0.25
8635	TC339920			-0.46
8640	TC334809	RF|XP_481420.1|50943785|XM_481420 chloroplast RNA processing protein-like {Oryza sativa (japonica cultivar-group		-0.57
8769	TC334408	GB|AAM08407.1|19919848|AF490590 Na+/H+ exchanger 6 {Arabidopsis thaliana}, 85%		-0.37

8772	TC362985	UP|MNB1A_MAIZE (P38564) Dof zinc finger protein MNB1A, 39%		-0.38
8775	TC359801	UP|Q27SS9_MASBA (Q27SS9) Alcohol dehydrogenase-like protein, 7%		-0.68
8874	TC326740	UP|Q4ZH87_ORYSA (Q4ZH87) DsRNA binding protein RBP, 52%		-0.42
8967	TC333031			-0.45
8988	TC325809	RF|NP_178812.1|15226146|NM_126771 ATP binding {Arabidopsis thaliana}, 28%		-0.78
8997	TC317193	Zea mays clone EL01N0519D06.c mRNA sequence		-0.97
9156	-	-		-0.81
9171	TC347659	UP|Q93W87_ARATH (Q93W87) AT3g05760/F10A16_5, 40%		-0.44
9179	TC355990	UP|Q8LPK4_ARATH (Q8LPK4) Alpha-adaptin C-like protein, 10%		-0.43
9226	TC360968	gb|AF036494.1|AF036494 Eucryphia lucida large subunit 26S ribosomal RNA gene, partial sequence, 11%	1.01	-0.78
9247	TC353132	GB|AAQ07403.1|33323347|AF499137 synaptopodin {Homo sapiens}, 3%		0.97
9261	TC352034			-0.65
9308	TC364971	UP|Q6EPG8_ORYSA (Q6EPG8) Flavonoid 7-O-methyltransferase-like, 43%		-0.68
9354	TC354153	UP|Q6BWL2_DEBHA (Q6BWL2) Debaryomyces hansenii chromosome B of strain CBS767 of Debaryomyces hansenii, 8%		-0.71
9446	TC344554	UP|Q72FA5_DESVH (Q72FA5) Transcriptional regulator, LysR family, 10%		0.89
9456	-	-		-1.05
9565	TC360292			-0.76
9576	TC331255	UP|RF2B_ORYSA (Q6S4P4) Transcription factor RF2b, 66%		-1.16
9671	TC326408			-0.51
9677	TC322680	RF|XP_506692.1|51963818|XM_506692 OSJNBb0088N06.21 gene product {Oryza sativa (japonica cultivar-group)}, 66%		0.68

9696	TC323565	UP|O65836_LYCES (O65836) P69F protein, 41%		-0.36
9737	TC357019	UP|Q9VFQ3_DROME (Q9VFQ3) CG9930-PA, 4%		0.53
9778	TC339942			-0.46
9802	TC340119	UP|Q2QTQ5_ORYSA (Q2QTQ5) Expressed protein, 37%		-0.61
9885	TC331270			0.58
9888	TC347513	UP|Q4S810_TETNG (Q4S810) Chromosome 9 SCAF14710, whole genome shotgun sequence., 5%		-1.00
9922	TC358551	UP|Q9FRF8_ORYSA (Q9FRF8) Expressed protein, 28%		-0.45
9925	TC352783	UP|Q1W7L7_ELAGV (Q1W7L7) Palmitoyl-ACP thioesterase, 38%		-0.54
9927	TC340808	UP|Q5VQ77_ORYSA (Q5VQ77) Coatomer protein complex, beta prime subunit-like, 19%		-0.42
9941	TC323192	UP|RS10_ORYSA (Q9AYP4) 40S ribosomal protein S10, 98%		-0.67
9945	TC356883	UP|Q8Y7I1_LISMO (Q8Y7I1) Lmo1297 protein, 4%		-0.61
9965	TC323824	GB|CAB51171.1|5541665|ATT6H20 dUTP pyrophosphatase-like protein {Arabidopsis thaliana} , 85%		-0.41
9971	TC346806	UP|Q9B0N5_9GOBI (Q9B0N5) NADH dehydrogenase subunit 2, 15%		-0.71
9977	TC356635	UP|RK16_MAIZE (P08528) Chloroplast 50S ribosomal protein L16		-0.81
10018	TC335804	Zea mays clone Contig94.F mRNA sequence		-0.43
10067	TC360879	UP|Q2KDZ5_RHIEC (Q2KDZ5) Translation initiation factor IF-2 protein, 3%		-0.74
10103	TC341385	UP|Q6PSU8_ARATH (Q6PSU8) Formin homology 2 domain-containing protein 5, 3%		-0.58
10114	TC346321	UP|Q6K246_ORYSA (Q6K246) Loricrin-like, 35%		-0.62
10193	TC333493	UP|Q8IC04_PLAF7 (Q8IC04) Heat shock protein 86 family protein, 3%		-0.27
10244	TC333279	UP|Q25Y68_MYCVN (Q25Y68) Primosomal protein N' (Replication factor Y)-superfamily II helicase-like precursor, 3%	0.89	-0.58

10248	TC334778			-0.60
10280	TC353921	UP|CS029_HUMAN (Q8WUQ7) Protein C19orf29 (NY-REN-24 antigen), 4%		-0.49
10282	TC339405			-0.67
10296	TC347256	UP|Q40553_TOBAC (Q40553) N.tabacum mRNA pNLA-28, 53%		-0.80
10328	TC338432	UP|Q9SN46_ARATH (Q9SN46) Extensin-like protein, 4%		-0.31
10417	TC327958	Zea mays clone EL01N0561B04.c mRNA sequence		0.68
10778	TC356846	PRF|1211356A|225315|1211356A zein gamma. {Zea mays} , 28%		-0.43
10812	TC340673	UP|Q29AF1_DROPS (Q29AF1) GA11058-PA, 6%		0.76
10829	TC324490			-0.56
11044	TC342338			-0.41
11126	TC340169	UP|Q4B2U1_9BURK (Q4B2U1) Enoyl-CoA hydratase/isomerase, 5%		-1.10
11174	TC316398	Zea mays clone EL01N0323A09.c mRNA sequence		-0.39
11220	TC327557	Zea mays clone EL01N0450G11.d mRNA sequence		-0.52
11329	TC327463	RF|XP_506904.1|51964240|XM_506904 P0666E12.10 gene product {Oryza sativa (japonica cultivar-group)}, 53%		0.53
11385	TC329437	Zea mays clone Contig96 mRNA sequence		-0.42
11401	TC359889	UP|Q8DB68_VIBVU (Q8DB68) Chemotaxis protein, 3%		-0.72
11424	TC317926			-0.78
11435	TC319393	UP|Q9BIU2_9ARAC (Q9BIU2) Fibroin 1, 13%		-0.52
11441	TC320318	UP|Q8H5Q5_ORYSA (Q8H5Q5) Organic solute transporter-like, 75%		-1.49
11442	TC352776	UP|Q3JV00_BURP1 (Q3JV00) Phosphoglycerate mutase family protein, 3%		-0.73
11471	TC321564	UP|Q7G7I8_ORYSA (Q7G7I8) Zinc finger transcription factor ZF1, 67%		-0.32

11478	TC368965	UP|Q7Z5F0_HUMAN (Q7Z5F0) MU-MB-2.76, 22%		-0.43
11482	TC345012			-0.60
11502	TC359432			-0.61
11569	TC352278	UP|Q40474_TOBAC (Q40474) Axi 1 protein, 11%		-0.86
11580	TC347272	RF|NP_174226.1|15218889|NM_102672 nucleotide binding {Arabidopsis thaliana}, 33%		-0.40
11623	TC324379	RF|NP_172164.1|15222202|NM_100556 DNA-directed RNA polymerase {Arabidopsis thaliana}, 41%		-0.50
11841	TC351292	GB|AAT68023.1|49618877|AY644637 caffeoyl-CoA O-methyltransferase {Oryza sativa (japonica cultivar-group)}, 91%		-0.71
11866	TC360932	RF|NP_914820.1|34906946|NM_189931 cysteine proteinase inhibitor Scb-like protein {Oryza sativa (japonica cultiva		-1.11
11872	TC363760	UP|Q69SJ5_ORYSA (Q69SJ5) NatC N(Alpha)-terminal acetyltransferase, Mak10 subunit-like, 32%		-0.77
11874	TC349939	UP|Q2UFJ1_ASPOR (Q2UFJ1) Predicted protein, 3%		-0.49
11903	TC346676			-0.30
11946	TC351190	UP|Q6AXW0_RAT (Q6AXW0) Cdca8 protein (Cell division cycle associated 8), 6%		-0.39
11971	TC331859	UP|Q9SC70_ORYSA (Q9SC70) Putitive Ser/Thr protein kinase, 42%		-0.59
12147	TC330175	RF|XP_506942.1|51964314|XM_506942 OJ1008_D06.15 gene product {Oryza sativa (japonica cultivar-group)}, 23%		1.10
12243	TC360585	UP|Q7YXH7_9ASCI (Q7YXH7) P1 protamine, 14%		-0.48
12264	TC330733	UP|Q7XTF6_ORYSA (Q7XTF6) OJ991214_12.11 protein, 90%		-0.77
12287	TC344989	UP|Q39JC1_BURS3 (Q39JC1) HAD-superfamily subfamily IB, PSPase-like, 6%	0.55	
12314	TC326426	UP|Q9J870_9NUCL (Q9J870) ORF65 p6.9 DNA binding protein, 37%		0.59
12355	TC345095	GB|AAA33533.1|168686|MZEZE22B 26.99 kd zein protein {Zea mays}, 45%		-0.32

12446	TC357440	UP|Q9LL87_MAIZE (Q9LL87) Beta-glucosidase aggregating factor, 13%		-0.50
12453	TC333515	UP|YB1E_SCHPO (P87179) Serine-rich protein C30B4.01c precursor, 21%		0.35
12594	TC332552	UP|Q7WED7_BORBR (Q7WED7) Branched-chain amino acid ABC transporter, permease protein, 5%		-0.26
12602	TC333402	UP|Q53N84_ORYSA (Q53N84) Expressed protein, 98%		-0.36
12721	TC358826	UP|Q6IMV8_ORYSA (Q6IMV8) Transposase, 10%		-0.34
12771	TC330074	UP|Q93ZM0_ARATH (Q93ZM0) AT3g18370/MYF24_8, 12%		-0.59
12794	TC341011			-0.66
12797	TC349111	RF|XP_507406.1|51979058|XM_507406 P0450A04.131 gene product {Oryza sativa (japonica cultivar-group)}, 12%		-0.85
12846	TC324229	GB|AAF79875.1|8778876|AC000348 T7N9.15 {Arabidopsis thaliana}, 9%		-0.25
12853	TC367668			-0.47
13041	TC365874			0.87
13086	TC333266	UP|Q26XN8_XANP2 (Q26XN8) Preprotein translocase SecG subunit, 8%		-0.27
13407	TC320322	UP|TKTC_MAIZE (Q7SIC9) Transketolase, chloroplast (TK) , 60%		1.07
13425	TC347115	Zea mays clone Contig383 mRNA sequence		0.75
13544	TC322043	UP|Q5UDB5_MAIZE (Q5UDB5) INDETERMINATE-related protein 1		-0.40
14205	TC338310			-0.29
14376	TC335325	Zea mays clone EL01N0554B02.c mRNA sequence		0.75
14663	TC345977	UP|Q6KA74_ORYSA (Q6KA74) Ankyrin repeat protein-like, 53%		-0.44
15116	TC322219	UP|Q5ZBG4_ORYSA (Q5ZBG4) Polygalacturonase-like, 57%		0.88
15157	TC317114	UP|Q941Y7_ORYSA (Q941Y7) RING finger-like protein, 75%		-0.41

	15204	TC349019			0.97
	15236	TC355503	UP|Q5C6Q1_SCHJA (Q5C6Q1) SJCHGC01964 protein, 13%		-0.37
	15326	TC348597			-0.42

Appendix S10. Validation of Maize cDNA microarray results by quantitative real-time PCR for (left) *Andropogon gerardii* and (right) *Sorghastrum nutans*. U: un-warmed; W: warmed; AMB: ambient precipitation; ALT: altered precipitation. SE's were calculated as described by Livak & Schmittgen (2001). Fold-change values were relative to the expression values of individual clones under the condition of un-warmed, ambient precipitation. The dots indicate the expression values from microarray analysis (missing for the clones CB380843, DV621372, DV622645, CD568792, CB833708, CD001262 under the conditions of U, AMB and W, AMB on 7/18/2006 for *A. gerardii*).

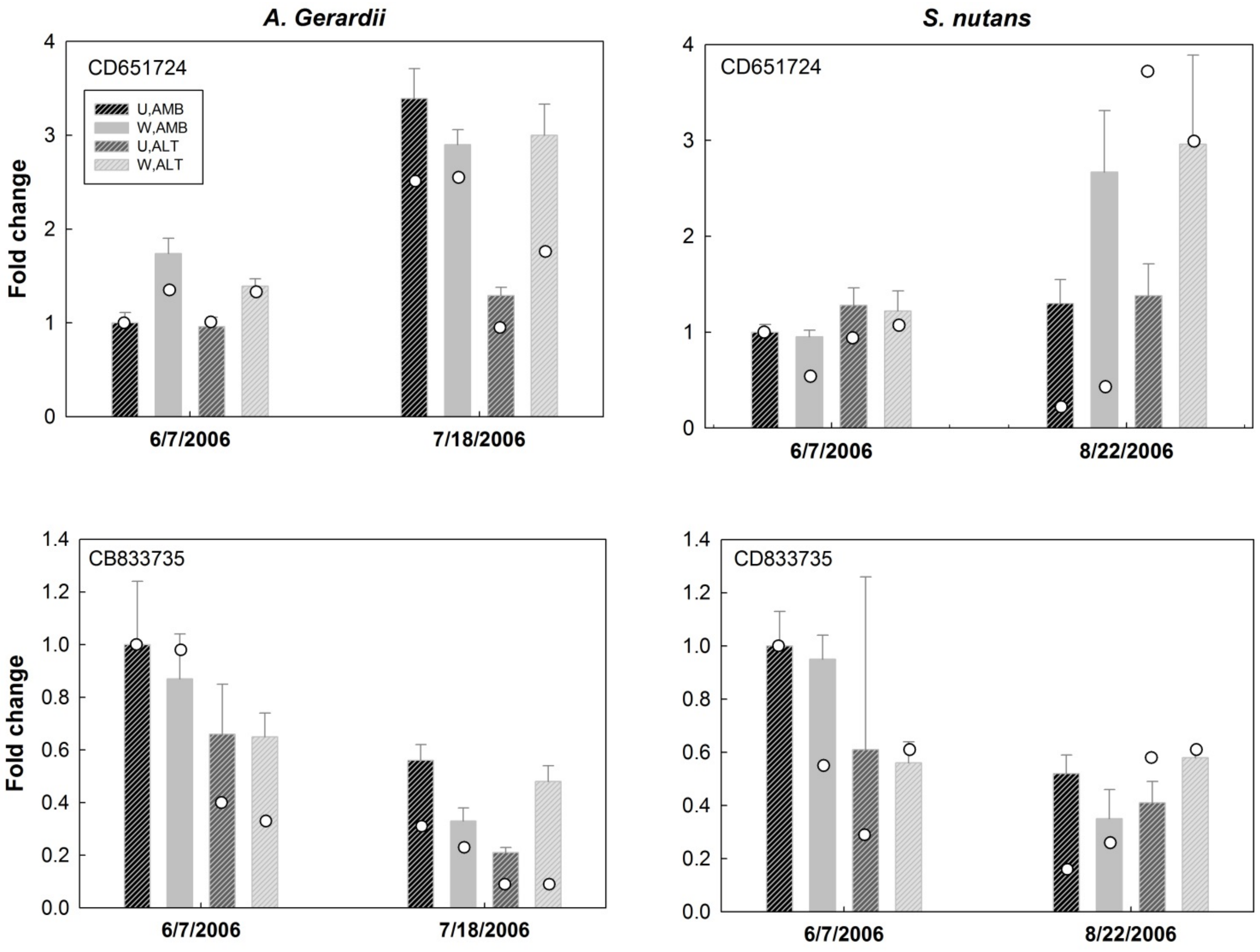

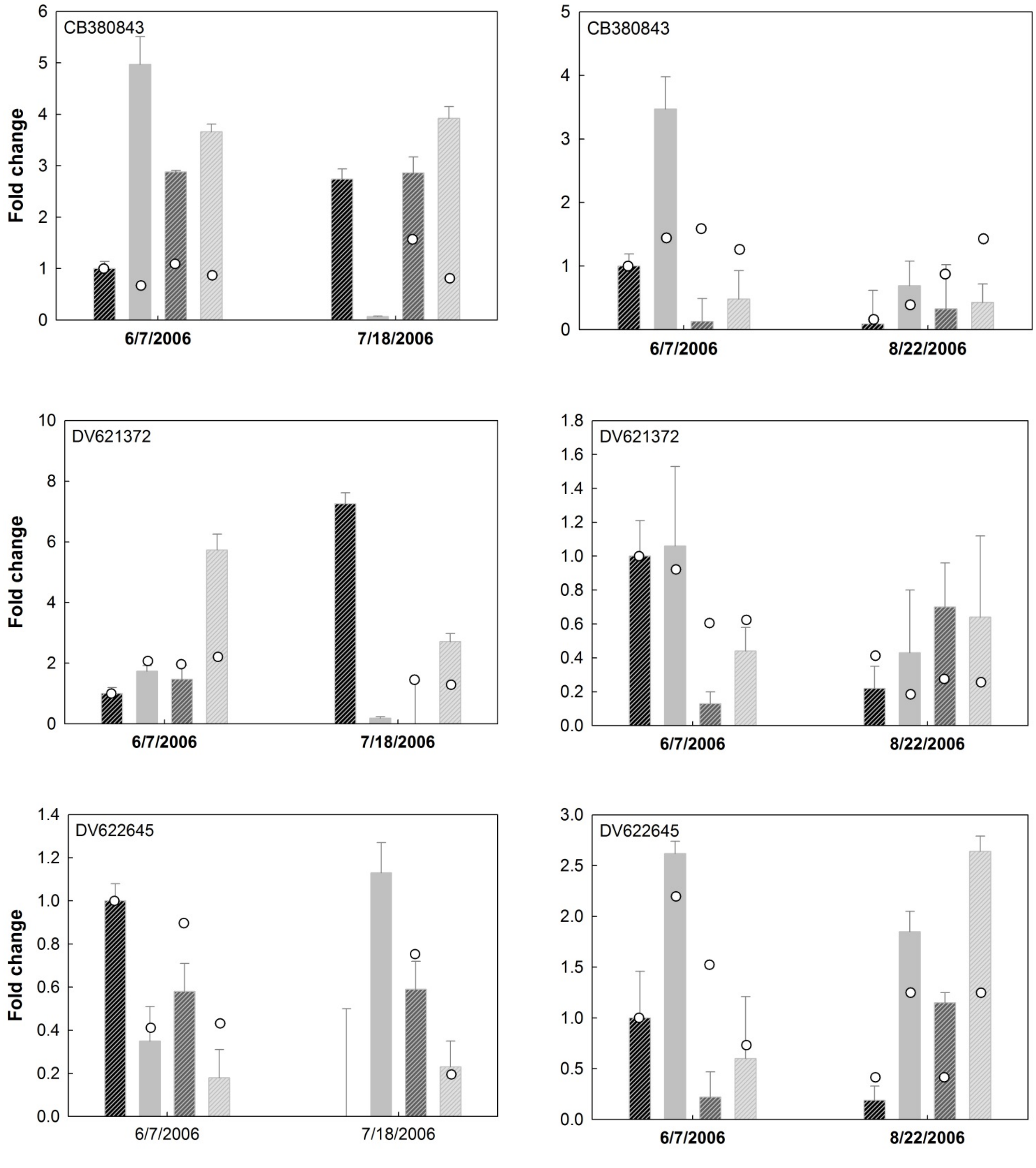
CB380843
Fold change
6/7/2006
7/18/2006
CB380843
6/7/2006
8/22/2006
DV621372
Fold change
6/7/2006
7/18/2006
DV621372
6/7/2006
8/22/2006
DV622645
Fold change
6/7/2006
7/18/2006
DV622645
6/7/2006
8/22/2006

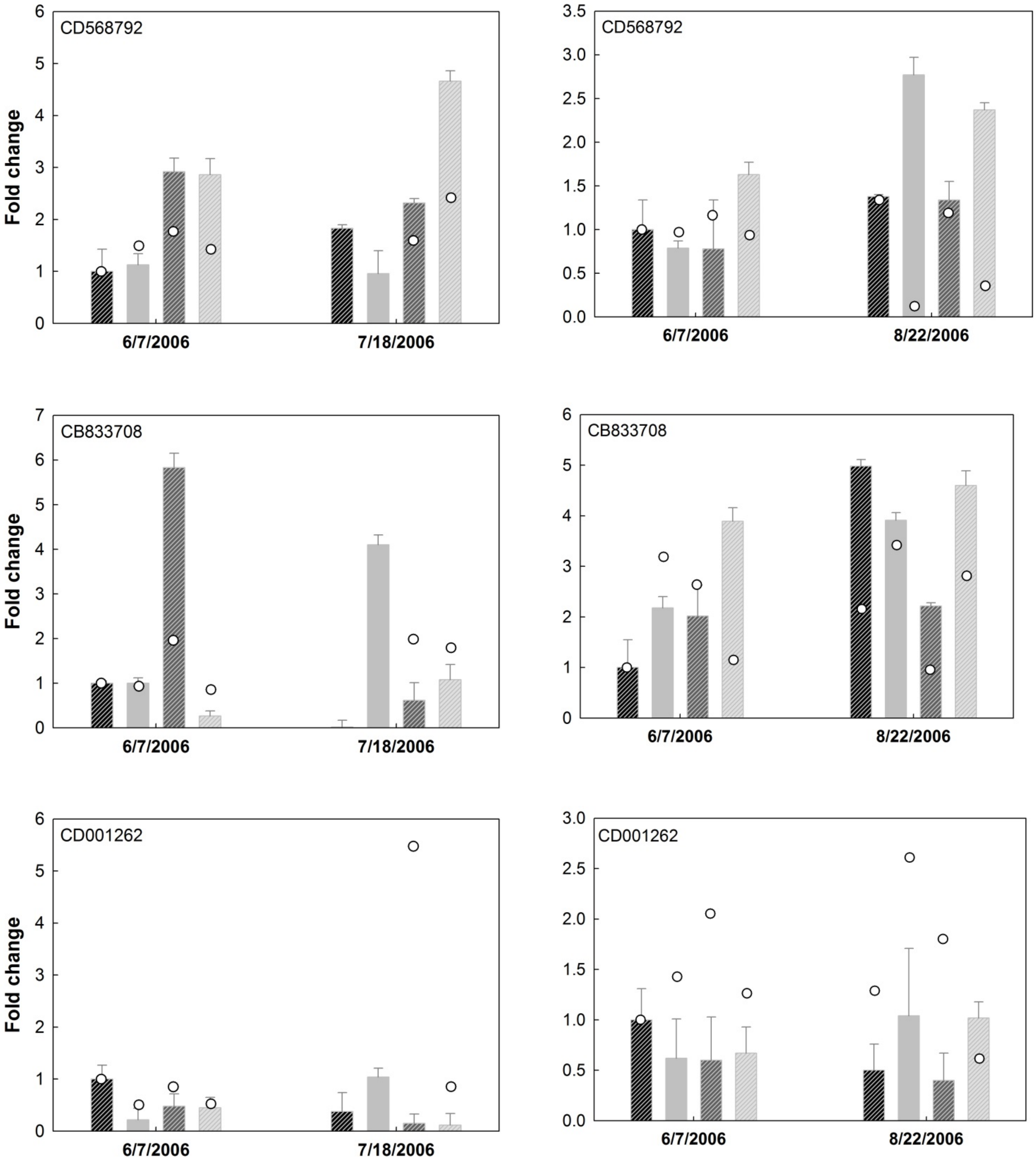
CD568792
Fold change
6/7/2006
7/18/2006
CD568792
6/7/2006
8/22/2006
CB833708
Fold change
6/7/2006
7/18/2006
CB833708
6/7/2006
8/22/2006
CD001262
Fold change
6/7/2006
7/18/2006
CD001262
6/7/2006
8/22/2006

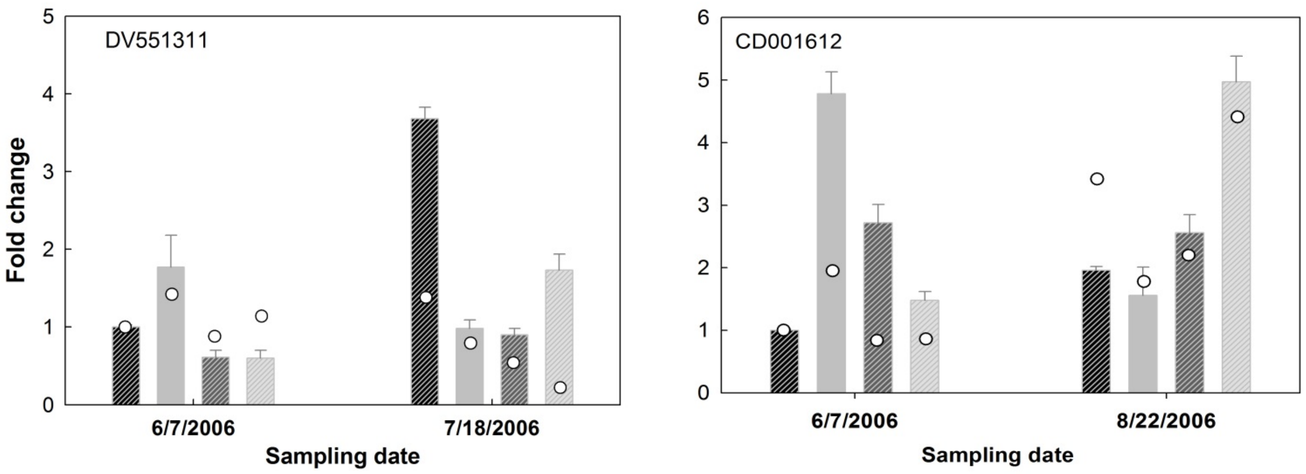
DV551311
Fold change
0
1
2
3
4
5
6/7/2006
7/18/2006
Sampling date
CD001612
0
1
2
3
4
5
6
6/7/2006
8/22/2006
Sampling date

Reference cited:

Livak, K.J. & Schmittgen, T.D. (2001) Analysis of relative gene expression data using real-time quantitative PCR and the 2 (-Delta Delta C(T)) method. *Methods*, **25**, 402-408.
